# Cu(ii)-mediated direct intramolecular cyclopropanation of distal olefinic acetate: access to cyclopropane-fused γ-lactones†

**DOI:** 10.1039/d3sc01752d

**Published:** 2023-05-25

**Authors:** Yulong Wang, Shenyu Shen, Chonglong He, Youkang Zhou, Keyuan Zhang, Bin Rao, Tian Han, Yaqiong Su, Xin-Hua Duan, Le Liu

**Affiliations:** a School of Chemistry, Engineering Research Center of Energy Storage Materials and Devices, Ministry of Education, Xi'an Key Laboratory of Sustainable Energy Material Chemistry, Xi'an Jiaotong University Xi'an 710049 China le.liu@xjtu.edu.cn

## Abstract

Cyclopropane-fused ring scaffolds represent one of the most appealing structural motifs in organic chemistry due to their wide presence in bioactive molecules and versatility in organic synthesis. These skeletons are typically prepared from olefinic diazo compounds *via* transition-metal catalysed intramolecular carbenoid insertion, which suffers from prefunctionalization of starting materials and limited substrate scope. Herein, we disclose a practical copper-mediated direct intramolecular cyclopropanation of distal olefinic acetate to synthesize cyclopropane-fused γ-lactones and lactams. This cascade reaction is postulated to proceed through a hydrogen atom transfer event induced radical cyclization and copper-mediated cyclopropanation sequence. The protocol features high atom- and step-economy, excellent diastereoselectivity, broad tolerance of functional groups, and operational simplicity.

## Abstract 2

A highly atom- and step-economy and diastereoselective copper-mediated direct intramolecular cyclopropanation method to synthesize cyclopropane-fused γ-lactones and lactams from distal olefinic acetate was disclosed.

## Graphical abstract

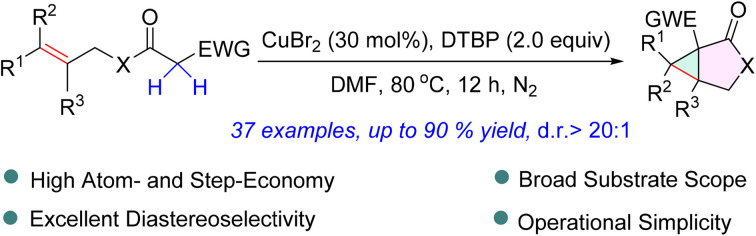

## Introduction

**Fig. 1:**
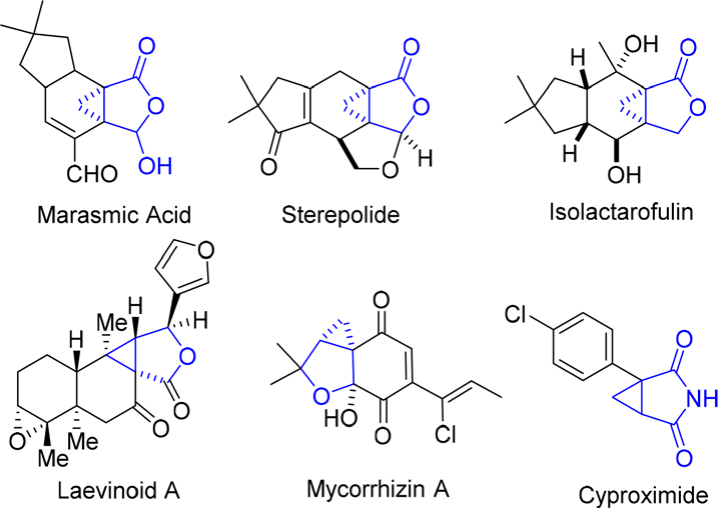
Selected naturally occurring cyclopropane-fused γ-lactones and lactams.

Cyclopropane represents one of the most appealing structural units with distinctive properties, containing three coplanar but highly strained π-character C–C bonds and stronger C–H bonds than common alkanes.^1^ These features made cyclopropane not only a highly attractive building block in synthetic chemistry,^2^ but also a valuable structure motif in medicinal chemistry. In particular, cyclopropane-fused γ-lactone and lactam units are prevalent in various bioactive naturally occurring products and drug molecules, which constitute key pharmacophores^3^ (Fig. 1).

**Scheme 1:**
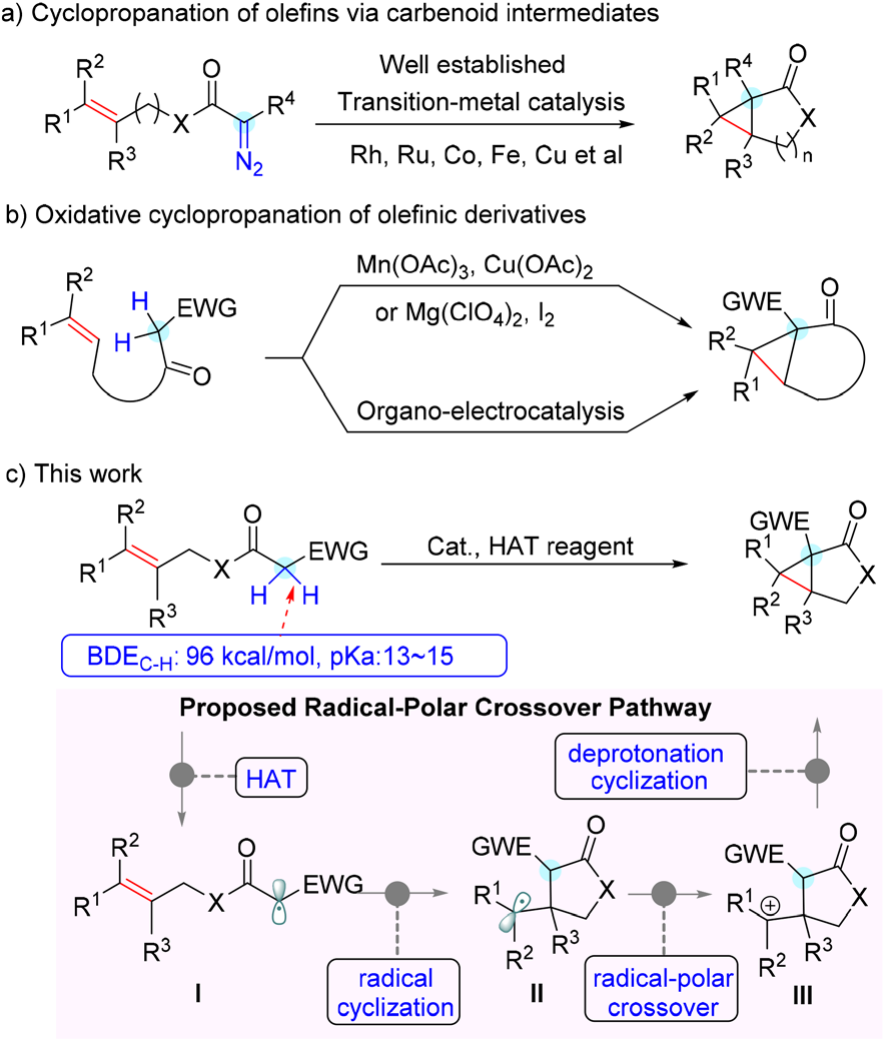
Examples of intramolecular cyclopropanation and our method.

Therefore, the chemical synthesis of cyclopropane-fused ring scaffolds has evoked considerable interest, and extensive efforts have been made in this field over the past few decades.^4^ Among the reported synthetic procedures, cyclopropanation of olefins with diazo compounds *via* metal-carbenoid intermediates represents one of the most frequently investigated methods. Robust variants of intramolecular cyclopropanation of olefins have been realized using allylic diazoacetates or diazoamides with transition-metal catalysts such as Rh, Ru, Co, Fe and Cu (Scheme 1a).^5^ Although they are efficient and provide a handle for accessing cyclopropane derivatives, the prefunctionalization required to install the diazo moiety and the potential hazardous properties of diazo compounds limit their further application. In contrast, direct intramolecular dehydrogenative annulation of distal alkenes represents an ideal approach from the atom- and step-economy point of view.^6^ Several elegant examples of this intramolecular oxidative cyclopropanation of alkenes were reported in the 1980s.^7^ However, stoichiometric amounts of Mn(OAc)_3_ or iodine were required for these transformations. Very recently, Xu and co-workers achieved such a transformation with an organo-electrocatalysis protocol, which provided convenient and efficient access to several types of cyclopropane-fused rings (Scheme 1b).^8^ In addition, reactions of ylides with electron-deficient alkenes^9^ and intermolecular cyclopropanation of allylic alcohols with aryldiazoacetates^10^ were also developed to build cyclopropane-fused skeletons. Despite these elegant achievements, it is still of great significance to develop concise and versatile methodologies to construct cyclopropane-fused rings from readily available starting substrates in a step-, atom-, and cost-economical fashion.

Recently, we have been interested in radical chemistry^11^ and heterocycle synthesis.^12^ We envisioned that the relatively weak bond dissociation energy (∼96 kcal mol^−1^) of the specific α–C–H bond of allyl acetate activated by an electron-withdrawing group would be abstracted by a suitable hydrogen atom transfer (HAT) reagent to generate the active radical intermediate (Scheme 1c, I). On the other hand, the rather acidic proton in the substrate would be easily deprotonated to engage in a nucleophilic addition. Thus, we hypothesized that a radical cyclization followed by a nucleophilic annulation *via* a radical-polar crossover process would be highly feasible to construct cyclopropane-fused rings (Scheme 1c). As the outcome of this effort, we report herein an efficient pathway for direct intramolecular alkene cyclopropanation *via* a Cu(ii)-mediated cascade process, which provides rapid and scalable access to cyclopropane-fused γ-lactones and-lactams.

Given the rather high O–H bond dissociation energy (BDE_O–H_ = 105 kcal mol^−1^) in alcohol as well as its ready availability, an alkoxyl radical derived from peroxide was selected as the HAT reagent to initiate the reaction. We probed the hypothesis by treating cinnamyl 2-(phenylsulfonyl)acetate 1aa with di-*tert*-butyl peroxide (DTBP) in the presence of CuBr. Gratifyingly, the expected cascade cyclopropanation reaction readily occurred to afford cyclopropane-fused lactone 2aa in a 64% isolated yield with high diastereoselectivity (Table 1, entry 1). Further optimization of the metal catalysts revealed that CuBr_2_ is superior to others (for details, see the ESI†), and the catalyst loading had a distinct influence on the outcome of this reaction. 30 mol% of CuBr_2_ is required to achieve a high 87% yield, while decreasing the loading of CuBr_2_ resulted in a dramatic drop in the yield (Table 1, entries 3–5). Other peroxides, including *tert*-butyl peroxybenzoate (TBPB), lauroyl peroxide (LPO), and dicumyl peroxide (DCP), were also surveyed, but none of them gave better results than DTBP (Table 1, entries 6 and 7). Heating was also important to this domino reaction, as conducting the reaction at a lower temperature led to a lower yield because of reduced substrate conversion (Table 1, entries 8 and 9). Presumably, heating benefits the conversion through several pathways, including facilitating the homolysis of peroxide, increasing mass transfer, and enhancing catalyst turnover.^12^ Further studies showed that polar solvents (CH_3_CN and DMSO) were tolerated well in the reaction, while less polar THF, DCE, or toluene were not compatible (Table 1, entries 10–12). Moreover, when the reaction was conducted in air, only trace 2aa was observed (Table 1, entry 13). It is worth mentioning that neither the Mn(OAc)_3_ mediated oxidative annulation^7*a*^ nor the halocyclization protocol^7*b*^ could efficiently produce the expected cyclopropane-fused γ-lactone 2aa from phenylsulfonylacetate 1aa, which highlighted the unique efficiency of our copper-catalysed reaction system.

**Table 1:** Optimization of the reaction conditions^a^

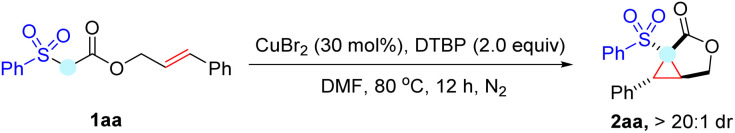		
Entry	Variation of reaction conditions	Yield of 2aa^b^ (%)
1^c^	CuBr instead of CuBr_2_	64
2^c^	CuI instead of CuBr_2_	71
3	None	87
4	20 mol% of CuBr_2_	61
5	10 mol% of CuBr_2_	22
6^c^	TBPB or LPO instead of DTBP	Trace
7^c^	DCP instead of DTBP	80
8^c^	Conducting at 40 °C	N.R.
9^c^	Conducting at 60 °C	81
10^c^	CH_3_CN as the solvent	83
11^c^	DMSO as the solvent	58
12^c^	THF, DCE or toluene	N.D.
13	Conducting in air	Trace

a Reaction conditions: 1aa (0.2 mmol, 1.0 equiv.), CuBr_2_ (30 mol%) and DTBP (0.4 mmol, 2.0 equiv.) in DMF (1 mL) stirred under N_2_ at 80 °C unless otherwise specified.

b Isolated yield.

c 40 mol% CuBr_2_ was employed.

With the optimized conditions in hand, we next studied a series of substituted cinnamyl acetates with different aryl sulfonyl groups to explore the reaction scope (Table 2). To our delight, all reactions of cinnamyl acetate with α-aryl sulfonyl groups bearing electron-donating and -withdrawing groups on the *para* and *meta* positions proceed smoothly and afford the expected products (2aa–2ai) in high yields with excellent diastereoselectivity (d.r. > 20 : 1).^13^ Halogens were well tolerated in the substrates, which enabled the possible further derivatization of the cyclopropane-fused γ-lactone by classical cross coupling methods. Overall, a minor effect on the reaction yield of *ortho*-substituted phenyl sulfonyl acetate was observed, and the expected products (2aj–2al) were obtained in slightly reduced yields, which might result from the steric hindrance of the substrates. In addition, we noticed that the reaction worked well by replacing the phenyl ring with a 2-naphthyl ring in the substrate to deliver product 2am in 88% yield under the standard conditions. It is noteworthy that heteroaryl bearing substituents such as pyridine (2an), thiophene (2ao) and benzothiazole (2ap) were also viable substrates, albeit with lower yields.

**Table 2:** Substrate scope investigation^a^

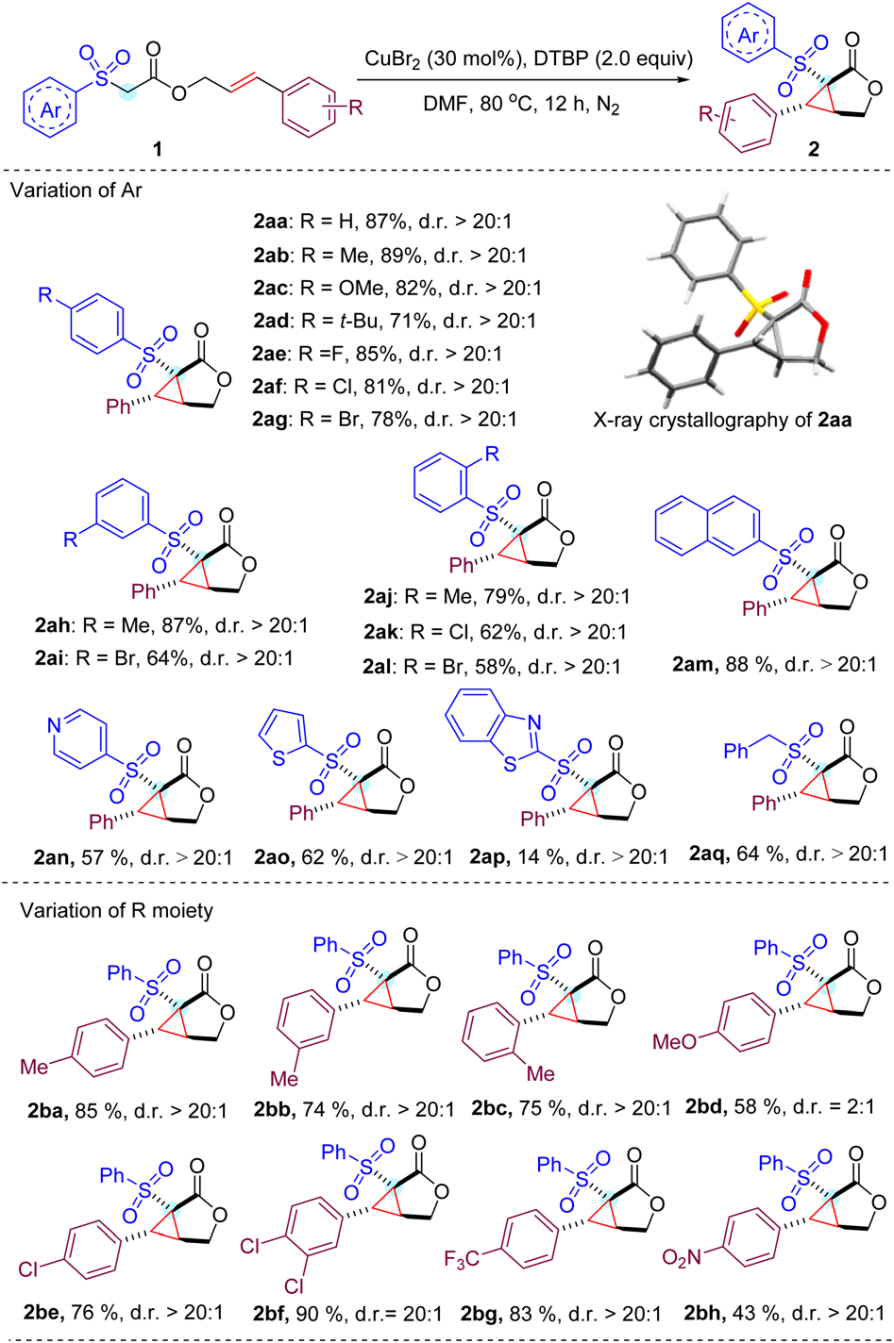

a Reaction conditions: 1 (0.2 mmol, 1.0 equiv.), CuBr_2_ (30 mol%) and DTBP (0.4 mmol, 2 equiv.) in DMF (1 mL) stirred under N_2_ at 80 °C and isolated yields are given. d.r. was determined by crude ^1^HNMR spectroscopy, for details see the ESI.

Then, the substituent effect on the cinnamyl moiety of the substrate was investigated. Methyl groups at the *para*, *meta*, and *ortho* positions did not significantly affect the reaction efficiency. In contrast, a strong electron-donating methoxy group at the *para* position of the cinnamyl phenyl ring lowered the yield as well as the diastereoselectivity (2bd). It is assumed that rather than participating in the cascade cyclization cycle, the relatively electron-rich benzylic radical involved in this transformation is more likely to be over oxidized to produce a number of unknown byproducts.^14^ Gratifyingly, 4-chlorophenyl, 3,4-dichlorophenyl and 4-trifluorophenyl substituents proved compatible with current reaction conditions, resulting in the corresponding products (2be–2bg) in good yields. Notably, the strong electron-withdrawing nitro group was also tolerated to provide product 2bh at 43% yield.

In addition to cinnamyl acetates, the CuBr_2_ mediated cyclopropanation was proven to be effective for allyl acetates (Table 3). However, the corresponding cyclopropane-fused γ-lactones (2bi–2bl) were generated in relatively lower yields. This is presumably due to the lower stability of the generated alkyl radical compared to the benzyl radical derived from the cinnamyl substrates. Notably, the steric effect in the allyl moiety likely played an important role in the diastereoselectivity of the reaction, as product 2bi with one methyl group was obtained in reduced 10 : 1 d.r., while both the phenyl and dimethyl substituted products (2aa and 2bj) were all obtained with excellent >20 : 1 diastereoselectivities. Alkylsulfonyl acetates were also capable of producing the corresponding cyclopropane-fused γ-lactones 2bm and 2bn with a moderate yield and good diastereoselectivities, demonstrating that the electron-withdrawing group (EWG) in the substituted acetates is not restricted to the arylsulfone unit. Additionally, the cyano, ketone, and ester groups were all compatible with the reaction conditions, resulting in the expected products (2bo–2br) being produced in moderate yields with good diastereoselectivities. It is worth mentioning that the electron-deficient aromatic system (4-nitrophenyl) was also tolerated to give the desired product in a synthetically useful 34% yield. However, phenyl or chloro substituted acetates (1bu and 1bv) were ineffective substrates under the standard conditions. This indicated that an appropriate second electron-withdrawing group is required for this copper-catalyzed cyclopropanation reaction. Presumably, it plays an important role in activating the protons of the substrates for deprotonation to engage in the cyclization cascade. We also succeeded in converting allyl acetamide into a cyclopropane-fused γ-lactam (2bt), albeit with a poor 30% yield. Unfortunately, the efficiency of current reaction conditions to prepare lactams is limited to terminal alkenes. Internal alkene 1bw only gave trace amounts of the expected products.

**Table 3:** Substrate scope investigation^a^

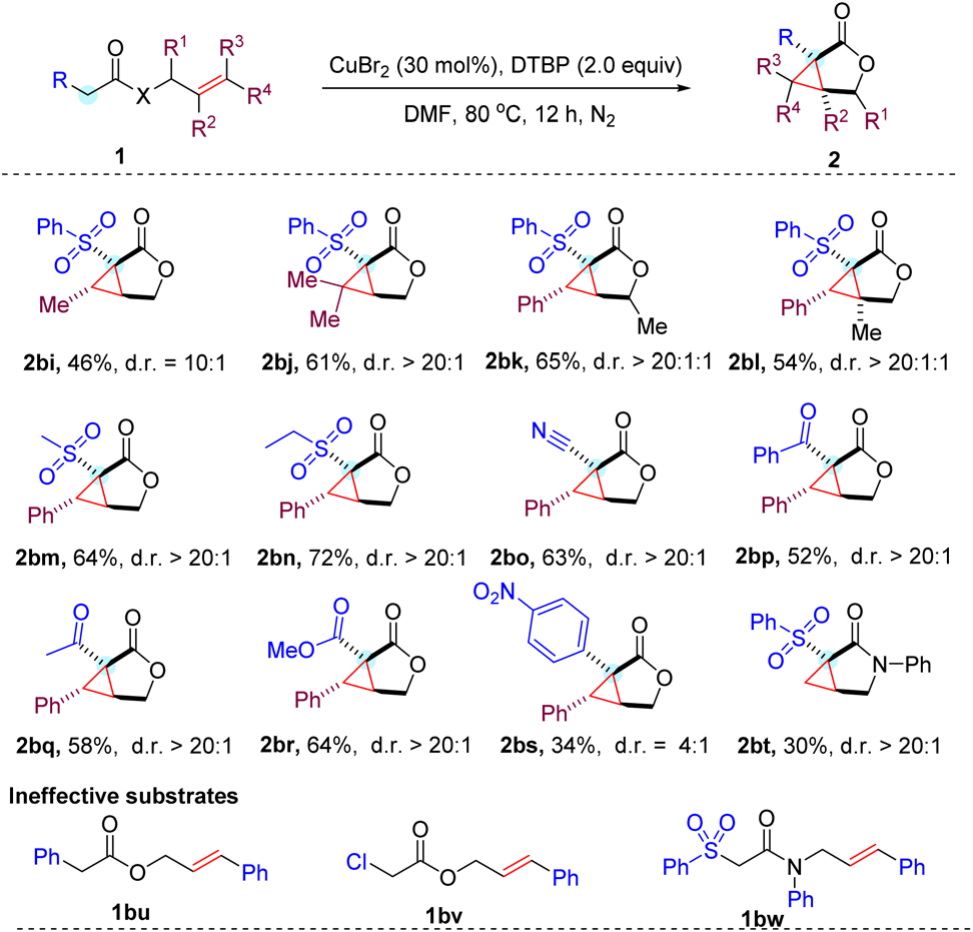

a Reaction conditions: 1 (0.2 mmol, 1.0 equiv.), CuBr_2_ (30 mol%) and DTBP (0.4 mmol, 2 equiv.) in DMF (1 mL) stirred under N_2_ at 80 °C and isolated yields are given. d.r. was determined by crude ^1^HNMR spectroscopy, for details see the ESI.

**Fig. 2:**
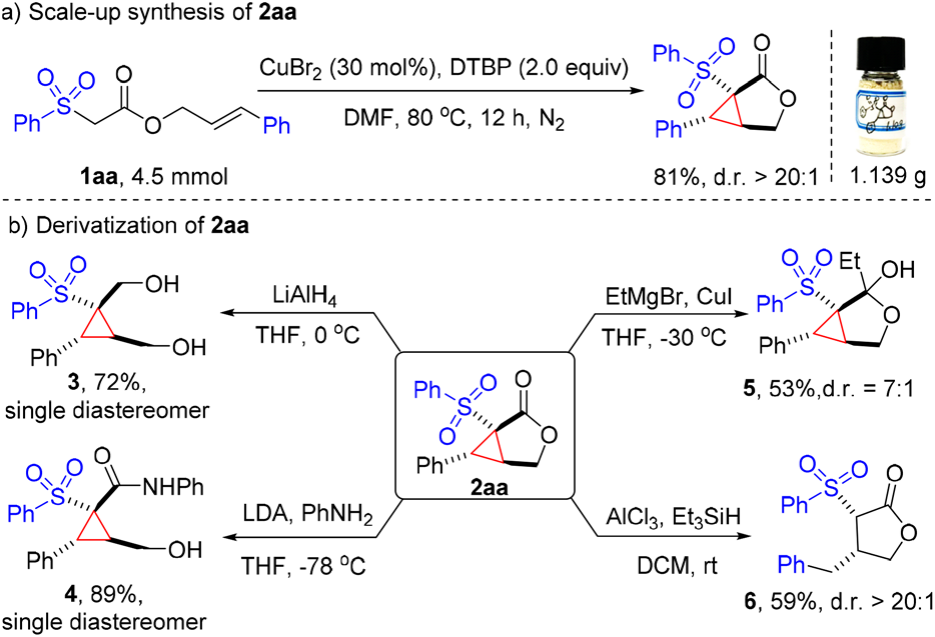
Scale-up synthesis (a) and derivatization of 2aa (b).

To illustrate the potential practicability of this methodology, a gram-scale (4.5 mmol) synthesis of cyclopropane-fused γ-lactone 2aa was conducted, and 81% yield (1.14 g) of the expected product was isolated with good diastereoselectivity (Fig. 2a). Remarkably, the cyclopropane-fused γ-lactone scaffold could serve as a versatile handle for further synthetic transformations to provide a wide variety of highly functionalized cyclic products. Reduction of 2aa with LiAlH_4_ afforded diol 3 with a 72% yield. Aminolysis of lactone 2aa with aniline easily generated the highly substituted cyclopropane 4 as a single diastereomer in a good 89% yield. Treatment of 2aa with EtMgBr in the presence of CuI provided hemiketal 5 in a moderate 53% yield. On the other hand, the strained cyclopropane ring can be deconstructed with Et_3_SiH under Lewis acid conditions, delivering the γ-lactone 6 in 59% yield (Fig. 2b).

**Fig. 3:**
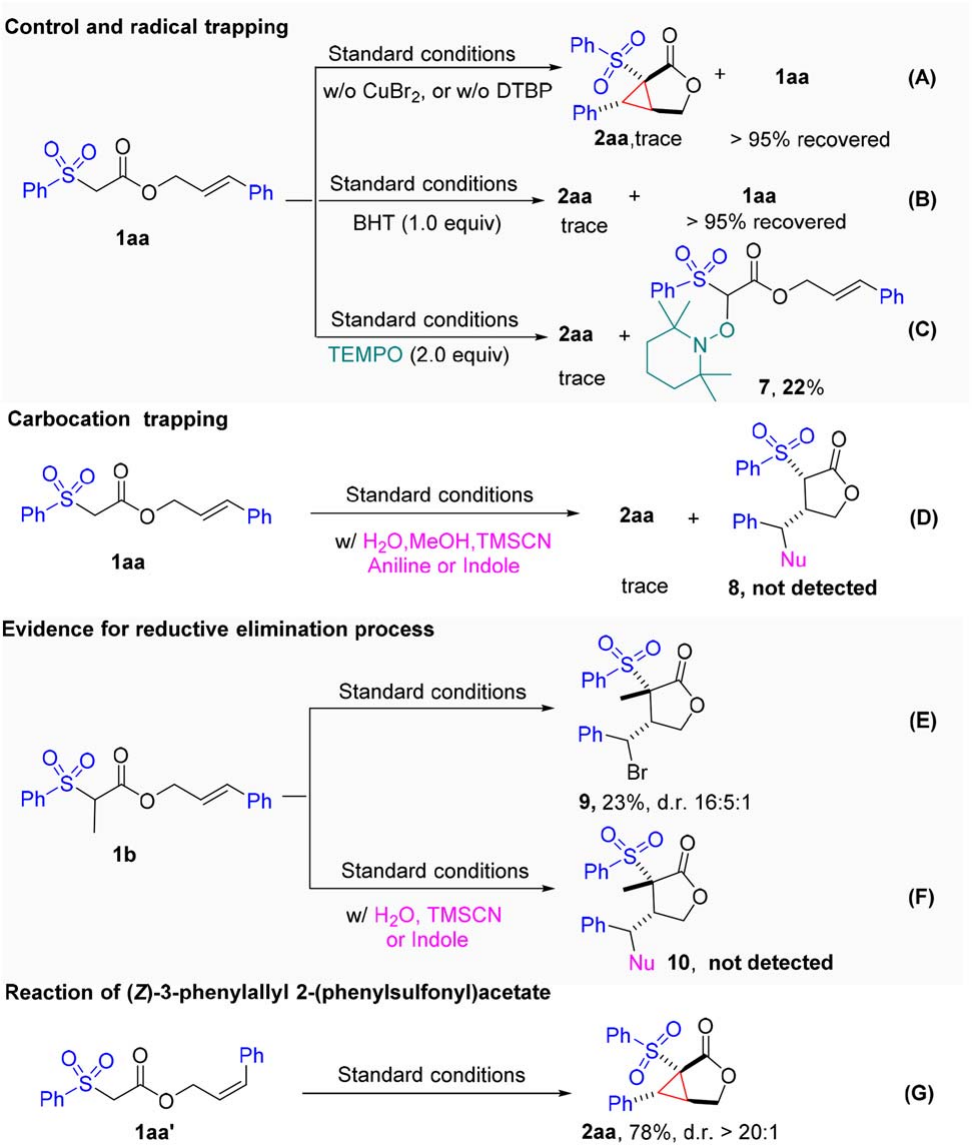
Control experiments and mechanism investigation (A-G).

To gain insight into the reaction mechanism, a series of control experiments were carried out (Fig. 3). First, the reaction completely shut down in the absence of either the peroxide (DTBP) or CuBr_2_, which indicated that both were crucial to this transformation (Fig. 3A). When a commonly used radical inhibitor, such as BHT or TEMPO, was introduced into the reaction, the cyclopropanation process was completely suppressed. Moreover, the TEMPO adduct 7 was isolated in 22% yield. These results indicated that a radical process might be involved in this transformation (Fig. 3B and C). To prove the involvement of the radical-polar crossover process in the cyclopropanation reaction, we next turned to capture the possible carbocation species under the standard conditions with various nucleophiles such as MeOH, H_2_O, TMSCN, aniline, and indole. Unfortunately, those attempts were unsuccessful, indicating that carbocation species were not likely involved (Fig. 3D). These results led us to speculate that the cyclopropane ring might be formed *via* another pathway rather than the initially proposed radical-polar crossover mechanism. Intriguingly, we found that when cinnamate 1b was subjected to the standard reaction conditions, the unexpected cyclized and bromo-substituted lactone 9 was obtained in a 23% yield (Fig. 3E). The addition of other nucleophiles led to suppression of the reaction, and no nucleophilic attached products formed (Fig. 3F). These results implied that the Br on lactone 9 might be introduced by reductive elimination from a hypervalent copper species. To determine whether the cyclopropanation process is stepwise or concerted, we examined the reaction of *cis*-allylic acetate 1aa′. The identical product, 2aa, was produced in a yield of 78% with excellent diastereoselectivity, suggesting that a stepwise mechanistic pathway may be at play (Fig. 3G).

**Scheme 2:**
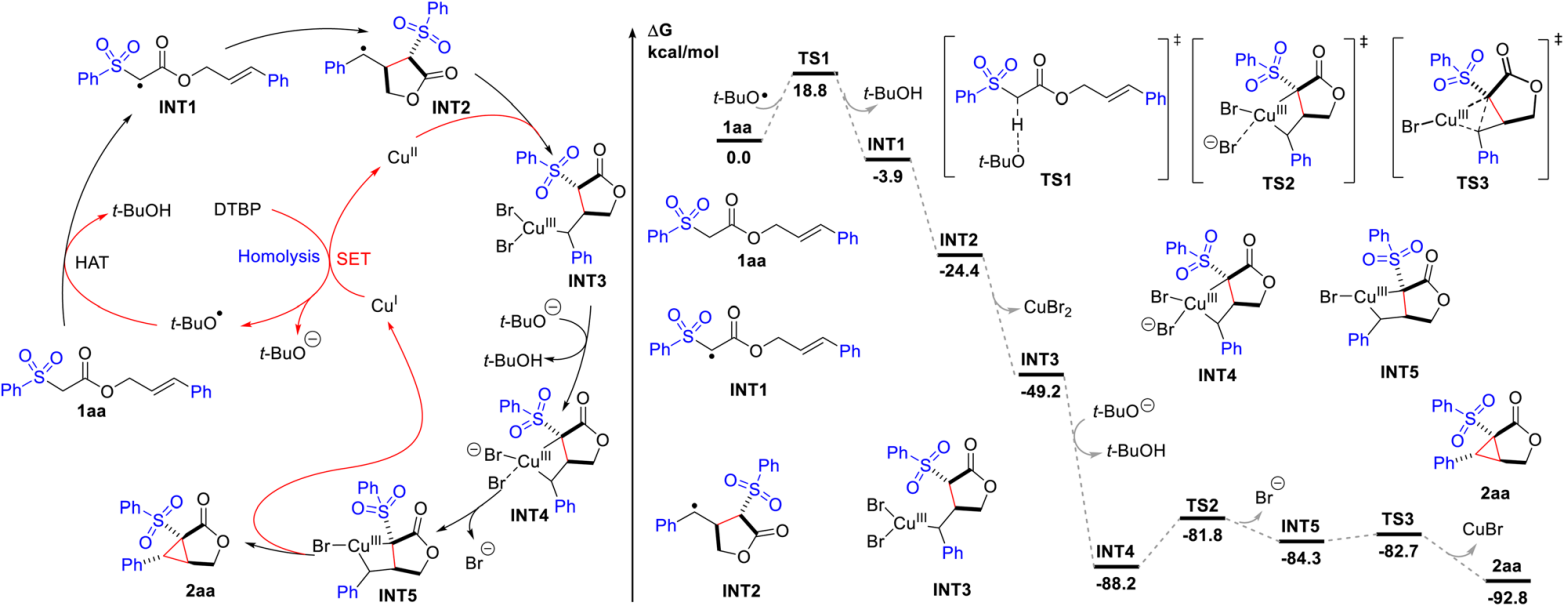
Proposed mechanism and computed energy profile for the Cu-catalysed intramolecular cyclopropanation of 2aa. Energies are in kcal mol^−1^ and bond distances are labelled in Å.

On the basis of these results and the literature,^15^ a reaction mechanism was proposed, as depicted in Scheme 2. And it was further analyzed by density functional theory (DFT) calculations to probe the energetics of the individual reaction steps. Initially, hydrogen atom transfer (HAT) from 1aa to the *tert*-butoxy radical generated from thermal homolysis of DTBP or Cu(i)-mediated SET reduction delivers the electrophilic radical INT1 (−3.9 kcal mol^−1^). The transition state of the HAT step is located as TS1, which has an energy barrier of 18.8 kcal mol^−1^. Intramolecular radical cyclization affords the benzyl radical INT2 (−24.4 kcal mol^−1^), which reacts with Cu(ii) species to produce the Cu(iii) species INT3 (−49.2 kcal mol^−1^). No transition state can be located suggesting that this cyclization process is concerted and spontaneous.^16^ The deprotonation of INT3 induces ligand exchange, leading to the formation of bicyclic INT4 (−88.2 kcal mol^−1^). The high exergonicity (39.0 kcal mol^−1^) of this step indicates that this process is very energetically favourable. Subsequently, bromide dissociates (TS2) with a barrier of 6.4 kcal mol^−1^, forming INT5 (−84.3 kcal mol^−1^). Finally, reductive elimination of INT5 gives the final cyclopropane-fused γ-lactone 2aa (−92.8 kcal mol^−1^), regenerating the reactive Cu(i) species. The transition state for this step is located as TS3, requiring only an energy barrier of 1.6 kcal mol^−1^. For substrate 1b which blocked the deprotonation and ligand exchange step (INT3 to INT4), direct reductive elimination affords bromolactone 9.

## Conclusions

In conclusion, we have developed a novel copper-catalyzed protocol to access cyclopropane-fused γ-lactones and lactams with good yields and diastereoselectivities. The practical advantages of this method include the use of readily available starting materials, the avoidance of prefunctionalized precursors of carbenes, broad substrate compatibility, and high diastereoselectivity. Further control experiments and DFT calculations indicated that the HAT event-induced radical cyclization and copper-mediated cyclopropanation cascade processes are likely operational. Given the ubiquity and intermediacy of cyclopropane-fused ring scaffolds in bioactive natural products and drugs, this efficient and scalable method would be of high interest to synthesis communities.

## Data availability

General information, detailed experimental procedures, characterization data for all new compounds, and NMR spectra are in the ESI.† Data for the crystal structure reported in this paper have been deposited at the Cambridge Crystallographic Data Centre (CCDC) under the deposition number CCDC 2254091.

## Author contributions

L. Liu conceptualized the project. L. Liu and X.-H. Duan supervised the investigation. Y. Wang, C. He, Y. Zhou and K. Zhang performed the research. S. Shen and Y. Su conducted the DFT calculations. B. Rao and T. Han analysed the X-ray structure of 2aa. L. Liu and Y. Wang co-wrote the paper. All authors analysed the data, discussed the results and commented on the manuscript.

## Conflicts of interest

There are no conflicts to declare.

## Supplementary Material

SC-014-D3SC01752D-s001

SC-014-D3SC01752D-s002
