# Supplementary material for: Cu(ii)-mediated direct intramolecular cyclopropanation of distal olefinic acetate: access to cyclopropane-fused γ-lactones
Source: Chem Sci. 2023 May 25;14(24):6663–8. doi: 10.1039/d3sc01752d (PMC10284120; doi:10.1039/d3sc01752d)
Supplement: SC-014-D3SC01752D-s001 [file SC-014-D3SC01752D-s001.pdf]

Electronic Supplementary Material (ESI) for Chemical Science.
This journal is © The Royal Society of Chemistry 2023

Cu(II)-mediated direct intramolecular cyclopropanation of distal olefinic acetate: access to cyclopropane-fused γ-lactone

Yulong Wang,[a] Shenyu Shen,[a] Chonglong He,[a], Youkang Zhou,[a] Keyuan Zhang,[a] Bin Rao,[a] Tian Han,[a] Yaqiong Su,[a] Xin-Hua Duan[a] and Le Liu*[a]

† *School of Chemistry, Engineering Research Center of Energy Storage Materials and Devices, Ministry of Education, Xi'an Key Laboratory of Sustainable Energy Material Chemistry, Xi'an Jiaotong University, Xi'an 710049, China.*

Supplementary Information

Table of Content

1. General information S3
2. Optimization of the reaction conditions S4
3. General procedure for the synthesis of starting materials S7
4. The general procedure 6 (GP6) for the synthesis of 2 S10
5. Scale-up synthesis of product **2aa** S11
6. Derivatization reactions of product **2aa** S11
   - 6.1 Synthesis of compound **3** S11
   - 6.2 Synthesis of compound **4** S12
   - 6.3 Synthesis of compound **5** S12
   - 6.4 Synthesis of compound **6** S13
7. Control experiments S13
   - 7.1 Comparing of this method with reported ones S13
   - 7.2 Radical trapping experiments S14
   - 7.3 Carbocation trapping S15
8. Characterization of products S16
9. Single-crystal X-ray diffraction data for **2aa** S41
10. Computational details S43
11. References S54
12. NMR spectra S56

1. General information

^{1}H NMR and ^{13}C NMR spectra were recorded on a Bruker Advance and JEOL III–400 spectrometer at 25 ^{o}C in solvents as indicated. Chemical shift values are reported in ppm with the solvent resonance refereed to the standard position ($CDCl_3$: ^{1}H NMR: δ = 7.26; ^{13}C NMR: δ = 77.16). The peak patterns are indicated as follows: s, singlet; d, doublet; t, triplet; q, quartet; m, multiplet and dd, doublet of doublets. The coupling constants *J* are reported in Hertz (Hz). HRMS were obtained on a QTOF micro spectrometer. Melting points were measured using open glass capillaries in SGW® X-4A apparatus. The fluorescent quenching experiment was conducted on a FL970 Plus Fluorescence Spectrometer.

All reactions were carried out in oven–dried Schlenk–tubes under an atmosphere of nitrogen. Unless otherwise stated, all the reagents were purchased from commercial sources and used without further purification. Conversion of the reactions was monitored by thin layer chromatography (TLC) using Merck TLC silica gel 60 F254. Compounds were visualized by UV light at 254 nm and by dipping the plates in an ethanolic phosphomolybdic acid solution followed by heating. Flash column chromatography was performed over silica gel (230-400 mesh). All the compounds were prepared according to reported method.

2. Optimization of the reaction conditions

Table S1. Solvents Screening[a]

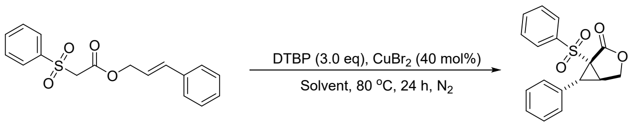

1aa **2aa**

Entry	Solvent	Yield of **2aa**[b] （%）	d.r.
1	MeCN	83	> 20:1
2	THF	N.D.	-
3	1,2-DCE	N.D.	-
4	DMSO	58	> 20:1
5	DMF	86	> 20:1
6	Toluene	N.D.	-

[a]Reaction conditions: **1aa** (0.2 mmol), DTBP (0.6 mmol) and $CuBr_2$ (40 mol%) in solvent (1.0 mL) under N_2 at 80 °C for 24 h. [b]Yield of isolated product. N.D. = Not Detected. 1,2-DCE = 1,2-dichloroethane. d.r. was determined by crude ^{1}HNMR spectroscopy.

Table S2. Effect of reaction time[a]

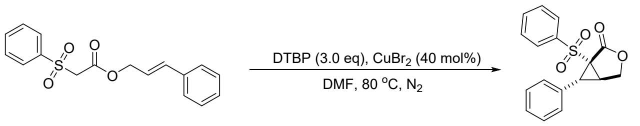

1aa **2aa**

Entry	Time	Yield of **2aa**[b] （%）	d.r.
1	6	72	> 20:1
2	12	87	> 20:1
3	18	87	> 20:1
4	24	86	> 20:1

[a]Reaction conditions: **1aa** (0.2 mmol), DTBP (0.6 mmol) and $CuBr_2$ (40 mol%) in DMF (1.0 mL) under N_2 at 80 °C. [b]Yield of isolated product. d.r. was determined by crude ^{1}HNMR spectroscopy.

Table S3. Effect of oxidants on the reaction[a]

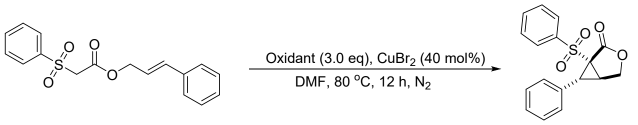

1aa **2aa**

Entry	Oxidant	Yield of **2aa**[b] (%)	d.r.
1	TBPB	Trace	-
2	BPO	N.D.	-
3	LPO	N.R.	-
4	TBHP	Trace	-
5	DCP	80	> 20:1
6	DTBP	87	> 20:1

[a]Reaction conditions: **1aa** (0.2 mmol), Oxidant (0.6 mmol) and $CuBr_2$ (40 mol%) in DMF (1.0 mL) under N_2 at 80 °C for 12 h. [b]Yield of isolated product. TBPB = *tert*-butyl peroxybenzoate; BPO = benzoyl peroxide; LPO = lauroyl peroxide; TBHP = tert-butyl hydroperoxide; DCP = dicumyl peroxide; N.D. = not detected. N.R. = no reaction. d.r. was determined by crude ^{1}HNMR spectroscopy.

Table S4. Effect of catalysts on the reaction

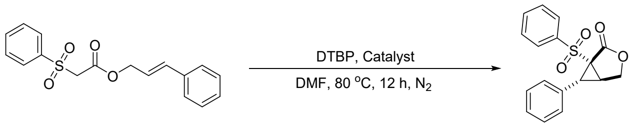

1aa **2aa**

Entry[a]	Catalyst	Yield of **2aa**[b] (%)	d.r.
1	$Cu(acac)_2$	8	-
2	$Cu(OTf)_2$	N.D.	-
3	$Cu(OAc)_2$	N.R.	-
4	$CuCl_2$	16	> 20:1
5	$CuBr_2$	87	> 20:1
6	CuBr	64	> 20:1
7	CuI	71	> 20:1
8	CuSCN	Trace	-
9	Cu_2O	Trace	-
10	$Fe(acac)_2$	N.R.	-
11	$Fe(OTf)_2$	N.R.	-
12	$Fe(OAc)_2$	N.R.	-
13	$FeCl_2$	N.R.	-
14	$FeBr_2$	N.R.	-
15	$Fe(acac)_3$	N.R.	-
16	$Fe(OTf)_3$	N.R.	-
17	$Fe(OTs)_3$	N.R.	-

[a]Reaction conditions: **1aa** (0.2 mmol), DTBP (0.6 mmol) and Copper salt (40 mol%) in DMF (1.0 mL) under N_2 at 80 °C for 12 h. [b]Yield of isolated product. [c]Reaction conditions: **1aa** (0.2 mmol), DTBP (0.4 mmol) and Ferric salt (20 mol%) in DMF (1.0 mL) under N_2 at 80 °C for 12 h. d.r. was determined by crude ^{1}HNMR spectroscopy.

Table S5. Effect of temperature on the reaction[a]

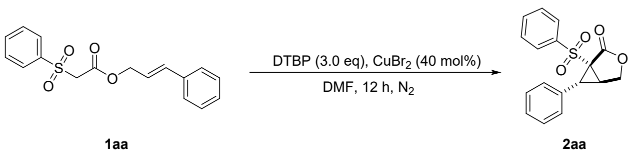

Entry	Temperature (°C)	Yield of **2aa**[b] (%)	d.r.
1	40	N.D.	-
2	60	81	> 20:1
3	80	87	> 20:1
4	100	87	> 20:1

[a]Reaction conditions: **1aa** (0.2 mmol), DTBP (0.6 mmol) and $CuBr_2$ (40 mol%) in DMF (1.0 mL) under N_2 for 12h. [b]Yield of isolated product. d.r. was determined by crude ^{1}HNMR spectroscopy.

Table S6. Reaction optimization[a]

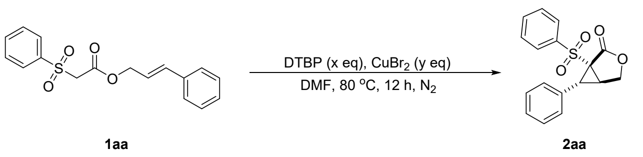

Entry	DTBP (x eq)	$CuBr_2$ (y eq)	Yield of **2aa**[b] (%)	d.r.
1	-	0.4	N.R.	-
2	1.5	0.4	71	> 20:1
3	2.0	0.4	89	> 20:1
4	2.5	0.4	88	> 20:1
5	3.0	0.4	87	> 20:1
6	3.5	0.4	87	> 20:1
7	2.0	-	N.R.	-
8	2.0	0.1	22	> 20:1
9	2.0	0.2	61	> 20:1
10	2.0	0.3	87	> 20:1

[a]Reaction conditions: **1aa** (0.2 mmol), DTBP (x eq) and $CuBr_2$ (y eq) in DMF (1.0 mL) under N_2 at 80 °C for 12 h. [b]Yield of isolated product. d.r. was determined by crude ^{1}HNMR spectroscopy.

3. General procedure for the synthesis of starting materials

3.1 Compound 1aa was prepared according to the general procedure 1 (GP1)

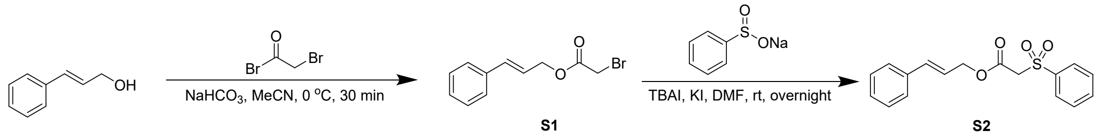

(1) Following the reported procedure,[1] to a solution of cinnamyl alcohol (10 mmol, 1.0 equiv) in acetonitrile (0.3 M) was added $NaHCO_3$ (15 mmol, 1.5 equiv) followed by the slowly addition of bromoacetyl bromide (12 mmol, 1.2 equiv) at 0 °C. After stirring 30 min at this temperature, the reaction was quenched with H_2O. The mixture was extracted with ethyl acetate (20 mL × 3). The combined organic layer was washed with brine (15 mL), dried over Na_2SO_4, filtered, and concentrated in vacuo. The crude product was purified by flash chromatography to afford **S1**.

(2) Following the reported procedure,[2] to a solution of **S1** (5 mmol, 1.0 equiv) in *N,N*-dimethylformamide (0.3 M) was added sodium benzenesulfinate (6 mmol, 1.2 equiv), tetrabutylammonium iodide (0.5 mmol, 0.1 equiv), potassium iodide (6 mmol, 1.2 equiv). The reaction mixture was stirred at room temperature overnight and then quenched with saturated aqueous $NaHCO_3$ (10 mL). The mixture was extracted with ethyl acetate (10 mL × 3). The combined organic layer was washed with brine (10 mL), dried over Na_2SO_4, filtered, and concentrated in vacuo. The crude product was purified by flash chromatography to afford **S2**.

3.2 Compounds 1ab - 1aq were prepared according to the general procedure 2 (GP2)

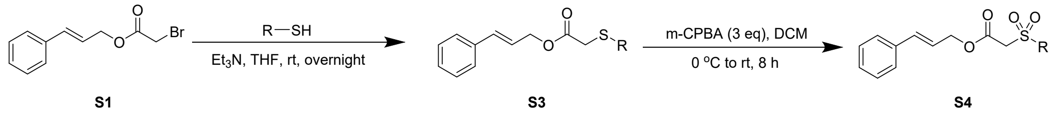

(1) Following the reported procedure,[3] to a solution of **S1** (5 mmol, 1.0 equiv) in tetrahydrofuran (0.3 M) was added thiophenol (5.0 mmol, 1.0 equiv) and triethylamine (10 mmol, 2.0 equiv). The reaction mixture was stirred at room temperature overnight and then quenched with saturated aqueous $NaHCO_3$ (10 mL). The mixture was extracted with ethyl acetate (15 mL × 3). The combined organic layer was washed with brine (15 mL), dried over Na_2SO_4, filtered, and concentrated in vacuo. The crude product was purified by flash chromatography to afford **S3**.

(2) Following the reported procedure,[4] to a solution of **S3** (5 mmol, 1.0 equiv) in dichloromethane (0.3 M) was added slowly 3-chloroperoxybenzoic acid (15 mmol, 3.0 equiv). Then the solution was allowed to warm to room temperature and stirred for 8 h.After completion, the reaction was quenched with saturated aqueous $NaHCO_3$ (10 mL). And the mixture was extracted with dichloromethane (15 mL × 3). The combined organic extract was washed with brine (15 mL), dried over Na_2SO_4 and concentrated in vacuo. The crude product was purified by flash column

chromatography (petroleum ether / ethyl acetate) to afford **S4**.[4]

3.3 Compounds 1ba - 1bh, 1bl were prepared according to the general procedure 3 (GP3)

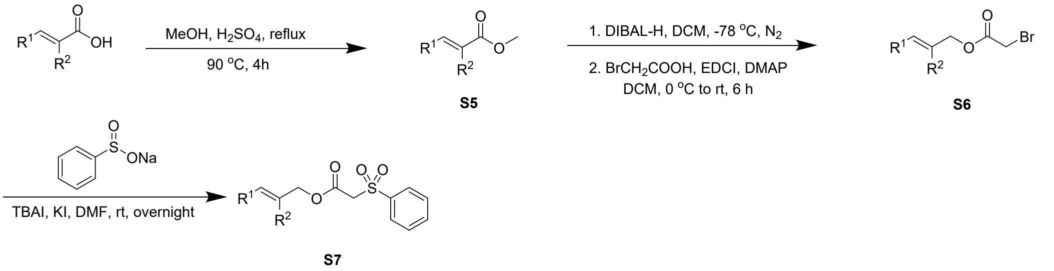

(1) Following the reported procedure,[5] to a solution of α,β-unsaturated carboxylic acid (5 mmol, 1.0 equiv) in methanol (0.5 M) was added H_2SO_4 (cat.). The reaction was stirred at 90 °C for 4 h then cooled to room temperature. And the reaction was quenched with saturated aqueous $NaHCO_3$ (10 mL). The mixture was extracted with ethyl acetate (15 mL × 3). The combined organic layer was washed with brine (15 mL), dried over Na_2SO_4, filtered, and concentrated in vacuo to afford **S5**, which was used without further purification unless stated.

(2) Following the reported procedure,[6] to a solution of methyl cinnamate (5 mmol, 1.0 eq.) in dry dichloromethane (0.5 M) was added DIBAL-H (10 mL, 10 mmol, 1.0 M solution in hexane, 2.0 eq.) at - 78°C dropwise. The reaction mixture was stirred for 2 h at - 78 °C and allowed to warm up gradually. Then the reaction mixture was cooled to 0 °C, and carefully quenched with saturated aqueous NH_4Cl (10 mL). The reaction mixture was stirred at room temperature for 1 h, and the resulting white precipitate was filtered through a pad of Celite®. The filtrate was extracted three times with dichloromethane (5 ml × 3). The organic phase was directly used for the next step.

Following the reported procedure,[7] to a solution of cinnamyl alcohol (5 mmol, 1.0 equiv) in dichloromethane (0.3 M) was added slowly bromoacetic acid (6 mmol, 1.2 equiv), EDCI HCl (6 mmol, 1.2 mmol) and 4-(dimethylamino)pyridine (0.5 mmol, 0.1 equiv) at 0 °C. Then the solution was allowed to warm to room temperature and stirred for 6 h. After completion, the reaction was quenched with saturated aqueous $NaHCO_3$ (15 mL) and the aqueous layer was extracted with dichloromethane (10 mL × 3). The combined organic extract was washed with brine (15 mL), dried over Na_2SO_4 and concentrated in vacuo. The crude product was purified by flash column chromatography (petroleum ether / ethyl acetate) to afford **S6**.

(3) To a solution of **S6** (5 mmol, 1.0 equiv) in N,N-dimethylformamide (0.3 M) was added sodium benzenesulfinate (6 mmol, 1.2 equiv), tetrabutylammonium iodide (0.5 mmol, 0.1 equiv) and potassium iodide (6 mmol, 1.2 equiv). The reaction mixture was stirred at room temperature overnight and then quenched with saturated aqueous $NaHCO_3$ (10 mL). The mixture was extracted with ethyl acetate (10 mL × 3). The combined organic layer

was washed with brine (10 mL), dried over Na_2SO_4, filtered, and concentrated in vacuo. The crude product was purified by flash chromatography to afford **S7**.

3.4 Compound 1bk was prepared according to the general procedure 4 (GP4)

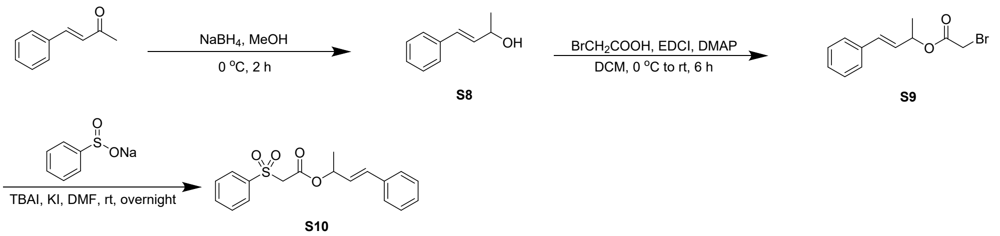

(1) Following the reported procedure,[8] to a solution of (*E*)-4-phenyl-3-buten-2-one (10 mmol, 1.0 equiv) in methanol (0.3 M) was added slowly sodium borohydride (15 mmol, 1.5 equiv) at 0 °C. After stirring 2 h at this temperature, the reaction was quenched with saturated aqueous NH_4Cl (20 mL). The mixture was extracted with ethyl acetate (20 mL × 3). The combined organic layer was washed with brine (15 mL), dried over Na_2SO_4, filtered, and concentrated in vacuo. The crude product was purified by flash chromatography to afford **S8**.

(2) To a solution of **S8** (8 mmol, 1.0 equiv) in dichloromethane (0.4 M) was added bromoacetic acid (9.6 mmol, 1.2 equiv), EDCI HCl (9.6 mmol, 1.2 mmol), and 4-(dimethylamino)pyridine (0.8 mmol, 0.1 equiv) at 0 °C. Then the solution was allowed to warm to room temperature and stirred for 6 h. After completion, the reaction was quenched with saturated aqueous $NaHCO_3$ (20 mL) and the mixture was extracted with dichloromethane (15 mL × 3). The combined organic extract was washed with brine (15 mL), dried over Na_2SO_4 and concentrated in vacuo. The crude product was purified by flash column chromatography (petroleum ether / ethyl acetate) to afford **S9**.

(3) To a solution of **S9** (5 mmol, 1.0 equiv) in N,N-dimethylformamide (0.3 M) was added sodium benzenesulfinate (6 mmol, 1.2 equiv), tetrabutylammonium iodide (0.5 mmol, 0.1 equiv) and potassium iodide (6 mmol, 1.2 equiv). The reaction mixture was stirred at room temperature overnight and then quenched with saturated aqueous $NaHCO_3$ (10 mL). The mixture was extracted with ethyl acetate (10 mL × 3). The combined organic layer was washed with brine (10 mL), dried over Na_2SO_4, filtered, and concentrated in vacuo. The crude product was purified by flash chromatography to afford **S10**.

3.5 Compound 1bk was prepared according to the general procedure 5 (GP5)

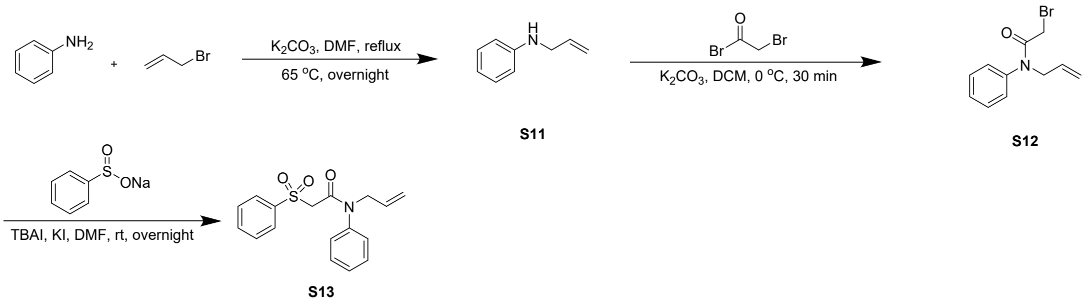

(1) To a solution of aniline (5 mmol, 1.0 equiv) in N,N-dimethylformamide (15 mL) was added potassium carbonate (6 mmol, 1.2 equiv) and allyl bromide (5 mmol, 1.0 equiv). The reaction mixture was stirred at 65 °C overnight and then cooled to room temperature. The reaction was quenched with H_2O (10 mL). The mixture was extracted with ethyl acetate (15 mL × 3). The combined organic layer was washed with brine (15 mL), dried over Na_2SO_4, filtered, and concentrated in vacuo. The crude product was purified by flash chromatography to afford **S11**.

(2) Following the reported procedure,[9] to a solution of **S11** (3 mmol, 1.0 eq) in dichloromethane (0.3 M) was added slowly K_2CO_3 (4.5 mmol, 1.5 eq), bromoacetyl bromide (3.6 mmol, 1.2 eq) at 0 °C and stirred for 30 min. The reaction was quenched with H_2O (10 mL). The mixture was extracted with dichloromethane (10 mL × 3). The combined organic layer was washed with brine (10 mL), dried over Na_2SO_4, filtered, and concentrated in vacuo. The crude product was purified by flash chromatography to afford **S12**.

(3) To a solution of **S12** (5 mmol, 1.0 equiv) in N,N-dimethylformamide (0.3 M) was added sodium benzenesulfinate (6 mmol, 1.2 equiv), tetrabutylammonium iodide (0.5 mmol, 0.1 equiv) and potassium iodide (6 mmol, 1.2 equiv). The reaction mixture was stirred at room temperature overnight and then quenched with saturated aqueous $NaHCO_3$ (10 mL). The mixture was extracted with ethyl acetate (10 mL × 3). The combined organic layer was washed with brine (10 mL), dried over Na_2SO_4, filtered, and concentrated in vacuo. The crude product was purified by flash chromatography to afford **S13**.

4. The general procedure 6 (GP6) for the synthesis of cyclopropanation product 2

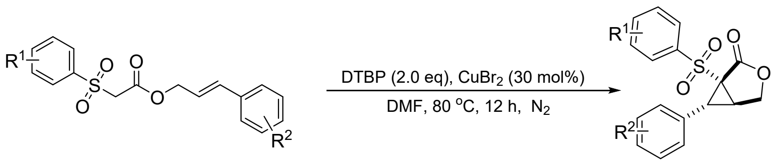

1 2

An oven-dried 5 mL Schlenk tube equipped with a PTFE-coated stir bar was charged with **1** (63.3 mg, 0.2 mmol, 1.0 equiv) and $CuBr_2$ (13.4 mg, 0.06 mmol, 30 mol%), then the tube was evacuated and backfilled with N_2 for three times. DMF (1.0 mL) and DTBP (58.5 mg, 0.40 mmol, 2.0 equiv) were successively added via syringe under N_2 atmosphere. The sealed tube was placed into a preheated oil bath at 80 °C with stirring for 12 h. After cooling to room temperature, the mixture was diluted with water (10 mL). The layer was separated and the aqueous layer was extracted with ethyl acetate (10 mL × 3). The combined organic layer was rinsed with brine (10 mL), dried over Na_2SO_4, and concentrated in vacuo. The resultant residue was purified by flash column chromatography (petroleum ether / ethyl acetate) on silica gel to afford **2.**

5. Scale-up synthesis of product 2aa

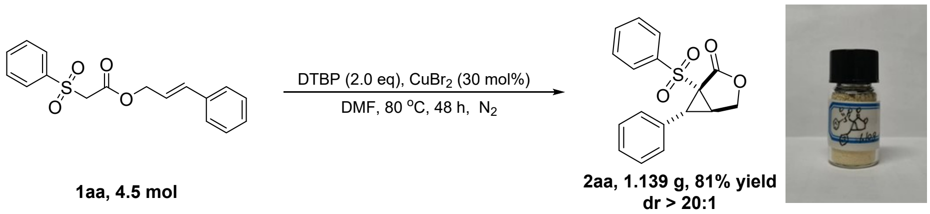

1aa, 4.5 mol

2aa, 1.139 g, 81% yield
dr > 20:1

An oven-dried 50 mL Schlenk tube equipped with a PTFE-coated stir bar was charged with **1aa** (1.42 g, 4.5 mmol, 1.0 equiv) and $CuBr_2$ (301.5 mg, 1.35 mmol, 30 mol%), then the tube was evacuated and backfilled with N_2 for three times. DMF (25 mL) and DTBP (1.32 g, 9.0 mmol, 2.0 equiv) were successively added via syringe under N_2 atmosphere. The sealed tube was placed into a preheated oil bath at 80 °C with stirring for 48 h. After cooling to room temperature, the mixture was diluted with water (50 mL). The layer was separated and the aqueous layer was extracted with ethyl acetate (30 mL × 3). The combined organic layer was rinsed with brine (30 mL), dried over Na_2SO_4, and concentrated in vacuo. The resultant residue was purified by flash column chromatography (petroleum ether / ethyl acetate) on silica gel to afford **2aa** (1.14 g, 3.62 mmol, 81%).

6. Derivatization reactions of product 2aa

6.1 Synthesis of compound 3

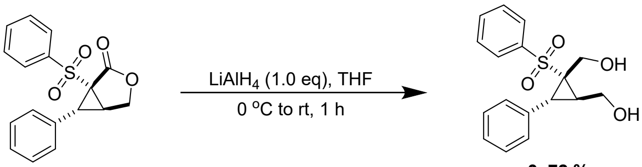

2aa

3, 72 %
single diastereomer

An oven-dried 5 mL Schlenk tube equipped with a PTFE-coated stir bar was charged with **2aa** (62.9 mg, 0.2 mmol, 1.0 equiv), then the tube was evacuated and backfilled with N_2 for three times. Dry THF (2 ml) and $LiAlH_4$ (200 µl 1.0 M solution in tetrahydrofuran, 0.2 mmol, 1.0 eq) were successively added via syringe under N_2 atmosphere at 0 °C. After stirring 1 h at this temperature, carefully quenched with 5 mL of a saturated solution of NH_4Cl. The reaction mixture was stirred at room temperature for 1 h, and the resulting white precipitate was filtered through a pad of Celite®. The filtrate was extracted three times with ethyl acetate (5 ml × 3) and concentrated in vacuo. The crude product was purified by flash chromatography to afford **3**.[10]

6.2 Synthesis of compound 4

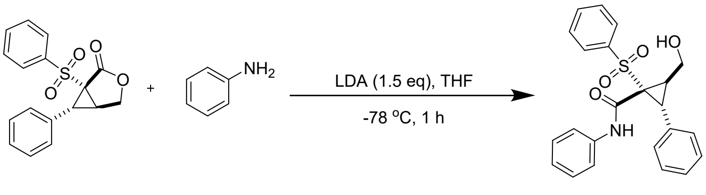

2aa

4, 89 %
single diastereomer

An oven-dried 5 mL Schlenk tube equipped with a PTFE-coated stir bar was charged with **2aa** (62.9 mg, 0.2 mmol,

1.0 equiv), then the tube was evacuated and backfilled with N_2 for three times. Dry THF (2 ml) and aniline (22.4 mg, 0.24 mmol, 1.2 equiv) were successively added via syringe under N_2 atmosphere. Then the LDA (150 µl 2.0 M solution in tetrahydrofuran, 0.3 mmol, 1.5 eq) was added dropwise to the solution of **2aa** under - 78 °C. After stirring 1 h at this temperature, the solution was allowed to warm to room temperature and quenched with saturated aqueous NH_4Cl (10 mL). The mixture was extracted with ethyl acetate (5 mL × 3). The combined organic layer was washed with brine (10 mL), dried over Na_2SO_4, filtered, and concentrated in vacuo. The crude product was purified by flash chromatography to afford **4.**[11]

6.3 Synthesis of compound 5

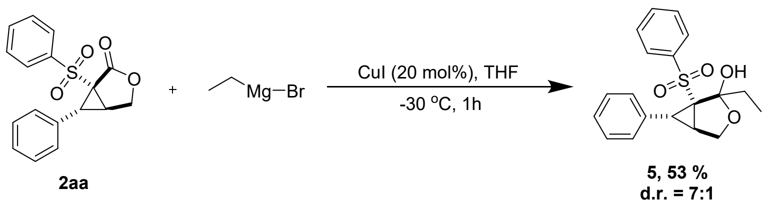

An oven-dried 5 mL Schlenk tube equipped with a PTFE-coated stir bar was charged with **2aa** (62.9 mg, 0.2 mmol, 1.0 equiv) and cuprous iodide (7.6 mg, 0.04 mmol, 0.2 eq), then the tube was evacuated and backfilled with N_2 for three times. Dry THF (2 ml) and ethylmagnesium bromide (240 µl 1.0 M solution in tetrahydrofuran, 0.24 mmol, 1.2 eq) were successively added via syringe under N_2 atmosphere at - 30 °C. After stirring 1 h at this temperature, the reaction was quenched with saturated aqueous NH_4Cl (5 mL). The mixture was extracted with ethyl acetate (5 mL × 3). The combined organic layer was washed with brine (15 mL), dried over Na_2SO_4, filtered, and concentrated in vacuo. The crude product was purified by flash chromatography to afford **5.**

6.3 Synthesis of compound 6

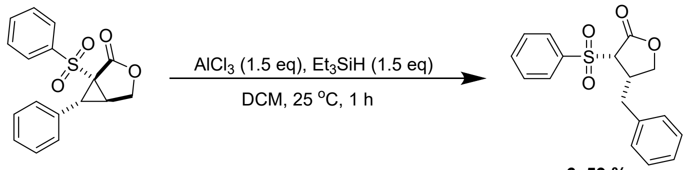

2aa **6, 59 %** **d.r. > 20:1**

An oven-dried 5 mL Schlenk tube equipped with a PTFE-coated stir bar was charged with **2aa** (62.9 mg, 0.2 mmol, 1.0 equiv) and $AlCl_3$ (40.0 mg, 0.3 mmol, 1.5 eq), then the tube was evacuated and backfilled with N_2 for three times. DCM (2.0 mL) and Et_3SiH (47.9 μl, 0.3 mmol, 1.5 equiv) were successively added via syringe under N_2 atmosphere.The reaction mixture was stirred at room temperature for 1 h and then quenched with saturated aqueous $NaHCO_3$ (10 mL). The mixture was extracted with DCM. The combined organic layer was washed with brine (10 mL), dried over Na_2SO_4, filtered, and concentrated in vacuo. The crude product was purified by flash chromatography to afford **6.**[12]

7. Control experiments

7.1 Comparing of this method with reported ones

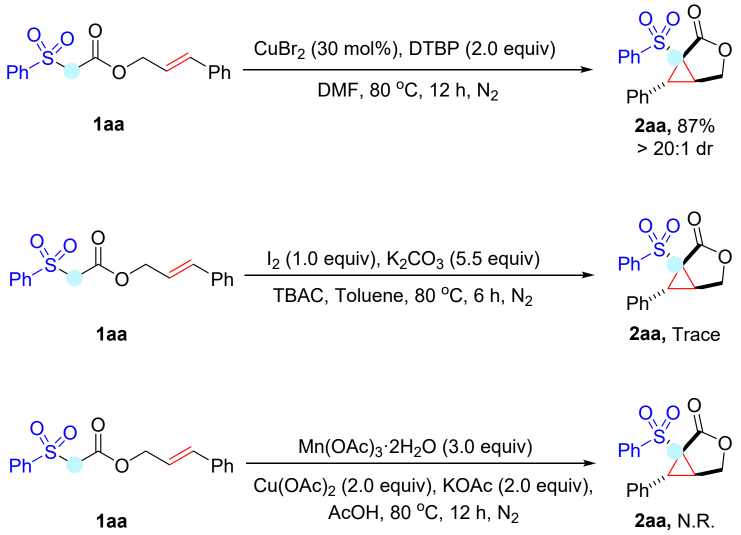

Followed the reported procedure for the intramolecular cyclopropanation reactions, both the halocyclization and $Mn(OAc)_3$ mediated cyclization protocols were evaluated.[13] Neither the $Mn(OAc)_3$ mediated oxidative annulation nor the halocyclization protocol could efficiently produce the expected cyclopropane-fused γ-lactone.

7.2 Radical trapping experiments

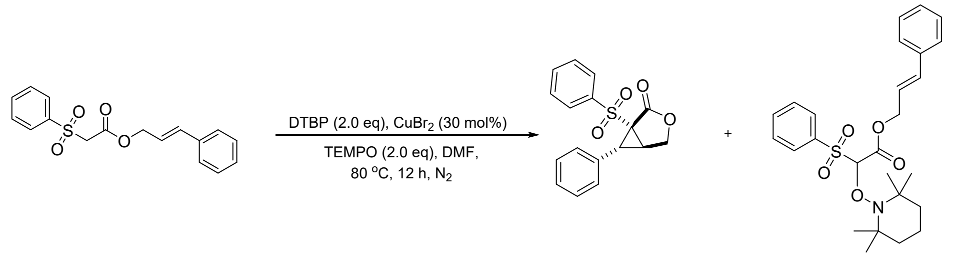

1aa **2aa, trace** **7, 22% yield**

An oven-dried 5 mL Schlenk tube equipped with a PTFE-coated stir bar was charged with **1aa** (63.3 mg, 0.2 mmol, 1.0 equiv), $CuBr_2$ (13.4 mg, 0.06 mmol, 30 mol%) and 2,2,6,6-tetramethylpiperidine N-oxide (TEMPO, 62.5 mg, 0.4 mmol, 2.0 equiv), then the tube was evacuated and backfilled with N_2 for thrree times. DMF (1.0 mL) and DTBP (58.5 mg, 0.4 mmol, 2.0 equiv) were successively added via syringe under N_2 atmosphere. The mixture was stirred at 80 °C for 12 h under N_2 atmosphere. After cooling to room temperature, the mixture was diluted with water (10 mL). The layer was separated and the aqueous layer was extracted with ethyl acetate (10 mL × 3). The combined organic layer was rinsed with brine (10 mL), dried over Na_2SO_4, and concentrated in vacuo. Traces of the product 2aa were observed by TLC analysis. The resultant residue was purified by flash column chromatography (petroleum ether / ethyl acetate) on silica gel to afford **7** (21.1 mg, 0.045 mmol, 22%).

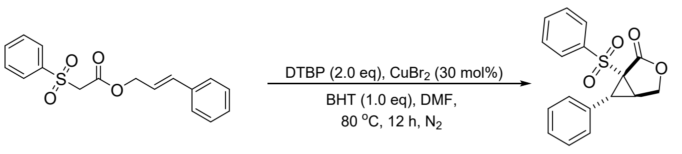

1aa **2aa, trace**

An oven-dried 5 mL Schlenk tube equipped with a PTFE-coated stir bar was charged with **1aa** (63.3 mg, 0.2 mmol, 1.0 equiv), $CuBr_2$ (13.4 mg, 0.06 mmol, 30 mol%) and 2,6-di-*tert*-butyl-4-methylphenol (BHT, 44.1 mg, 0.2 mmol, 1.0 equiv), then the tube was evacuated and backfilled with N_2 for three times. DMF (1.0 mL) and DTBP (58.5 mg,0.4 mmol, 2.0 equiv) were successively added via syringe under N_2 atmosphere. The mixture was stirred at 80 °C for 12 h under N_2 atmosphere. After cooling to room temperature, the mixture was diluted with water (10 mL). The layer was separated and the aqueous layer was extracted with ethyl acetate (10 mL × 3). The combined organic layer was rinsed with brine (10 mL), dried over Na_2SO_4, and concentrated in vacuo. Traces of the product **2aa** were observed by TLC analysis.

7.3 Carbocation trapping

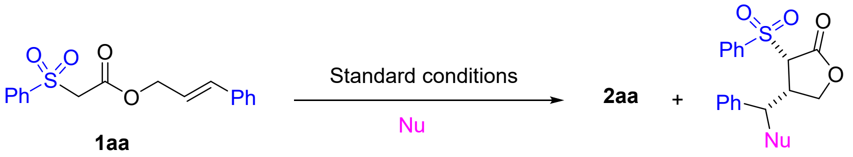

Entry	Nucleophile	Yield of **2aa** （%）	Yield of **8** （%）
1	H_2O (30 equiv)	Trace	-
2	MeOH (3.0 equiv)	Trace	-
3	TMSCN (1.5 equiv)	-	-
4	Aniline (1.5 equiv)	-	-
5	Indole (3.0 equiv)	-	-
6	Et_3N • 3HF (3.0 equiv)	-	-
7	TFE (3.0 equiv)	58	-
8	CH_3ONa (3.0 equiv)	-	-
9	NaOH (3.0 equiv)	Trace	-
10	CH_3COONa (3.0 equiv)	Trace	-
11	KBr (3.0 equiv)	69	-
12	1,3,5-Trimethoxybenzene (3.0 equiv)	60	-

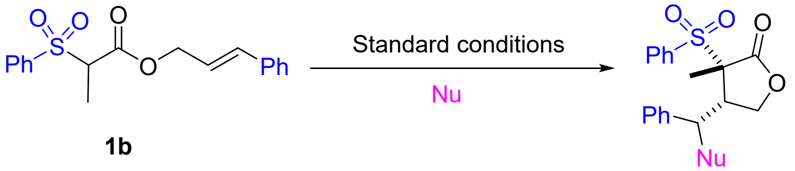

Entry	Nucleophile	Yield of **10** （%）
1	H_2O (30 equiv)	-.
2	MeOH (3.0 equiv)	-
3	TMSCN (1.5 equiv)	-
4	Indole (3.0 equiv)	-
5	1,3,5-Trimethoxybenzene (3.0 equiv)	-

8. Characterization of products

Cinnamyl 2-(phenylsulfonyl)acetate (1aa)

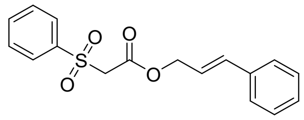

The title compound was prepared according to GP1 and isolated as a white solid. 81% yield, 1.281 g. mp 89 - 91 °C. ^{1}H NMR (400 MHz, $CDCl_3$) δ 7.96 – 7.93 (m, 2H), 7.65 – 7.61 (m, 1H), 7.54 – 7.50 (m, 2H), 7.38 – 7.28 (m, 5H), 6.61 (d, *J* = 15.9 Hz, 1H), 6.13 (dt, *J* = 16.0, 6.6 Hz, 1H), 4.73 (dd, *J* = 6.6, 1.3 Hz, 2H), 4.16 (s, 2H). ^{13}C NMR (100 MHz, $CDCl_3$) δ 162.3, 138.6, 135.9, 135.6, 134.4, 129.3, 128.8, 128.7, 128.5, 126.8, 121.6, 66.9, 61.1. HRMS (ESI, m/z): calcd. for $C_{17}H_{16}O_4S$ + Na^+ [M + Na]$^+$: 339.0662; found: 339.0669.

(*Z*)-3-Phenylallyl 2-(phenylsulfonyl)acetate (1aa')

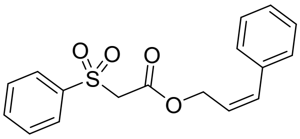

The title compound was prepared according to literature reports[14] and isolated as a colorless oil. 60% yield, 65 mg. ^{1}H NMR (400 MHz, $CDCl_3$) δ 7.96 – 7.94 (m, 2H), 7.69 – 7.65 (m, 1H), 7.58 – 7.53 (m, 2H), 7.37 – 7.33 (m, 2H), 7.31 – 7.27 (m, 1H), 7.18 – 7.16 (m, 2H), 6.68 (d, *J* = 11.6 Hz, 1H), 5.68 (dt, *J* = 11.6, 6.8 Hz, 1H), 4.84 (dd, *J* = 6.8, 1.6 Hz, 2H), 4.15 (s, 2H). ^{13}C NMR (100 MHz, $CDCl_3$) δ 162.3, 138.7, 135.7, 134.4, 134.2, 129.4, 128.8, 128.7, 128.6, 127.9, 124.2, 63.3, 61.1. HRMS (ESI, m/z): calcd. for $C_{17}H_{16}O_4S$ + Na^+ [M + Na]$^+$: 339.0662; found: 339.0665.

Cinnamyl 2-((4-methoxyphenyl)sulfonyl)acetate (1ac)

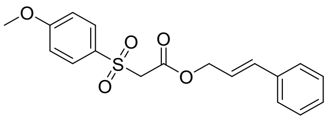

The title compound was prepared according to GP2 and isolated as a white solid. 66% yield, 1.142 g, mp 86 - 90 °C. ^{1}H NMR (400 MHz, $CDCl_3$) δ 7.87 – 7.82 (m, 2H), 7.38 – 7.27 (m, 5H), 6.97 – 6.93 (m, 2H), 6.61 (d, *J* = 15.9 Hz, 1H), 6.15 (dt, *J* = 15.9, 6.6 Hz, 1H), 4.74 (dd, *J* = 6.6, 1.3 Hz, 2H), 4.13 (s, 2H), 3.79 (s, 3H). ^{13}C NMR (100 MHz, $CDCl_3$) δ 164.3, 162.6, 135.9, 135.5, 131.0, 130.0, 128.8, 128.5, 126.8, 121.7, 114.5, 66.8, 61.4, 55.7. HRMS (ESI, m/z): calcd. for $C_{18}H_{18}O_5S$ + Na^+ [M + Na]$^+$: 369.0767; found: 369.0766.

Cinnamyl 2-((4-(*tert*-butyl)phenyl)sulfonyl)acetate (1ad)

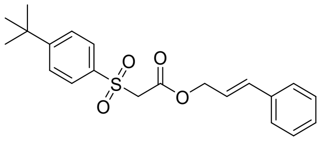

The title compound was prepared according to GP2 and isolated as a colorless oil. 45% yield, 838.1 mg. ^{1}H NMR (400 MHz, $CDCl_3$) δ 7.87 – 7.83 (m, 2H), 7.53 – 7.51 (m, 2H), 7.39 – 7.27 (m, 5H), 6.64 (d, *J* = 15.9 Hz, 1H), 6.18 (dt, *J* = 15.9, 6.6 Hz, 1H), 4.76 (dd, *J* = 6.6, 1.3 Hz, 2H), 4.15 (s, 2H), 1.30 (s, 9H). ^{13}C NMR (100 MHz, $CDCl_3$) δ 162.4, 158.5, 135.9, 135.7, 135.6, 128.8, 128.6, 128.5, 126.8, 126.3, 121.7, 66.9, 61.1, 35.4, 31.1. HRMS (ESI, m/z): calcd. for $C_{21}H_{24}O_4S$ + Na^+ $[M + Na]^+$: 395.1288; found: 395.1289.

Cinnamyl 2-((4-fluorophenyl)sulfonyl)acetate (1ae)

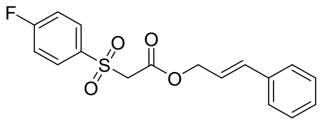

The title compound was prepared according to GP2 and isolated as a white solid. 40% yield, 668.8 mg, mp 59 - 63 °C. ^{1}H NMR (400 MHz, $CDCl_3$) δ 7.97 – 7.93 (m, 2H), 7.39 – 7.27 (m, 5H), 7.22 – 7.14 (m, 2H), 6.63 (d, *J* = 15.9 Hz, 1H), 6.15 (dt, *J* = 15.9, 6.6 Hz, 1H), 4.75 (dd, *J* = 6.6, 0.8 Hz, 2H), 4.16 (s, 2H). ^{19}F NMR (376 MHz, $CDCl_3$) δ -102.6 – -102.8 (m, 1F). ^{13}C NMR (100 MHz, $CDCl_3$) δ 166.3 (d, $J_{C–F}$ = 256.0 Hz), 162.3, 135.9, 135.8, 134.6 (d, $J_{C–F}$ = 3.5 Hz), 131.8 (d, $J_{C–F}$ = 9.7 Hz), 128.8, 128.6, 126.8, 121.5, 116.7 (d, $J_{C–F}$ = 22.9 Hz), 67.0, 61.1. HRMS (ESI, m/z): calcd. for $C_{17}H_{15}FO_4S$ + Na^+ $[M + Na]^+$: 357.0567; found: 357.0566.

Cinnamyl 2-((4-chlorophenyl)sulfonyl)acetate (1af)

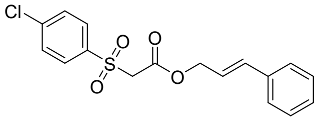

The title compound was prepared according to GP2 and isolated as a white solid. 50% yield, 877.1 mg, mp 76 - 80 °C. ^{1}H NMR (400 MHz, $CDCl_3$) δ 7.89 – 7.84 (m, 2H), 7.50 – 7.45 (m, 2H), 7.40 – 7.27 (m, 5H), 6.63 (d, *J* = 15.9 Hz, 1H), 6.14 (dt, *J* = 15.8, 6.7 Hz, 1H), 4.74 (dd, *J* = 6.7, 1.3 Hz, 2H), 4.16 (s, 2H). ^{13}C NMR (100 MHz, $CDCl_3$) δ 162.2, 141.3, 137.0, 135.9, 135.8, 130.3, 129.7, 128.9, 128.6, 126.8, 121.5, 67.0, 61.0. HRMS (ESI, m/z): calcd. for $C_{17}H_{15}{}^{35}ClO_4S$ + Na^+ $[M + Na]^+$: 37.0272; found: 373.0289.

Cinnamyl 2-((4-bromophenyl)sulfonyl)acetate (1ag)

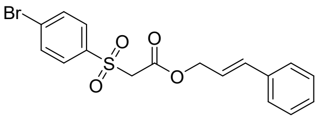

The title compound was prepared according to GP2 and isolated as a white solid. 55% yield, 1.089 g, mp 89 - 95 °C. ^{1}H NMR (400 MHz, $CDCl_3$) δ 7.81 – 7.77 (m, 2H), 7.66 – 7.63 (m, 2H), 7.39 – 7.28 (m, 5H), 6.63 (d, *J* = 15.9 Hz, 1H), 6.14 (dt, *J* = 15.9, 6.6 Hz, 1H), 4.75 (dd, *J* = 6.6, 1.3 Hz, 2H), 4.15 (s, 2H). ^{13}C NMR (100 MHz, $CDCl_3$) δ 162.1, 137.5, 135.9, 135.8, 132.7, 130.3, 130.0, 128.9, 128.6, 126.8, 121.4, 67.0, 61.0. HRMS (ESI, m/z): calcd. for $C_{17}H_{15}{}^{79}BrO_4S + Na^+$ $[M + Na]^+$: 416.9767; found: 416.9768.

Cinnamyl 2-(*m*-tolylsulfonyl)acetate (1ah)

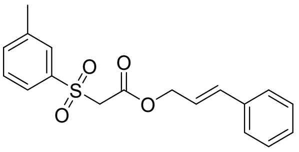

The title compound was prepared according to GP2 and isolated as a colorless oil. 56% yield, 925.1 mg. ^{1}H NMR (400 MHz, $CDCl_3$) δ 7.77 – 7.72 (m, 2H), 7.39 – 7.27 (m, 7H), 6.60 (d, *J* = 15.9 Hz, 1H), 6.13 (dt, *J* = 15.9, 6.6 Hz, 1H), 4.73 (dd, *J* = 6.6, 1.3 Hz, 2H), 4.16 (s, 2H), 2.37 (s, 3H). ^{13}C NMR (100 MHz, $CDCl_3$) δ 162.3, 139.7, 138.5, 135.9, 135.4, 135.2, 129.2, 128.9, 128.8, 128.5, 126.8, 125.8, 121.7, 66.8, 61.1, 21.4. HRMS (ESI, m/z): calcd. for $C_{18}H_{18}O_4S + Na^+$ $[M + Na]^+$: 353.0818; found: 353.0821.

Cinnamyl 2-((3-bromophenyl)sulfonyl)acetate (1ai)

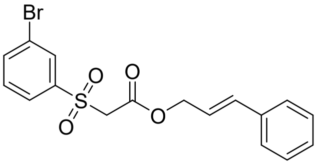

The title compound was prepared according to GP2 and isolated as a colorless oil. 45% yield, 889.4 mg. ^{1}H NMR (400 MHz, $CDCl_3$) δ 8.09 (t, *J* = 1.9 Hz, 1H), 7.87 (d, *J* = 7.9 Hz, 1H), 7.75 (d, *J* = 8.0 Hz, 1H), 7.39 – 7.27 (m, 6H), 6.63 (d, *J* = 15.8 Hz, 1H), 6.15 (dt, *J* = 15.8, 6.6 Hz, 1H), 4.75 (d, *J* = 6.6 Hz, 2H), 4.17 (s, 2H). ^{13}C NMR (100 MHz, $CDCl_3$) δ 162.0, 140.4, 137.5, 135.9, 135.8, 131.6, 130.8, 128.8, 128.6, 127.4, 126.9, 123.3, 121.3, 67.1, 61.0. HRMS (ESI, m/z): calcd. for $C_{17}H_{15}{}^{79}BrO_4S + Na^+$ $[M + Na]^+$: 416.9767; found: 416.9771.

Cinnamyl 2-(*o*-tolylsulfonyl)acetate (1aj)

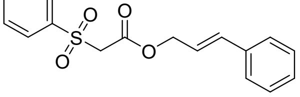

The title compound was prepared according to GP2 and isolated as a colorless oil. 64% yield, 1.056 g.

^{1}H NMR (400 MHz, $CDCl_3$) δ 7.99 (dd, *J* = 8.0, 1.4 Hz, 1H), 7.48 (td, *J* = 7.4, 1.4 Hz, 1H), 7.36 – 7.27 (m, 7H), 6.57 (d, *J* = 15.9 Hz, 1H), 6.07 (dt, *J* = 15.9, 6.6 Hz, 1H), 4.69 (dd, *J* = 6.6, 1.3 Hz, 2H), 4.19 (s, 2H), 2.71 (s, 3H). ^{13}C NMR (100 MHz, $CDCl_3$) δ 162.2, 138.3, 136.8, 135.9, 135.5, 134.4, 132.9, 130.9, 128.8, 128.5, 126.8, 126.7, 121.6, 66.9, 60.4, 20.5. HRMS (ESI, m/z): calcd. for $C_{18}H_{18}O_4S + Na^+$ $[M + Na]^+$: 353.0818; found: 353.0826.

Cinnamyl 2-((2-chlorophenyl)sulfonyl)acetate (1ak)

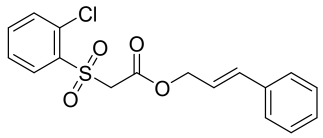

The title compound was prepared according to GP2 and isolated as a colorless oil. 57% yield, 999.8 mg. ^{1}H NMR (400 MHz, $CDCl_3$) δ 8.10 (dd, *J* = 8.0, 1.5 Hz, 1H), 7.56 – 7.51 (m, 2H), 7.37 – 7.27 (m, 6H), 6.56 (d, *J* = 15.9 Hz, 1H), 6.05 (dt, *J* = 15.8, 6.6 Hz, 1H), 4.69 (dd, *J* = 6.6, 1.3 Hz, 2H), 4.49 (s, 2H). ^{13}C NMR (100 MHz, $CDCl_3$) δ 162.0, 136.2, 135.9, 135.6, 135.3, 132.8, 132.3, 131.9, 128.8, 128.5, 127.5, 126.8, 121.5, 66.9, 58.8. HRMS (ESI, m/z): calcd. for $C_{17}H_{15}{}^{35}ClO_4S + Na^+$ $[M + Na]^+$: 373.0272; found: 373.0276.

Cinnamyl 2-((2-bromophenyl)sulfonyl)acetate (1al)

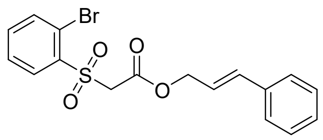

The title compound was prepared according to GP2 and isolated as a colorless oil. 53% yield, 1.047 g. ^{1}H NMR (400 MHz, $CDCl_3$) δ 8.15 – 8.12 (m, 1H), 7.76 – 7.74 (m, 1H), 7.43 – 7.38 (m, 2H), 7.36 – 7.32 (m, 4H), 7.31 – 7.27 (m, 1H), 6.56 (dt, *J* = 15.8, 1.3 Hz, 1H), 6.06 (dt, *J* = 15.9, 6.6 Hz, 1H), 4.69 (dd, *J* = 6.6, 1.3 Hz, 2H), 4.53 (s, 2H). ^{13}C NMR (100 MHz, $CDCl_3$) δ 162.0, 137.8, 135.9, 135.6, 135.5, 135.3, 132.7, 128.8, 128.5, 128.1, 126.8, 121.5, 120.9, 66.9, 58.4. HRMS (ESI, m/z): calcd. for $C_{17}H_{15}{}^{79}BrO_4S + Na^+$ $[M + Na]^+$: 416.9767; found: 416.9778.

Cinnamyl 2-(naphthalen-2-ylsulfonyl)acetate (1am)

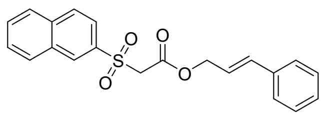

The title compound was prepared according to GP2 and isolated as a colorless oil. 76% yield, 1.392 g. ^{1}H NMR (400 MHz, $CDCl_3$) δ 8.52 (d, *J* = 1.8 Hz, 1H), 7.96 (d, *J* = 8.7 Hz, 1H), 7.92 – 7.88 (m, 3H),

7.68 – 7.63 (m, 1H), 7.60 – 7.56 (m, 1H), 7.34 – 7.27 (m, 5H), 6.54 (d, *J* = 15.9 Hz, 1H), 6.05 (dt, *J* = 15.9, 6.6 Hz, 1H), 4.71 (dd, *J* = 6.7, 1.3 Hz, 2H), 4.24 (s, 2H). ^{13}C NMR (100 MHz, $CDCl_3$) δ 162.3, 148.8, 135.8, 135.6, 135.6, 135.5, 132.1, 130.9, 129.7, 129.6, 128.7, 128.5, 128.1, 127.9, 126.8, 123.0, 121.5, 66.9, 61.2. HRMS (ESI, m/z): calcd. for $C_{21}H_{18}O_4S + Na^+$ $[M + Na]^+$: 389.0818; found: 389.0821.

Cinnamyl 2-(pyridin-4-ylsulfonyl)acetatev (1an)

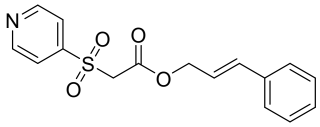

The title compound was prepared according to GP2 and isolated as a white solid. 35% yield, 555.4 mg. mp 57 - 61 °C. ^{1}H NMR (400 MHz, $CDCl_3$) δ 8.88 – 8.86 (m, 2H), 7.81 – 7.78 (m, 2H), 7.39 – 7.28 (m, 5H), 6.64 (d, *J* = 15.9 Hz, 1H), 6.15 (dt, *J* = 15.9, 6.8 Hz, 1H), 4.76 (dd, *J* = 6.8, 1.3 Hz, 2H), 4.20 (s, 2H). ^{13}C NMR (100 MHz, $CDCl_3$) δ 161.7, 151.1, 146.8, 136.2, 135.7, 128.9, 128.7, 126.8, 121.9, 121.2, 67.3, 60.4. HRMS (ESI, m/z): calcd. for $C_{16}H_{15}NO_4S + H^+$ $[M + H]^+$: 318.0795; found: 318.0795.

Cinnamyl 2-(thiophen-2-ylsulfonyl)acetate (1ao)

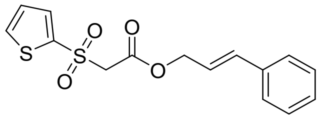

The title compound was prepared according to GP2 and isolated as a white solid. 41% yield, 660.9 mg. mp 45 - 48 °C. ^{1}H NMR (400 MHz, $CDCl_3$) δ 7.76 – 7.75 (m, 1H), 7.72 – 7.70 (m, 1H), 7.39 – 7.26 (m, 5H), 7.12 – 7.09 (m, 1H), 6.65 (d, *J* = 15.9 Hz, 1H), 6.19 (dt, *J* = 15.9, 6.6 Hz, 1H), 4.79 (dd, *J* = 6.7, 1.3 Hz, 2H), 4.25 (s, 2H). ^{13}C NMR (100 MHz, $CDCl_3$) δ 162.2, 139.3, 135.9, 135.7, 135.6, 135.2, 128.8, 128.5, 128.1, 126.8, 121.7, 67.0, 62.1. HRMS (ESI, m/z): calcd. for $C_{15}H_{14}O_4S_2+Na^+$ $[M + Na]^+$: 345.0226; found: 345.0226.

Cinnamyl 2-(benzo[d]thiazol-2-ylsulfonyl)acetate (1ap)

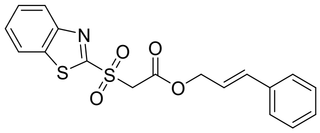

The title compound was prepared according to GP2 and isolated as a white solid. 30% yield, 560.2 mg. mp 71 - 76 °C. ^{1}H NMR (400 MHz, $CDCl_3$) δ 8.20 – 8.18 (m, 1H), 7.91 – 7.88 (m, 1H), 7.62 – 7.58 (m, 1H), 7.54 – 7.53 (m, 1H), 7.33 – 7.27 (m, 3H), 7.26 – 7.23 (m, 2H), 6.52 (d, *J* = 15.9 Hz, 1H), 6.04 (dt, *J* = 15.9, 6.6 Hz, 1H), 4.74 (dd, *J* = 6.6, 1.3 Hz, 2H), 4.62 (s, 2H). ^{13}C NMR (100 MHz, $CDCl_3$) δ 164.9,

161.5, 152.5, 137.0, 135.8, 135.7, 128.7, 128.5, 128.3, 127.9, 126.8, 125.6, 122.5, 121.3, 67.2, 58.9. HRMS (ESI, m/z): calcd. for $C_{18}H_{15}NO_4S_2 + Na^+$ $[M + Na]^+$: 396.0335; found: 396.0338.

Cinnamyl 2-(benzylsulfonyl)acetate (1aq)

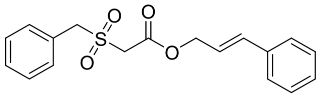

The title compound was prepared according to GP2 and isolated as a colorless oil. 50% yield, 826.0 mg. ^{1}H NMR (400 MHz, $CDCl_3$) δ 7.51 – 7.49 (m, 2H), 7.43 – 7.38 (m, 5H), 7.37 – 7.28 (m, 3H), 6.74 (d, *J* = 15.9 Hz, 1H), 6.30 (dt, *J* = 15.9, 6.6 Hz, 1H), 4.90 (dd, *J* = 6.6, 1.3 Hz, 2H), 4.53 (s, 2H), 3.82 (s, 2H). ^{13}C NMR (100 MHz, $CDCl_3$) δ 163.3, 135.9, 131.1, 129.5, 129.3, 128.8, 128.7, 128.6, 127.8, 126.9, 121.6, 67.2, 59.6, 54.7. HRMS (ESI, m/z): calcd. for $C_{18}H_{18}O_4S + Na^+$ $[M + Na]^+$: 353.0818; found: 353.0819.

(*E*)-3-(*p*-tolyl)allyl 2-(phenylsulfonyl)acetate (1ba)

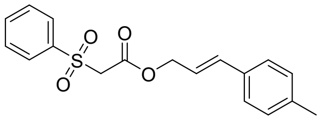

The title compound was prepared according to GP3 and isolated as a colorless oil. 75% yield, 1.239 g. ^{1}H NMR (400 MHz, $CDCl_3$) δ 7.95 – 7.93 (m, 2H), 7.65 – 7.61 (m, 1H), 7.54 – 7.49 (m, 2H), 7.27 – 7.25 (m, 2H), 7.14 (d, *J* = 8 Hz, 2H), 6.57 (d, *J* = 15.8 Hz, 1H), 6.08 (dt, *J* = 15.8, 6.7 Hz, 1H), 4.71 (dd, *J* = 6.7, 1.3 Hz, 2H), 4.16 (s, 2H), 2.35 (s, 3H). ^{13}C NMR (100 MHz, $CDCl_3$) δ 162.3, 138.6, 138.5, 135.7, 134.4, 133.1, 129.5, 129.3, 128.7, 126.7, 120.5, 67.1, 61.1, 21.4. HRMS (ESI, m/z): calcd. for $C_{18}H_{18}O_4S$ + Na^+ $[M + Na]^+$: 353.0818; found: 353.0821.

(*E*)-3-(*m*-tolyl)allyl 2-(phenylsulfonyl)acetate (1bb)

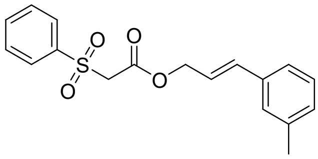

The title compound was prepared according to GP3 and isolated as a colorless oil. 70% yield, 1.156 g. ^{1}H NMR (400 MHz, $CDCl_3$) δ 7.96 – 7.93 (m, 2H), 7.66 – 7.65 (m, 1H), 7.54 – 7.50 (m, 2H), 7.25 – 7.21 (m, 1H), 7.18 – 7.15 (m, 2H), 7.10 (d, *J* = 7.4 Hz, 1H), 6.58 (d, *J* = 15.9 Hz, 1H), 6.12 (dt, *J* = 15.8, 6.6 Hz, 1H), 4.72 (dd, *J* = 6.6, 1.3 Hz, 2H), 4.16 (s, 2H), 2.36 (s, 3H). ^{13}C NMR (100 MHz, $CDCl_3$) δ 162.3, 138.6, 138.4, 135.8, 135.8, 134.4, 129.3, 129.3, 128.7, 128.7, 127.5, 124.0, 121.4, 67.0, 61.1, 21.5. HRMS (ESI, m/z): calcd. for $C_{18}H_{18}O_4S + Na^+$ $[M + Na]^+$: 353.0818; found: 353.0822.

(*E*)-3-(*o*-Tolyl)allyl 2-(phenylsulfonyl)acetate (1bc)

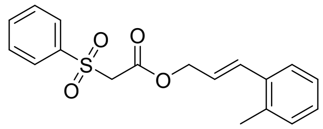

The title compound was prepared according to GP3 and isolated as a colorless oil. 66% yield, 1.090 g. ^{1}H NMR (400 MHz, $CDCl_3$) δ 7.96 – 7.93 (m, 2H), 7.64 – 7.60 (m, 1H), 7.53 – 7.49 (m, 2H), 7.41 – 7.38 (m, 1H), 7.20 – 7.14 (m, 3H), 6.86 (d, *J* = 15.7 Hz, 1H), 6.01 (dt, *J* = 15.7, 6.6 Hz, 1H), 4.75 (dd, *J* = 6.6, 1.3 Hz, 2H), 4.17 (s, 2H), 2.34 (s, 3H). ^{13}C NMR (100 MHz, $CDCl_3$) δ 162.3, 138.7, 135.9, 135.1, 134.5, 133.5, 130.5, 129.4, 128.7, 128.4, 126.3, 125.9, 123.0, 67.1, 61.1, 19.9. HRMS (ESI, m/z): calcd. for $C_{18}H_{18}O_4S + Na^+$ $[M + Na]^+$: 353.0818; found: 353.0819.

(*E*)-3-(4-chlorophenyl)allyl 2-(phenylsulfonyl)acetate (1be)

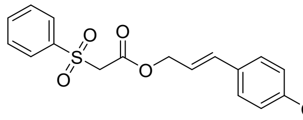

The title compound was prepared according to GP3 and isolated as a colorless oil. 69% yield, 1.210 g. ^{1}H NMR (400 MHz, $CDCl_3$) δ 7.95 – 7.92 (m, 2H), 7.65 – 7.61 (m, 1H), 7.54 – 7.50 (m, 2H), 7.31 – 7.26 (m, 4H), 6.56 (d, *J* = 16.0 Hz, 1H), 6.10 (dt, *J* = 15.9, 6.4 Hz, 1H), 4.72 (dd, *J* = 6.5, 1.4 Hz, 2H), 4.16 (s, 2H). ^{13}C NMR (100 MHz, $CDCl_3$) δ 162.3, 138.6, 134.5, 134.4, 134.1, 134.1, 129.4, 129.0, 128.7, 128.0, 122.4, 66.6, 61.0. HRMS (ESI, m/z): calcd. for $C_{17}H_{15}{}^{35}ClO_4S + Na^+$ $[M + Na]^+$: 373.0272; found: 373.0277.

(*E*)-3-(3,4-dichlorophenyl)allyl 2-(phenylsulfonyl)acetate (1bf)

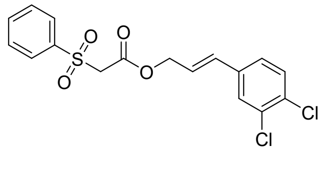

The title compound was prepared according to GP3 and isolated as a white solid. 80% yield, 1.541 g. mp 46 - 51 °C. ^{1}H NMR (400 MHz, $CDCl_3$) δ 7.94 – 7.92 (m, 2H), 7.68 – 7.64 (m, 1H), 7.56 – 7.52 (m, 2H), 7.41 (d, *J* = 2.1 Hz, 1H), 7.38 (d, *J* = 8.4 Hz, 1H), 7.17 (dd, *J* = 8.3, 2.1 Hz, 1H), 6.51 (d, *J* = 15.8 Hz, 1H), 6.11 (dt, *J* = 15.9, 6.3 Hz, 1H), 4.72 (d, *J* = 6.3 Hz, 2H), 4.17 (s, 2H). ^{13}C NMR (100 MHz, $CDCl_3$) δ 162.2, 138.7, 136.1, 134.5, 132.9, 132.5, 132.1, 130.7, 129.4, 128.6, 128.4, 126.0, 123.8, 66.2, 61.0. HRMS (ESI, m/z): calcd. for $C_{17}H_{14}{}^{35}Cl_2O_4S + Na^+$ $[M + Na]^+$: 406.9882; found: 406.9884.

(*E*)-3-(4-(trifluoromethyl)phenyl)allyl 2-(phenylsulfonyl)acetate (1bg)

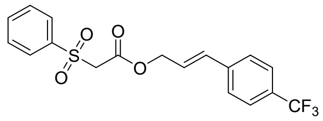

The title compound was prepared according to GP3 and isolated as a white solid. 67% yield, 1.288 g, mp 73 - 76 °C. ^{1}H NMR (400 MHz, $CDCl_3$) δ 7.96 – 7.93 (m, 2H), 7.67 – 7.63 (m, 1H), 7.58 (d, J = 8.2 Hz, 2H), 7.56 – 7.51 (m, 2H), 7.46 (d, J = 8.2 Hz, 2H), 6.65 (d, J = 16.0 Hz, 1H), 6.24 (dt, J = 16.0, 6.2 Hz, 1H), 4.77 (dd, J = 6.2, 1.4 Hz, 2H), 4.18 (s, 2H). ^{19}F NMR (376 MHz, $CDCl_3$) δ -63.3 (s, 3F). ^{13}C NMR (100 MHz, $CDCl_3$) δ 162.2, 139.4, 138.7, 134.5, 133.5, 130.1 (q, $J_{C\text{–}F}$ = 32.4 Hz), 129.4, 128.7, 127.0, 125.7 (q, $J_{C\text{–}F}$ = 3.9 Hz), 124.4, 124.2 (q, $J_{C\text{–}F}$ = 270.6 Hz), 66.3, 61.0. HRMS (ESI, m/z): calcd. for $C_{18}H_{15}F_3O_4S + Na^+$ $[M + Na]^+$: 407.0535; found: 407.0540.

(*E*)-3-(4-nitrophenyl)allyl 2-(phenylsulfonyl)acetate (1bh)

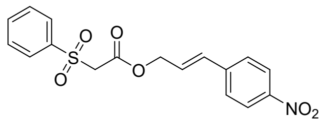

The title compound was prepared according to GP3 and isolated as a yellow solid. 77% yield, 1.391 g, mp 93 - 98 °C. ^{1}H NMR (400 MHz, $CDCl_3$) δ 8.22 – 8.18 (m, 2H), 7.97 – 7.94 (m, 2H), 7.69 – 7.65 (m, 1H), 7.58 – 7.50 (m, 4H), 6.72 (d, J = 16.0 Hz, 1H), 6.34 (dt, J = 16.0, 6.0 Hz, 1H), 4.81 (dd, J = 6.0, 1.6 Hz, 2H), 4.19 (s, 2H). ^{13}C NMR (100 MHz, $CDCl_3$) δ 162.2, 147.5, 142.3, 138.7, 134.5, 132.3, 129.4, 128.6, 127.4, 126.6, 124.2, 66.0, 61.0. HRMS (ESI, m/z): calcd. for $C_{17}H_{15}NO_6S + Na^+$ $[M + Na]^+$: 384.0512; found: 384.0535.

(*E*)-4-phenylbut-3-en-2-yl 2-(phenylsulfonyl)acetate (1bk)

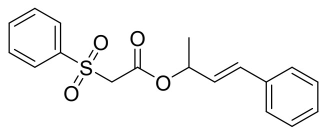

The title compound was prepared according to GP4 and isolated as a colorless oil. 69% yield, 1.140 g. ^{1}H NMR (400 MHz, $CDCl_3$) δ 7.94 – 7.91 (m, 2H), 7.64 – 7.59 (m, 1H), 7.51 – 7.49 (m, 2H), 7.37 – 7.27 (m, 5H), 6.57 (d, J = 15.9 Hz, 1H), 6.03 (dd, J = 16.0, 7.2 Hz, 1H), 5.50 – 5.46 (m, 1H), 4.13 (s, 2H), 1.36 (d, J = 6.4 Hz, 3H). ^{13}C NMR (100 MHz, $CDCl_3$) δ 161.7, 138.6, 136.0, 134.4, 132.9, 129.3, 128.8, 128.7, 128.4, 127.3, 126.8, 73.8, 61.3, 20.2. HRMS (ESI, m/z): calcd. for $C_{18}H_{18}O_4S + Na^+$ $[M + Na]^+$: 353.0818; found: 353.0823.

(*E*)-2-methyl-3-phenylallyl 2-(phenylsulfonyl)acetate (1bl)

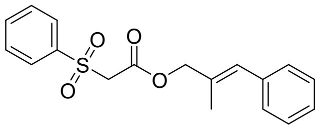

The title compound was prepared according to GP3 and isolated as a colorless oil. 75% yield, 1.239 g. ^{1}H NMR (400 MHz, $CDCl_3$) δ 7.97 – 7.94 (m, 2H), 7.68 – 7.63 (m, 1H), 7.57 – 7.53 (m, 2H), 7.37 – 7.33 (m, 2H), 7.27 – 7.23 (m, 3H), 6.49 (s, 1H), 4.65 (s, 2H), 4.19 (s, 2H), 1.82 (d, J = 1.2 Hz, 3H). ^{13}C NMR (100 MHz, $CDCl_3$) δ 162.3, 138.7, 136.7, 134.4, 131.5, 129.8, 129.4, 129.0, 128.7, 128.3, 127.2, 72.3, 61.1, 15.6. HRMS (ESI, m/z): calcd. for $C_{18}H_{18}O_4S + Na^+$ $[M + Na]^+$: 353.0818; found: 353.0825.

Cinnamyl 2-(ethylsulfonyl)acetate (1bn)

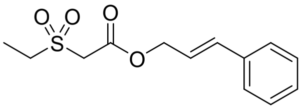

The title compound was prepared according to GP2 and isolated as a yellow oil. 61% yield, 817 mg. ^{1}H NMR (400 MHz, $CDCl_3$) δ 7.41 – 7.38 (m, 2H), 7.35 – 7.28 (m, 3H), 6.71 (d, J = 15.9 Hz, 1H), 6.27 (dt, J = 15.9, 6.6 Hz, 1H), 4.86 (dd, J = 6.6, 1.3 Hz, 2H), 4.00 (s, 2H), 3.29 (q, J = 7.5 Hz, 2H), 3.29 (t, J = 7.4 Hz, 3H). ^{13}C NMR (100 MHz, $CDCl_3$) δ 163.0, 135.9, 135.8, 128.8, 128.5, 126.9, 121.6, 67.2, 56.6, 48.2, 6.7. HRMS (ESI, m/z): calcd. for $C_{13}H_{16}O_4S + Na^+$ $[M + Na]^+$: 291.0662; found: 291.0667.

N-allyl-N-phenyl-2-(phenylsulfonyl)acetamide (1bt)

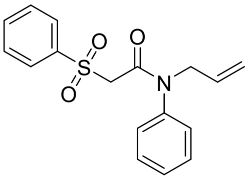

The title compound was prepared according to GP5 and isolated as a yellow oil. 60% yield, 946.2 mg. ^{1}H NMR (400 MHz, $CDCl_3$) δ 7.92 – 7.89 (m, 2H), 7.68 – 7.64 (m, 1H), 7.58 – 7.54 (m, 2H), 7.42 – 7.36 (m, 3H), 7.16 – 7.14 (m, 2H), 5.82 – 5.75 (m, 1H), 5.14 – 5.08 (m, 2H), 4.26 (d, J = 6.2 Hz, 2H), 3.98 (s, 2H). ^{13}C NMR (100 MHz, $CDCl_3$) δ 161.2, 141.2, 139.5, 134.1, 132.0, 130.1, 129.1, 129.0, 128.9, 128.5, 118.7, 59.4, 52.7. HRMS (ESI, m/z): calcd. for $C_{17}H_{17}NO_3S + Na^+$ $[M + Na]^+$: 338.0821; found: 338.0820.

Cinnamyl 2-(phenylsulfonyl)propanoate (1b)

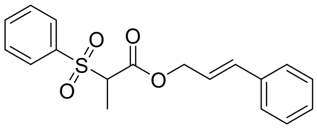

The title compound was isolated as a yellow oil. 80% yield, 1.322 g. ^{1}H NMR (400 MHz, $CDCl_3$) δ 7.90 – 7.86 (m, 2H), 7.64 – 7.59 (m, 1H), 7.51 – 7.47 (m, 2H), 7.38 – 7.27 (m, 5H), 6.60 (d, *J* = 15.9 Hz, 1H), 6.11 (dt, *J* = 15.9, 6.6 Hz, 1H), 4.71 (d, *J* = 6.6 Hz, 2H), 4.10 (q, *J* = 7.2 Hz, 1H), 1.61 (d, *J* = 7.1 Hz, 3H). ^{13}C NMR (100 MHz, $CDCl_3$) δ 166.2, 136.9, 135.9, 135.5, 134.4, 129.5, 129.2, 128.8, 128.5, 126.8, 121.8, 66.8, 65.5, 11.8. HRMS (ESI, m/z): calcd. for $C_{18}H_{18}O_4S$ + Na^+ $[M + Na]^+$: 353.0818; found: 353.0824.

6-Phenyl-1-(phenylsulfonyl)-3-oxabicyclo[3.1.0]hexan-2-one (2aa)

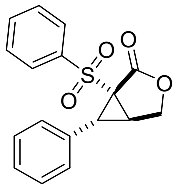

Purified by flash chromatography with EA/PE (1/4) as eluent, 87% yield, 54.7 mg; white solid. mp 174 - 178 °C. d.r. > 20 : 1; ^{1}H NMR (400 MHz, $CDCl_3$) δ 7.56 – 7.52 (m, 1H), 7.46 – 7.43 (m, 2H), 7.36 – 7.29 (m, 3H), 7.26 – 7.23 (m, 2H), 7.04 – 7.02 (m, 2H), 4.61 (dd, *J* = 9.5, 4.8 Hz, 1H), 4.38 (d, *J* = 9.6 Hz, 1H), 3.84 (dd, *J* = 6.1, 4.5 Hz, 1H), 2.87 (d, *J* = 6.2 Hz, 1H). ^{13}C NMR (100 MHz, $CDCl_3$) δ 168.6, 138.6, 134.0, 130.0, 128.6, 128.6, 128.6, 128.3, 127.9, 67.6, 52.7, 37.3, 28.0. HRMS (ESI, m/z): calcd. for $C_{17}H_{14}O_4S$ + Na^+ $[M + Na]^+$: 337.0505; found: 337.0508.

6-Phenyl-1-(phenylsulfonyl)-3-oxabicyclo[3.1.0]hexan-2-one [2aa (1aa')]

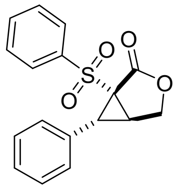

Purified by flash chromatography with EA/PE (1/3.5) as eluent, 78% yield, 49.4 mg; white solid. mp 176 - 179 °C. d.r. > 20 : 1; ^{1}H NMR (400 MHz, $CDCl_3$) δ 7.57 – 7.51 (m, 1H), 7.45 – 7.43 (m, 2H), 7.36 – 7.30 (m, 3H), 7.27 – 7.23 (m, 2H), 7.04 – 7.02 (m, 2H), 4.63 (dd, *J* = 9.5, 4.8 Hz, 1H), 4.39 (d, *J* = 9.5 Hz, 1H), 3.84 (dd, *J* = 6.2, 4.6 Hz, 1H), 2.87 (d, *J* = 6.2 Hz, 1H). ^{13}C NMR (100 MHz, $CDCl_3$) δ 168.6, 138.5, 134.0, 130.0, 128.6, 128.6, 128.6, 128.3, 127.9, 67.6, 52.7, 37.3, 28.0. HRMS (ESI, m/z): calcd. for $C_{17}H_{14}O_4S$ + Na^+ $[M + Na]^+$: 337.0505; found: 337.0507.

6-Phenyl-1-tosyl-3-oxabicyclo[3.1.0]hexan-2-one (2ab)

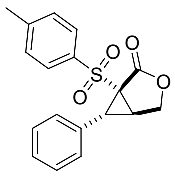

Purified by flash chromatography with EA/PE (1/4) as eluent, 89% yield, 58.5 mg; white solid. mp 184 - 188 °C. d.r. > 20 : 1; ^{1}H NMR (400 MHz, $CDCl_3$) δ 7.37 – 7.27 (m, 5H), 7.13 – 7.11 (m, 2H), 7.09 – 7.06 (m, 2H), 4.62 (dd, J = 9.5, 4.7 Hz, 1H), 4.38 (d, J = 9.5 Hz, 1H), 3.81 (dd, J = 6.1, 4.8 Hz, 1H), 2.86 (d, J = 6.2 Hz, 1H), 2.39 (s, 3H). ^{13}C NMR (100 MHz, $CDCl_3$) δ 168.7, 145.1, 135.6, 130.0, 129.2, 128.8, 128.6, 128.2, 128.1, 67.6, 52.7, 37.2, 27.9, 21.8. HRMS (ESI, m/z): calcd. for $C_{18}H_{16}O_4S$ + Na^+ $[M + Na]^+$: 351.0662; found: 351.0663.

1-((4-Methoxyphenyl)sulfonyl)-6-phenyl-3-oxabicyclo[3.1.0]hexan-2-one (2ac)

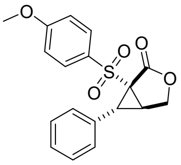

Purified by flash chromatography with EA/PE (1/5) as eluent, 82% yield, 56.5 mg; white solid. mp 155 - 160 °C. d.r. > 20 : 1; ^{1}H NMR (400 MHz, $CDCl_3$) δ 7.36 – 7.33 (m, 3H), 7.30 – 7.26 (m, 2H), 7.09 – 7.07 (m, 2H), 6.79 – 6.76 (m, 2H), 4.59 (dd, J = 9.5, 4.6 Hz, 1H), 4.35 (d, J = 9.4 Hz, 1H), 3.83 (s, 3H), 3.79 (dd, J = 6.2, 4.7 Hz, 1H), 2.85 (d, J = 6.1 Hz, 1H). ^{13}C NMR (100 MHz, $CDCl_3$) δ 168.9, 164.0, 131.1, 130.1, 128.6, 128.6, 128.2, 128.2, 113.7, 67.6, 55.8, 52.8, 37.1, 27.9. HRMS (ESI, m/z): calcd. for $C_{18}H_{16}O_5S$ + Na^+ $[M + Na]^+$: 367.0611; found: 367.0610.

1-((4-(*tert*-Butyl)phenyl)sulfonyl)-6-phenyl-3-oxabicyclo[3.1.0]hexan-2-one (2ad)

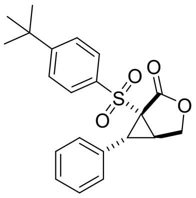

Purified by flash chromatography with EA/PE (1/6) as eluent, 71% yield, 52.9 mg; white solid. mp 224 - 228 °C. d.r. > 20 : 1; ^{1}H NMR (400 MHz, $CDCl_3$) δ 7.38 – 7.30 (m, 5H), 7.26 – 7.22 (m, 2H), 7.06 – 7.03 (m, 2H), 4.62 (dd, J = 9.5, 4.7 Hz, 1H), 4.37 (d, J = 9.6 Hz, 1H), 3.81 (dd, J = 6.1, 4.6 Hz, 1H), 2.86 (d, J = 6.2 Hz, 1H), 1.31 (s, 9H). ^{13}C NMR (100 MHz, $CDCl_3$) δ 168.7, 157.9, 135.6, 130.0, 128.6, 128.5, 128.2, 128.1, 125.6, 67.6, 52.8, 37.2, 35.3, 31.1, 28.0. HRMS (ESI, m/z): calcd. for $C_{21}H_{22}O_4S$ + Na^+ $[M + Na]^+$: 393.1131; found: 393.1132.

1-((4-Fluorophenyl)sulfonyl)-6-phenyl-3-oxabicyclo[3.1.0]hexan-2-one (2ae)

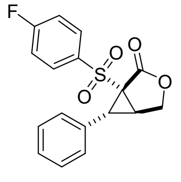

Purified by flash chromatography with EA/PE (1/5) as eluent, 85% yield, 56.6 mg; white solid. mp 210 - 215 °C. d.r. > 20 : 1; ^{1}H NMR (400 MHz, $CDCl_3$) δ 7.47 – 7.40 (m, 2H), 7.38 – 7.34 (m, 1H), 7.30 – 7.26 (m, 2H), 7.06 – 7.03 (m, 2H), 7.02 – 6.95 (m, 2H), 4.65 (dd, *J* = 9.6, 4.7 Hz, 1H), 4.40 (d, *J* = 9.6 Hz, 1H), 3.84 (dd, *J* = 6.2, 4.7 Hz, 1H), 2.87 (d, *J* = 6.2 Hz, 1H). ^{19}F NMR (376 MHz, $CDCl_3$) δ -103.6 – -103.7 (m, 1F). ^{13}C NMR (100 MHz, $CDCl_3$) δ 168.5, 166.0 (d, $J_{C–F}$ = 255.1 Hz), 134.5 (d, $J_{C–F}$ = 3.0 Hz), 131.7 (d, $J_{C–F}$ = 9.6 Hz), 130.0, 128.8, 128.3, 127.8, 115.8 (d, $J_{C–F}$ = 22.7 Hz), 67.6, 52.7, 37.3, 27.9. HRMS (ESI, m/z): calcd. for $C_{17}H_{13}FO_4S + Na^+$ $[M + Na]^+$: 355.0411; found: 355.0418.

1-((4-Chlorophenyl)sulfonyl)-6-phenyl-3-oxabicyclo[3.1.0]hexan-2-one (2af)

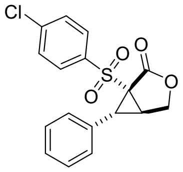

Purified by flash chromatography with EA/PE (1/4) as eluent, 81% yield, 56.4 mg; white solid. mp 182 - 188 °C. d.r. > 20 : 1; ^{1}H NMR (400 MHz, $CDCl_3$) δ 7.38 – 7.32 (m, 3H), 7.30 – 7.27 (m, 4H), 7.06 – 7.03 (m, 2H), 4.64 (dd, *J* = 9.6, 4.7 Hz, 1H), 4.40 (d, *J* = 9.6 Hz, 1H), 3.85 (dd, *J* = 6.3, 4.6 Hz, 1H), 2.88 (d, *J* = 6.2 Hz, 1H). ^{13}C NMR (100 MHz, $CDCl_3$) δ 168.5, 140.8, 136.9, 130.2, 130.0, 128.9, 128.8, 128.3, 127.7, 67.7, 52.7, 37.3, 28.0. HRMS (ESI, m/z): calcd. for $C_{17}H_{13}{}^{35}ClO_4S + Na^+$ $[M + Na]^+$: 371.0115; found: 371.0122.

1-((4-Bromophenyl)sulfonyl)-6-phenyl-3-oxabicyclo[3.1.0]hexan-2-one (2ag)

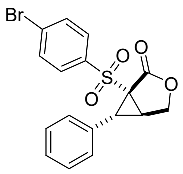

Purified by flash chromatography with EA/PE (1/4) as eluent, 78% yield, 61.4 mg; white solid. mp 166 - 170 °C. d.r. > 20 : 1; ^{1}H NMR (400 MHz, $CDCl_3$) δ 7.47 – 7.43 (m, 2H), 7.38 – 7.34 (m, 1H), 7.30 – 7.27 (m, 3H), 7.25 – 7.24 (m, 1H), 7.06 – 7.03 (m, 2H), 4.64 (dd, *J* = 9.6, 4.7 Hz, 1H), 4.40 (d, *J* = 9.6 Hz, 1H), 3.85 (dd, *J* = 6.3, 4.6 Hz, 1H), 2.88 (d, *J* = 6.3 Hz, 1H). ^{13}C NMR (100 MHz, $CDCl_3$) δ 168.4,

137.4, 131.9, 130.2, 130.0, 129.5, 128.8, 128.3, 127.7, 67.7, 52.7, 37.3, 28.0. HRMS (ESI, m/z): calcd. for $C_{17}H_{13}{}^{79}BrO_4S + Na^+$ $[M + Na]^+$: 414.9610; found: 414.9618.

6-Phenyl-1-(*m*-tolylsulfonyl)-3-oxabicyclo[3.1.0]hexan-2-one (2ah)

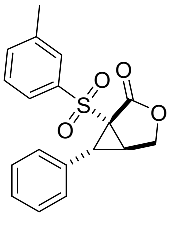

Purified by flash chromatography with EA/PE (1/4) as eluent, 87% yield, 57.1 mg; white solid. mp 180 - 183 °C. d.r. > 20 : 1; ^1H NMR (400 MHz, $CDCl_3$) δ 7.46 – 7.43 (m, 1H), 7.36 – 7.32 (m, 2H), 7.28 – 7.22 (m, 3H), 7.05 – 7.03 (m, 2H), 6.98 – 6.97 (m, 1H), 4.61 (dd, J = 9.6, 4.8 Hz, 1H), 4.37 (d, J = 9.6 Hz, 1H), 3.83 (dd, J = 6.2, 4.6 Hz, 1H), 2.85 (d, J = 6.2 Hz, 1H), 2.23 (s, 3H). ^{13}C NMR (100 MHz, $CDCl_3$) δ 168.6, 138.6, 138.3, 134.7, 130.1, 128.8, 128.6, 128.6, 128.1, 128.0, 126.0, 67.6, 52.8, 37.2, 27.9, 21.3. HRMS (ESI, m/z): calcd. for $C_{18}H_{16}O_4S + Na^+$ $[M + Na]^+$: 351.0662; found: 351.0662.

1-((3-Bromophenyl)sulfonyl)-6-phenyl-3-oxabicyclo[3.1.0]hexan-2-one (2ai)

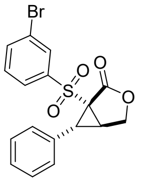

Purified by flash chromatography with EA/PE (1/4) as eluent, 64% yield, 50.1 mg; white solid. mp 168 - 172 °C. d.r. > 20 : 1; ^1H NMR (400 MHz, $CDCl_3$) δ 7.75 – 7.72 (m, 1H), 7.69 – 7.66 (m, 1H), 7.41 – 7.37 (m, 1H), 7.33 – 7.28 (m, 3H), 7.14 – 7.13 (m, 1H), 7.04 – 7.01 (m, 2H), 4.66 (dd, J = 9.6, 4.8 Hz, 1H), 4.41 (d, J = 9.6 Hz, 1H), 3.87 (dd, J = 6.3, 4.7 Hz, 1H), 2.90 (d, J = 6.3 Hz, 1H). ^{13}C NMR (100 MHz, $CDCl_3$) δ 168.3, 140.2, 137.0, 131.2, 130.3, 129.9, 129.1, 128.4, 127.6, 127.3, 122.4, 67.6, 52.6, 37.4, 28.0. HRMS (ESI, m/z): calcd. for $C_{17}H_{13}{}^{79}BrO_4S + Na^+$ $[M + Na]^+$: 414.9610; found: 414.9619.

6-Phenyl-1-(*o*-tolylsulfonyl)-3-oxabicyclo[3.1.0]hexan-2-one (2aj)

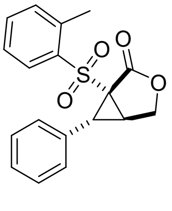

Purified by flash chromatography with EA/PE (1/4) as eluent, 79% yield, 51.9 mg; white solid. mp 133 - 136 °C. d.r. > 20 : 1; ^1H NMR (400 MHz, $CDCl_3$) δ 7.74 – 7.72 (m, 1H), 7.43 – 7.39 (m, 1H), 7.32 –

7.26 (m, 2H), 7.25 – 7.14 (m, 5H), 4.58 (dd, *J* = 9.5, 4.7 Hz, 1H), 4.39 (d, *J* = 9.6 Hz, 1H), 3.81 (dd, *J* = 6.2, 4.6 Hz, 1H), 3.01 (d, *J* = 6.1 Hz, 1H), 2.42 (s, 3H). ^{13}C NMR (100 MHz, $CDCl_3$) δ 168.4, 139.2, 137.4, 133.8, 132.6, 130.6, 129.9, 128.7, 128.5, 128.4, 126.5, 67.4, 53.7, 37.5, 29.2, 20.6. HRMS (ESI, m/z): calcd. for $C_{18}H_{16}O_4S + Na^+$ $[M + Na]^+$: 351.0662; found: 351.0662.

1-((2-Chlorophenyl)sulfonyl)-6-phenyl-3-oxabicyclo[3.1.0]hexan-2-one (2ak)

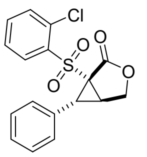

Purified by flash chromatography with EA/PE (1/4) as eluent, 62% yield, 43.3 mg; white solid. mp 192 - 198 °C. d.r. > 20 : 1; ^{1}H NMR (400 MHz, $CDCl_3$) δ 8.16 – 8.14 (m, 1H), 7.56 – 7.33 (m, 8H), 4.60 (dd, *J* = 9.6, 4.7 Hz, 1H), 4.40 (d, *J* = 9.5 Hz, 1H), 3.89 (dd, *J* = 6.2, 4.7 Hz, 1H), 3.18 (d, *J* = 6.1 Hz, 1H). ^{13}C NMR (100 MHz, $CDCl_3$) δ 167.9, 136.9, 135.2, 133.2, 131.7, 131.6, 129.9, 129.1, 128.9, 128.6, 127.6, 67.7, 52.2, 37.9, 31.6. HRMS (ESI, m/z): calcd. for $C_{17}H_{13}{}^{35}ClO_4S + Na^+$ $[M + Na]^+$: 371.0115; found: 371.0119.

1-((2-Bromophenyl)sulfonyl)-6-phenyl-3-oxabicyclo[3.1.0]hexan-2-one (2al)

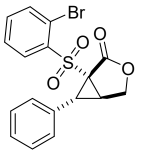

Purified by flash chromatography with EA/PE (1/3) as eluent, 58% yield, 45.7 mg; white solid. mp 155 - 161 °C. d.r. > 20 : 1; ^{1}H NMR (400 MHz, $CDCl_3$) δ 8.21 (dd, *J* = 7.8, 1.9 Hz, 1H), 7.70 (dd, *J* = 7.7, 1.4 Hz, 1H), 7.52 – 7.33 (m, 7H), 4.66 (dd, *J* = 9.5, 4.6 Hz, 1H), 4.41 (d, *J* = 9.6 Hz, 1H), 3.95 (dd, *J* = 6.2, 4.6 Hz, 1H), 3.17 (d, *J* = 6.2 Hz, 1H). ^{13}C NMR (100 MHz, $CDCl_3$) δ 167.9, 138.5, 135.3, 135.2, 133.8, 130.0, 129.0, 128.9, 128.6, 128.1, 119.7, 67.7, 52.1, 37.8, 32.0. HRMS (ESI, m/z): calcd. for $C_{17}H_{13}{}^{79}BrO_4S + Na^+$ $[M + Na]^+$: 414.9610; found: 414.9615.

1-(Naphthalen-2-ylsulfonyl)-6-phenyl-3-oxabicyclo[3.1.0]hexan-2-one (2am)

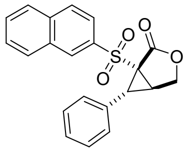

Purified by flash chromatography with EA/PE (1/3) as eluent, 88% yield, 64.0 mg; white solid. mp 194 - 204 °C. d.r. > 20 : 1; ^{1}H NMR (400 MHz, $CDCl_3$) δ 7.88 – 7.81 (m, 3H), 7.67 – 7.62 (m, 2H), 7.58 – 7.50 (m, 2H), 7.37 – 7.33 (m, 1H), 7.20 – 7.16 (m, 2H), 6.99 – 6.97 (m, 2H), 4.66 (dd, *J* = 9.5, 4.7 Hz,

1H), 4.40 (d, J = 9.5 Hz, 1H), 3.89 (dd, J = 6.2, 4.6 Hz, 1H), 2.86 (d, J = 6.2 Hz, 1H). ^{13}C NMR (100 MHz, $CDCl_3$) δ 168.6, 135.5, 135.3, 131.6, 130.7, 130.0, 129.8, 129.4, 128.9, 128.7, 128.2, 127.9, 127.8, 127.3, 123.4, 67.6, 52.5, 37.3, 27.9. HRMS (ESI, m/z): calcd. for $C_{21}H_{16}O_4S + Na^+$ $[M + Na]^+$: 387.0662; found: 387.0667.

6-Phenyl-1-(pyridin-4-ylsulfonyl)-3-oxabicyclo[3.1.0]hexan-2-one(2an)

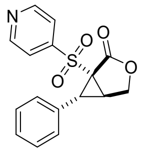

Purified by flash chromatography with EA/PE (1/2) as eluent, 57% yield, 36.0 mg; white solid. mp 203 - 206 °C. d.r. > 20 : 1; ^{1}H NMR (400 MHz, $(CD_3)_2SO$) δ 8.76 (s, 2H), 7.35 – 7.23 (m, 5H), 7.16 – 7.14 (m, 2H), 4.69 (dd, J = 9.5, 4.9 Hz, 1H), 4.49 (d, J = 9.5 Hz, 1H), 4.28 (dd, J = 6.4, 4.6 Hz, 1H), 3.47 (d, J = 6.5 Hz, 1H). ^{13}C NMR (100 MHz, $(CD_3)_2SO$) δ 168.7, 151.3, 146.9, 130.6, 129.1, 128.8, 128.3, 121.0, 68.6, 52.5, 37.3, 29.3. HRMS (ESI, m/z): calcd. for $C_{16}H_{13}NO_4S + Na^+$ $[M + Na]^+$: 338.0458; found: 338.0460.

6-Phenyl-1-(thiophen-2-ylsulfonyl)-3-oxabicyclo[3.1.0]hexan-2-one (2ao)

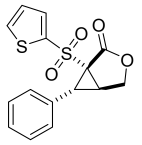

Purified by flash chromatography with EA/PE (1/3) as eluent, 62% yield, 39.5 mg; white solid. mp 196 - 203 °C. d.r. > 20 : 1; ^{1}H NMR (400 MHz, $CDCl_3$) δ 7.63 (dd, J = 5.0, 1.4 Hz, 1H), 7.36 – 7.29 (m, 3H), 7.21 (dd, J = 3.9, 1.4 Hz, 1H), 7.17 – 7.15 (m, 2H), 6.98 (dd, J = 4.9, 3.8 Hz, 1H), 4.61 (dd, J = 9.5, 4.7 Hz, 1H), 4.39 (d, J = 9.6 Hz, 1H), 3.81 (dd, J = 6.0, 4.5 Hz, 1H), 2.95 (d, J = 6.2 Hz, 1H). ^{13}C NMR (100 MHz, $CDCl_3$) δ 168.4, 138.8, 135.6, 134.9, 130.0, 128.8, 128.3, 128.0, 127.3, 67.5, 52.9, 37.6, 28.3. HRMS (ESI, m/z): calcd. for $C_{15}H_{12}O_4S_2 + Na^+$ $[M + Na]^+$: 343.0069; found: 343.0077.

1-(Benzo[*d*]thiazol-2-ylsulfonyl)-6-phenyl-3-oxabicyclo[3.1.0]hexan-2-one (2ap)

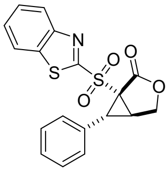

Purified by flash chromatography with EA/PE (1/1) as eluent, 14% yield, 10.2 mg; white solid. mp 182 - 190 °C. d.r. > 20 : 1; ^{1}H NMR (400 MHz, $CDCl_3$) δ 8.17 – 8.15 (m, 1H), 7.96 – 7.94 (m, 1H), 7.63 –

7.57 (m, 2H), 7.36 – 7.27 (m, 5H), 4.72 (dd, J = 9.5, 4.7 Hz, 1H), 4.48 (d, J = 9.5 Hz, 1H), 3.95 (dd, J = 6.3, 4.6 Hz, 1H), 3.26 (d, J = 6.3 Hz, 1H). ^{13}C NMR (100 MHz, $CDCl_3$) δ 167.4, 165.1, 152.1, 137.2, 129.8, 129.0, 128.5, 128.5, 128.2, 127.6, 125.7, 122.3, 67.6, 51.8, 38.1, 29.8. HRMS (ESI, m/z): calcd. for $C_{18}H_{13}NO_4S_2$ + Na^+ [M + Na]$^+$: 394.0178; found: 394.0183.

1-(Benzylsulfonyl)-6-phenyl-3-oxabicyclo[3.1.0]hexan-2-one (2aq)

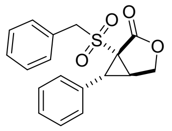

Purified by flash chromatography with EA/PE (1/3) as eluent, 64% yield, 42.1 mg; white solid. mp 177 - 183 °C. d.r. > 20 : 1; ^{1}H NMR (400 MHz, $CDCl_3$) δ 7.38 – 7.29 (m, 6H), 7.24 – 7.19 (m, 4H), 4.65 (d, J = 13.6 Hz, 1H), 4.32 (d, J = 9.5 Hz, 1H), 4.24 (dd, J = 9.5, 4.4 Hz, 1H), 4.18 (d, J = 13.5 Hz, 1H), 3.39 (t, J = 5.2 Hz, 1H), 2.99 (d, J = 5.9 Hz, 1H). ^{13}C NMR (100 MHz, $CDCl_3$) δ 169.3, 131.3, 129.6, 129.2, 129.0, 128.8, 128.7, 128.5, 126.2, 67.9, 59.8, 49.9, 36.9, 27.8. HRMS (ESI, m/z): calcd. for $C_{18}H_{16}O_4S$ + Na^+ [M + Na]$^+$: 351.0662; found: 351.0663.

1-(Phenylsulfonyl)-6-(p-tolyl)-3-oxabicyclo[3.1.0]hexan-2-one (2ba)

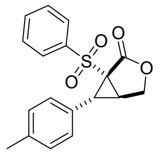

Purified by flash chromatography with EA/PE (1/4) as eluent, 85% yield, 55.9 mg; white solid. mp 207 - 213 °C. d.r. > 20 : 1; ^{1}H NMR (400 MHz, $CDCl_3$) δ 7.57 – 7.53 (m, 1H), 7.50 – 7.45 (m, 2H), 7.34 – 7.31 (m, 2H), 7.07 (d, J = 8.0 Hz, 2H), 6.93 (d, J = 8.0 Hz, 2H), 4.61 (dd, J = 9.5, 4.8 Hz, 1H), 4.37 (d, J = 9.5 Hz, 1H), 3.81 (dd, J = 6.3, 4.7 Hz, 1H), 2.84 (d, J = 6.3 Hz, 1H), 2.37 (s, 3H). ^{13}C NMR (100 MHz, $CDCl_3$) δ 168.6, 138.6, 133.9, 129.8, 128.9, 128.8, 128.7, 128.5, 124.9, 67.6, 52.7, 37.2, 28.0, 21.3. HRMS (ESI, m/z): calcd. for $C_{18}H_{16}O_4S$ + Na^+ [M + Na]$^+$: 351.0662; found: 351.0659.

1-(Phenylsulfonyl)-6-(*m*-tolyl)-3-oxabicyclo[3.1.0]hexan-2-one (2bb)

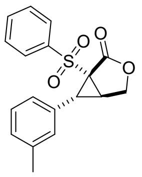

Purified by flash chromatography with EA/PE (1/5) as eluent, 74% yield, 48.9 mg; white solid. mp 155 - 160 °C. d.r. > 20 : 1; ^{1}H NMR (400 MHz, $CDCl_3$) δ 7.57 – 7.53 (m, 1H), 7.48 – 7.45 (m, 2H), 7.36 –

7.30 (m, 2H), 7.19 – 7.12 (m, 2H), 6.88 – 6.86 (m, 1H), 6.73 – 6.72 (m, 1H), 4.62 (dd, *J* = 9.5, 4.7 Hz, 1H), 4.37 (d, *J* = 9.5 Hz, 1H), 3.82 (dd, *J* = 6.3, 4.7 Hz, 1H), 2.84 (d, *J* = 6.2 Hz, 1H), 2.24 (s, 3H). ^{13}C NMR (100 MHz, $CDCl_3$) δ 168.6, 138.6, 137.8, 133.8, 131.1, 129.4, 128.7, 128.5, 128.2, 127.7, 126.7, 67.6, 52.6, 37.3, 28.0, 21.3. HRMS (ESI, m/z): calcd. for $C_{18}H_{16}O_4S + Na^+$ $[M + Na]^+$: 351.0662; found: 351.0667.

1-(Phenylsulfonyl)-6-(*o*-tolyl)-3-oxabicyclo[3.1.0]hexan-2-one (2bc)

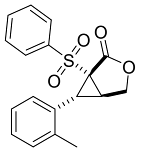

Purified by flash chromatography with EA/PE (1/4) as eluent, 75% yield, 49.1 mg; white solid. mp 128 - 133 °C. d.r. > 20 : 1; ^{1}H NMR (400 MHz, $CDCl_3$) δ 7.60 – 7.56 (m, 1H), 7.41 – 7.38 (m, 2H), 7.34 – 7.27 (m, 4H), 7.25 – 7.22 (m, 1H), 6.97 – 6.95 (m, 1H), 4.66 (dd, *J* = 9.5, 4.8 Hz, 1H), 4.37 (d, *J* = 9.5 Hz, 1H), 3.89 (dd, *J* = 6.4, 4.8 Hz, 1H), 2.77 (d, *J* = 6.4 Hz, 1H), 1.52 (s, 3H). ^{13}C NMR (100 MHz, $CDCl_3$) δ 168.8, 139.7, 138.1, 134.2, 130.0, 128.9, 128.9, 128.6, 128.5, 126.2, 125.9, 67.8, 52.1, 35.8, 27.4, 18.8. HRMS (ESI, m/z): calcd. for $C_{18}H_{16}O_4S + Na^+$ $[M + Na]^+$: 351.0662; found: 351.0665.

6-(4-Methoxyphenyl)-1-(phenylsulfonyl)-3-oxabicyclo[3.1.0]hexan-2-one (2bd)

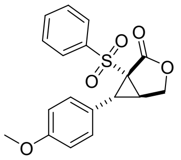

Purified by flash chromatography with EA/PE (1/3) as eluent, 58% yield, 40.0 mg; white solid. mp 170 - 175 °C. d.r. = 2 : 1; ^{1}H NMR (400 MHz, $CDCl_3$) δ 7.57 – 7.53 (m, 1H), 7.50 – 7.47 (m, 2H), 7.36 – 7.32 (m, 2H), 6.96 – 6.94 (m, 2H), 6.79 – 6.77 (m, 2H), 4.61 (dd, *J* = 9.5, 4.8 Hz, 1H), 4.37 (d, *J* = 9.4 Hz, 1H), 3.83 (s, 3H), 3.78 (dd, *J* = 6.1, 4.6 Hz, 1H), 2.83 (d, *J* = 6.2 Hz, 1H). ^{13}C NMR (100 MHz, $CDCl_3$) δ 168.6, 159.9, 138.7, 133.9, 131.1, 128.7, 128.6, 119.8, 113.6, 67.6, 55.5, 52.6, 37.1, 28.2. HRMS (ESI, m/z): calcd. for $C_{18}H_{16}O_5S + Na^+$ $[M + Na]^+$: 367.0611; found: 367.0594.

6-(4-Chlorophenyl)-1-(phenylsulfonyl)-3-oxabicyclo[3.1.0]hexan-2-one (2be)

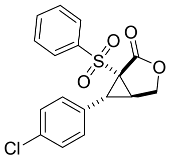

Purified by flash chromatography with EA/PE (1/3) as eluent, 76% yield, 52.8 mg; white solid. mp 180 - 185 °C. d.r. > 20 : 1; ^{1}H NMR (400 MHz, $CDCl_3$) δ 7.60 – 7.56 (m, 1H), 7.53 – 7.51 (m, 2H), 7.41 –

7.35 (m, 2H), 7.24 – 7.21 (m, 2H), 6.98 – 6.96 (m, 2H), 4.62 (dd, *J* = 9.6, 4.8 Hz, 1H), 4.39 (d, *J* = 9.6 Hz, 1H), 3.79 (dd, *J* = 6.2, 4.7 Hz, 1H), 2.83 (d, *J* = 6.0 Hz, 1H). ^{13}C NMR (100 MHz, $CDCl_3$) δ 168.3, 138.5, 134.7, 134.2, 131.2, 128.7, 128.5, 128.4, 126.7, 67.5, 52.7, 36.5, 28.2. HRMS (ESI, m/z): calcd. for $C_{17}H_{13}{}^{35}ClO_4S + Na^+$ $[M + Na]^+$: 371.0115; found: 371.0119.

6-(3,4-Dichlorophenyl)-1-(phenylsulfonyl)-3-oxabicyclo[3.1.0]hexan-2-one (2bf)

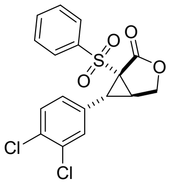

Purified by flash chromatography with EA/PE (1/6) as eluent, 90% yield, 68.6 mg; white solid. mp 191 - 200 ^{o}C. d.r. = 20 : 1; ^{1}H NMR (400 MHz, $CDCl_3$) δ 7.65 – 7.60 (m, 1H), 7.59 – 7.57 (m, 2H), 7.44 – 7.40 (m, 2H), 7.38 – 7.36 (m, 1H), 7.00 (d, *J* = 2.1 Hz, 1H), 6.95 (dd, *J* = 8.4, 2.2 Hz, 1H), 4.63 (dd, *J* = 9.6, 4.7 Hz, 1H), 4.39 (d, *J* = 9.6 Hz, 1H), 3.77 (dd, *J* = 6.1, 4.7 Hz, 1H), 2.81 (d, *J* = 6.1 Hz, 1H). ^{13}C NMR (100 MHz, $CDCl_3$) δ 168.0, 138.3, 134.4, 133.0, 132.5, 132.3, 130.2, 128.8, 128.8, 128.5, 128.4, 67.4, 52.6, 35.7, 28.2. HRMS (ESI, m/z): calcd. for $C_{17}H_{12}{}^{35}Cl_2O_4S + Na^+$ $[M + Na]^+$: 404.9726; found: 404.9730.

1-(Phenylsulfonyl)-6-(4-(trifluoromethyl)phenyl)-3-oxabicyclo[3.1.0]hexan-2-one (2bg)

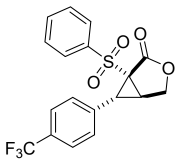

Purified by flash chromatography with EA/PE (1/6) as eluent, 83% yield, 63.7 mg; white solid. mp 191 - 197 ^{o}C. d.r. > 20 : 1; ^{1}H NMR (400 MHz, $CDCl_3$) δ 7.59 – 7.55 (m, 1H), 7.51 – 7.46 (m, 4H), 7.35 – 7.31 (m, 2H), 7.16 (d, *J* = 8.1 Hz, 2H), 4.65 (dd, *J* = 9.6, 4.7 Hz, 1H), 4.42 (d, *J* = 9.8 Hz, 1H), 3.87 (dd, *J* = 6.2, 4.6 Hz, 1H), 2.91 (d, *J* = 6.2 Hz, 1H). ^{19}F NMR (376 MHz, $CDCl_3$) δ -63.4 (s, 3F). ^{13}C NMR (100 MHz, $CDCl_3$) δ 168.1, 138.5, 134.2, 132.2, 130.8 (q, J_{C-F} = 32.7 Hz), 130.3, 128.8, 128.4, 125.1 (q, J_{C-F} = 3.6 Hz), 123.9 (q, J_{C-F} = 270.8 Hz), 67.5, 52.8, 36.4, 28.2. HRMS (ESI, m/z): calcd. for $C_{18}H_{13}F_3O_4S + Na^+$ $[M + Na]^+$: 405.0379; found: 405.0380.

6-(4-Nitrophenyl)-1-(phenylsulfonyl)-3-oxabicyclo[3.1.0]hexan-2-one (2bh)

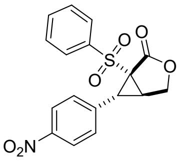

Purified by flash chromatography with EA/PE (1/3) as eluent, 43% yield, 31.0 mg; white solid. mp 149 - 152 °C. d.r. > 20 : 1; ^{1}H NMR (400 MHz, $(CD_3)_2SO$) δ 8.13 (d, *J* = 8.2 Hz, 2H), 7.76 – 7.68 (m, 1H), 7.53 – 7.46 (m, 6H), 4.64 (dd, *J* = 9.6, 4.7 Hz, 1H), 4.47 (d, *J* = 9.5 Hz, 1H), 4.31 (t, *J* = 5.6 Hz, 1H), 3.60 (d, *J* = 6.3 Hz, 1H). ^{13}C NMR (100 MHz, $(CD_3)_2SO$) δ 168.6, 147.6, 139.0, 137.9, 135.0, 131.9, 129.6, 128.3, 123.2, 68.3, 53.1, 35.8, 29.5. HRMS (ESI, m/z): calcd. for $C_{17}H_{13}NO_6S + Na^+$ $[M + Na]^+$: 382.0356; found: 382.0369.

6-Methyl-1-(phenylsulfonyl)-3-oxabicyclo[3.1.0]hexan-2-one (2bi) [15]

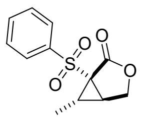

Purified by flash chromatography with EA/PE (1/5) as eluent, 46% yield, 23.3 mg; white solid. mp 169 - 178 °C. d.r. = 10 : 1; ^{1}H NMR (400 MHz, $CDCl_3$, two diastereoisomers) δ the major isomer 8.16 – 8.13 (m, 2H), 7.71 – 7.67 (overlapped, 1.1H), 7.61 – 7.57 (overlapped, 2.2H), 4.40 (dd, *J* = 9.5, 4.6 Hz, 1H), 4.23 (d, *J* = 9.5 Hz, 1H), 3.00 (t, *J* = 5.0 Hz, 1H), 1.75 – 1.70 (m, 1H), 1.51 (d, *J* = 6.4 Hz, 3H); the minor isomer 8.05 – 8.02 (m, 2H), 7.71 – 7.67 (overlapped, 1H), 7.61 – 7.57 (overlapped, 2H), 4.25 (dd, *J* = 10.2, 5.3 Hz, 1H), 4.13 (d, *J* = 10.2 Hz, 1H), 3.17 – 3.13 (m, 1H), 2.53 – 2.50 (m, 1H), 1.13 (d, *J* = 6.5 Hz, 3H). ^{13}C NMR (100 MHz, $CDCl_3$, two diastereoisomers) δ the major isomer 169.0 (overlapped), 139.5 (overlapped), 134.3, 129.2, 128.9, 67.5, 50.9 (overlapped), 31.6, 29.1, 10.8; the minor isomer 169.0 (overlapped), 139.5 (overlapped), 134.4, 129.3, 129.0, 63.8, 50.9 (overlapped), 31.4, 25.1, 7.5.

6,6-Dimethyl-1-(phenylsulfonyl)-3-oxabicyclo[3.1.0]hexan-2-one (2bj)

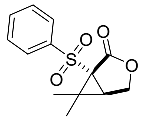

Purified by flash chromatography with EA/PE (1/4) as eluent, 61% yield, 32.5 mg; colorless oil. d.r. > 20 : 1; ^{1}H NMR (400 MHz, $CDCl_3$) δ 8.13 – 8.10 (m, 2H), 7.69 – 7.65 (m, 1H), 7.60 – 7.55 (m, 2H), 4.48 (dd, *J* = 10.2, 5.4 Hz, 1H), 4.15 (dd, *J* = 10.0, 0.9 Hz, 1H), 3.01 (dd, *J* = 5.2, 1.0 Hz, 1H), 1.52 (s, 3H), 1.16 (s, 3H). ^{13}C NMR (100 MHz, $CDCl_3$) δ 168.4, 140.0, 134.2, 129.2, 128.5, 64.9, 56.8, 36.6, 33.0, 19.9, 16.9. HRMS (ESI, m/z): calcd. for $C_{13}H_{14}O_4S + Na^+$ $[M + Na]^+$: 289.0505; found: 289.0508.

4-Methyl-6-phenyl-1-(phenylsulfonyl)-3-oxabicyclo[3.1.0]hexan-2-one (2bk)

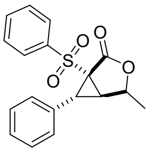

Purified by flash chromatography with EA/PE (1/3) as eluent, 65% yield, 42.7 mg; white solid. mp 210 - 216 ^{o}C. d.r. > 20 : 1 : 1; ^{1}H NMR (400 MHz, $CDCl_3$) δ 7.57 – 7.52 (m, 1H), 7.48 – 7.44 (m, 2H), 7.36 – 7.31 (m, 3H), 7.28 – 7.24 (m, 2H), 7.07 – 7.05 (m, 2H), 4.65 (q, *J* = 6.3 Hz, 1H), 3.57 (d, *J* = 6.2 Hz, 1H), 2.89 (d, *J* = 6.2 Hz, 1H), 1.60 (d, *J* = 6.3 Hz, 3H). ^{13}C NMR (100 MHz, $CDCl_3$) δ 167.9, 138.4, 134.0, 130.0, 128.8, 128.6, 128.6, 128.3, 128.2, 76.0, 53.2, 37.7, 33.6, 22.0. HRMS (ESI, m/z): calcd. for $C_{18}H_{16}O_4S + Na^+$ $[M + Na]^+$: 351.0662; found: 351.0663.

5-Methyl-6-phenyl-1-(phenylsulfonyl)-3-oxabicyclo[3.1.0]hexan-2-one (2bl)

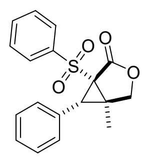

Purified by flash chromatography with EA/PE (1/4) as eluent, 54% yield, 35.4 mg; white solid. mp 135 - 140 ^{o}C. d.r. > 20 : 1 : 1; ^{1}H NMR (400 MHz, $CDCl_3$) δ 7.93 – 7.90 (m, 2H), 7.67 – 7.62 (m, 1H), 7.54 – 7.47 (m, 4H), 7.40 – 7.33 (m, 3H), 4.33 (d, *J* = 9.3 Hz, 1H), 4.03 (d, *J* = 9.2 Hz, 1H), 3.07 (s, 1H), 1.90 (s, 3H). ^{13}C NMR (100 MHz, $CDCl_3$) δ 169.5, 139.9, 134.4, 130.4, 129.9, 129.1, 129.1, 128.5, 128.4, 73.3, 52.4, 40.0, 37.4, 12.0. HRMS (ESI, m/z): calcd. for $C_{18}H_{16}O_4S + Na^+$ $[M + Na]^+$: 351.0662; found: 351.0664.

1-(Methylsulfonyl)-6-phenyl-3-oxabicyclo[3.1.0]hexan-2-one (2bm)

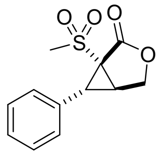

Purified by flash chromatography with EA/PE (1/4) as eluent, 64% yield, 32.5 mg; white solid. mp 152 - 156 ^{o}C. d.r. > 20 : 1; ^{1}H NMR (400 MHz, $CDCl_3$) δ 7.40 – 7.36 (m, 3H), 7.35 – 7.32 (m, 2H), 4.62 (dd, *J* = 9.4, 4.7 Hz, 1H), 4.43 (d, *J* = 9.6 Hz, 1H), 3.73 (dd, *J* = 6.1, 4.7 Hz, 1H), 3.06 (d, *J* = 6.1 Hz, 1H), 2.91 (s, 3H). ^{13}C NMR (100 MHz, $CDCl_3$) δ 169.2, 129.5, 129.1, 128.8, 128.5, 68.1, 51.1, 41.2, 37.0, 26.5. HRMS (ESI, m/z): calcd. for $C_{12}H_{12}O_4S + Na^+$ $[M + Na]^+$: 275.0349; found: 275.0354.

1-(Ethylsulfonyl)-6-phenyl-3-oxabicyclo[3.1.0]hexan-2-one (2bn)

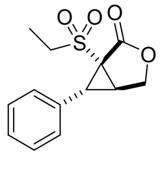

Purified by flash chromatography with EA/PE (1/4) as eluent, 72% yield, 38.6 mg; white solid. mp 138 - 141°C. d.r. > 20 : 1; ^{1}H NMR (400 MHz, $CDCl_3$) δ 7.39 – 7.32 (m, 5H), 4.62 (dd, *J* = 9.6, 4.7 Hz, 1H),

4.43 (d, J = 9.6 Hz, 1H), 3.72 (dd, J = 6.2, 4.7 Hz, 1H), 3.33 – 3.24 (m, 1H), 3.05 (d, J = 6.2 Hz, 1H), 2.81 – 2.72 (m, 1H), 1.22 (t, J = 7.5 Hz, 3H). ^{13}C NMR (100 MHz, $CDCl_3$) δ 169.2, 129.6, 129.0, 128.8, 128.7, 68.1, 50.8, 48.0, 37.0, 26.8, 5.2. HRMS (ESI, m/z): calcd. for $C_{13}H_{14}O_4S + Na^+$ $[M + Na]^+$: 289.0505; found: 289.0509.

2-Oxo-6-phenyl-3-oxabicyclo[3.1.0]hexane-1-carbonitrile (2bo) [16]

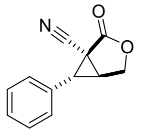

Purified by flash chromatography with EA/PE (1/4) as eluent, 63% yield, 25.2 mg; yellow oil. d.r. > 20 : 1; ^1H NMR (400 MHz, $CDCl_3$) δ 7.44 – 7.37 (m, 3H), 7.27 – 7.24 (m, 2H), 4.59 (dd, J = 9.7, 4.7 Hz, 1H), 4.43 (d, J = 9.7 Hz, 1H), 3.37 (t, J = 5.2 Hz, 1H), 2.93 (d, J = 5.5 Hz, 1H). ^{13}C NMR (100 MHz, $CDCl_3$) δ 168.3, 130.8, 129.3, 129.2, 127.9, 113.1, 68.5, 36.5, 30.6, 26.2.

1-Benzoyl-6-phenyl-3-oxabicyclo[3.1.0]hexan-2-one (2bp) [17]

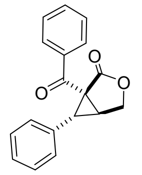

Purified by flash chromatography with EA/PE (1/4) as eluent, 52% yield, 29.2 mg; white solid. mp 152 - 156 °C. d.r. > 20 : 1; ^1H NMR (400 MHz, $CDCl_3$) δ 7.90 – 7.82 (m, 2H), 7.50 – 7.46 (m, 1H), 7.38 – 7.33 (m, 2H), 7.19 – 7.07 (m, 5H), 4.59 (dd, J = 9.4, 4.9 Hz, 1H), 4.47 (d, J = 9.4 Hz, 1H), 3.46 (t, J = 5.0 Hz, 1H), 3.05 (d, J = 5.2 Hz, 1H). ^{13}C NMR (100 MHz, $CDCl_3$) δ 189.9, 171.2, 135.5, 133.9, 131.9, 130.2, 128.7, 128.2, 128.1, 127.7, 68.1, 44.7, 38.3, 26.2.

1-Acetyl-6-phenyl-3-oxabicyclo[3.1.0]hexan-2-one (2bq) [16]

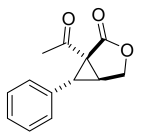

Purified by flash chromatography with EA/PE (1/10) as eluent, 58% yield, 25.2 mg; yellow oil. d.r. > 20 : 1; ^1H NMR (400 MHz, $CDCl_3$) δ 7.34 – 7.28 (m, 3H), 7.19 – 7.16 (m, 2H), 4.45 (dd, J = 9.4, 4.7 Hz, 1H), 4.37 (d, J = 9.3 Hz, 1H), 3.38 (t, J = 5.2 Hz, 1H), 3.00 (d, J = 5.6 Hz, 1H), 2.35 (s, 3H). ^{13}C NMR (100 MHz, $CDCl_3$) δ 197.0, 172.1, 131.1, 128.8, 128.6, 128.4, 67.6, 45.5, 40.7, 29.7, 27.1.

Methyl 2-oxo-6-phenyl-3-oxabicyclo[3.1.0]hexane-1-carboxylate (2br) [18]

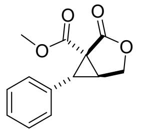

Purified by flash chromatography with EA/PE (1/5) as eluent, 64% yield, 29.8 mg; white solid. mp 115 - 121 °C. d.r. > 20 : 1; ^{1}H NMR (400 MHz, $CDCl_3$) δ 7.35 – 7.29 (m, 3H), 7.26 – 7.23 (m, 2H), 4.51 (dd, *J* = 9.4, 4.8 Hz, 1H), 4.37 (d, *J* = 9.4 Hz, 1H), 3.52 (s, 3H), 3.31 (t, *J* = 5.2 Hz, 1H), 2.93 (d, *J* = 5.6 Hz, 1H). ^{13}C NMR (100 MHz, $CDCl_3$) δ 170.1, 164.1, 131.8, 128.8, 128.6, 128.4, 67.4, 52.8, 37.9, 37.7, 27.8.

1-(4-Nitrophenyl)-6-phenyl-3-oxabicyclo[3.1.0]hexan-2-one (2bs)

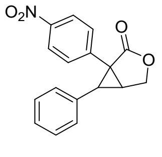

Purified by flash chromatography with EA/PE (1/3.5) as eluent, 34% yield, 20.0 mg; green oil. d.r. = 4 : 1; ^{1}H NMR (400 MHz, $CDCl_{3,}$ two diastereoisomers) δ the major isomer 8.05 – 8.02 (m, 2H), 7.45 – 7.42 (m, 2H), 7.14 – 7.10 (m, 3H), 6.89 – 6.87 (m, 2H), 4.67 (dd, *J* = 9.4, 4.5 Hz, 1H), 4.56 (d, *J* = 9.4 Hz, 1H), 3.27 (t, *J* = 4.6 Hz, 1H), 2.87 (d, *J* = 4.6 Hz, 1H); the minor isomer 8.29 – 8.24 (m, 2H), 7.80 – 7.77 (m, 2H), 7.40 – 7.35 (m, 5H), 4.56 – 4.52 (overlapped, 1H), 4.23 (d, *J* = 10.0 Hz, 1H), 3.14 (d, 8.3 Hz, 1H), 3.08 (dd, *J* = 8.4, 4.8 Hz, 1H). ^{13}C NMR (100 MHz, $CDCl_{3,}$ two diastereoisomers) δ the major isomer 174.3, 147.3, 137.7, 132.4, 131.2, 128.6, 128.1, 127.8, 123.4, 68.4, 39.3, 36.4, 27.1; the minor isomer 173.4, 147.4, 142.4, 131.4, 129.3, 129.2, 129.1, 128.5, 124.1, 64.6, 38.1, 35.6, 31.0. HRMS (ESI, m/z): calcd. for $C_{17}H_{13}NO_4$ + H^+ $[M + H]^+$: 296.0917; found: 296.0905.

3-Phenyl-1-(phenylsulfonyl)-3-azabicyclo[3.1.0]hexan-2-one (2bt)

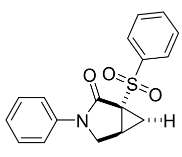

Purified by flash chromatography with EA/PE (1/4) as eluent, 30% yield, 18.9 mg; white solid. mp 160 - 165 °C. d.r. > 20 : 1; ^{1}H NMR (400 MHz, $CDCl_3$) δ 8.14 – 8.11 (m, 2H), 7.70 – 7.66 (m, 1H), 7.61 – 7.57 (m, 2H), 7.46 – 7.43 (m, 2H), 7.35 – 7.30 (m, 2H), 7.16 – 7.12 (m, 1H), 4.12 (dd, *J* = 10.6, 5.9 Hz, 1H), 3.72 (d, *J* = 10.6 Hz, 1H), 3.04 – 2.99 (m, 1H), 2.11 (dd, *J* = 8.6, 5.1 Hz, 1H), 1.40 (t, *J* = 5.2 Hz, 1H). ^{13}C NMR (100 MHz, $CDCl_3$) δ 165.5, 139.0, 138.3, 134.2, 129.3, 129.1, 129.1, 125.5, 120.3, 49.1, 48.0, 20..4, 20.1. HRMS (ESI, m/z): calcd. for $C_{17}H_{15}NO_3S$ + Na^+ $[M + Na]^+$: 336.0665; found: 336.0667.

(3-Phenyl-1-(phenylsulfonyl)cyclopropane-1,2-diyl)dimethanol (3)

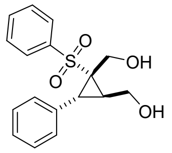

Purified by flash chromatography with EA/PE (1/6) as eluent, 72% yield, 46.0 mg; white solid. mp 132 - 136 ^{o}C. d.r. > 20 : 1; ^{1}H NMR (400 MHz, $CDCl_3$) δ 7.53 – 7.49 (m, 1H), 7.32 – 7.26 (m, 2H), 7.25 – 7.22 (m, 3H), 7.07 – 7.05 (m, 4H), 4.64 (d, *J* = 14.2 Hz, 1H), 4.28 (dd, *J* = 12.6, 4.9 Hz, 1H), 3.62 – 3.55 (m, 4H), 3.34 – 3.28 (m, 1H), 2.43 (d, *J* = 7.9 Hz, 1H). ^{13}C NMR (100 MHz, $CDCl_3$) δ 137.7, 133.7, 130.9, 130.2, 128.8, 128.6, 128.2, 127.8, 63.3, 61.8, 54.5, 35.8, 27.7. HRMS (ESI, m/z): calcd. for $C_{17}H_{18}O_4S + Na^+$ $[M + Na]^+$: 341.0818; found: 341.0824.

2-(Hydroxymethyl)-N,3-diphenyl-1-(phenylsulfonyl)cyclopropane-1-carboxamide (4)

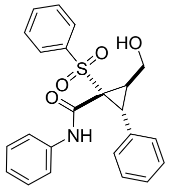

Purified by flash chromatography with EA/PE (1/6) as eluent, 89% yield, 72.5 mg; colorless oil. d.r. > 20 : 1; ^{1}H NMR (400 MHz, $CDCl_3$) δ 9.40 (s, 1H), 7.57 – 7.53 (m, 3H), 7.41 – 7.27 (m, 11H), 7.22 – 7.18 (m, 1H), 4.26 (dd, *J* = 12.0, 4.7 Hz, 1H), 3.62 (dd, *J* = 12.0, 9.0 Hz, 1H), 3.45 – 3.35 (m, 2H), 2.17 (br, 1H). ^{13}C NMR (100 MHz, $CDCl_3$) δ 161.7, 138.5, 137.3, 134.1, 131.0, 130.0, 129.3, 129.0, 128.1, 128.0, 125.4, 120.4, 61.6, 57.5, 35.2, 31.6. HRMS (ESI, m/z): calcd. for $C_{23}H_{21}NO_4S + Na^+$ $[M + Na]^+$: 430.1084; found: 430.1095.

2-Ethyl-6-phenyl-1-(phenylsulfonyl)-3-oxabicyclo[3.1.0]hexan-2-ol (5)

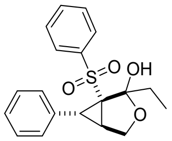

Purified by flash chromatography with EA/PE (1/7) as eluent, 53% yield, 36.3 mg; colorless oil. d.r. = 7 : 1; ^{1}H NMR (400 MHz, $CDCl_3$, two diastereoisomers) δ the major isomer 7.57 – 7.52 (overlapped, 1H), 7.35 – 7.23 (overlapped, 7H), 7.14 – 7.11 (m, 2H), 5.49 (overlapped, 1H), 4.30 (dd, *J* = 8.8, 2.7 Hz, 1H), 3.94 (d, *J* = 8.8 Hz, 1H), 3.27 (dd, *J* = 6.1, 2.6 Hz, 1H), 2.77 (d, *J* = 6.2 Hz, 1H), 1.72 (q, overlapped, *J* = 7.5 Hz, 2H), 1.01 (t, *J* = 7.4 Hz, 3H); the minor isomer 7.57 – 7.52 (overlapped, 1H), 7.46 – 7.40 (m, 4H), 7.35 – 7.23 (overlapped, 5H), 5.49 (overlapped, 1H), 4.15 – 7.09 (m, 1H), 3.41 – 3.36 (m, 1H), 3.18

– 3.15 (m, 1H), 3.01 (d, J = 8.3 Hz, 1H), 1.75 – 1.64 (overlapped, 2H), 1.11 (t, J = 7.1 Hz, 3H). ^{13}C NMR (100 MHz, $CDCl_3$, two diastereoisomers) δ the major isomer 139.0 (overlapped), 133.8, 131.3 (overlapped), 130.1, 128.7, 128.4, 128.1, 127.8, 105.5 (overlapped), 65.7, 58.9, 33.1, 30.7, 28.6, 7.9; the minor isomer 139.0 (overlapped), 133.9, 131.3 (overlapped), 129.7, 129.0, 128.3, 128.2, 127.9, 105.5 (overlapped), 61.6, 61.3, 37.0, 34.2, 32.6, 8.2. HRMS (ESI, m/z): calcd. for $C_{19}H_{20}O_4S + Na^+$ $[M + Na]^+$: 367.0975; found: 367.0981.

4-Benzyl-3-(phenylsulfonyl)dihydrofuran-2(3H)-one (6)

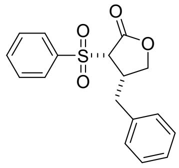

Purified by flash chromatography with EA/PE (1/5) as eluent, 59% yield, 37.3 mg; colorless oil. d.r. > 20 : 1; ^{1}H NMR (400 MHz, $CDCl_3$) δ 7.90 – 7.85 (m, 2H), 7.73 – 7.69 (m, 1H), 7.60 – 7.55 (m, 2H), 7.35 – 7.27 (m, 3H), 7.17 – 7.14 (m, 2H), 4.52 (dd, J = 9.3, 7.1 Hz, 1H), 4.11 (dd, J = 9.2, 3.8 Hz, 1H), 3.80 (d, J = 4.2 Hz, 1H), 3.57 – 3.51 (m, 1H), 3.00 – 2.88 (m, 2H). ^{13}C NMR (100 MHz, $CDCl_3$) δ 168.0, 136.8, 136.3, 134.8, 129.4, 129.4, 129.2, 129.1, 127.5, 71.5, 68.2, 38.7, 38.3. HRMS (ESI, m/z): calcd. for $C_{17}H_{16}O_4S + Na^+$ $[M + Na]^+$: 339.0662; found: 339.0659.

Cinnamyl 2-(phenylsulfonyl)-2-((2,2,6,6-tetramethylpiperidin-1-yl)oxy)acetate] (7)

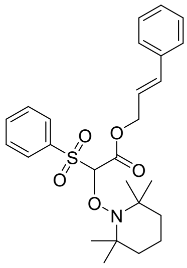

Purified by flash chromatography with EA/PE (1/40) as eluent, 22% yield, 21.1 mg; colorless oil. ^{1}H NMR (400 MHz, $CDCl_3$) δ 7.96 – 7.94 (m, 2H), 7.68 – 7.64 (m, 1H), 7.54 – 7.50 (m, 2H), 7.40 – 7.27 (m, 5H), 6.65 (d, J = 15.8 Hz, 1H), 6.22 (dt, J = 15.9, 6.6 Hz, 1H), 5.55 (s, 1H), 4.80 – 4.76 (m, 2H), 1.47 – 1.40 (m, 8H), 1.29 – 1.23 (m, 4H), 0.95 – 0.89 (m, 6H). ^{13}C NMR (100 MHz, $CDCl_3$) δ 165.7, 136.3, 136.1, 135.4, 134.5, 130.5, 128.8, 128.6, 128.4, 126.8, 121.7, 98.7, 66.7, 60.8, 40.8, 32.9, 20.6, 16.8. HRMS (ESI, m/z): calcd. for $C_{26}H_{33}NO_5S + Na^+$ $[M + Na]^+$: 494.1972; found: 494.1978.

4-(Bromo(phenyl)methyl)-3-methyl-3-(phenylsulfonyl)dihydrofuran-2(3H)-one (9)

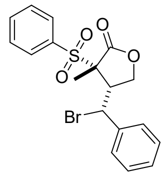

Purified by flash chromatography with EA/PE (1/10) as eluent, 23% yield, 19.1 mg; colorless oil. d.r. = 16 : 5 : 1; ^{1}H NMR (400 MHz, $CDCl_3$) δ 7.72 – 7.61 (m, 5H), 7.53 – 7.49 (m, 2H), 7.44 – 7.35 (m, 3H), 6.08(d, *J* = 12.0 Hz, 1H), 4.88 (dd, *J* = 11.4, 8.8 Hz, 1H), 4.77 (dd, *J* = 9.0, 8.8 Hz, 1H), 3.75 – 3.68 (m, 1H), 0.99 (s, 3H). ^{13}C NMR (100 MHz, $CDCl_3$) δ 172.0, 139.5, 135.3, 135.1, 130.6, 129.4, 128.8, 128.7, 128.6, 72.0, 71.1, 54.0, 50.1, 19.9. HRMS (ESI, m/z): calcd. for $C_{18}H_{17}{}^{79}BrO_4S + Na^+$ $[M + Na]^+$: 430.9923; found: 430.9904.

9. Single-crystal X-ray diffraction data for 2aa

Preparation of the single crystals of 2aa: 15.0 mg of pure **2aa** was dissolved in the combined solvents of petroleum ether and CH_2Cl_2 (5 mL, v/v = 9:1) at room temperature. The bottle was sealed by a piece of plastic film with several tiny holes, thus allowing the slow solvent evaporation at 0 °C. After about two days, several small crystals were observed at the bottom of the bottle. The crystals were collected and subjected to the single crystal X-ray diffraction analysis for the determination of the structure of **2aa**

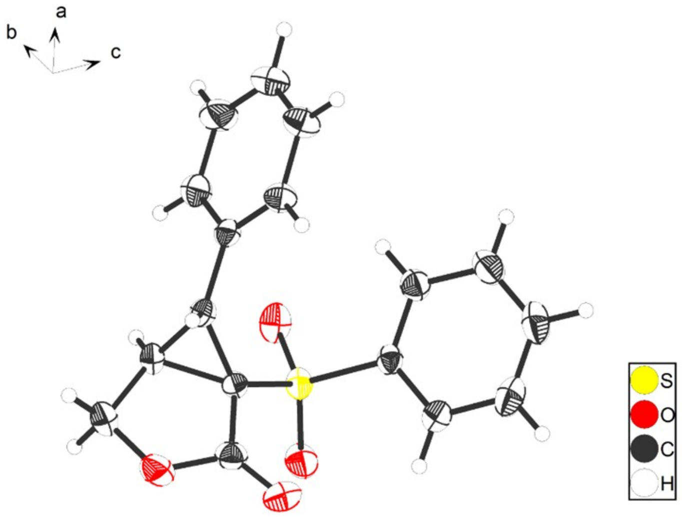

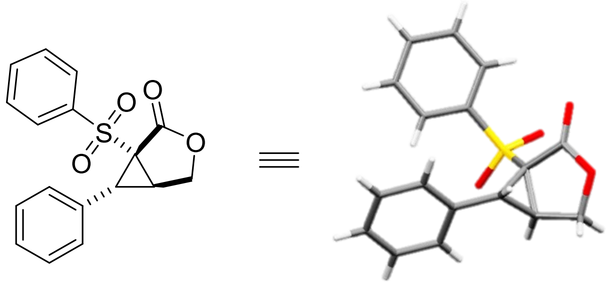

(50% probability ellipsoids)

Table S7. Crystallographic data and structure refinement for compound (2aa).

Compound	2aa
Formula	$C_{17}H_{14}O_4S$
M, g mol^{-1}	314.34
Crystal system	monoclinic
Space group	$P2_1/n$
a, Å	13.8755(13)
b, Å	7.1896(5)
c, Å	14.6846(13)

α, deg	90
β, deg	93.679(3)
γ, deg	90
V, Å^3	1461.9(2)
Z	4
d_{cal}, g cm^{-3}	1.428
F(000)	656.0
Radiation	MoKα(λ = 0.71073)
Temperature, K	300
2θ range, deg	3.916 to 52.762
Completeness	0.997
Residual map, e Å^{-3}	0.20/-0.33
Goodness-of-fit on F^2	1.015
Final indices [I>2$\sigma(I)$]	R_1 = 0.0403,<br>wR_2 = 0.0931
R indices (all data)	R_1 =0.0606,<br>wR_2 = 0.1050

10. Computational Details

For this study, all DFT calculations were conducted using Gaussian 16 Rev. A.03.[19] To ensure the accuracy of results, molecular geometry optimizations and frequency calculations were performed with the B3LYP[20] functional with D3(BJ) dispersion correction[21], and a mixed basis set was employed using SDD[22] for Cu and Br, while 6-31G(d)[23] for all other atoms. Frequency calculations were crucial in determining stationary points (no imaginary frequencies) and transition state structures (only one imaginary frequency) in the system. Single point energies based upon the optimized structures were calculated at B3LYP-D3/Def2-TZVP[24] level with SMD[25] solvation model (DMF as solvent). The visualization of molecular structure was generated through the utilization of CYLview 1.0.[26]

The Cartesian Coordinates of calculated structures

1aa

C	-0.18648600	1.13417000	-0.13002200
H	0.49272200	0.33829600	-0.43801500
H	-0.83447500	1.44932800	-0.94850200
C	0.56953600	2.31524200	0.44531300
O	1.78264800	1.92694700	0.86738200
O	0.12194300	3.43494100	0.53594200
C	2.55212900	2.94772000	1.56498300
H	2.58211200	3.83837600	0.92772800
H	2.02331300	3.20735700	2.48648700
C	3.90924500	2.39417000	1.83247300
H	4.49175800	2.11722400	0.95613900
C	4.40434800	2.25015100	3.06937000
H	3.76120500	2.51669400	3.90837900
C	5.73437500	1.75620400	3.44395700
C	6.01153200	1.52258800	4.80090900
C	6.75115300	1.50328800	2.50592500
C	7.25343600	1.04129500	5.20990000
H	5.23742600	1.71739700	5.53899100
C	7.99116000	1.02280800	2.91271200
H	6.57302700	1.69335700	1.45197400
C	8.24870300	0.78788400	4.26643300
H	7.44336500	0.86572200	6.26497200
H	8.76389700	0.83519500	2.17237700
H	9.21928400	0.41525900	4.58093300
S	-1.27761300	0.43373100	1.17643400
O	-2.52589700	1.20552700	1.19393700
O	-0.45550600	0.27178300	2.38352500
C	-1.63625600	-1.19605400	0.51909000
C	-2.76965600	-1.37217000	-0.27399800

H	-3.43504800	-0.53498700	-0.45536300
C	-3.03112200	-2.63810900	-0.79745500
H	-3.91158100	-2.79608000	-1.41289700
C	-2.16710800	-3.70042700	-0.52338100
H	-2.37588800	-4.68466100	-0.93243100
C	-1.04048700	-3.50830900	0.28037800
H	-0.37936800	-4.34105500	0.50041100
C	-0.76746200	-2.24834300	0.81025700
H	0.08943600	-2.07729300	1.45327100

TS1

C	-0.66445200	0.24579400	0.43707300
H	-0.04943700	-0.64159200	0.58840900
H	-0.55370700	0.44032000	-0.87162800
C	-0.17081900	1.45900200	1.15500500
O	1.15478600	1.34913100	1.37471800
O	-0.84217600	2.42084100	1.45967900
C	1.78251700	2.51123400	1.99004500
H	1.40122300	3.40266600	1.47959100
H	1.47055700	2.56378100	3.03738200
C	3.25710700	2.35983100	1.84621800
H	3.62573500	2.27815200	0.82583000
C	4.09425100	2.34394300	2.89270500
H	3.66110400	2.40865900	3.89133300
C	5.55778000	2.24547700	2.86216300
C	6.25354100	2.14157300	4.07781000
C	6.30257200	2.24825100	1.66916300
C	7.64242400	2.03477200	4.10604200
H	5.69258300	2.14153300	5.00915600
C	7.68868400	2.14117400	1.69586000
H	5.79419700	2.34082300	0.71441300
C	8.36605800	2.03305100	2.91397600
H	8.15833900	1.95384000	5.05854500
H	8.24585900	2.14565900	0.76323000
H	9.44900800	1.95171400	2.93100300
S	-2.41096800	-0.13293700	0.70215300
O	-3.24147800	0.82549000	-0.03331600
O	-2.57084700	-0.32892500	2.15394900
C	-2.55201900	-1.73661300	-0.08778500
C	-2.55746800	-1.81157200	-1.48203000
H	-2.44695800	-0.91212900	-2.07628400
C	-2.67565100	-3.06460700	-2.08042500
H	-2.67597900	-3.14515400	-3.16322800
C	-2.79889100	-4.21134300	-1.29186900

H	-2.89587400	-5.18390300	-1.76581200
C	-2.79885100	-4.11583400	0.10110400
H	-2.89930900	-5.00946500	0.70968700
C	-2.67119900	-2.87025700	0.71521500
H	-2.67295900	-2.76317900	1.79426900
C	-0.28872900	1.84254600	-2.51290200
C	-0.35201800	1.68521100	-4.04948400
H	-1.32417300	1.28542800	-4.35092900
H	-0.21242400	2.66640400	-4.51579900
H	0.43391700	1.01018300	-4.40020700
C	-1.43926800	2.73145400	-2.02576000
H	-2.40170500	2.29102100	-2.29831300
H	-1.42407700	2.84240400	-0.93854000
H	-1.36325900	3.72835500	-2.47400100
C	1.08251500	2.39374000	-2.09712600
H	1.16440600	2.44677700	-1.00759200
H	1.88062200	1.74124800	-2.46538900
H	1.23727400	3.40130000	-2.49776700
O	-0.43818000	0.50399700	-2.06268300

INT1

C	-0.16928700	0.44778300	1.07481500
H	0.40186300	-0.44295800	0.84114100
C	0.46106100	1.63840900	1.63287600
O	1.79012500	1.43499500	1.78536200
O	-0.12001300	2.66762400	1.92391600
C	2.52162500	2.56768600	2.32961800
H	2.28363300	3.44424800	1.71579000
H	2.16478400	2.76641700	3.34503800
C	3.97302800	2.23161500	2.29961500
H	4.38473500	1.99646200	1.32039500
C	4.74382000	2.23485100	3.39593500
H	4.26773400	2.45656500	4.35159600
C	6.18441600	1.96675600	3.47404200
C	6.79086400	1.89435100	4.73882200
C	6.99249300	1.77661300	2.33885300
C	8.15267000	1.63112800	4.87090000
H	6.18085800	2.04356000	5.62637500
C	8.35169800	1.51333700	2.46918300
H	6.55577000	1.84317100	1.34718500
C	8.93901500	1.43806500	3.73551200
H	8.59877900	1.57821900	5.85995100
H	8.95886200	1.37056800	1.57964700
H	10.00148200	1.23484200	3.83335600

S	-1.91004800	0.42589900	0.76973200
O	-2.19957000	1.38940700	-0.30206900
O	-2.60322100	0.49339800	2.06428100
C	-2.13407400	-1.22447400	0.10654900
C	-2.03155700	-1.41988200	-1.27086300
H	-1.84462600	-0.57295400	-1.92215000
C	-2.19596000	-2.70778800	-1.77885800
H	-2.12489400	-2.87913700	-2.84865500
C	-2.46029600	-3.77236300	-0.91400600
H	-2.58916300	-4.77343900	-1.31508700
C	-2.56899800	-3.55757200	0.46195600
H	-2.78680200	-4.38711200	1.12775400
C	-2.40700100	-2.27471200	0.98323800
H	-2.50552900	-2.07774000	2.04527500

INT2

S	9.58954900	1.58242100	5.31938300
O	10.97769100	1.60437700	4.82222000
O	8.52919700	0.89754800	4.56067100
O	6.89819900	3.81705200	4.76605900
O	7.11839800	3.25277000	6.93579300
C	9.60403400	0.97091800	6.99626600
C	10.78964800	1.05232800	7.72801000
H	11.68548700	1.44799600	7.26225400
C	10.79384800	0.59661000	9.04480500
H	11.70729800	0.64537200	9.62957700
C	9.62691900	0.07277800	9.60621300
H	9.63501800	-0.28024900	10.63330500
C	8.45082300	-0.00344000	8.85708000
H	7.54782800	-0.41297900	9.29871100
C	8.42993200	0.44867900	7.53935900
H	7.52916100	0.39914500	6.93993300
C	7.60997200	3.45171000	5.85557300
C	7.73790500	3.88041400	3.59152600
H	7.39414900	4.72405600	2.99077600
H	7.61483800	2.94791400	3.03678200
C	9.17910200	4.05151400	4.10555100
H	9.88708700	3.53270400	3.44995100
C	10.89039900	5.89984200	4.60140100
C	11.16830300	7.28145800	4.80813700
H	10.35583400	7.99705200	4.71084500
C	12.44360200	7.71624100	5.12710300
H	12.62982300	8.77579100	5.27880300
C	13.49296500	6.79567700	5.25627400

H	14.49225800	7.13860900	5.50714700
C	13.24406100	5.43183500	5.05962300
H	14.05420200	4.71454500	5.15801000
C	11.97206600	4.98091100	4.73774900
H	11.80590900	3.91823900	4.59173300
C	9.08765200	3.34342400	5.47838600
H	9.72897800	3.77276800	6.24843600
C	9.57763500	5.48529100	4.28079600
H	8.79414300	6.23753900	4.22943800

INT3

S	10.98732600	3.08140700	4.86151600
O	10.91815300	2.69718400	3.43674000
O	10.38319800	2.21308300	5.88201200
O	8.74500200	5.06859500	6.81749500
O	10.94337900	5.40311500	7.18509000
C	12.67088300	3.46459700	5.28199400
C	13.43341900	4.20210100	4.37301500
H	13.02239000	4.50985200	3.41708600
C	14.73416000	4.54899100	4.72949900
H	15.33944700	5.13250800	4.04335900
C	15.24978700	4.15188600	5.96645300
H	16.26417100	4.42760000	6.23901700
C	14.47046800	3.41234700	6.85875200
H	14.87596200	3.11404800	7.82033900
C	13.16142700	3.06908600	6.52509200
H	12.52537500	2.51481000	7.20525300
C	10.04136900	5.12306300	6.44553100
C	7.87793300	4.70341100	5.74054400
H	7.08052600	5.44781200	5.69418800
H	7.45562900	3.72177500	5.96807200
C	8.72031200	4.65818600	4.43678100
H	8.55842600	3.69898300	3.92962400
C	8.67836500	5.69326900	2.04211900
C	8.12392200	6.69712600	1.18814400
H	7.41504200	7.40762100	1.60448800
C	8.50555600	6.77596500	-0.13874600
H	8.09530100	7.55021800	-0.77823000
C	9.43530500	5.85849500	-0.64843300
H	9.74542300	5.93193600	-1.68639100
C	9.96812600	4.85112400	0.16418100
H	10.68514500	4.14655000	-0.24340800
C	9.60731300	4.76338700	1.49817100
H	10.04343300	3.99542800	2.12776100

C	8.34653600	5.74283000	3.44401800
H	7.45213500	6.30629200	3.68933800
Cu	9.86982800	7.12684700	3.27872500
Br	8.95137000	8.21144200	5.11861500
Br	11.97690800	7.46246200	2.33636400
C	10.16473400	4.72335700	4.97310100
H	10.82995600	5.41499600	4.44969200

INT4

S	11.17848400	3.25306200	4.67767900
O	11.26309800	2.99125100	3.22522800
O	10.62459200	2.18994000	5.55105700
O	8.48052100	4.85089600	6.63340200
O	10.60891000	5.24490900	7.24346200
C	12.82647900	3.61121500	5.26442700
C	13.82950600	3.83913400	4.32549200
H	13.58020200	3.86681800	3.27324000
C	15.13497700	4.03289400	4.77424600
H	15.92678600	4.21926200	4.05483600
C	15.41452000	4.01569600	6.14084100
H	16.43181200	4.18122200	6.48648600
C	14.39449200	3.78963300	7.06901000
H	14.61498600	3.77892600	8.13255000
C	13.08843100	3.57662200	6.63432100
H	12.28608600	3.39706900	7.33718800
C	9.82235900	4.95537600	6.37556000
C	7.80486400	4.19948200	5.54361100
H	6.82512800	4.67050900	5.42814200
H	7.68268500	3.14165200	5.79911200
C	8.70381800	4.37328200	4.31743200
H	8.65910700	3.50997700	3.65108800
C	8.68335000	5.79393300	2.14944100
C	8.09866800	6.89346100	1.48427900
H	7.61332800	7.66290600	2.07491200
C	8.17072500	7.00551400	0.10194300
H	7.71045800	7.85652500	-0.39281000
C	8.84985400	6.03918000	-0.64751400
H	8.91702900	6.13488900	-1.72823100
C	9.45700700	4.96194200	0.00103600
H	10.00614100	4.21956900	-0.57177900
C	9.37981100	4.83350000	1.38525500
H	9.89564400	4.01721900	1.87858800
C	8.54496000	5.69043000	3.60353200
H	7.86267100	6.40147200	4.06107700

Cu	10.35774000	6.47778000	4.10482900
Br	9.43092200	8.74303300	4.28395800
Br	12.68802500	6.90682000	3.69987500
C	10.08645700	4.66026900	4.92016700

TS2

S	11.04401900	3.10187900	4.70152100
O	10.99479300	2.91221800	3.23685300
O	10.67119200	1.97482900	5.57879500
O	8.36926600	4.62603300	6.81744200
O	10.52509100	4.70995200	7.44986300
C	12.69390100	3.62727300	5.13535300
C	13.49208000	4.21138700	4.15265300
H	13.10679000	4.34801200	3.15015200
C	14.77702500	4.62902400	4.49635500
H	15.40129400	5.10600300	3.74772000
C	15.24151500	4.45816800	5.80067200
H	16.24022000	4.79473700	6.06590000
C	14.42799600	3.86726900	6.77192000
H	14.79224200	3.74273700	7.78749800
C	13.14135100	3.44620500	6.44523300
H	12.48619000	3.00052600	7.18213100
C	9.69824600	4.63877600	6.57302300
C	7.58534300	4.53397100	5.61661000
H	7.02547500	5.46665700	5.52017000
H	6.90735300	3.68471400	5.73433900
C	8.56936000	4.32941400	4.43715200
H	8.40358700	3.39885500	3.89229400
C	9.00420200	5.57459900	2.16286600
C	8.98990600	6.85141000	1.55431200
H	8.87864000	7.72824400	2.18616400
C	9.12732900	6.97295000	0.17852800
H	9.11764600	7.95726300	-0.27977400
C	9.29252000	5.82909000	-0.60949400
H	9.40996200	5.92489500	-1.68588200
C	9.31601700	4.56263500	-0.01679300
H	9.45571700	3.67741400	-0.63095500
C	9.17520300	4.42913900	1.35996100
H	9.24205400	3.45331200	1.82253400
C	8.78619400	5.53753100	3.59451200
H	8.44448900	6.47503300	4.06071600
Cu	10.66019100	6.15865000	4.33487000
Br	8.17492000	8.25094400	5.49561400
Br	12.32251200	7.52104000	3.43576900

C	9.94280400	4.47447900	5.09037000

INT5

S	11.07689100	2.81016400	4.98590800
O	10.95496000	2.54942300	3.53874300
O	10.82710300	1.74011500	5.95768100
O	8.09856800	4.60636700	6.69281800
O	10.04271400	4.24857200	7.78336000
C	12.67907300	3.54493200	5.28666200
C	13.47652300	3.89546200	4.19337900
H	13.13727600	3.67631000	3.18749000
C	14.69135500	4.53375800	4.43497300
H	15.32097100	4.82568400	3.60065600
C	15.08828500	4.81072400	5.74463900
H	16.02780200	5.32395000	5.92446400
C	14.28344100	4.44145300	6.82557400
H	14.59703800	4.66440300	7.84023300
C	13.06835500	3.79628200	6.60773100
H	12.41736700	3.52102600	7.42964000
C	9.43289000	4.32324600	6.74749000
C	7.56783600	4.39477000	5.36942100
H	6.85806900	5.19860900	5.16179800
H	7.04325900	3.43437800	5.35947400
C	8.76877400	4.38220900	4.41462500
H	8.67610000	3.65753600	3.60769100
C	9.80871300	6.02683200	2.66705100
C	9.97852900	7.38274100	2.30872200
H	9.77068200	8.15515700	3.04255200
C	10.43272600	7.72471400	1.04360500
H	10.55476300	8.76993900	0.77822700
C	10.75134200	6.72092500	0.12224800
H	11.11902200	6.98826700	-0.86401500
C	10.60832000	5.37505000	0.47246300
H	10.86595700	4.59709500	-0.23962500
C	10.14058600	5.02197000	1.73336400
H	10.07233400	3.97726800	2.01033600
C	9.95634500	4.18129000	5.34154200
C	9.27475100	5.72668500	3.98747400
H	8.87037100	6.57373900	4.54455700
Cu	11.04755000	5.80961200	5.10076100
Br	12.34259100	7.70116000	5.20848900

TS3

S	8.89088200	2.02344800	4.36735900

O	9.28925000	2.02902800	2.95203700
O	7.90281200	1.06446800	4.86682300
O	6.19158600	4.59499800	5.46754800
O	7.30476400	3.31045600	6.95562400
C	10.36094800	1.96662500	5.37780100
C	11.58949800	2.32608500	4.80571000
H	11.64037200	2.60580100	3.75957400
C	12.72267800	2.31844900	5.61589200
H	13.68155100	2.60958500	5.19993900
C	12.62078800	1.95351200	6.96034700
H	13.50545000	1.96838400	7.58882400
C	11.39142000	1.57871600	7.50757200
H	11.32174000	1.30308700	8.55446600
C	10.24373700	1.57688400	6.71828500
H	9.27467800	1.30853400	7.12236000
C	7.22483300	3.80142700	5.86194700
C	6.20653100	4.83666700	4.04663100
H	5.98879500	5.89504000	3.88581200
H	5.42492500	4.22633700	3.58518900
C	7.59106300	4.42514400	3.55919500
H	7.66800500	3.97386500	2.57529100
C	9.99845400	5.43651500	3.27657500
C	10.88091900	6.42991700	3.73989000
H	10.65161500	6.97196900	4.65203800
C	12.06307400	6.69887000	3.05716800
H	12.73503600	7.46348100	3.43380100
C	12.38366900	5.97965800	1.90411800
H	13.30953100	6.18340200	1.37433500
C	11.50785600	4.99988700	1.43205300
H	11.74711900	4.44169700	0.53188900
C	10.31908300	4.72944100	2.10724000
H	9.66641500	3.94941200	1.73976100
C	8.21372600	3.68240100	4.71336000
C	8.74293000	5.21728500	4.04294700
H	8.47695900	6.03204300	4.71705700
Cu	9.86246500	4.47043200	5.84547600
Br	11.16058700	5.52472100	7.41189000

2aa

S	9.56642300	1.41316300	5.07323800
O	10.82935500	1.63055000	4.34768900
O	8.69335700	0.28654600	4.72918800
O	6.56644500	3.71226100	4.02883700
O	6.40650400	2.19451400	5.68891100

C	9.92042100	1.39879400	6.82440700
C	11.24372900	1.51078200	7.24300600
H	12.03287300	1.60946400	6.50751600
C	11.51124000	1.52416000	8.61098300
H	12.53600600	1.61756800	8.95685800
C	10.46397500	1.43552900	9.52909400
H	10.67681600	1.45363900	10.59422200
C	9.14170200	1.32723300	9.08830800
H	8.33101100	1.25861000	9.80744000
C	8.85711000	1.30165200	7.72448200
H	7.83775200	1.22730600	7.35891000
C	8.59069900	2.91150200	4.85428900
C	7.09145600	2.83787600	4.93924800
C	7.57527600	4.24466300	3.14218400
H	7.40079900	5.32047100	3.05606600
H	7.45297400	3.77523100	2.16120100
C	8.91731200	3.89526800	3.76254000
H	9.76988400	3.70776700	3.12055400
C	9.12563300	4.32293900	5.17930000
H	8.34614600	4.96054500	5.59468000
C	10.44902100	4.53262700	5.83645200
C	10.45005100	4.83326900	7.20530500
H	9.50398800	4.89825000	7.73623700
C	11.64551000	5.01878000	7.89325700
H	11.62704000	5.24076400	8.95610600
C	12.86187300	4.90966500	7.21737200
H	13.79769100	5.05228400	7.75014400
C	12.86966800	4.61833200	5.85301800
H	13.81140000	4.53371000	5.31863200
C	11.67112100	4.43250800	5.16468200
H	11.69463900	4.18897700	4.10944400

t-BuO·

C	-0.65614700	0.02845600	0.02288700
C	-0.15703800	-1.42775100	-0.00530000
H	0.93772100	-1.44929000	-0.02306200
H	-0.49553800	-1.97749000	0.88002800
H	-0.52537500	-1.93614000	-0.90129000
C	-0.15687700	0.78106600	1.26981200
H	0.93789200	0.80675200	1.27978800
H	-0.52479500	1.81135800	1.26176800
H	-0.49578100	0.28959300	2.18862100
C	-2.21446800	0.05611100	-0.02469700
H	-2.57863200	1.08653200	-0.02750700

H	-2.57883700	-0.45649700	-0.91849300
H	-2.59396200	-0.45751500	0.86490900
O	-0.29730600	0.69499800	-1.13178400

t-BuOH

C	-0.67939300	0.02425800	0.03019200
C	-0.15619400	-1.41761000	0.00129100
H	0.94167500	-1.43234000	-0.00036400
H	-0.49397900	-1.98392700	0.87663000
H	-0.50602100	-1.92841900	-0.90146600
C	-0.15601500	0.77032000	1.26431000
H	0.94185900	0.77876100	1.27637000
H	-0.50550700	1.80765000	1.25503300
H	-0.49407800	0.29575000	2.19249900
C	-2.20699600	0.04945100	-0.01316700
H	-2.56746900	1.08290800	-0.02938100
H	-2.56761800	-0.45289300	-0.91641000
H	-2.62986600	-0.45567900	0.86149100
O	-0.26477600	0.71230400	-1.16177300
H	0.70578300	0.71587200	-1.16749100

t-BuO-

C	-0.64984100	0.07869500	-0.06414200
C	-0.16141800	-1.41977400	0.00310300
H	0.93747300	-1.42875700	-0.01601500
H	-0.49946800	-1.99326100	0.88620000
H	-0.50796000	-1.93966800	-0.90112400
C	-0.16121400	0.76985300	1.26704900
H	0.93768200	0.79070000	1.26527900
H	-0.50757700	1.81294300	1.26501800
H	-0.49937900	0.29201700	2.20531500
C	-2.22546900	0.04027500	0.00273700
H	-2.59992300	1.07343800	-0.01633900
H	-2.60006900	-0.45953200	-0.90162800
H	-2.65381500	-0.46957200	0.88569200
O	-0.20816400	0.70282400	-1.14546400

Br-

Br	1.60054800	0.71656300	-1.16820300

CuBr

Cu	-0.88627600	0.10026700	0.00000000
Br	-3.12400200	0.10026700	0.00000000

$CuBr_2$

Cu	-0.85013900	0.10026700	0.00000000
Br	1.39084700	0.10026700	0.00000000
Br	-3.09112500	0.10026700	0.00000000

11. References

[1] (a) M. Ma, W. Hao, L. Ma, Y. Zheng, P. Lian and X. Wan, *Org. Lett.*, 2018, **20**, 5799−5802. (b) F. Li, S. Zhu and R. M. Koenigs, *Chem. Commun.*, 2022, **58***,* 7526−7529.

[2] C. Wang, R.-H. Liu, M.-Q. Tian, X.-H. Hu and T.-P. Loh, *Org. Lett.*, 2018, **20**, 4032−4035.

[3] V. K. Vyas, G. J. Clarkson and M. Wills, *Angew. Chem. Int. Ed.*, 2020, **59**, 14265−14269.

[4] M. G. Johnson, M. W. Gribble, J. B. Houze and N. A. Paras, *Org. Lett.*, 2014, **16**, 6248−6251.

[5] S. Pape, L. Daukšaitė, S. Lucks, X. Gu and H. Brunner, *Org. Lett.*, 2016, **18**, 6376−6379.

[6] C. Cruché, W. Neiderer and S. K. Collins, *ACS Catal.*, 2021, **11**, 8829−8836.

[7] (a) J. Mao, F. Liu, M. Wang, L. Wu, B. Zheng, S. Liu, J. Zhong, Q. Bian and P. J. Walsh, *J. Am. Chem. Soc.*, 2014, **136**, 17662−17668. (b) J. H. Reed, P. A. Donets, S. Miaskiewicz and N. Cramer, *Angew. Chem. Int. Ed.*, 2019, **58**, 8893−8897.

[8] S. Gandomkar, E. Jost, D. Loidolt, A. Swoboda, M. Pickl, W. Elaily, B. Daniel, M. W. Fraaije, P. Macheroux and W. Kroutil, *Adv. Synth. Catal.*, 2019, **361**, 5264−5271.

[9] X. Ren, A. L. Chandgude and R. Fasan, *ACS Catal.*, 2020, **10**, 2308−2313.

[10] M. Ito, A. Osaku, A. Shiibashi and T. Ikariya, *Org. Lett.*, 2007, **9**, 1821−1824.

[11] X. Xu, H. Lu, J. V. Ruppel, X. Cui, S. L. Mesa, L. Wojtas and X. P. Zhang, *J. Am. Chem. Soc.*, 2011, **133**, 15292−15295.

[12] T. Niwa, H. Yorimitsu and H. Yorimitsu, *Tetrahedron Lett.*, 2009, **65**, 1971−1976.

[13 (a) L. Tóke, G. T. Szabó, Z. Hell and G. Tóth, *Tetrahedron Lett.*, 1990, **31**, 7501-7504. (b) N. A. Swain, R. C. D. Brown and G. Bruton, *J. Org. Chem.*, 2004, **69**, 122-129.

[14] K. Singh, S. J. Staig, and J. D. Weaver, *J. Am. Chem. Soc.,* 2014, **136**, 5275–5278.

[15] F. Benedetti, F. Berti, A. Risaliti, *Tetrahedron Lett.,* 1993, **34,** 6443-6446.

[16] X. Xu, X. P. Zhang, *J. Am. Chem. Soc.* 2011, **133,** 15292–15295.

[17] S. Takada, Y. Nishii, *Org. Biomol. Chem.*, 2017, **15,** 2443–2449.

[18] S. Takada, Y. Nishii, *Tetrahedron Lett.,* 2016, **57,** 2422–2425.

[19] M. J. Frisch, G. W. Trucks, H. B. Schlegel, et al. *Gaussian 16, Revision A.03*; Gaussian, Inc.: Wallingford,

CT, 2016.

[20] (a) A. D. Becke, *J. Chem. Phys.,* 1993, **98**, 5648; (b) K. Raghavachari, *Theor. Chem. Acc.,* 2000, **103**, 361; (c) A. D. Becke, *J. Chem. Phys.,* 1993, **98**, 1372; (d) C. Lee; W. Yang; R. G. Parr, *Phys. Rev. B.,* 1988, **37**, 785.

[21] S. Grimme, S. Ehrlich and L. Goerigk, *J. Comput. Chem.,* 2011, **32**, 1456

[22] D. Andrae, U. Häußermann, M. Dolg, H. Stoll and H. Preuß, *Theor. Chim. Acta.,* 1990, **77**, 123.

[23] (a) R. Ditchfield, W. J. Hehre and J. A. Pople, *J. Chem. Phys.,* 1971, **54**, 724; (b) W. J. Hehre, R. Ditchfield and J. A. Pople, *J. Chem. Phys.,* 1972, **56**, 2257.

[24] F. Weigend and R. Ahlrichs, *Phys. Chem. Chem. Phys.,* 2005, **7**, 3297.

[25] A. V. Marenich, C. J. Cramer and D. G. Truhlar, *J. Phys. Chem., B* 2009, **113**, 6378.

[26] CYLview20; Legault, C. Y., Université de Sherbrooke, 2020 (http://www.cylview.org).

12. NMR Spectra

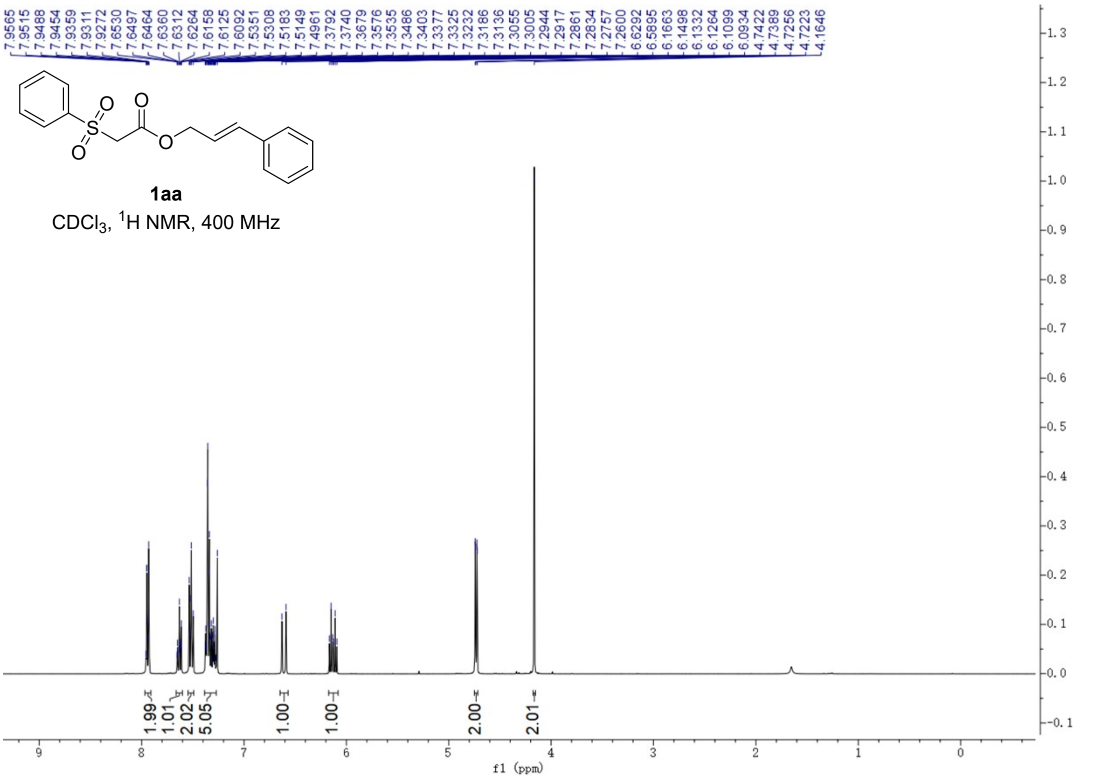

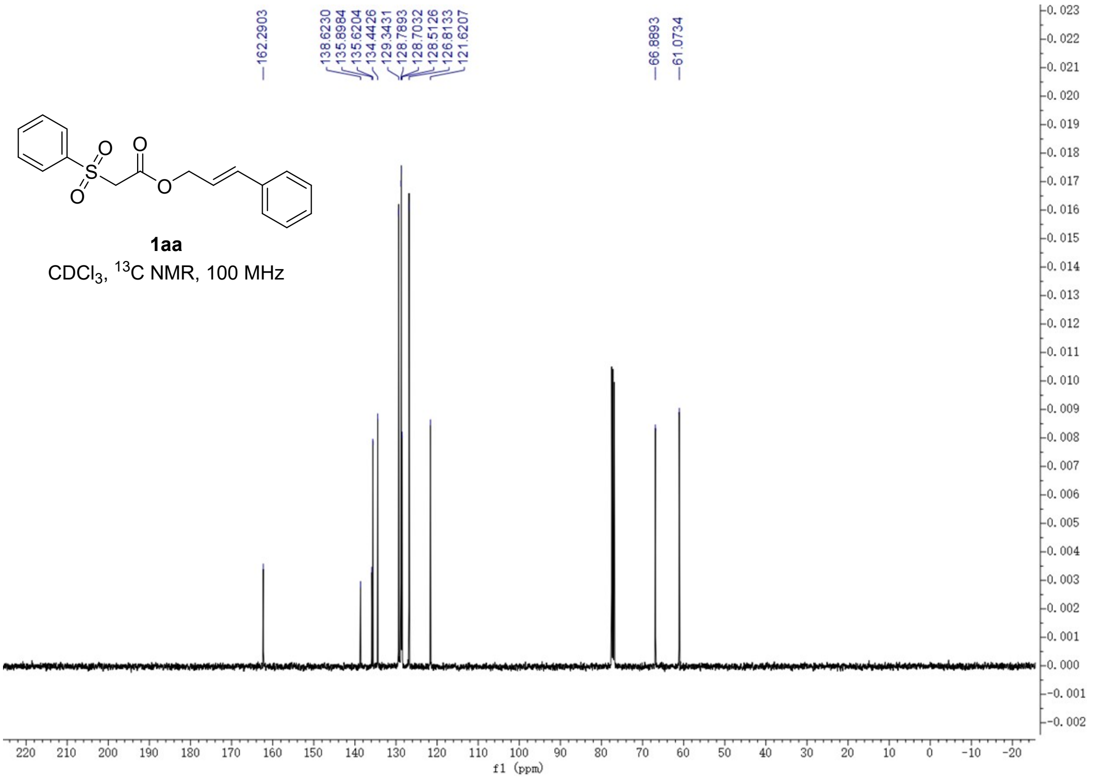

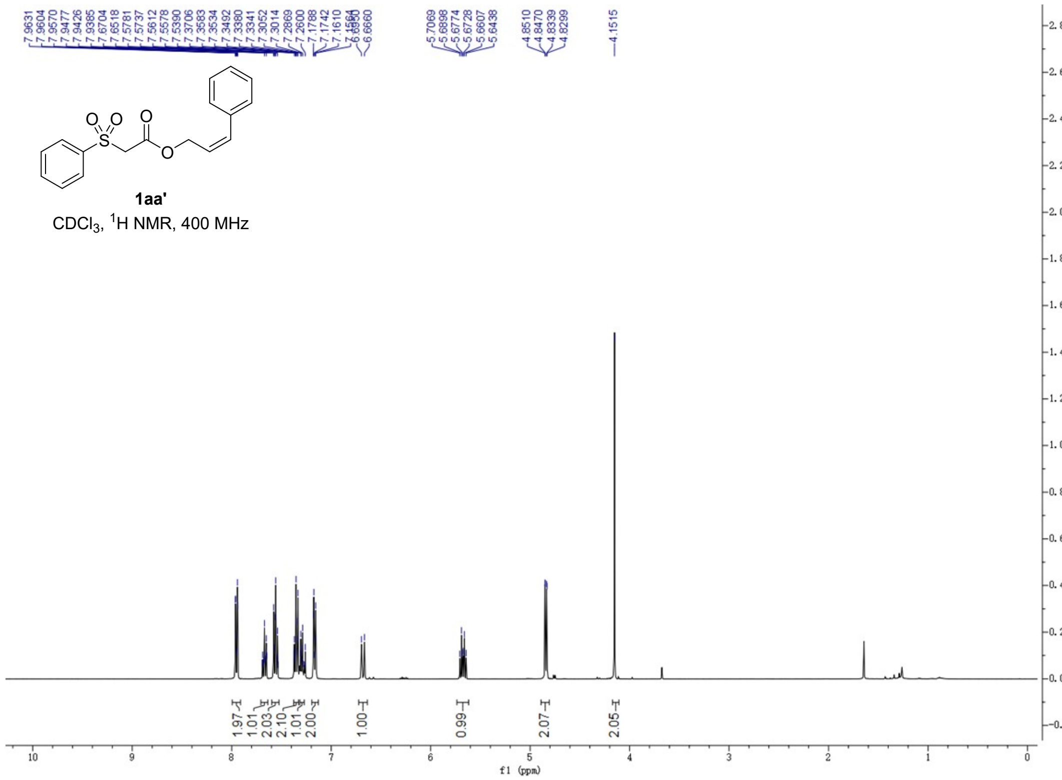
1aa'
CDCl$_3$, ^{1}H NMR, 400 MHz

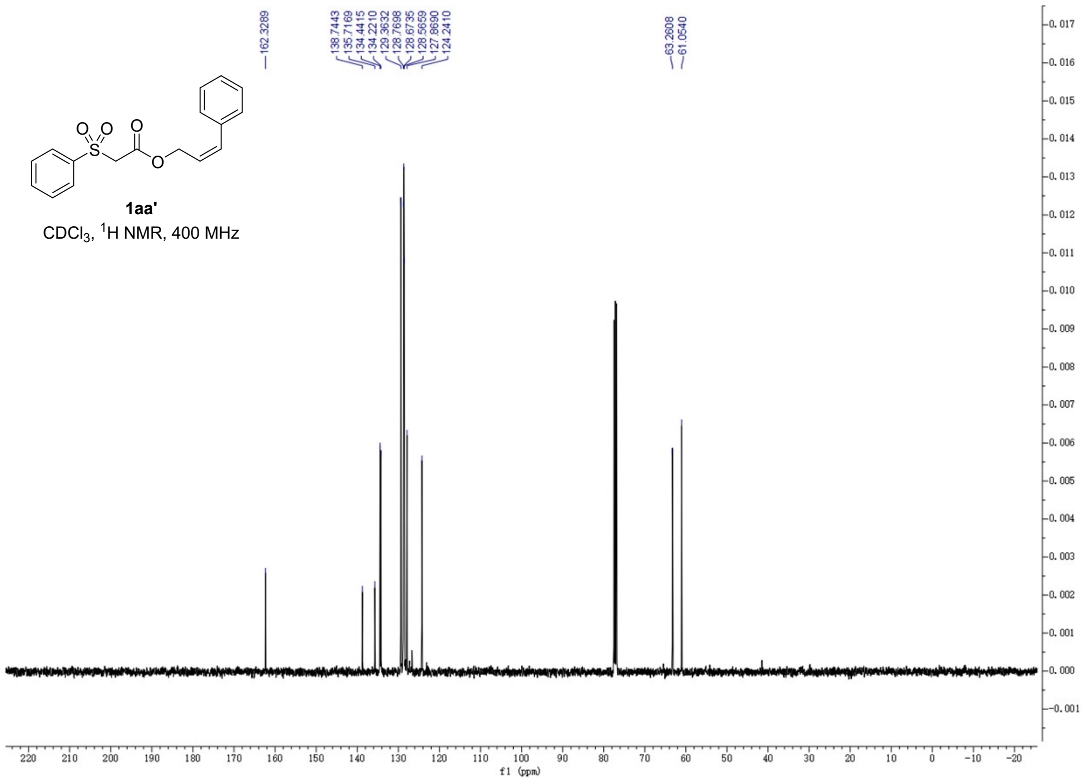
1aa'
CDCl$_3$, ^{1}H NMR, 400 MHz

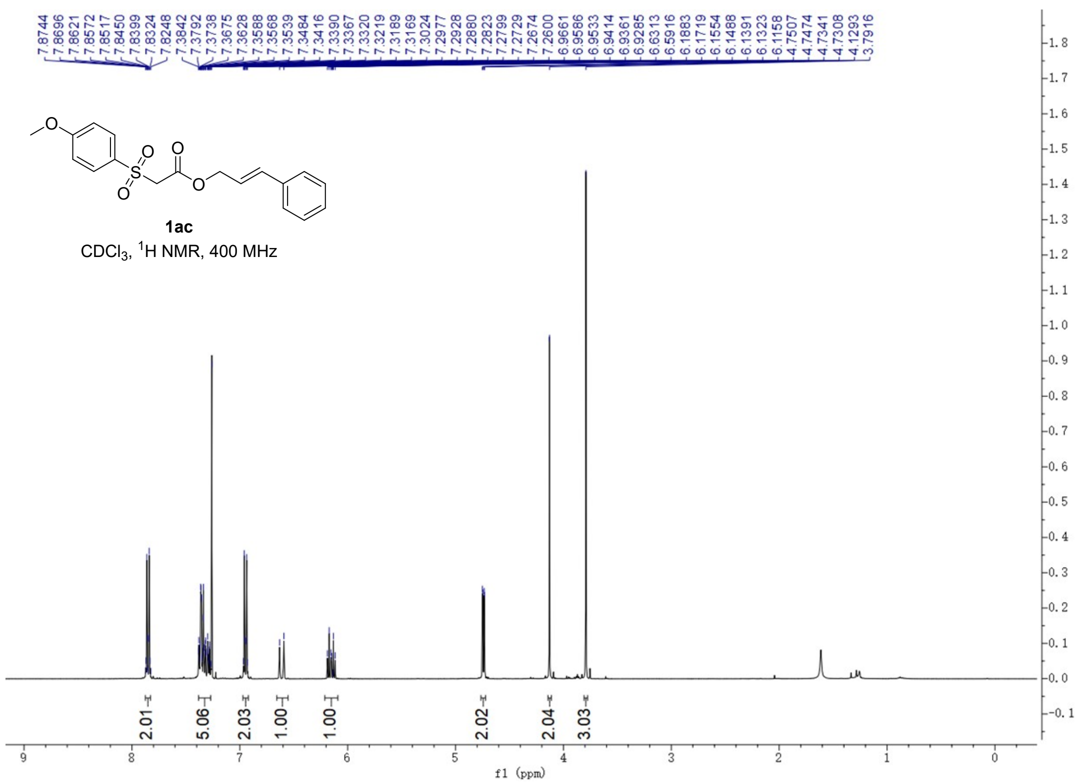
1ac
CDCl3, 1H NMR, 400 MHz

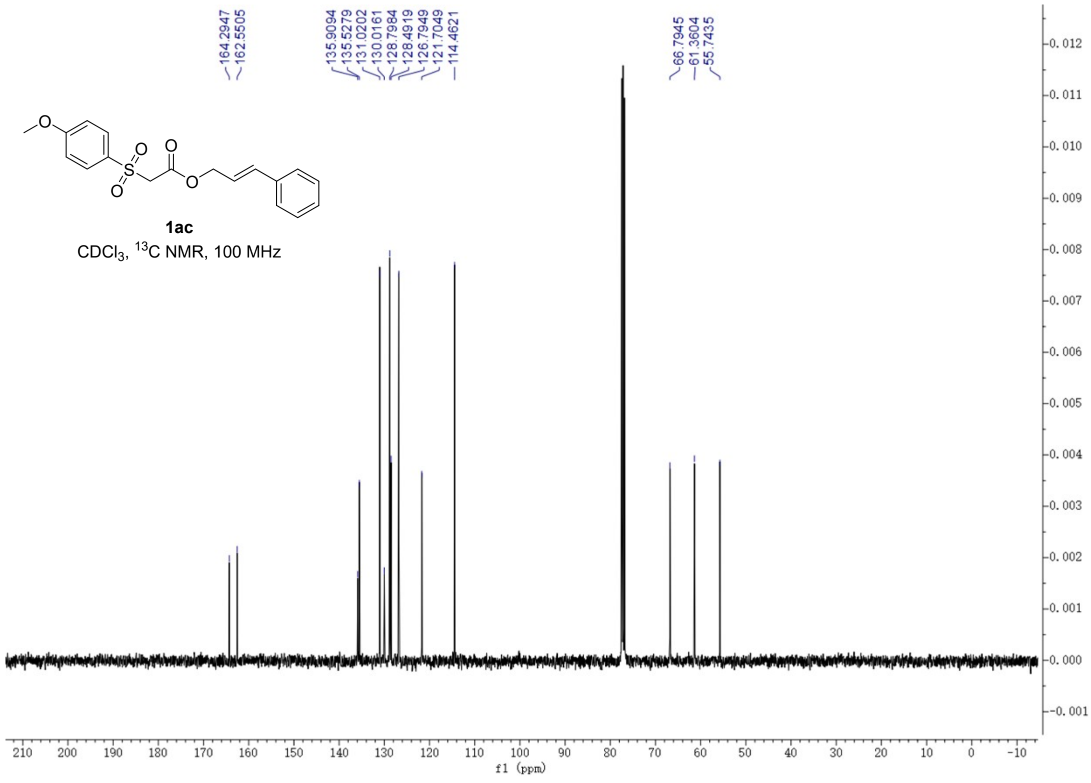
1ac
CDCl3, 13C NMR, 100 MHz

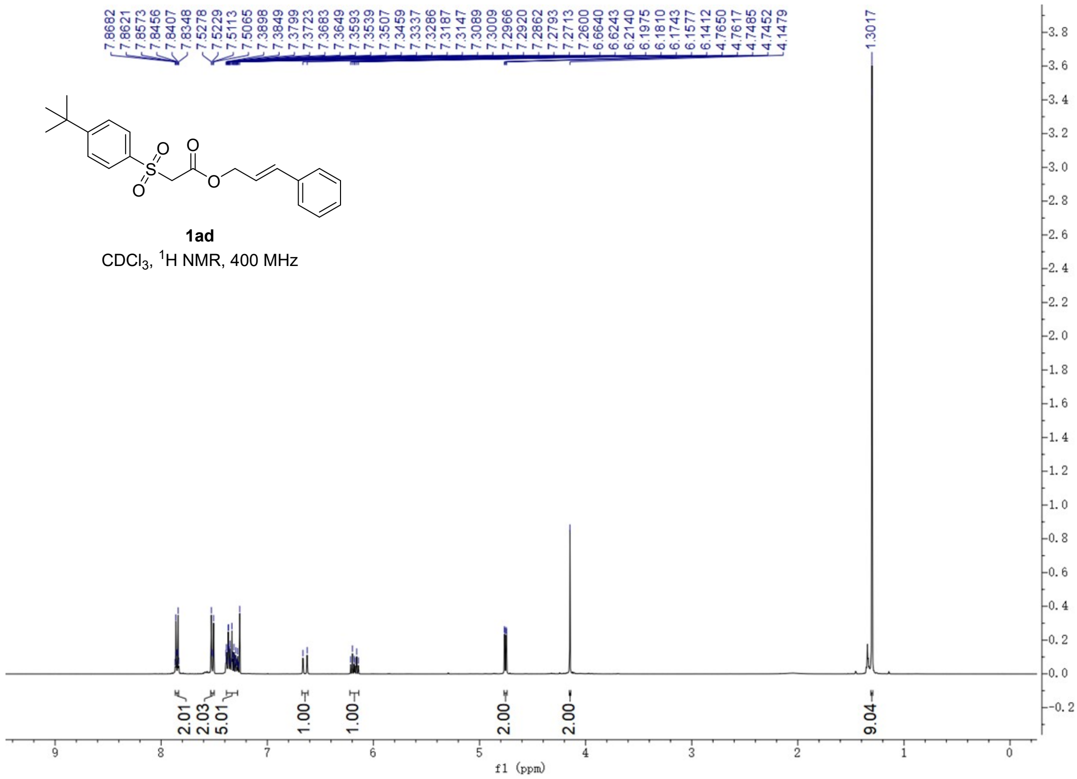

1ad

CDCl3, 1H NMR, 400 MHz

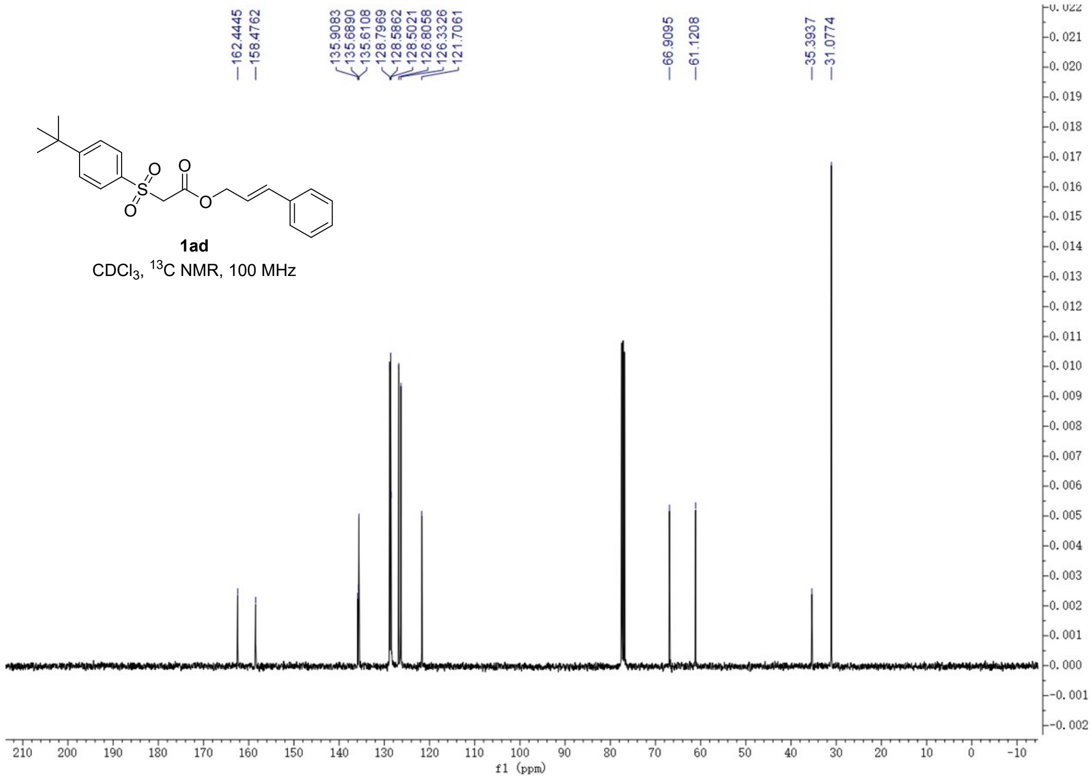

1ad

CDCl3, 13C NMR, 100 MHz

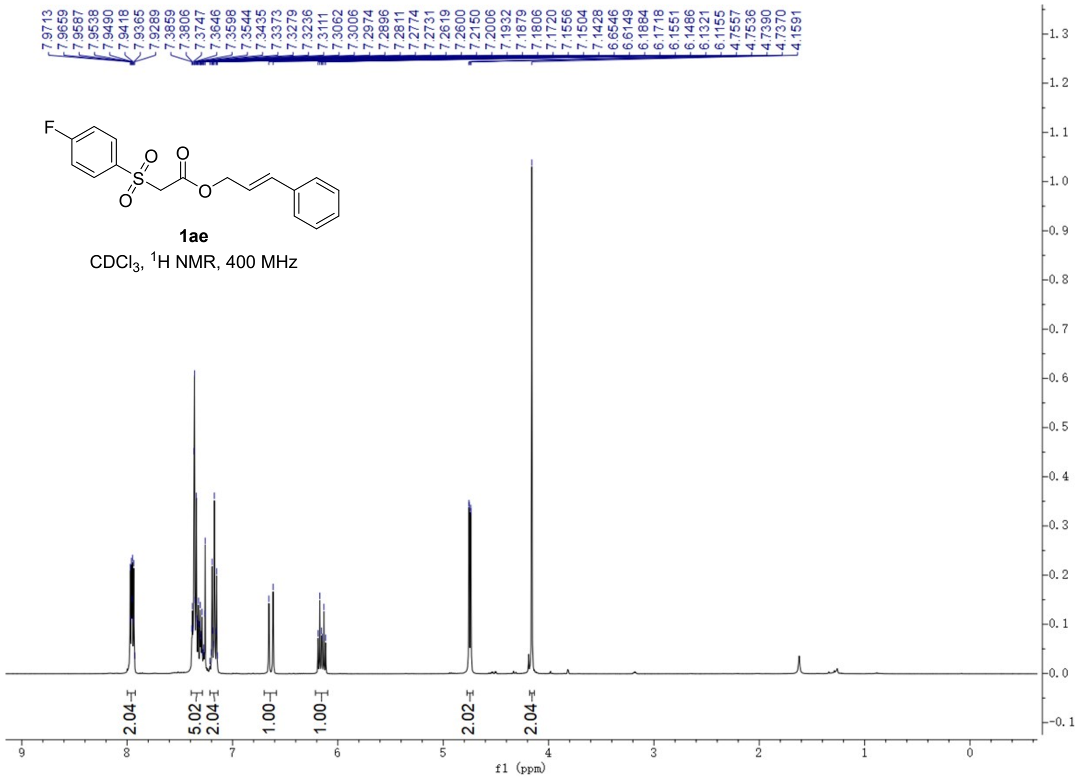
7.9713
7.9659
7.9587
7.9538
7.9490
7.9418
7.9365
7.9289
7.3859
7.3806
7.3747
7.3646
7.3598
7.3544
7.3435
7.3373
7.3279
7.3236
7.3111
7.3062
7.3006
7.2974
7.2896
7.2811
7.2774
7.2731
7.2619
7.2600
7.2150
7.2006
7.1932
7.1879
7.1806
7.1720
7.1556
7.1504
7.1428
6.6546
6.6149
6.1884
6.1718
6.1551
6.1486
6.1321
6.1155
4.7557
4.7536
4.7390
4.7370
4.1591
F
O
S
O
O
O
1ae
CDCl3, 1H NMR, 400 MHz
2.04
5.02
2.04
1.00
1.00
2.02
2.04
f1 (ppm)

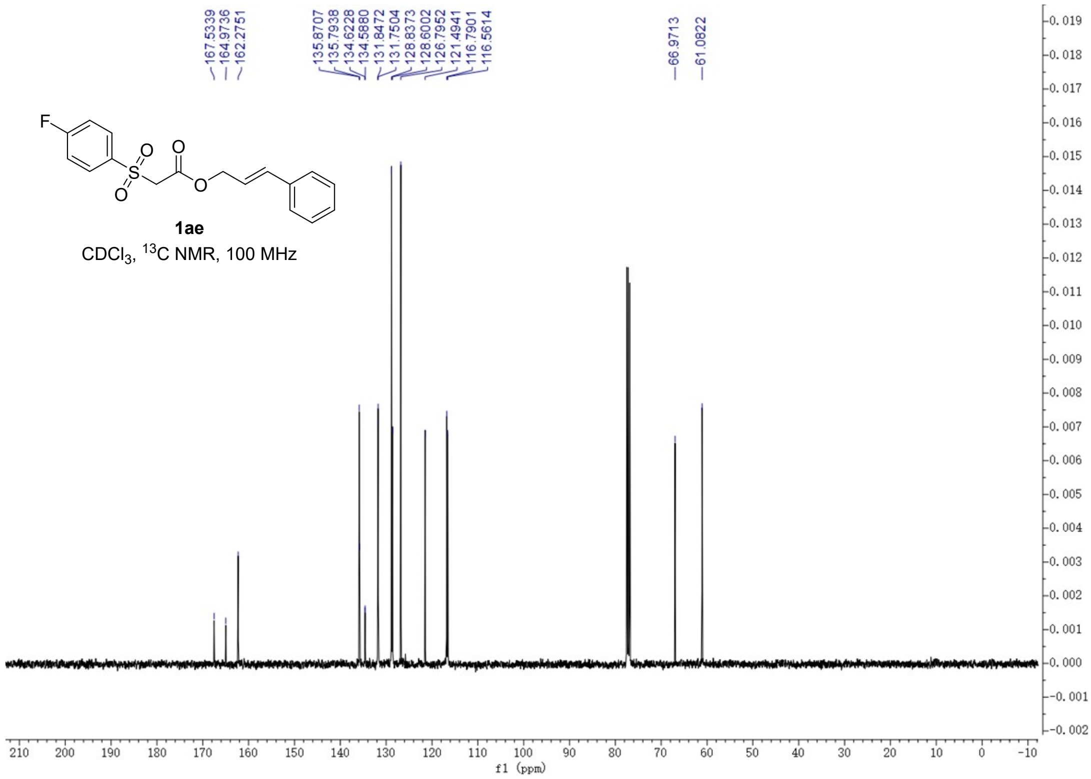
167.5339
164.9736
162.2751
135.8707
135.7938
134.6228
134.5880
131.8472
131.7504
128.8373
128.6002
126.7952
121.4941
116.7901
116.5614
66.9713
61.0822
F
O
S
O
O
O
1ae
CDCl3, 13C NMR, 100 MHz
f1 (ppm)

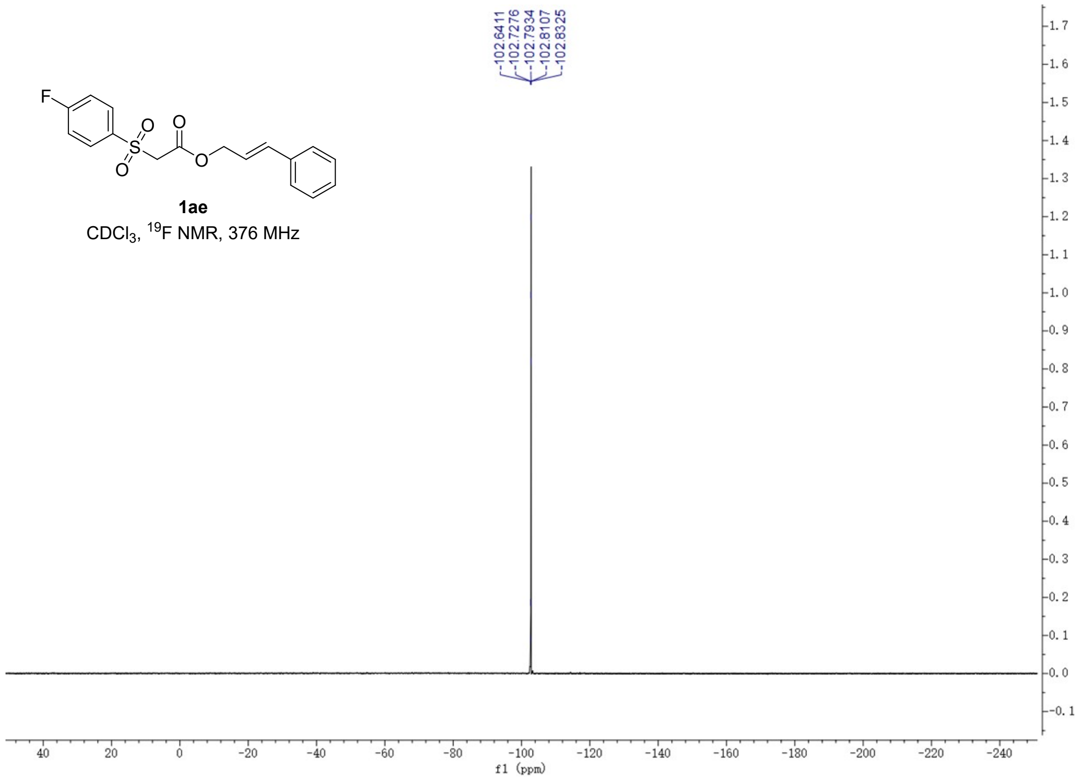

F
S
O
O
O
O
1ae
CDCl3, 19F NMR, 376 MHz
-102.6411
-102.7276
-102.7934
-102.8107
-102.8325
1.7
1.6
1.5
1.4
1.3
1.2
1.1
1.0
0.9
0.8
0.7
0.6
0.5
0.4
0.3
0.2
0.1
0.0
-0.1
40
20
0
-20
-40
-60
-80
-100
-120
-140
-160
-180
-200
-220
-240
f1 (ppm)

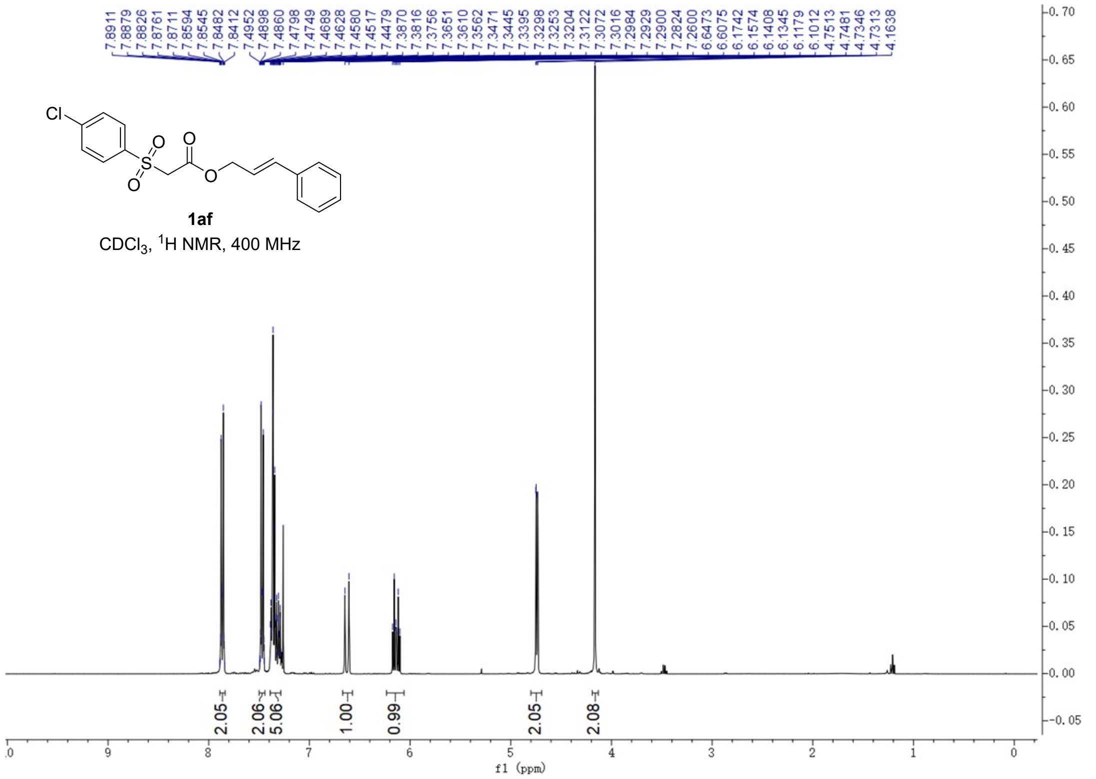

1af
$CDCl_3$, ^1H NMR, 400 MHz

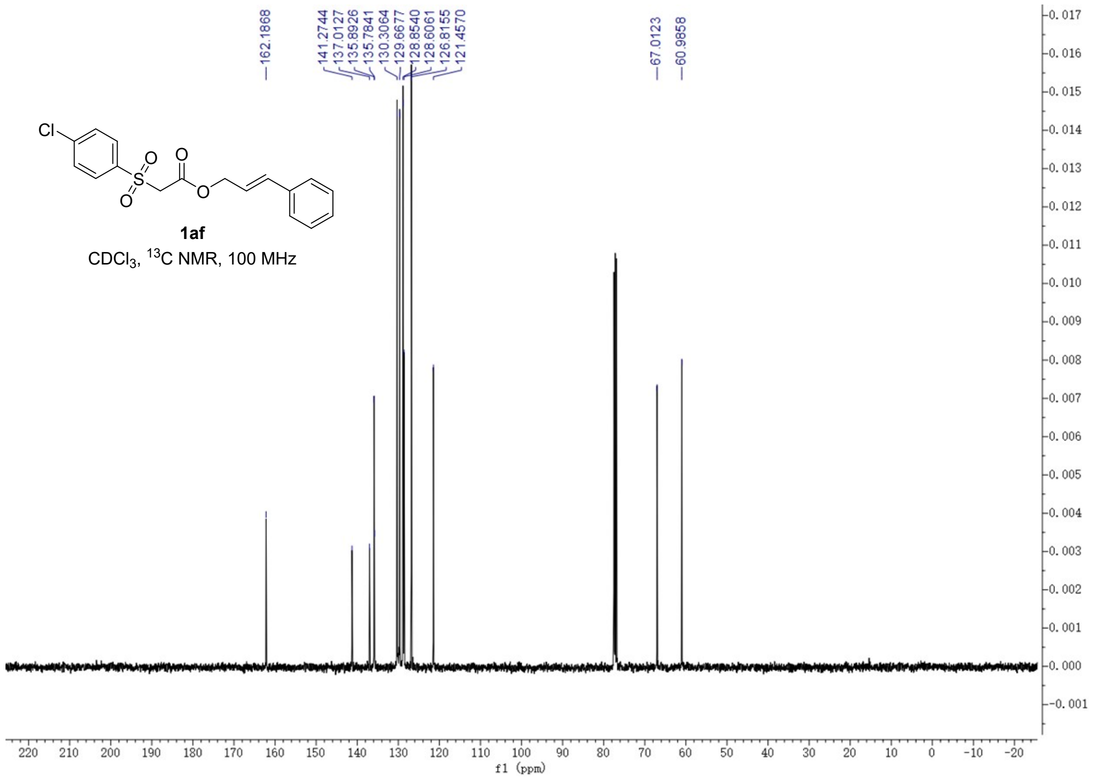

1af
$CDCl_3$, ^{13}C NMR, 100 MHz

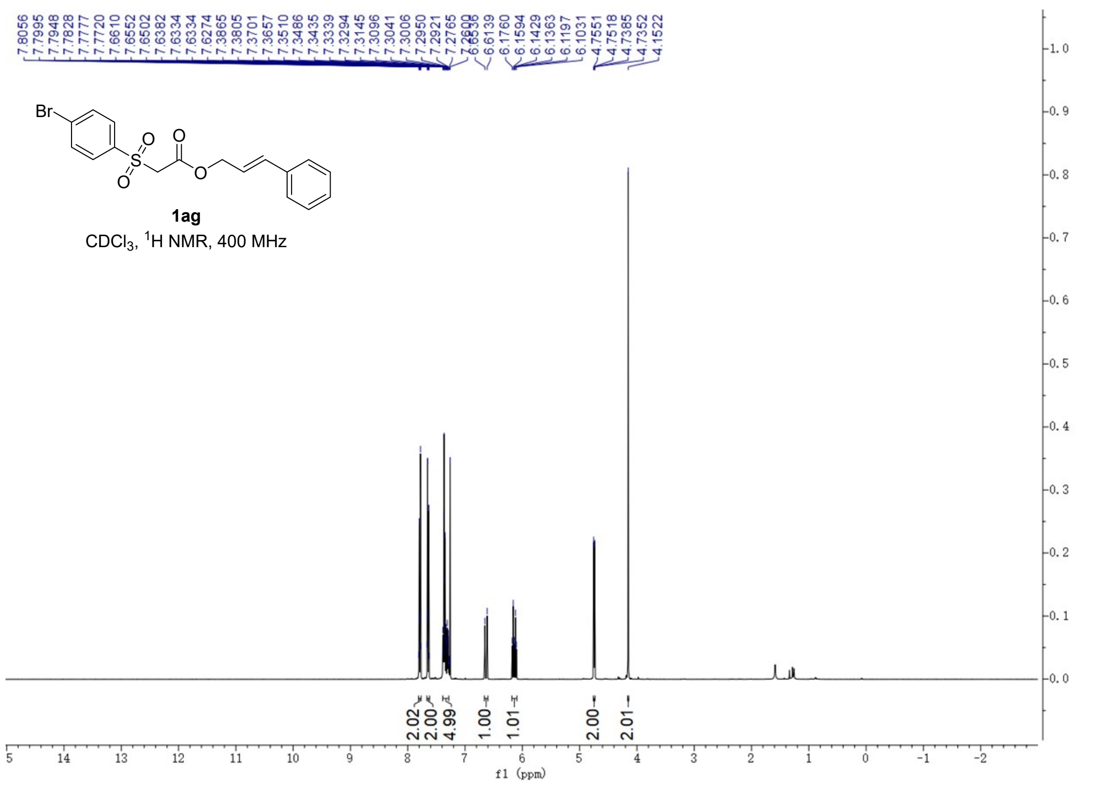
7.8056
7.7995
7.7948
7.7828
7.7777
7.7720
7.6610
7.6552
7.6502
7.6382
7.6334
7.6334
7.6274
7.3865
7.3805
7.3701
7.3657
7.3510
7.3486
7.3435
7.3339
7.3294
7.3145
7.3096
7.3041
7.3006
7.2950
7.2921
7.2765
6.6139
6.1760
6.1594
6.1429
6.1363
6.1197
6.1031
4.7551
4.7518
4.7385
4.7352
4.1522
Br
O
S
O
O
O
1ag
CDCl3, 1H NMR, 400 MHz
2.02
2.00
4.99
1.00
1.01
2.00
2.01
f1 (ppm)

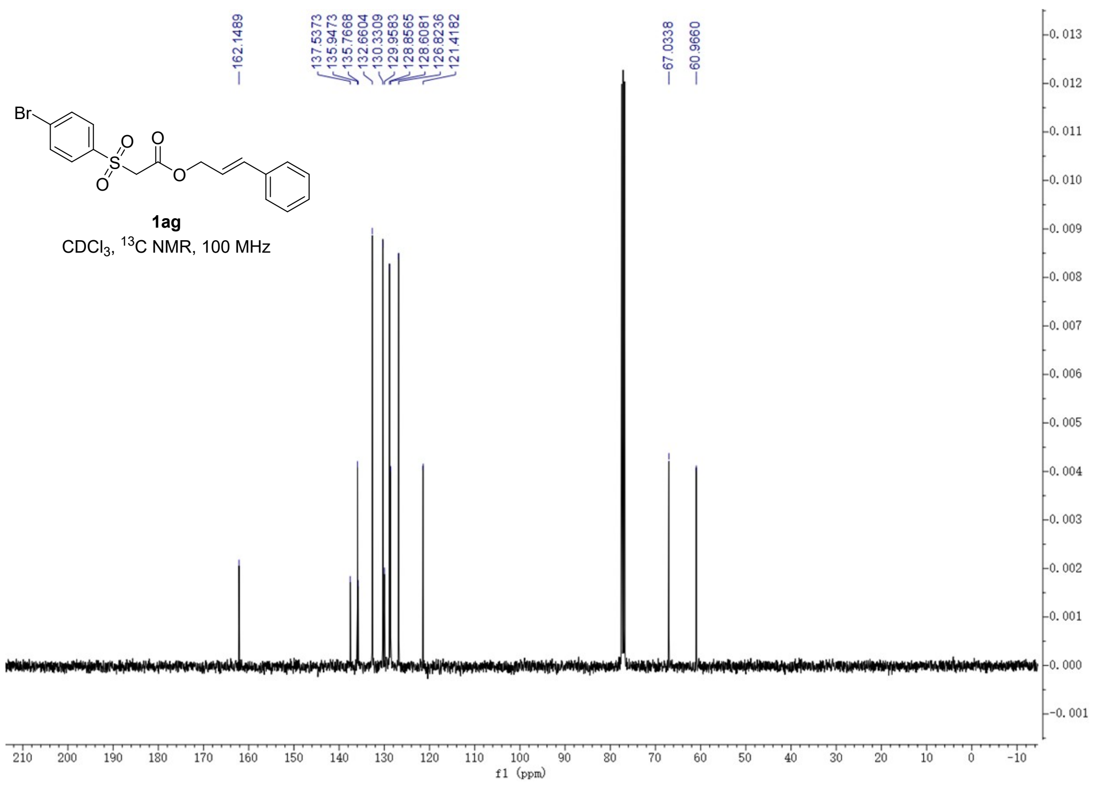
162.1489
137.5373
135.9473
135.7668
132.6604
130.3309
129.9583
128.8565
126.6081
126.8236
121.4182
67.0338
60.9660
Br
O
S
O
O
O
1ag
CDCl3, 13C NMR, 100 MHz
f1 (ppm)

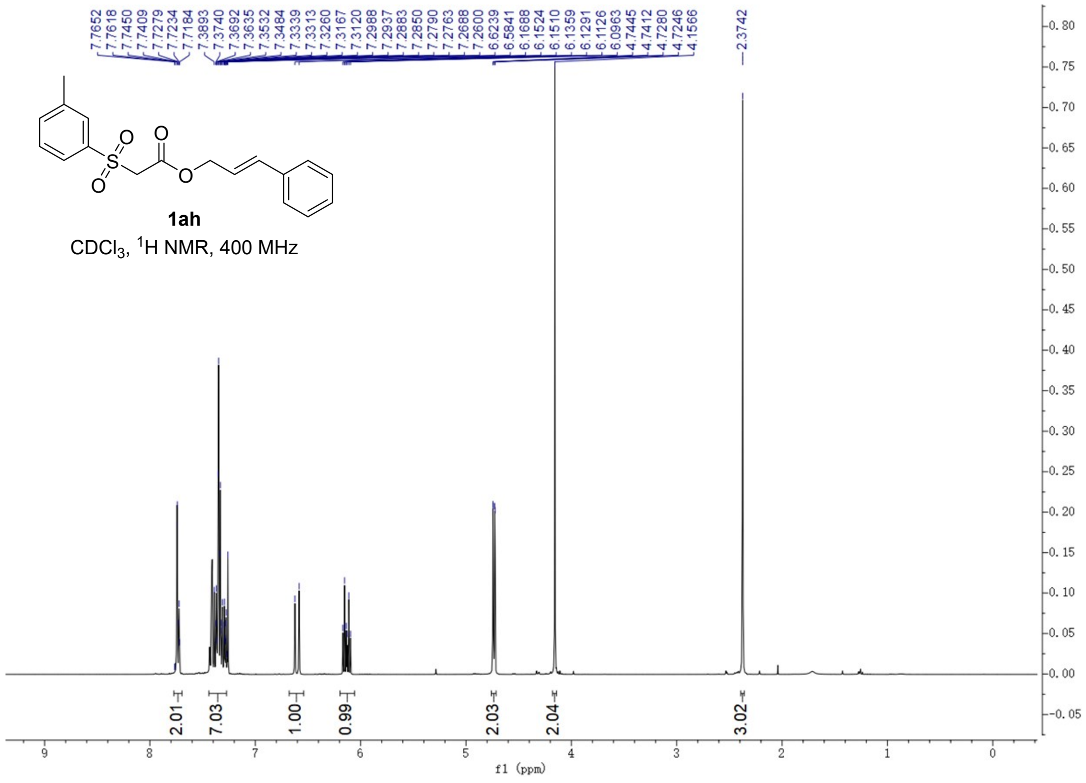
7.7652 7.7618 7.7450 7.7409 7.7279 7.7234 7.7184 7.3893 7.3740 7.3692 7.3635 7.3532 7.3484 7.3339 7.3313 7.3260 7.3167 7.3120 7.2988 7.2937 7.2883 7.2850 7.2790 7.2763 7.2688 7.2600 6.6239 6.5841 6.1688 6.1524 6.1510 6.1359 6.1291 6.1126 6.0963 4.7445 4.7412 4.7280 4.7246 4.1566
2.3742
1ah
CDCl3, 1H NMR, 400 MHz
2.01 7.03 1.00 0.99 2.03 2.04 3.02
f1 (ppm)

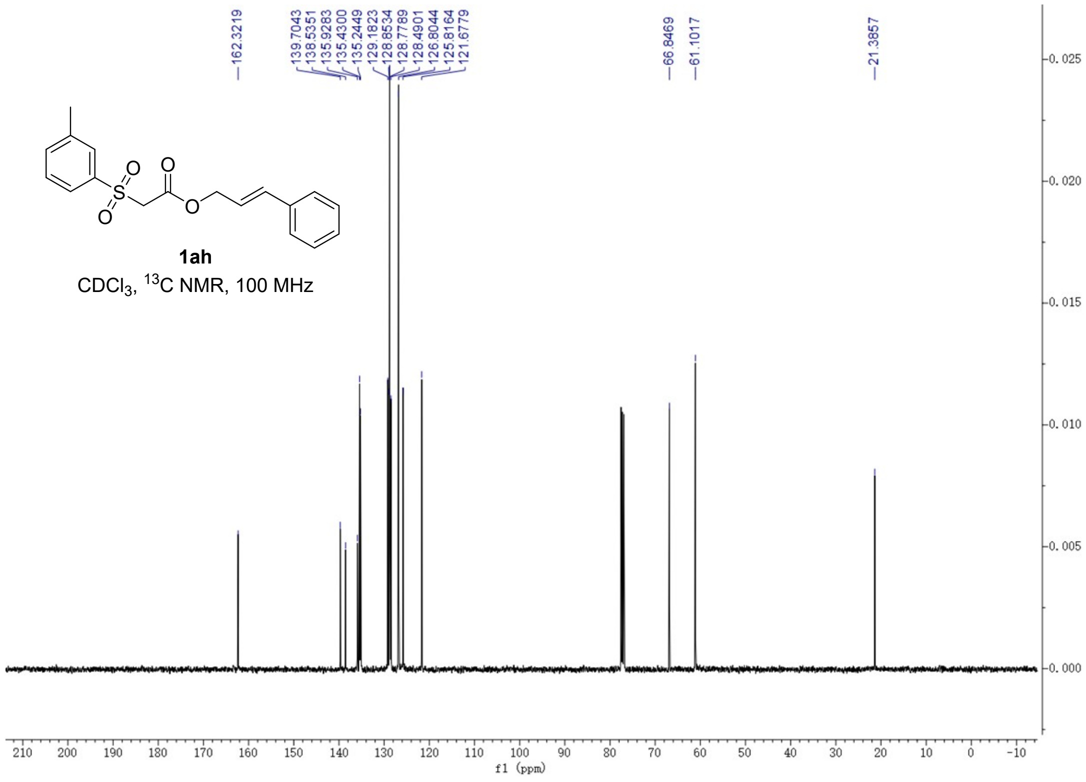
162.3219
139.7043 138.5351 135.9283 135.4300 135.2449 129.1823 128.8534 128.7789 128.4901 126.8044 125.8164 121.6779
66.8469
61.1017
21.3857
1ah
CDCl3, 13C NMR, 100 MHz
f1 (ppm)

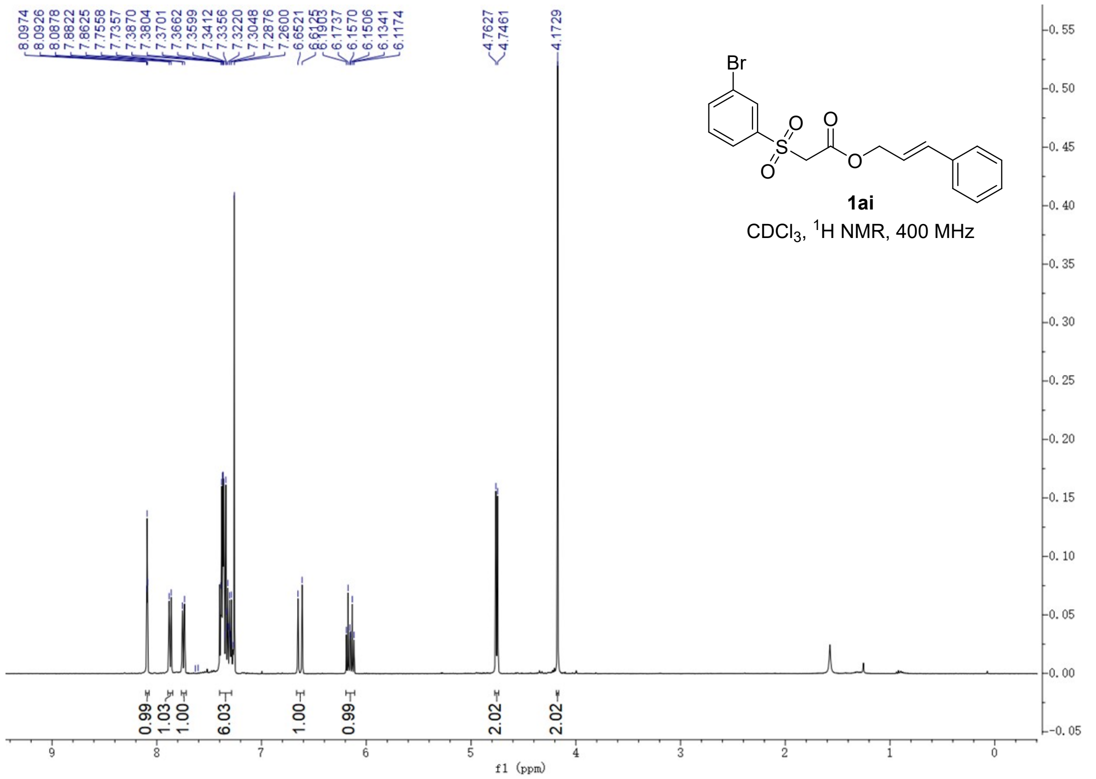
Br
1ai
CDCl3, 1H NMR, 400 MHz
f1 (ppm)

0

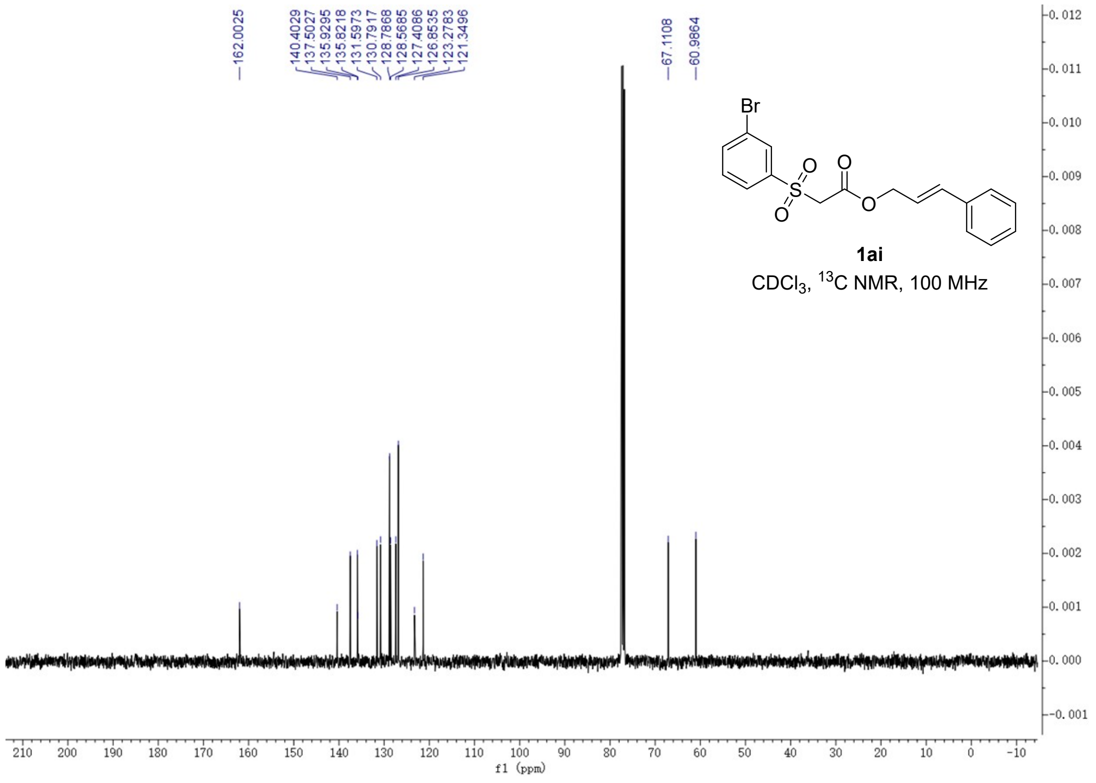
Br
1ai
CDCl3, 13C NMR, 100 MHz
f1 (ppm)

0

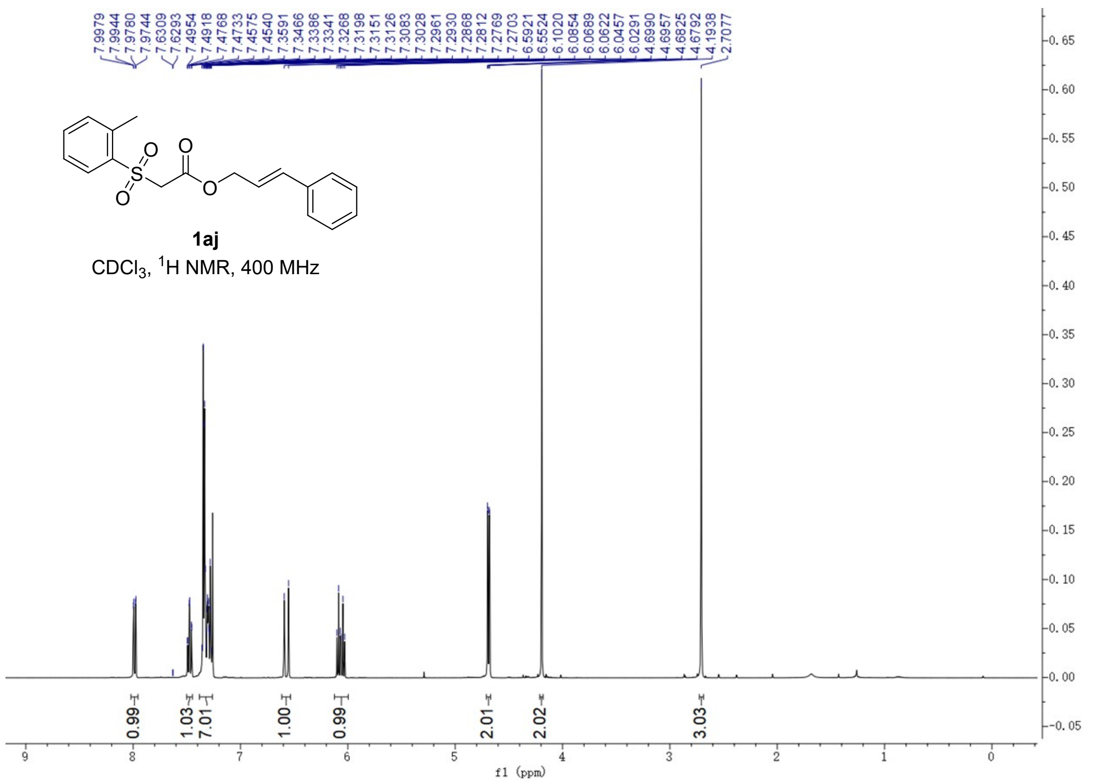
7.9979
7.9944
7.9780
7.9744
7.6309
7.6293
7.4954
7.4918
7.4768
7.4733
7.4575
7.4540
7.3591
7.3466
7.3386
7.3341
7.3268
7.3198
7.3151
7.3126
7.3083
7.3028
7.2961
7.2930
7.2868
7.2812
7.2769
7.2703
6.5921
6.5524
6.1020
6.0854
6.0689
6.0622
6.0457
6.0291
4.6990
4.6957
4.6825
4.6792
4.1938
2.7077
1aj
CDCl3, 1H NMR, 400 MHz
0.99
1.03
7.01
1.00
0.99
2.01
2.02
3.03
f1 (ppm)

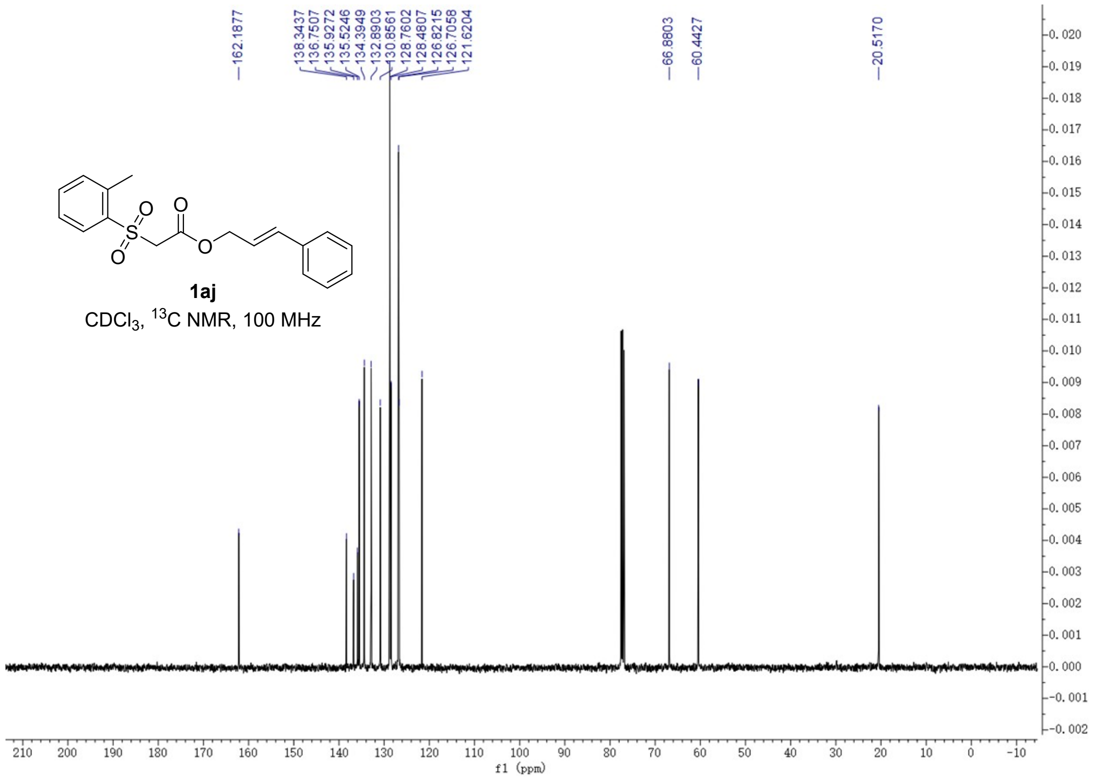
162.1877
138.3437
136.7507
135.9272
135.5246
134.3949
132.8903
130.8561
128.7602
128.4807
126.8215
126.7058
121.6204
66.8803
60.4427
20.5170
1aj
CDCl3, 13C NMR, 100 MHz
f1 (ppm)

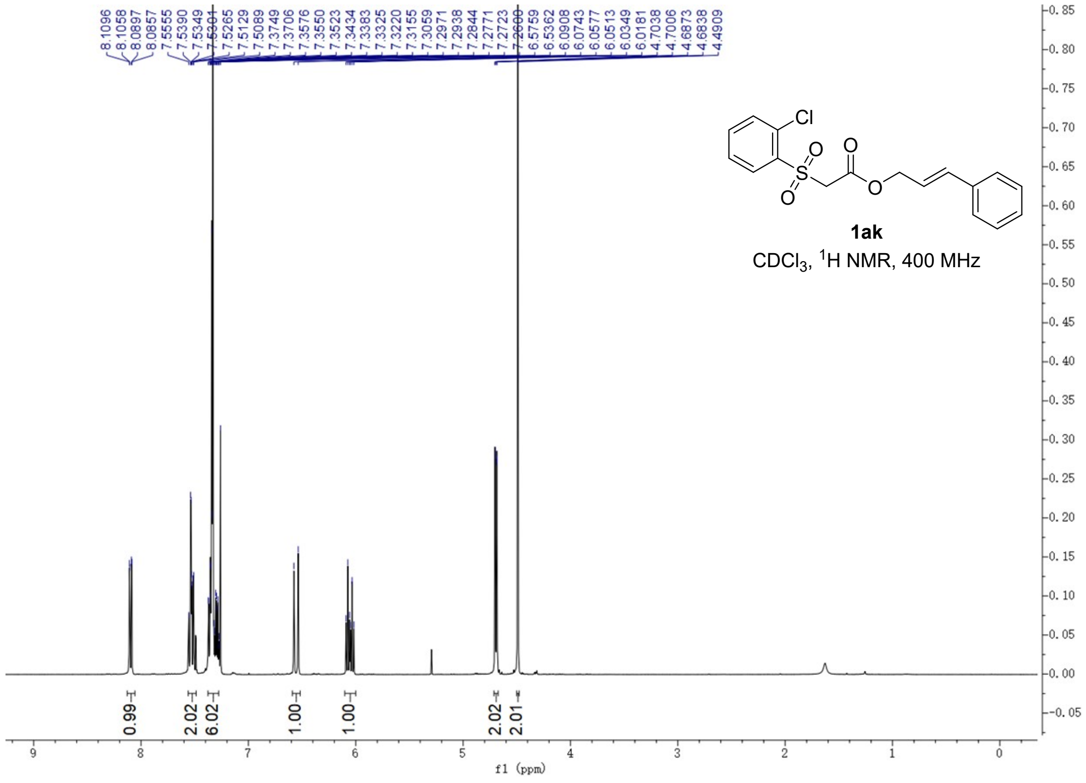

1ak

$CDCl_3$, ^1H NMR, 400 MHz

0

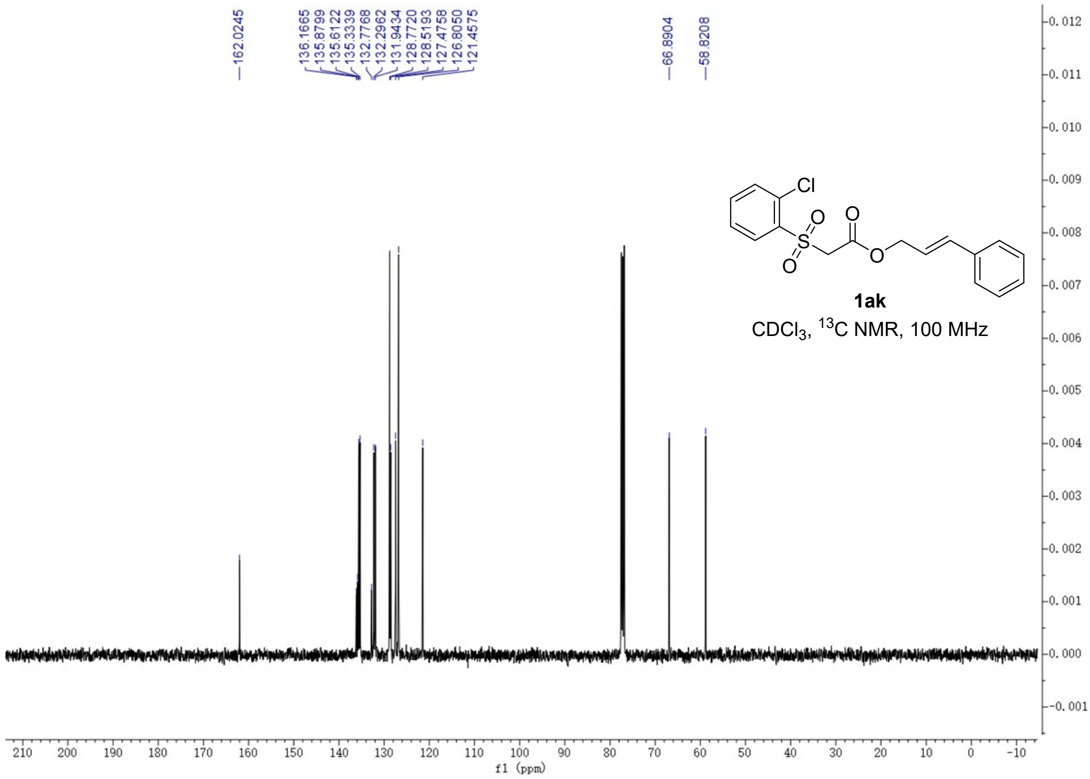

1ak

$CDCl_3$, ^{13}C NMR, 100 MHz

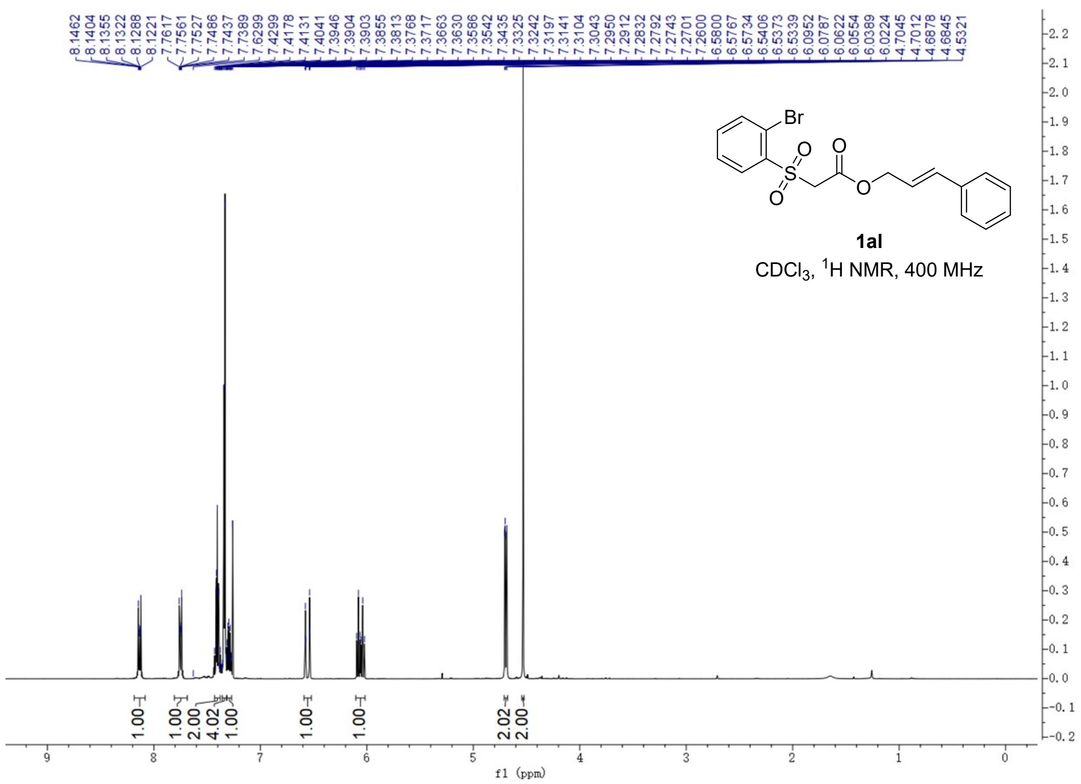
1al
CDCl3, 1H NMR, 400 MHz

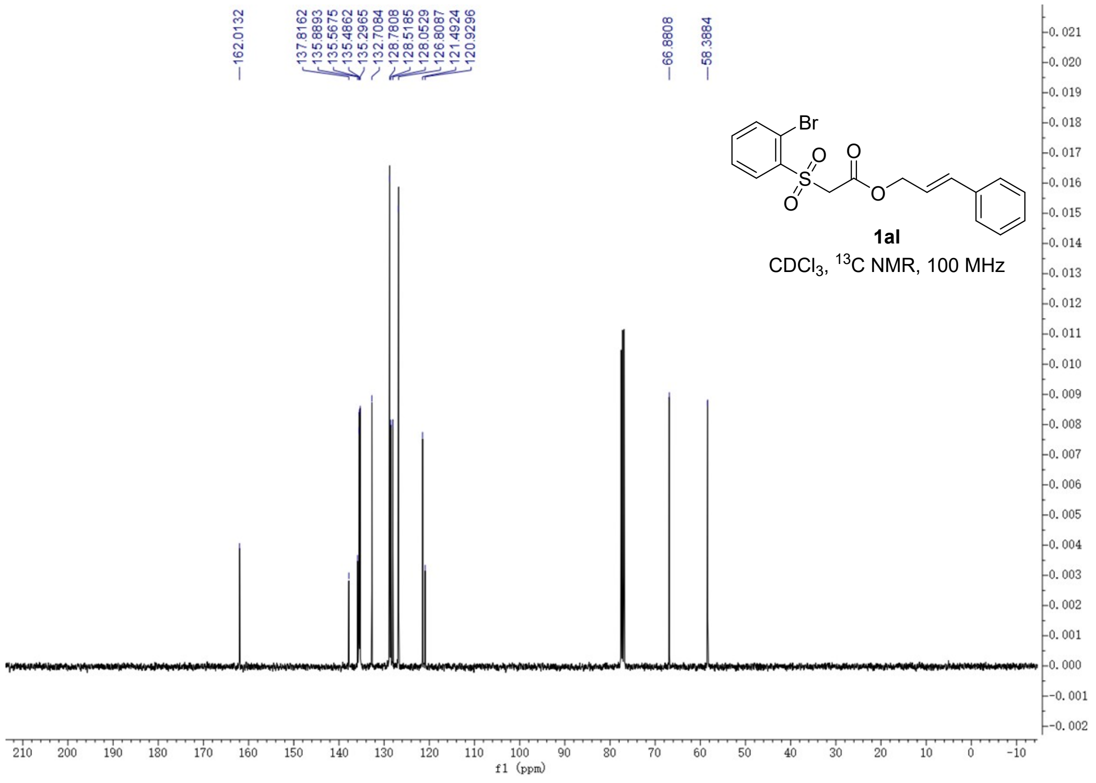
1al
CDCl3, 13C NMR, 100 MHz

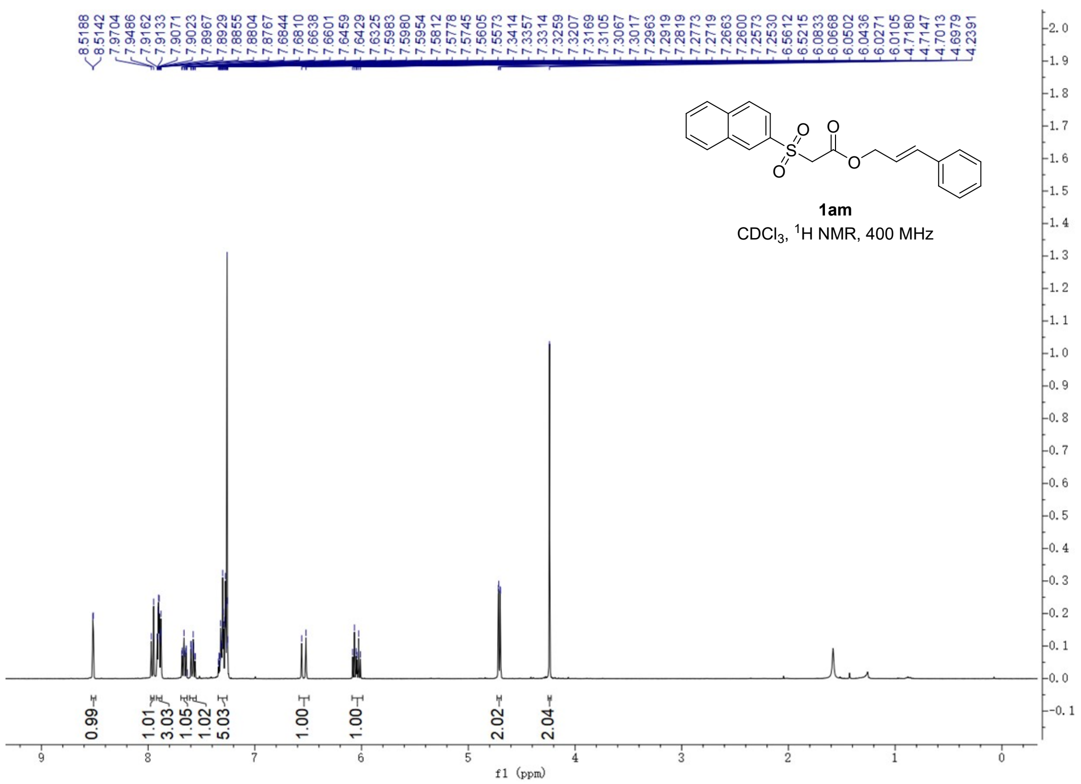

1am
CDCl3, 1H NMR, 400 MHz
f1 (ppm)

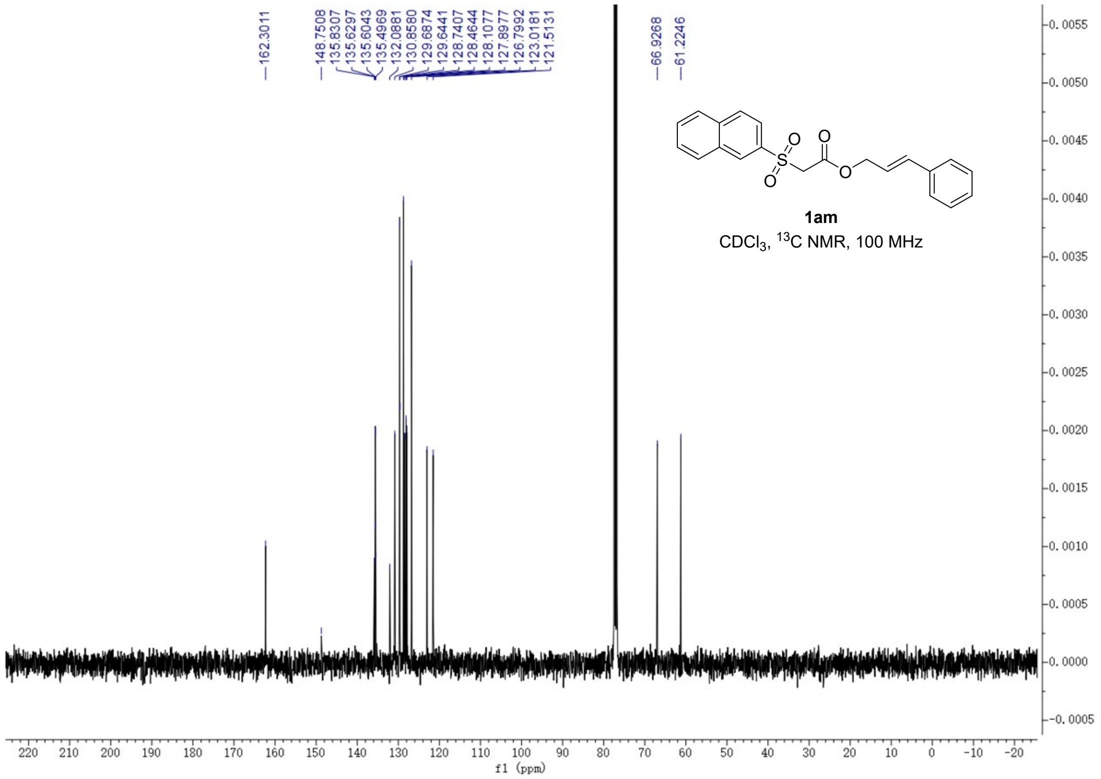

1am
CDCl3, 13C NMR, 100 MHz
f1 (ppm)

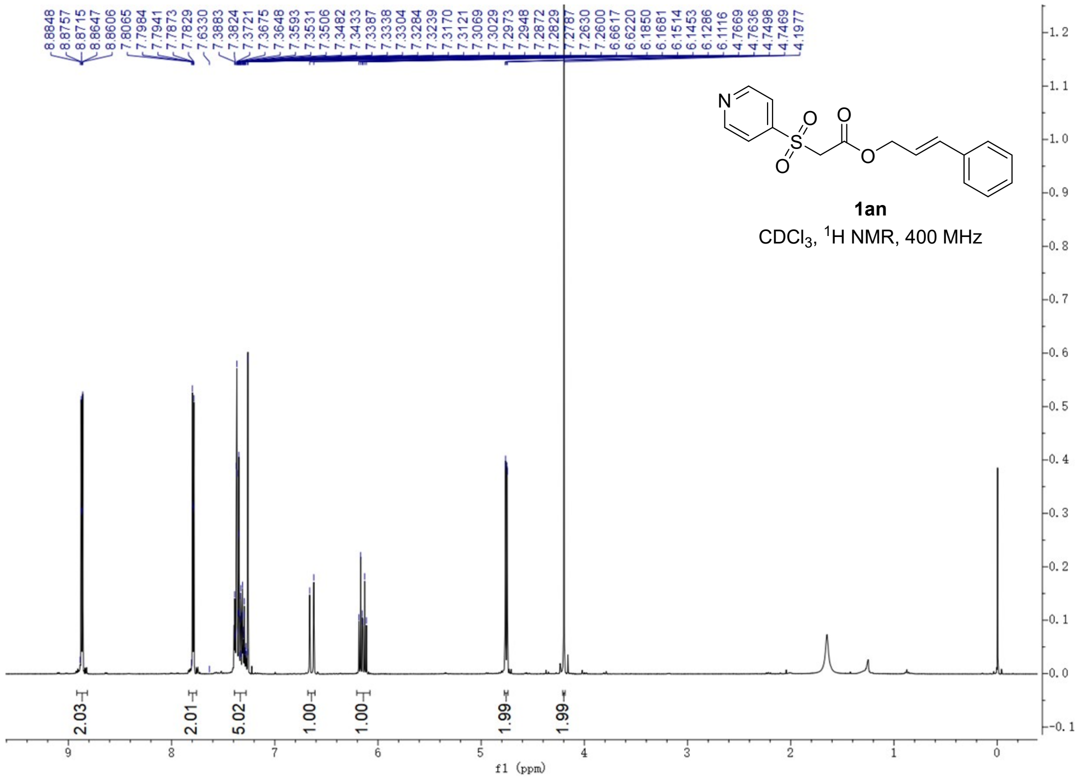
8.8848
8.8757
8.8715
8.8647
8.8606
7.8065
7.7984
7.7941
7.7873
7.7829
7.6330
7.3883
7.3824
7.3721
7.3675
7.3648
7.3593
7.3531
7.3506
7.3482
7.3433
7.3387
7.3338
7.3304
7.3284
7.3239
7.3170
7.3121
7.3069
7.3029
7.2973
7.2948
7.2872
7.2829
7.2787
7.2630
7.2600
6.6617
6.6220
6.1850
6.1681
6.1514
6.1453
6.1286
6.1116
4.7669
4.7636
4.7498
4.7469
4.1977
N
O
S
O
O
O
1an
CDCl3, 1H NMR, 400 MHz
2.03
2.01
5.02
1.00
1.00
1.99
1.99
f1 (ppm)

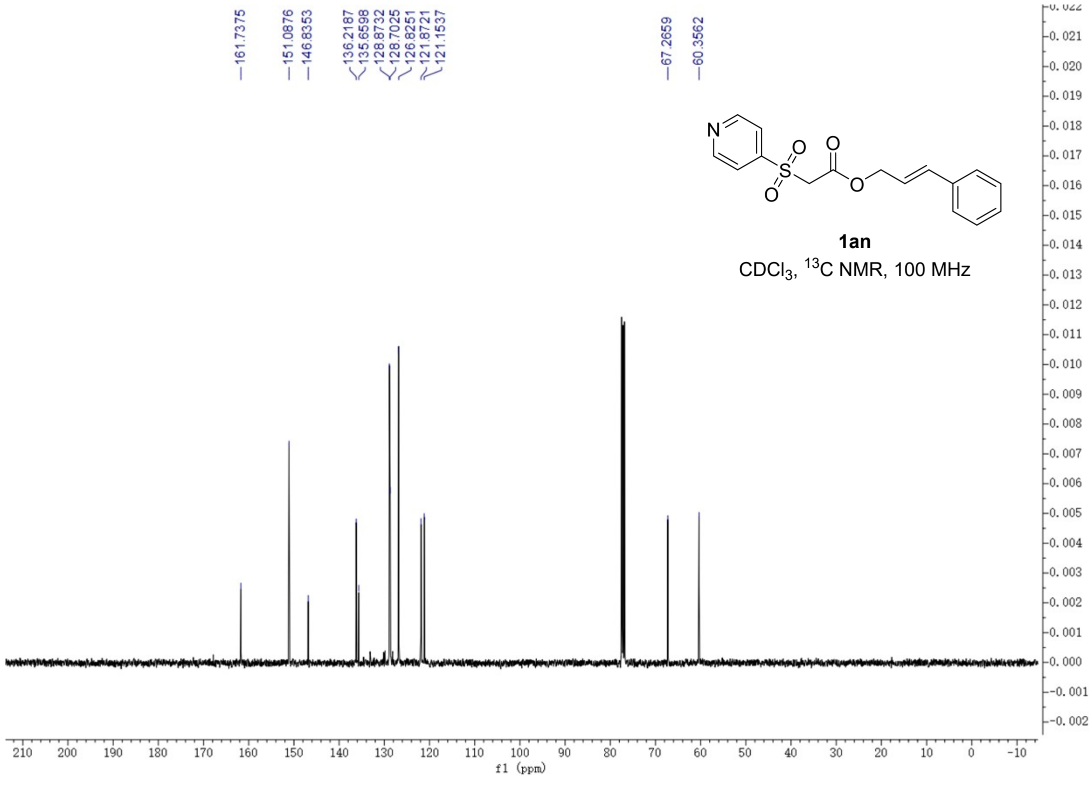
161.7375
151.0876
146.8353
136.2187
135.6598
128.8732
128.7025
126.8251
121.8721
121.1537
67.2659
60.3562
N
O
S
O
O
O
1an
CDCl3, 13C NMR, 100 MHz
f1 (ppm)

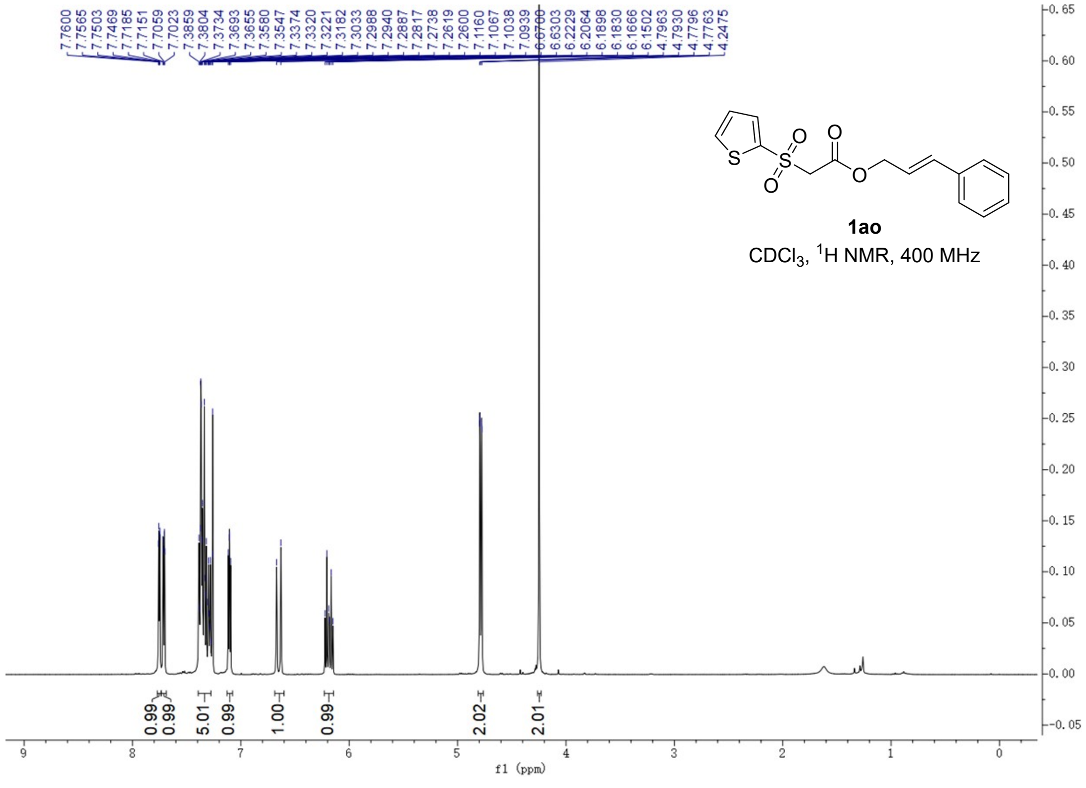
7.7600
7.7565
7.7503
7.7469
7.7185
7.7151
7.7059
7.7023
7.3859
7.3804
7.3734
7.3693
7.3655
7.3580
7.3547
7.3374
7.3320
7.3221
7.3182
7.3033
7.2988
7.2940
7.2887
7.2817
7.2738
7.2619
7.2600
7.1160
7.1067
7.1038
7.0939
6.6700
6.6303
6.2229
6.2064
6.1898
6.1830
6.1666
6.1502
4.7963
4.7930
4.7796
4.7763
4.2475
1ao
CDCl3, 1H NMR, 400 MHz
0.99
0.99
5.01
0.99
1.00
0.99
2.02
2.01
f1 (ppm)

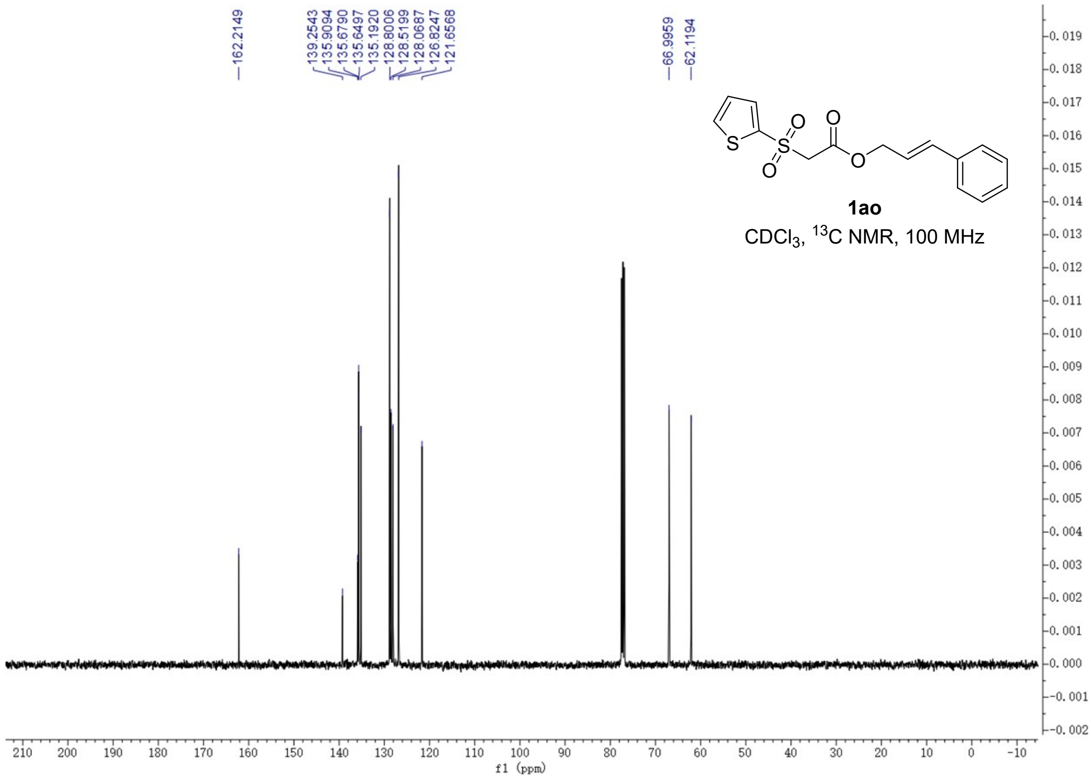
162.2149
139.2543
135.9094
135.6790
135.6497
135.1920
128.8006
128.5199
128.0687
126.8247
121.6568
66.9959
62.1194
1ao
CDCl3, 13C NMR, 100 MHz
f1 (ppm)

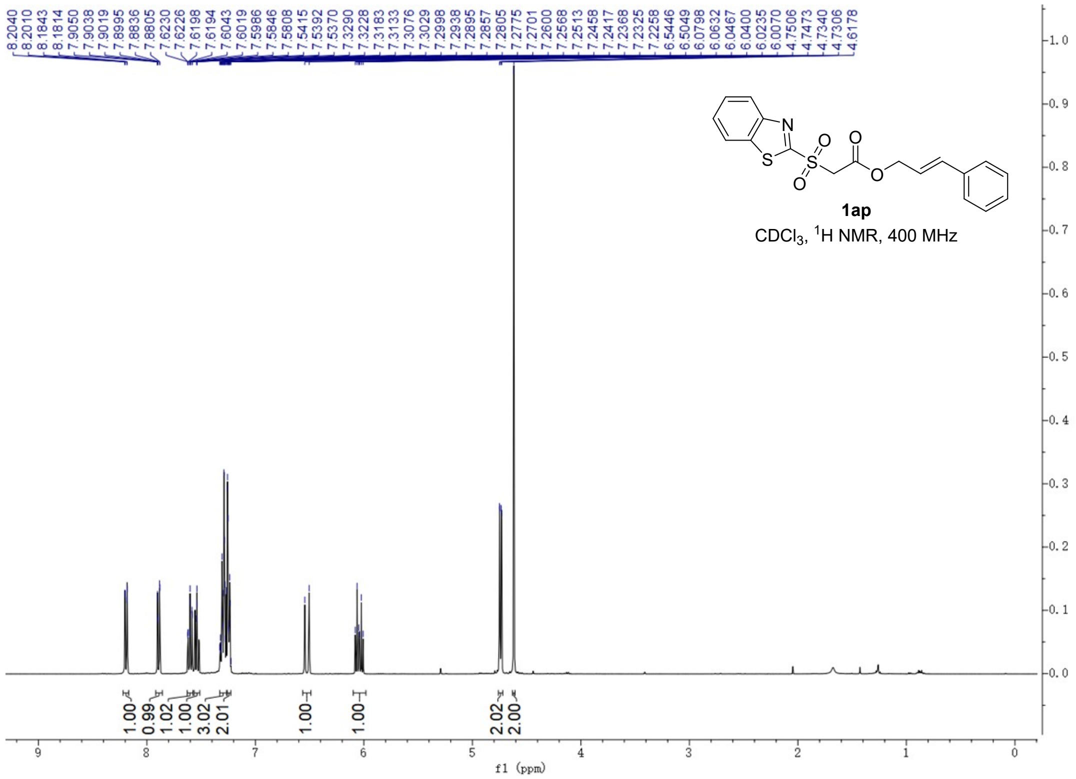
1ap
CDCl3, 1H NMR, 400 MHz
f1 (ppm)

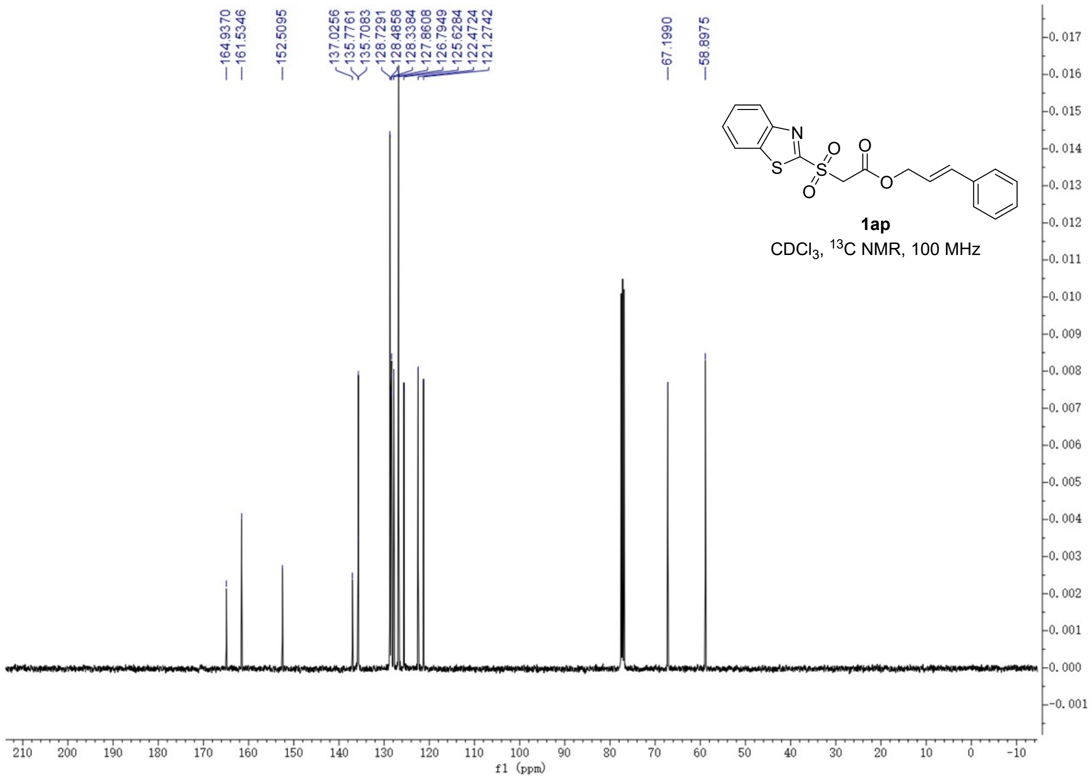
1ap
CDCl3, 13C NMR, 100 MHz
f1 (ppm)

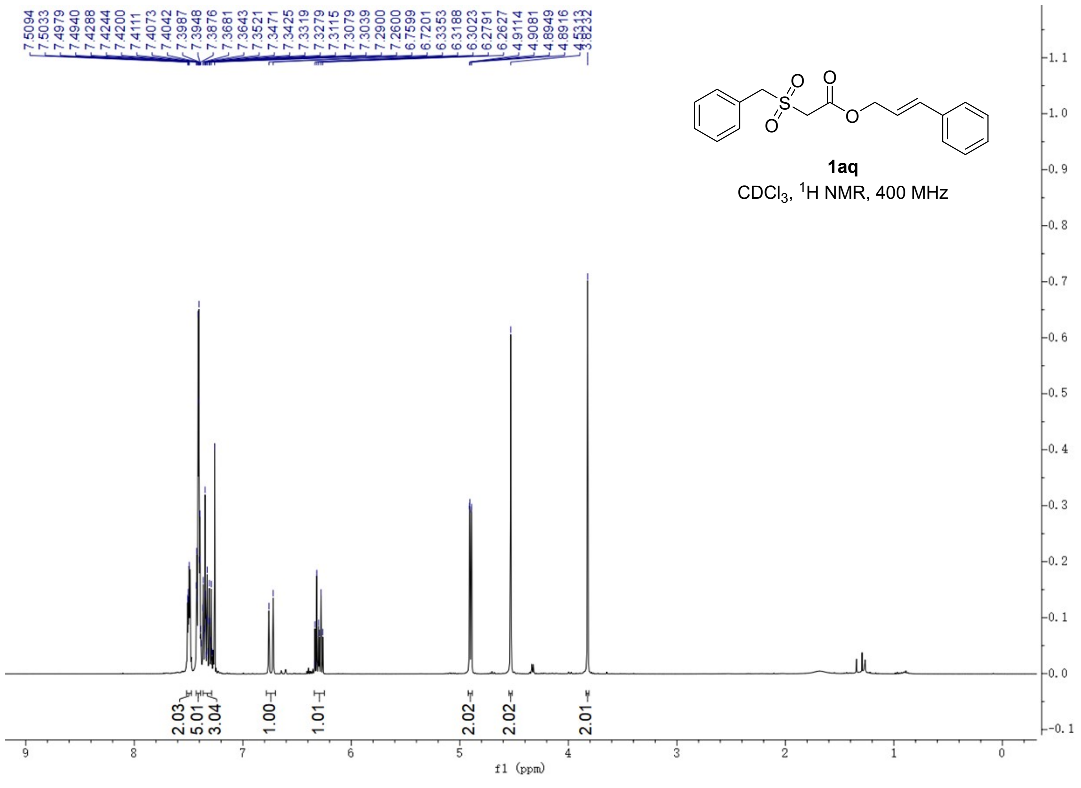
1aq
CDCl3, 1H NMR, 400 MHz
f1 (ppm)

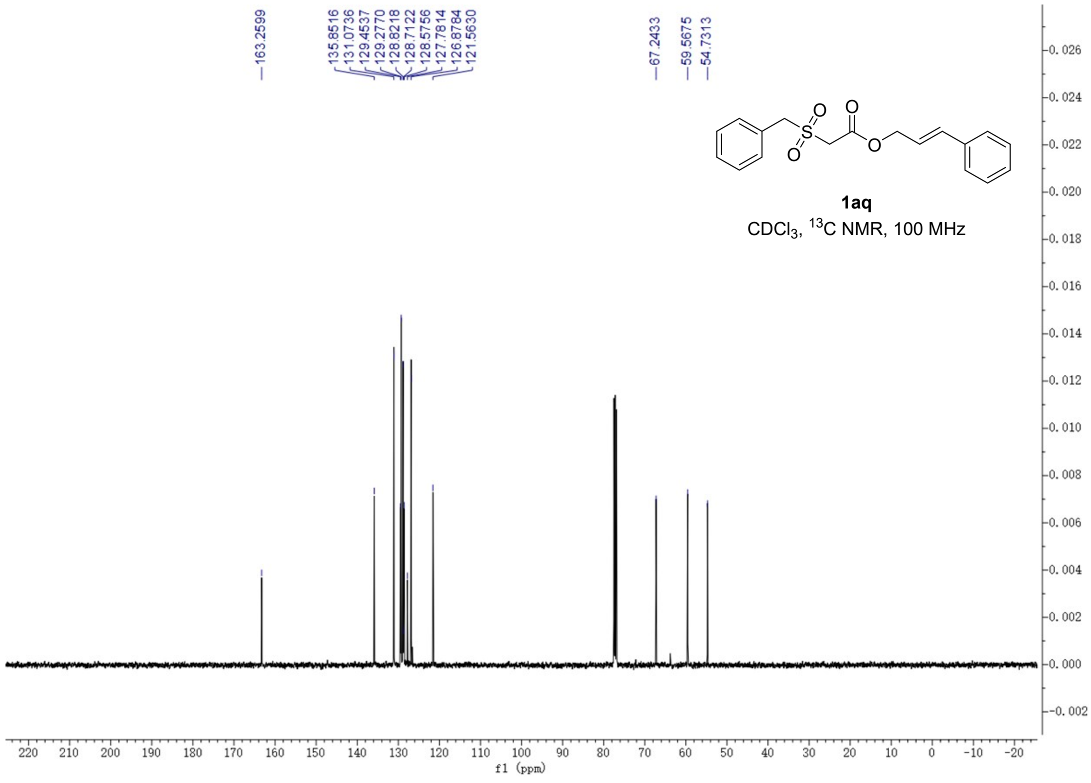
1aq
CDCl3, 13C NMR, 100 MHz
f1 (ppm)

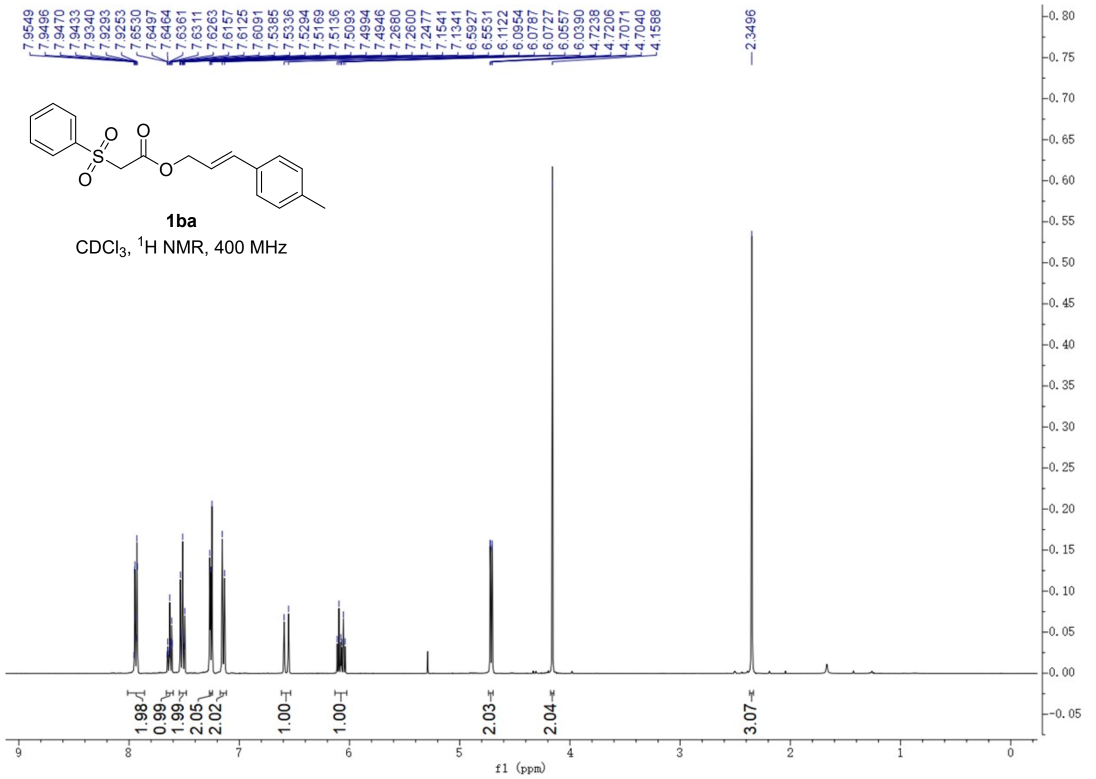
1ba
CDCl3, 1H NMR, 400 MHz
f1 (ppm)

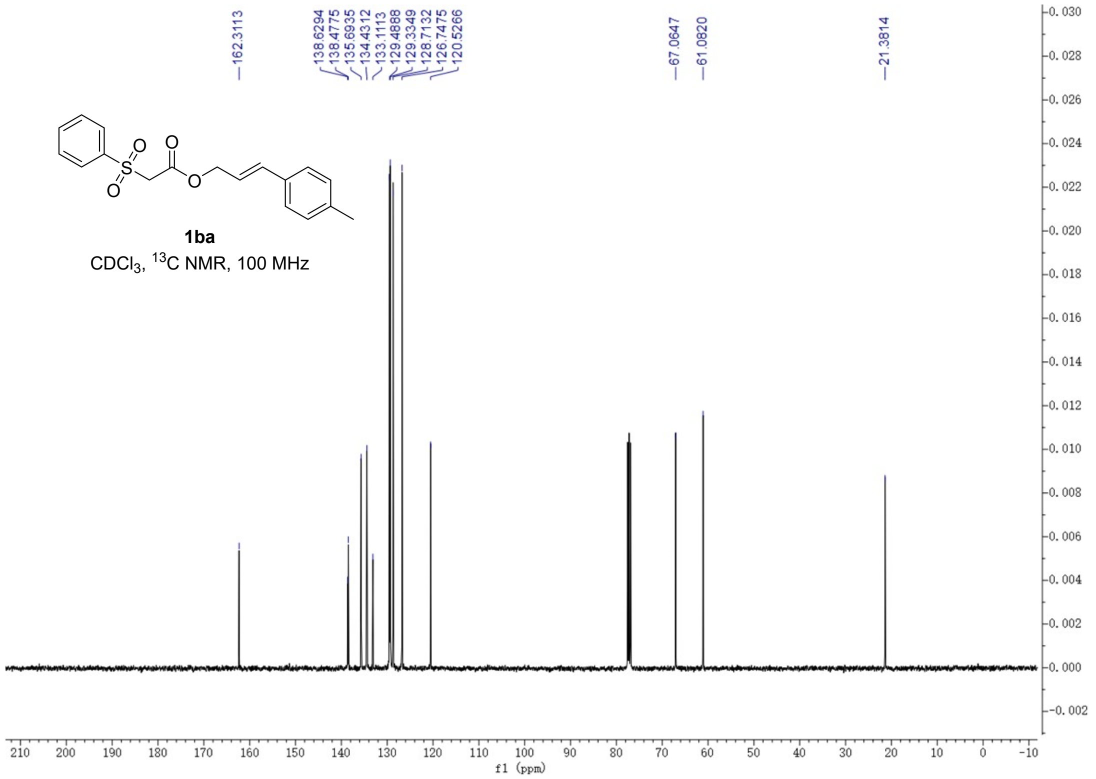
1ba
CDCl3, 13C NMR, 100 MHz
f1 (ppm)

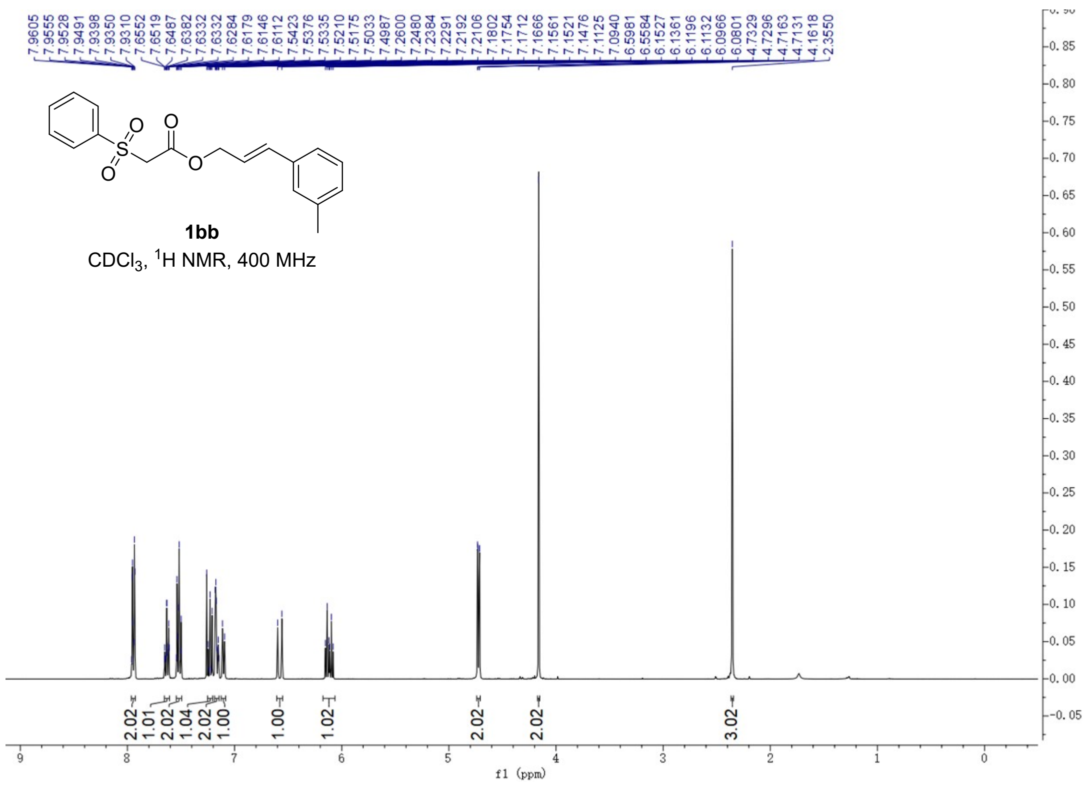
1bb
CDCl3, 1H NMR, 400 MHz
f1 (ppm)

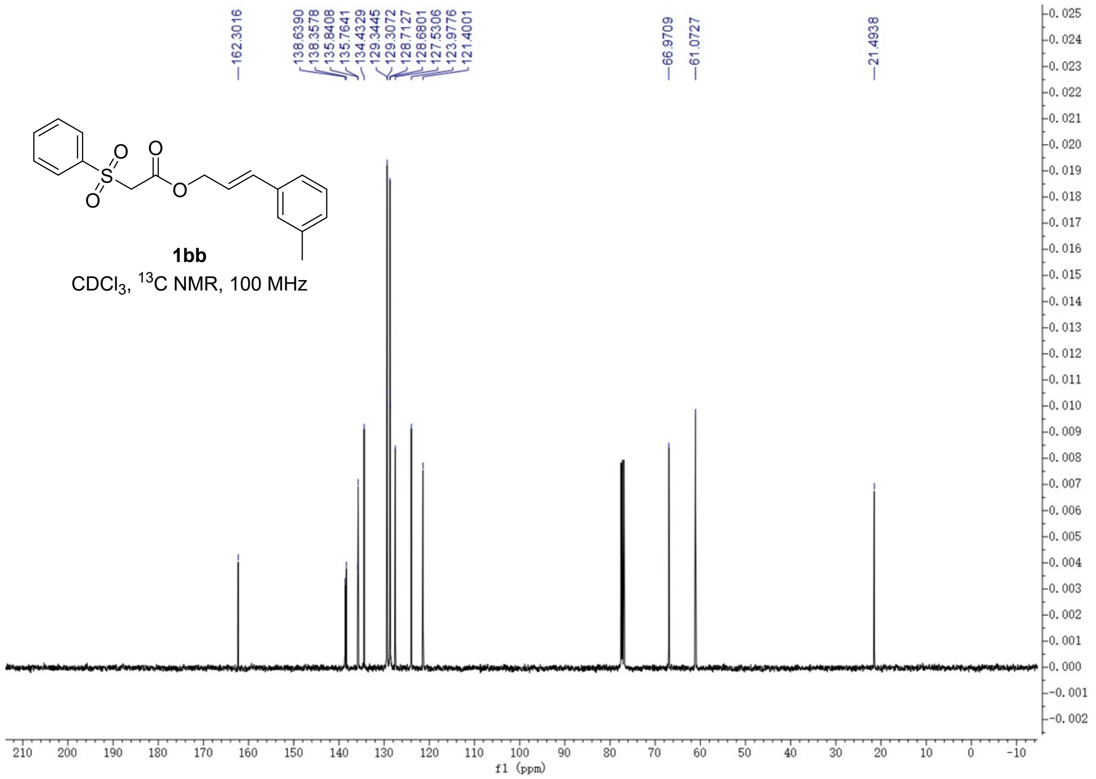
1bb
CDCl3, 13C NMR, 100 MHz
f1 (ppm)

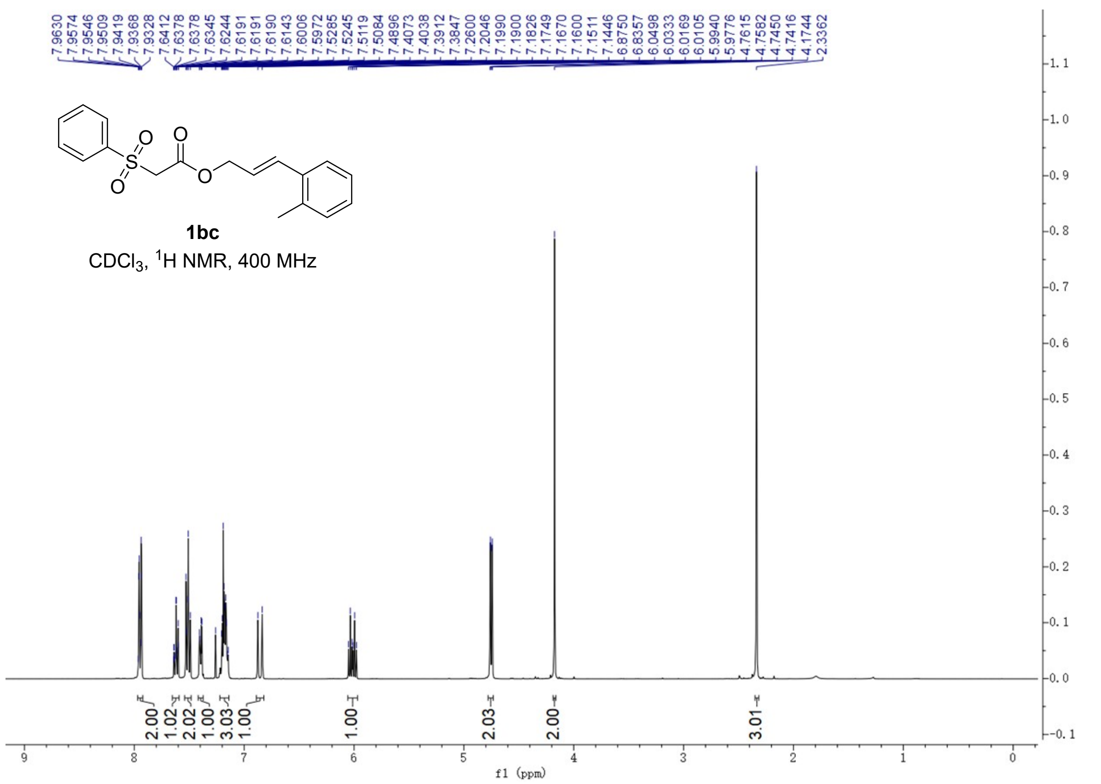

1bc

$CDCl_3$, ^1H NMR, 400 MHz

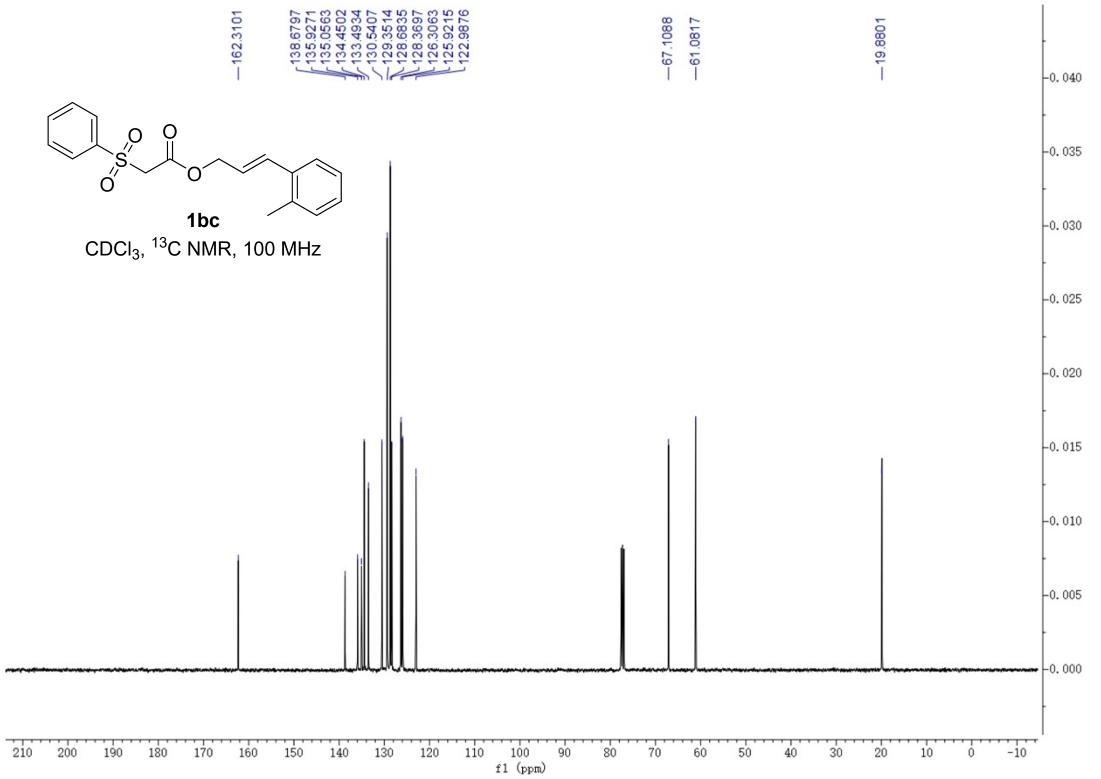

1bc

$CDCl_3$, ^{13}C NMR, 100 MHz

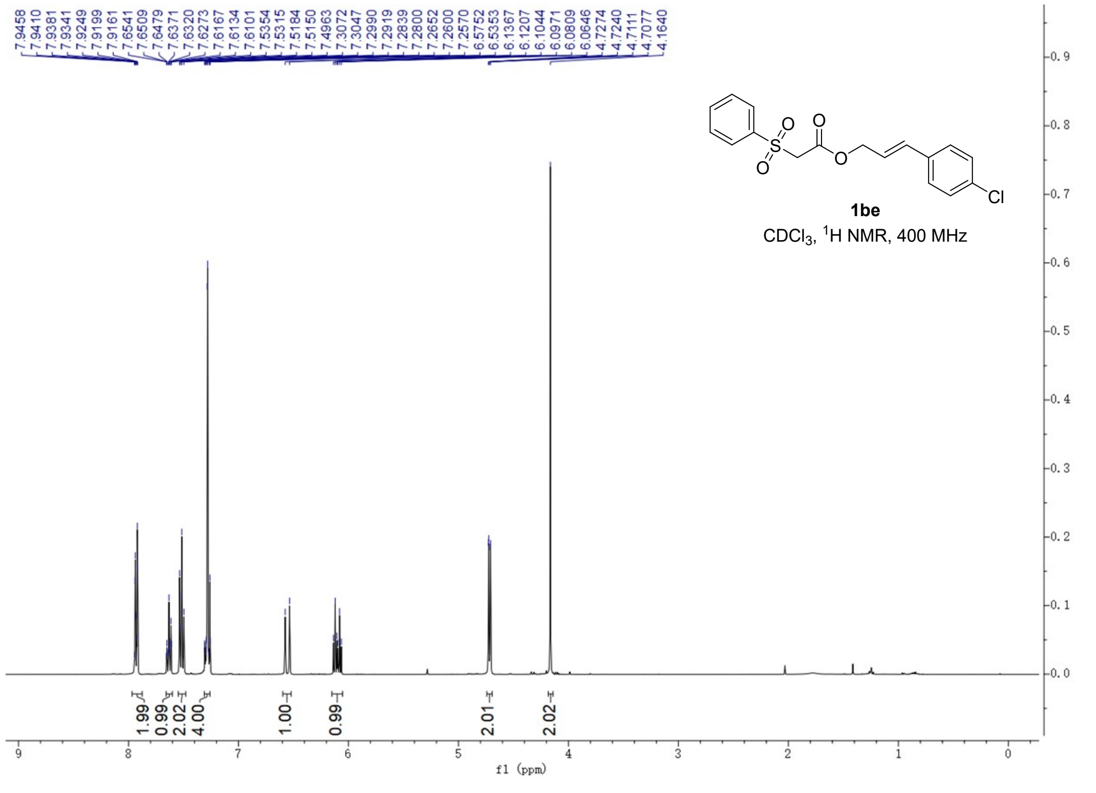
1be
CDCl3, 1H NMR, 400 MHz
f1 (ppm)

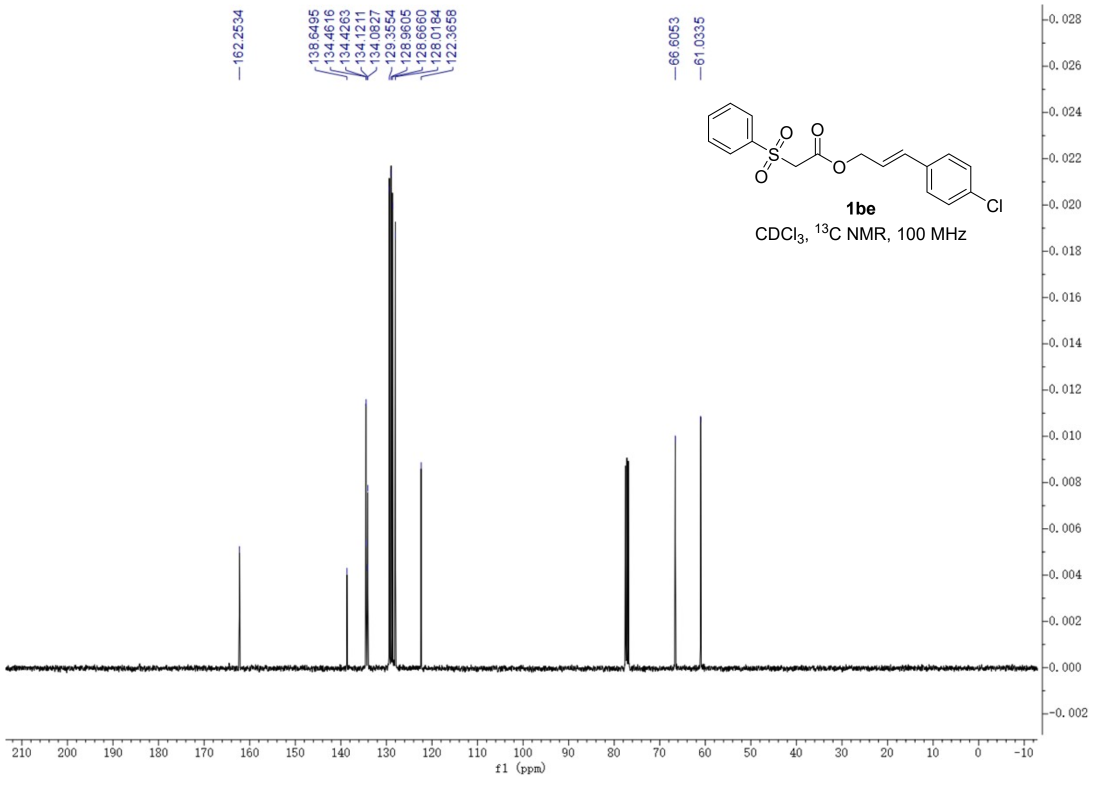
1be
CDCl3, 13C NMR, 100 MHz
f1 (ppm)

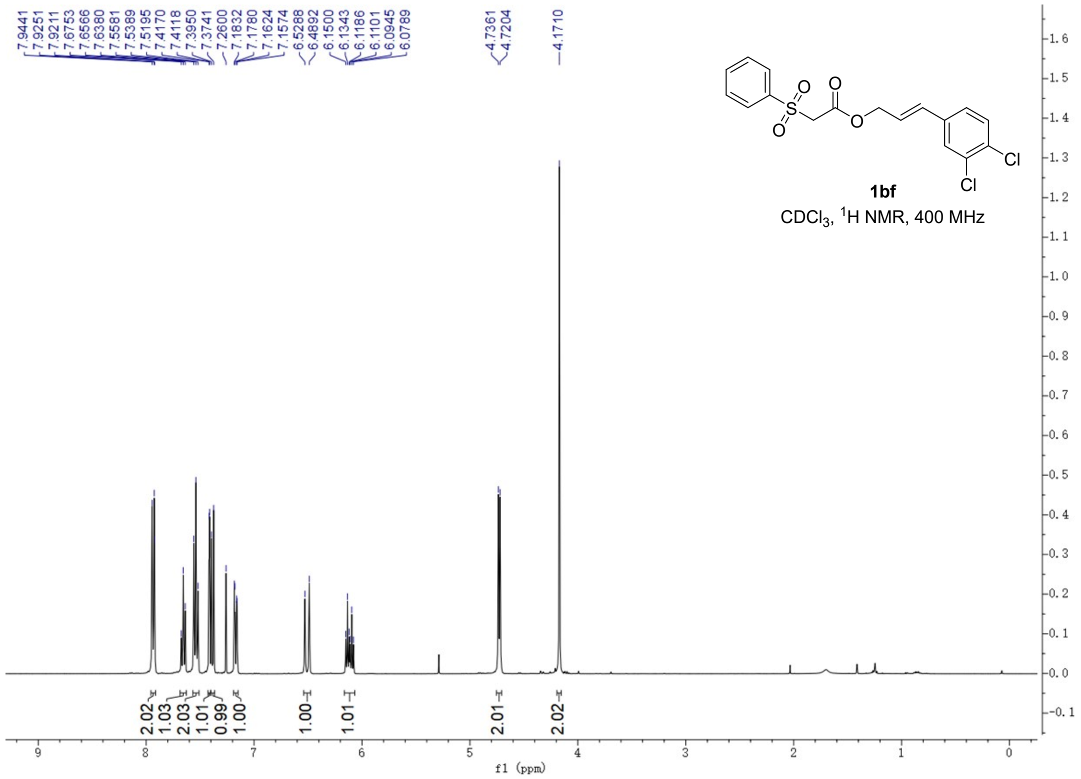
1bf
$CDCl_3$, ^1H NMR, 400 MHz
f1 (ppm)

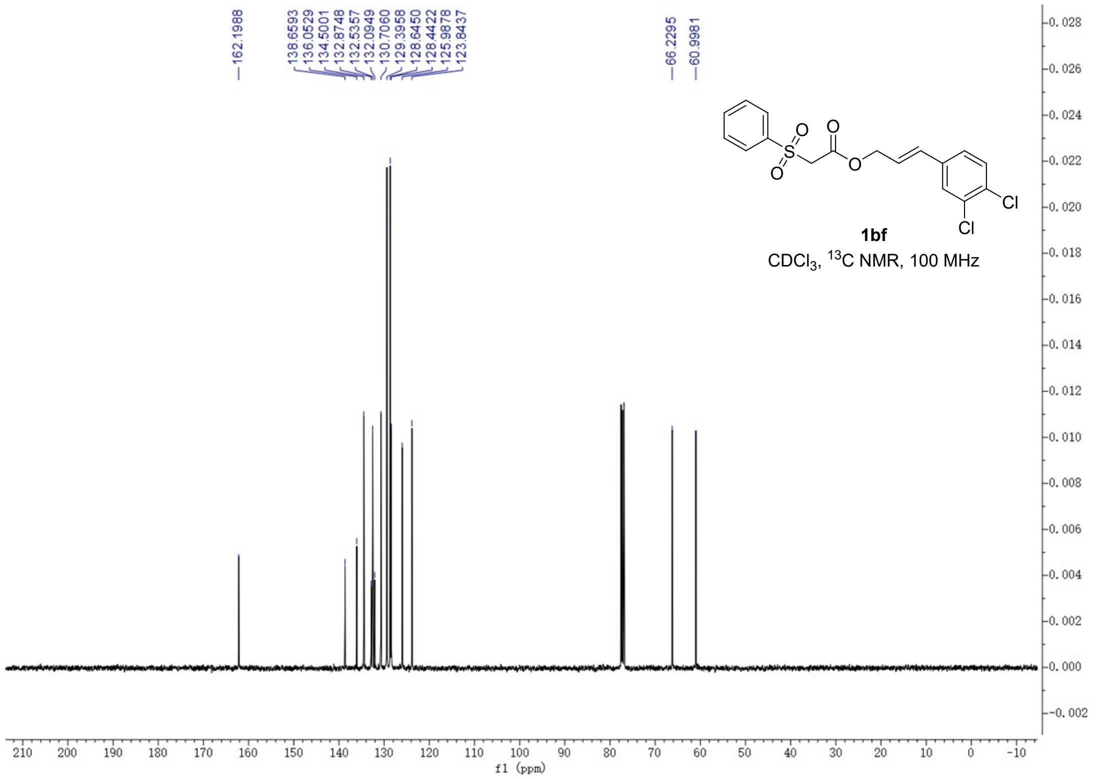
1bf
$CDCl_3$, ^{13}C NMR, 100 MHz
f1 (ppm)

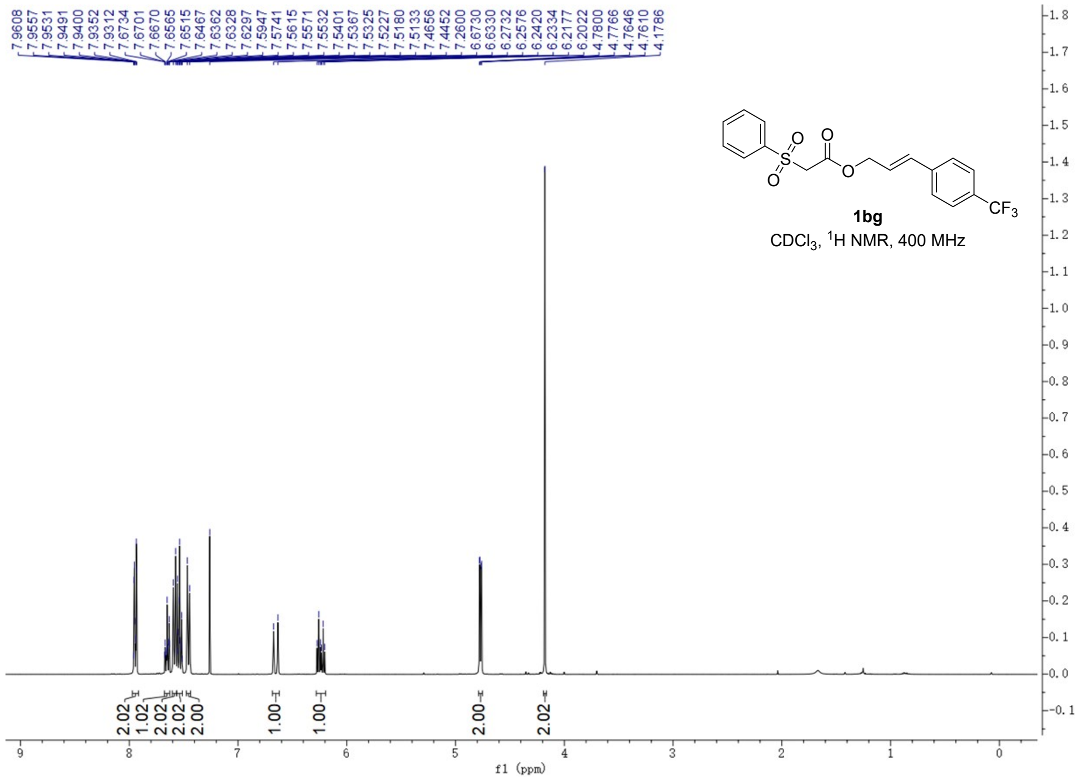
1bg
CDCl3, 1H NMR, 400 MHz
f1 (ppm)

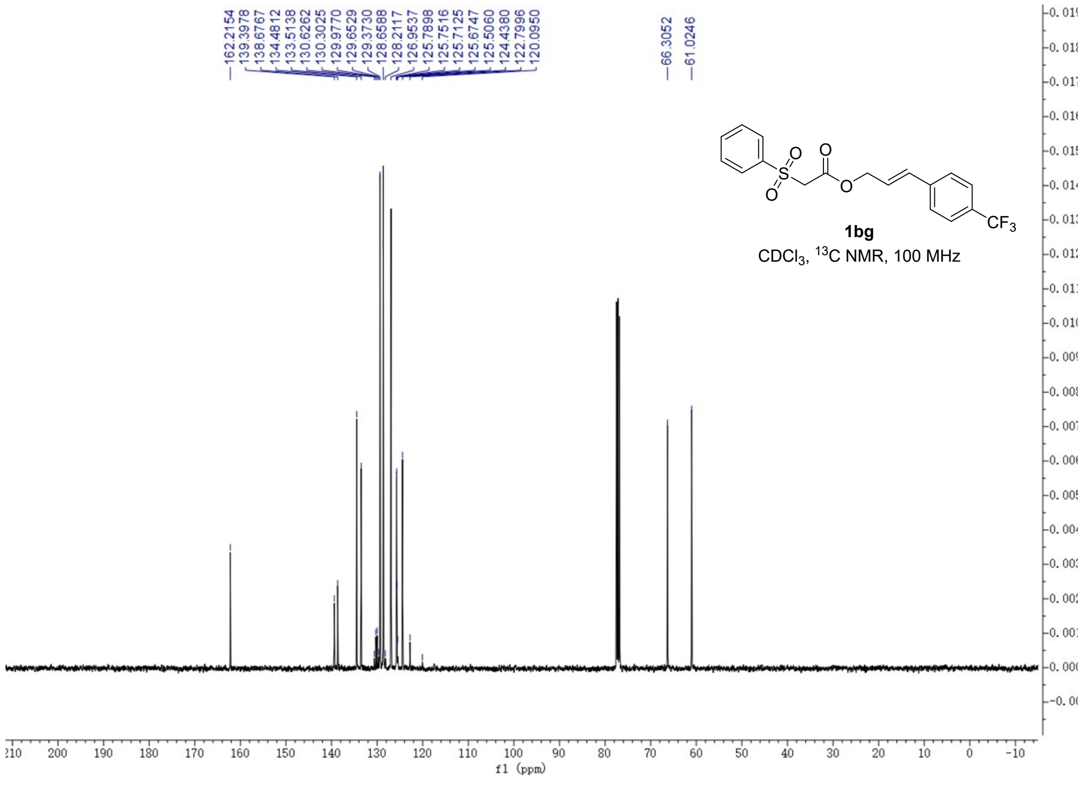
1bg
CDCl3, 13C NMR, 100 MHz
f1 (ppm)

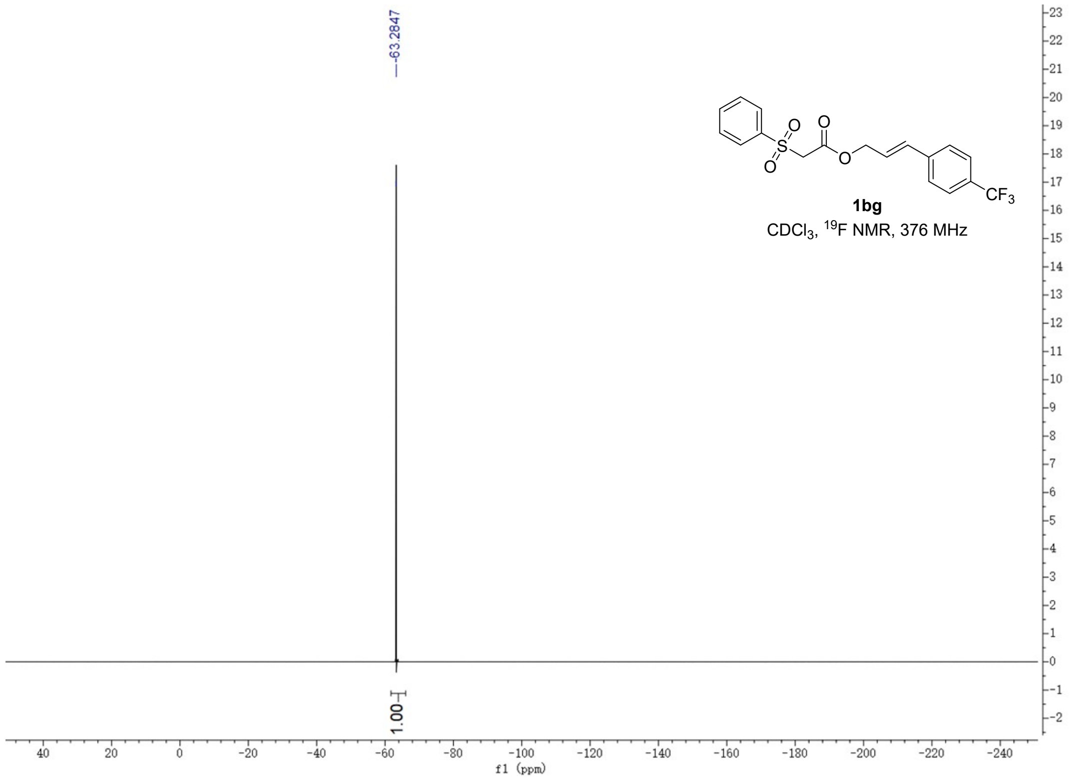

-63.2847
1bg
CDCl3, 19F NMR, 376 MHz
1.00
f1 (ppm)

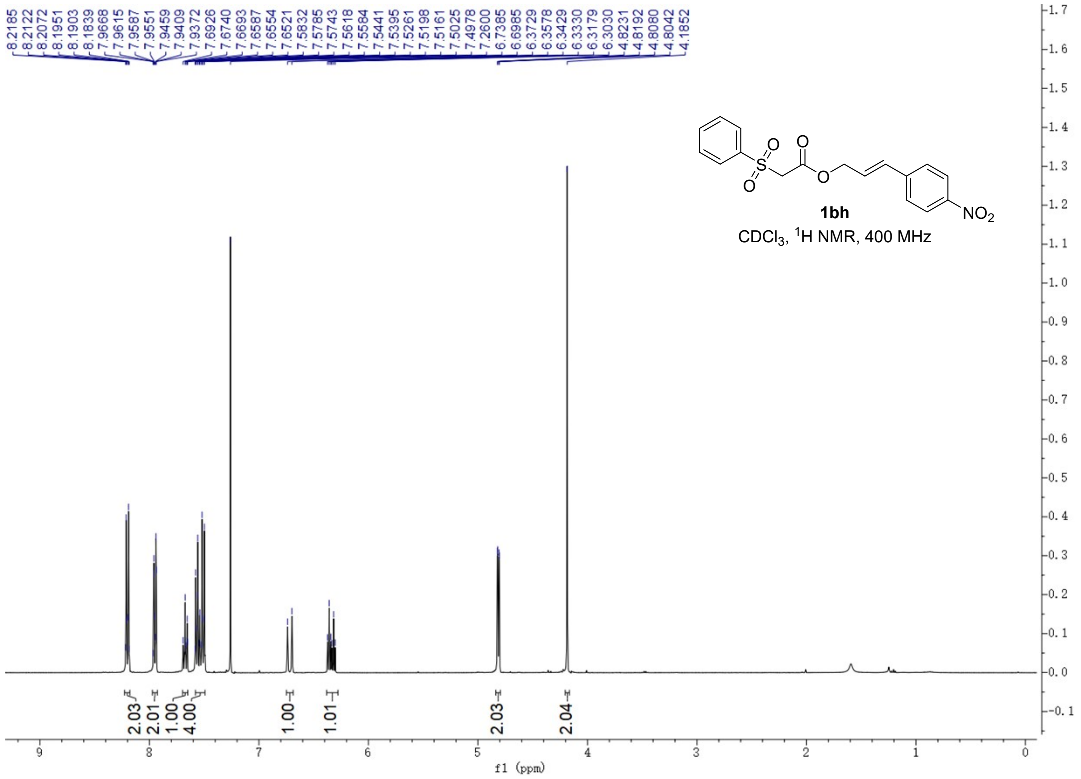
8.2185
8.2122
8.2072
8.1951
8.1903
8.1839
7.9668
7.9615
7.9587
7.9551
7.9459
7.9409
7.9372
7.6926
7.6740
7.6693
7.6587
7.6554
7.6521
7.5832
7.5785
7.5743
7.5618
7.5584
7.5441
7.5395
7.5261
7.5198
7.5161
7.5025
7.4978
7.2600
6.7385
6.6985
6.3729
6.3578
6.3429
6.3330
6.3179
6.3030
4.8231
4.8192
4.8080
4.8042
4.1852
1bh
CDCl3, 1H NMR, 400 MHz
2.03
2.01
1.00
4.00
1.00
1.01
2.03
2.04
f1 (ppm)

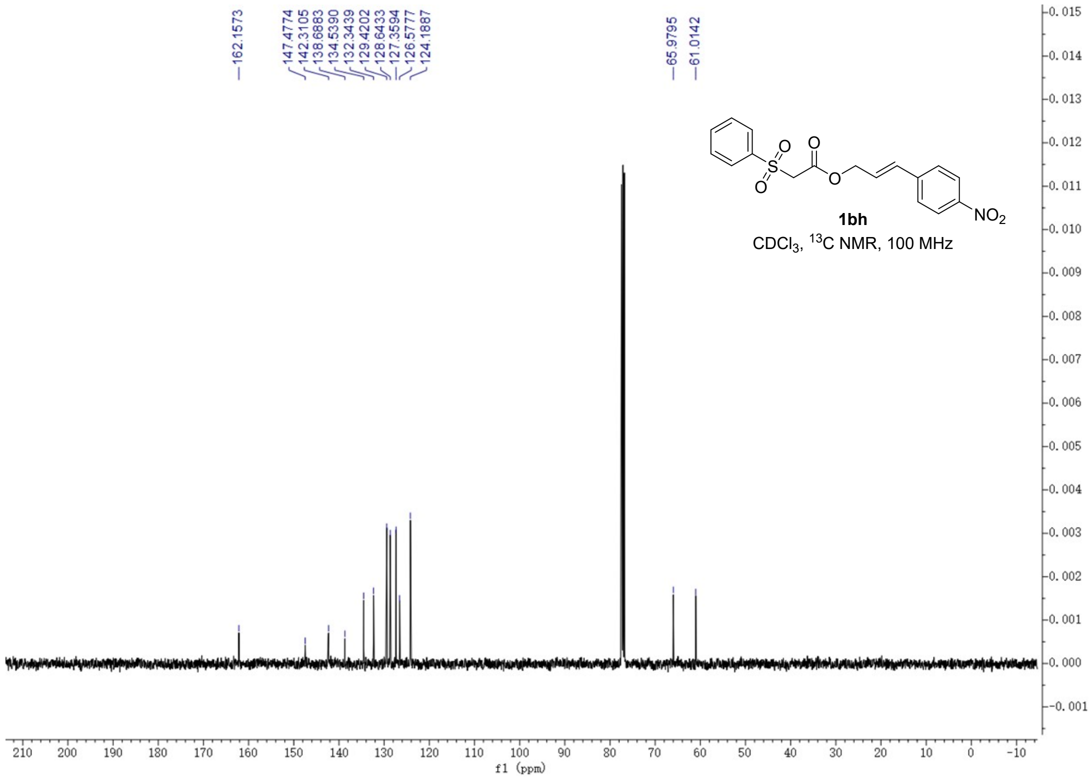
162.1573
147.4774
142.3105
138.6883
134.5390
132.3439
129.4202
128.6433
127.3594
126.5777
124.1887
65.9795
61.0142
1bh
CDCl3, 13C NMR, 100 MHz
f1 (ppm)

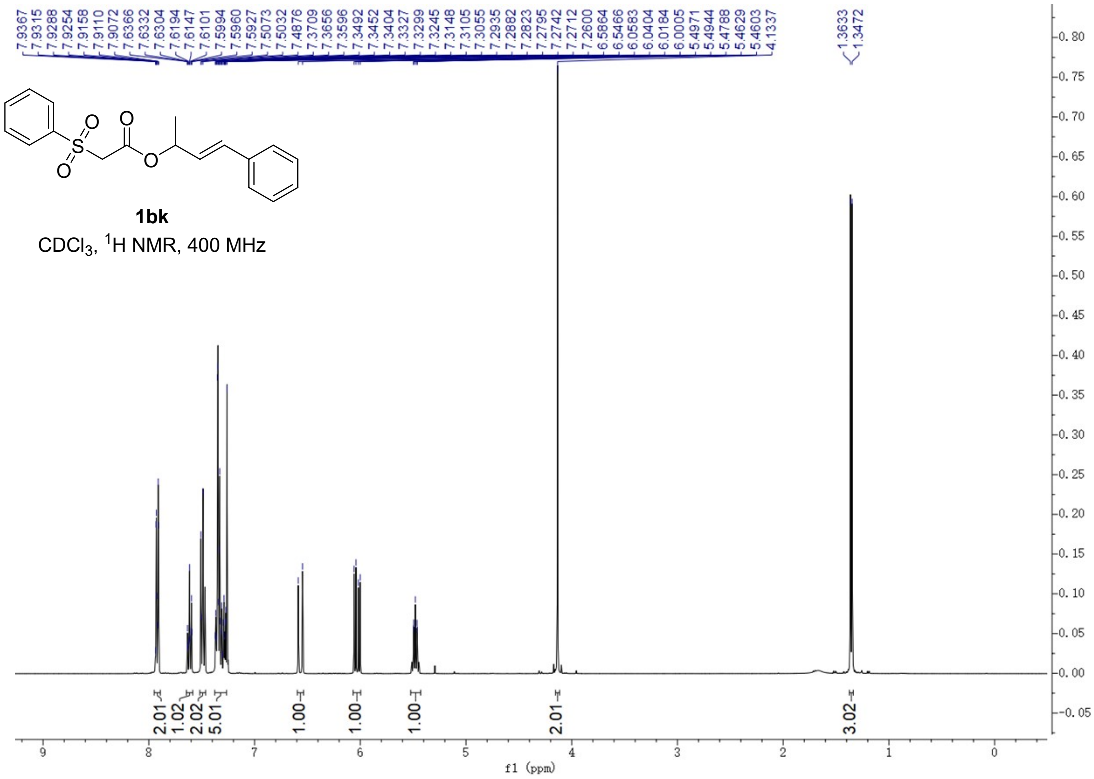
7.9367
7.9315
7.9288
7.9254
7.9158
7.9110
7.9072
7.6366
7.6332
7.6304
7.6194
7.6147
7.6101
7.5994
7.5960
7.5927
7.5073
7.5032
7.4876
7.3709
7.3656
7.3596
7.3492
7.3452
7.3404
7.3327
7.3299
7.3245
7.3148
7.3105
7.3055
7.2935
7.2882
7.2823
7.2795
7.2742
7.2712
7.2600
6.5864
6.5466
6.0583
6.0404
6.0184
6.0005
5.4971
5.4944
5.4788
5.4629
5.4603
4.1337
1.3633
1.3472
1bk
CDCl3, 1H NMR, 400 MHz
2.01
1.02
2.02
5.01
1.00
1.00
1.00
2.01
3.02
f1 (ppm)

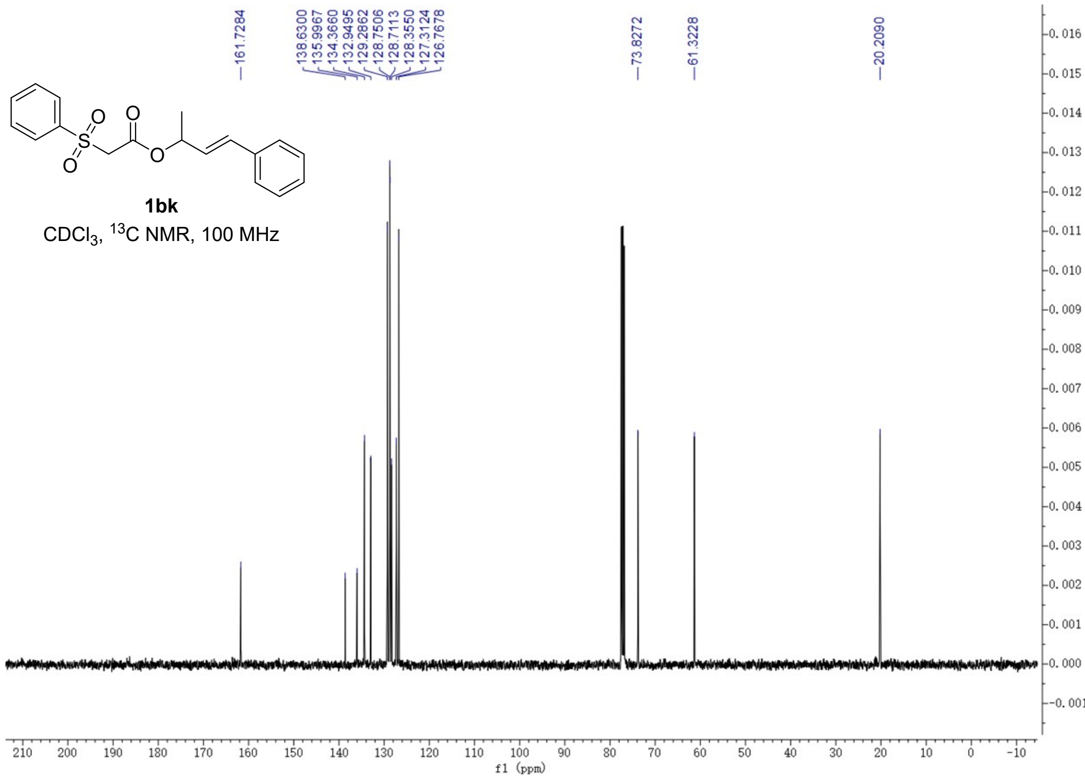
161.7284
138.6300
135.9967
134.3660
132.9495
129.2862
128.7506
128.7113
128.3550
127.3124
126.7678
73.8272
61.3228
20.2090
1bk
CDCl3, 13C NMR, 100 MHz
f1 (ppm)

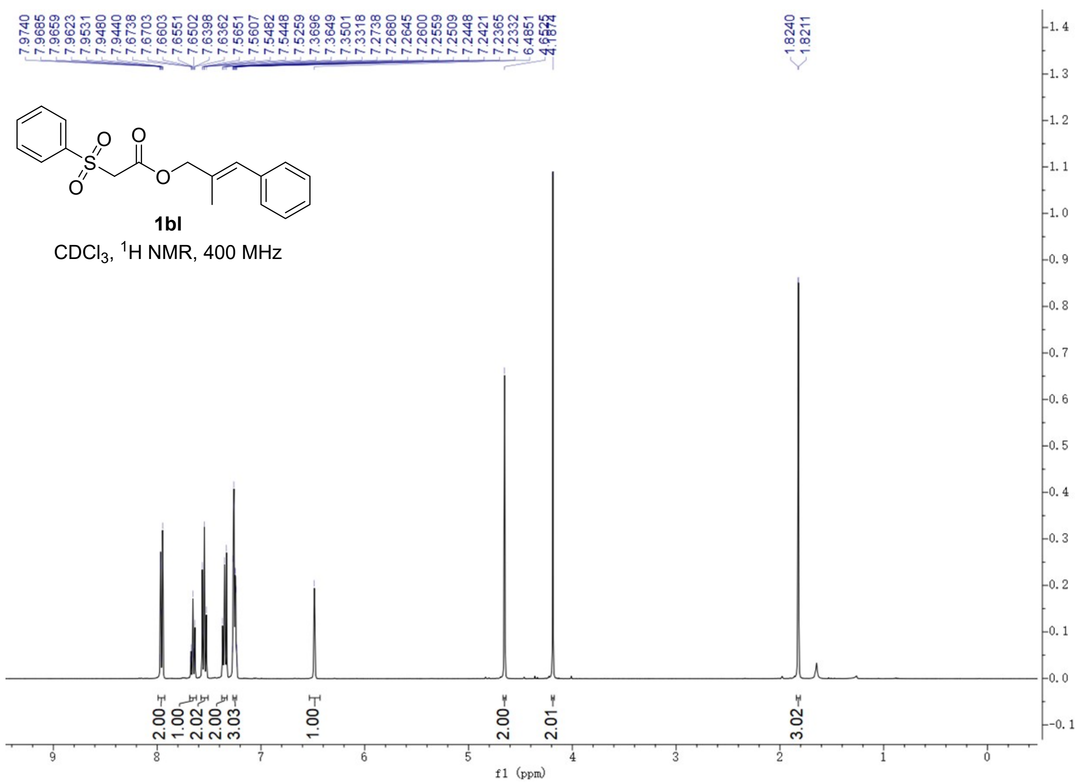
7.9740
7.9685
7.9659
7.9623
7.9531
7.9480
7.9440
7.6738
7.6703
7.6603
7.6551
7.6502
7.6398
7.6362
7.5651
7.5607
7.5482
7.5448
7.5259
7.3696
7.3649
7.3501
7.3318
7.2738
7.2680
7.2645
7.2600
7.2559
7.2509
7.2448
7.2421
7.2365
7.2332
6.4851
4.6525
4.1874
1.8240
1.8211
1bl
CDCl3, 1H NMR, 400 MHz
2.00
1.00
2.02
2.00
3.03
1.00
2.00
2.01
3.02
f1 (ppm)

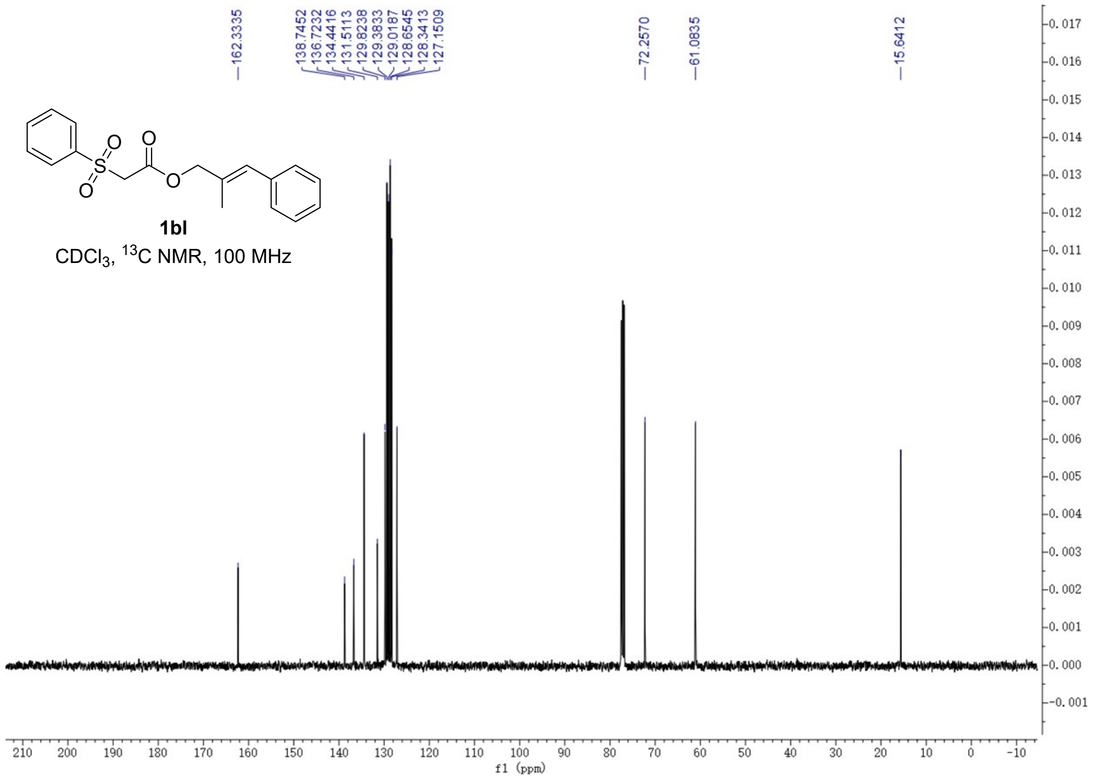
162.3335
138.7452
136.7232
134.4416
131.5113
129.8238
129.3833
129.0187
128.6545
128.3413
127.1509
72.2570
61.0835
15.6412
1bl
CDCl3, 13C NMR, 100 MHz
f1 (ppm)

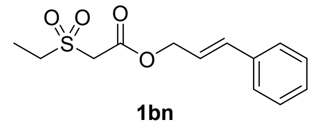

1bn

$CDCl_3$, ^{1}H NMR, 400 MHz

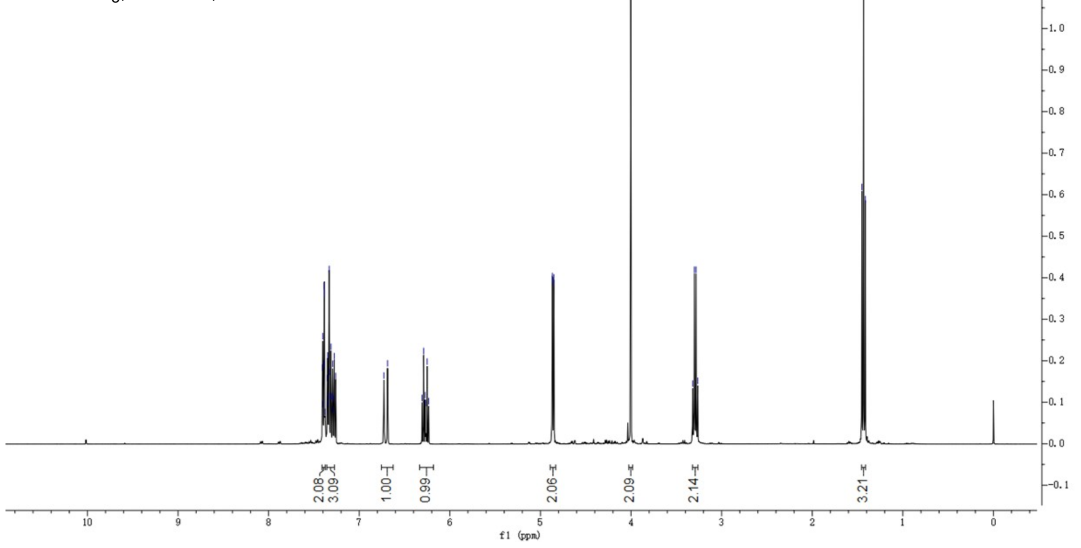

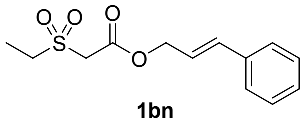

1bn

$CDCl_3$, ^{13}C NMR, 100 MHz

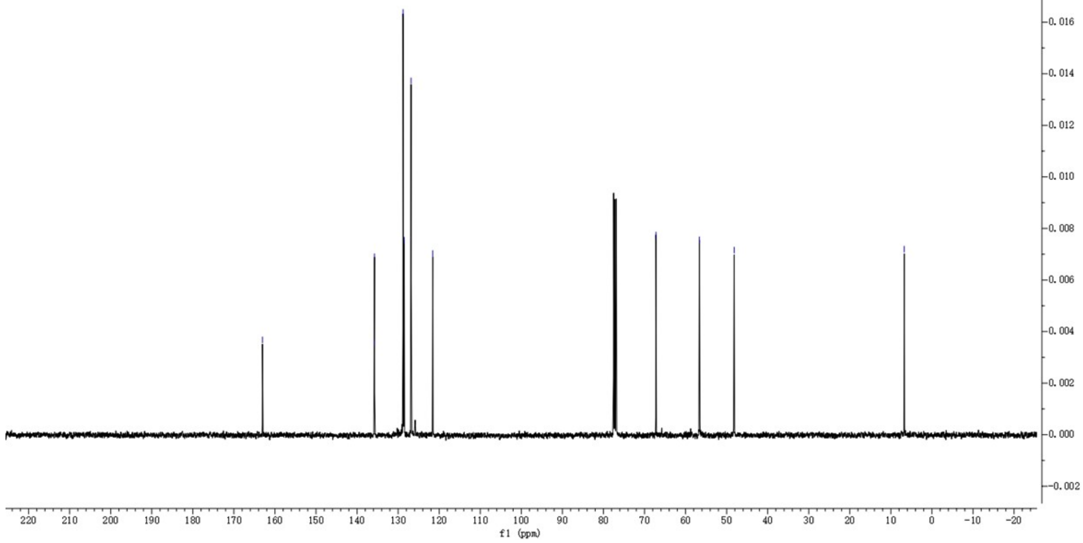

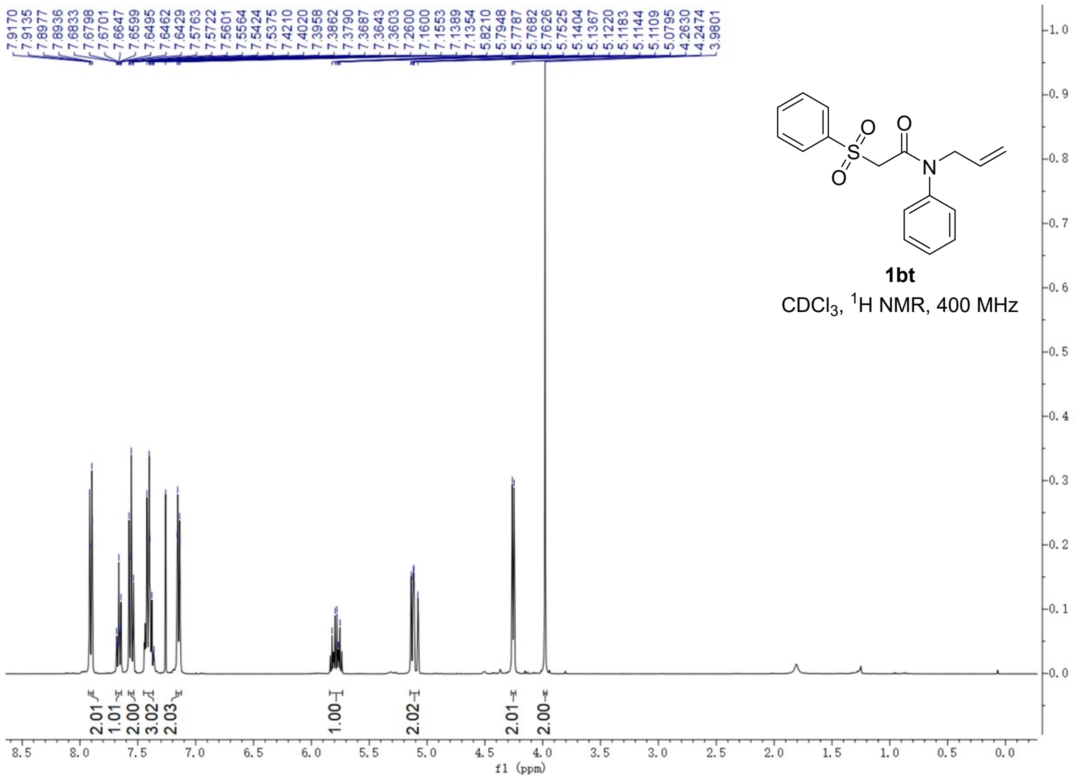

1bt

$CDCl_3$, ^{1}H NMR, 400 MHz

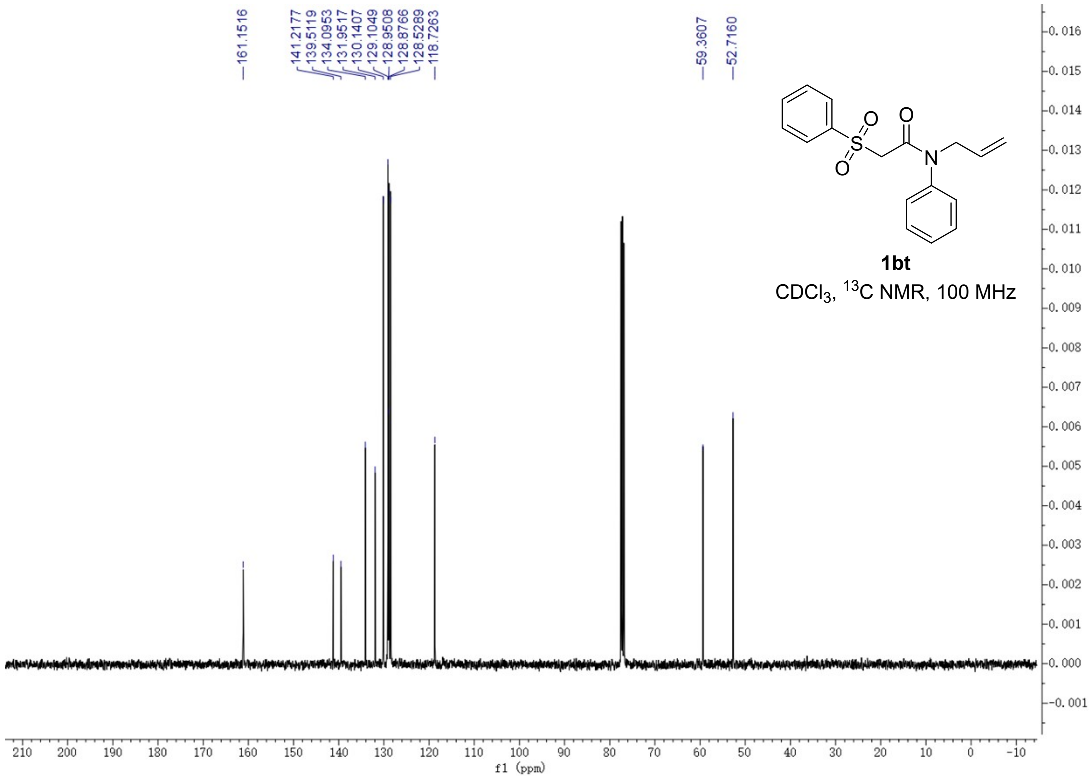

1bt

$CDCl_3$, ^{13}C NMR, 100 MHz

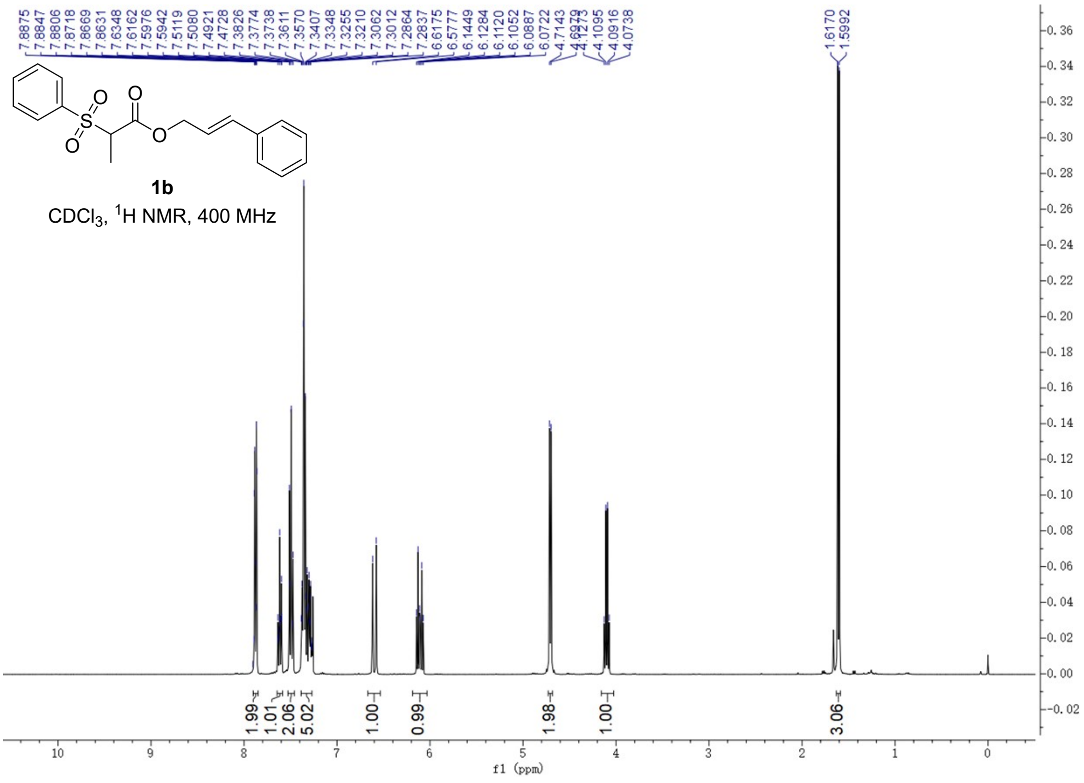
7.8875
7.8847
7.8806
7.8718
7.8669
7.8631
7.6348
7.6162
7.5976
7.5942
7.5119
7.5080
7.4921
7.4728
7.3826
7.3774
7.3738
7.3611
7.3570
7.3407
7.3348
7.3255
7.3210
7.3062
7.3012
7.2864
7.2837
6.6175
6.5777
6.1449
6.1284
6.1120
6.1052
6.0887
6.0722
4.7143
4.1095
4.0916
4.0738
1.6170
1.5992
1b
CDCl3, 1H NMR, 400 MHz
1.99
1.01
2.06
5.02
1.00
0.99
1.98
1.00
3.06
f1 (ppm)

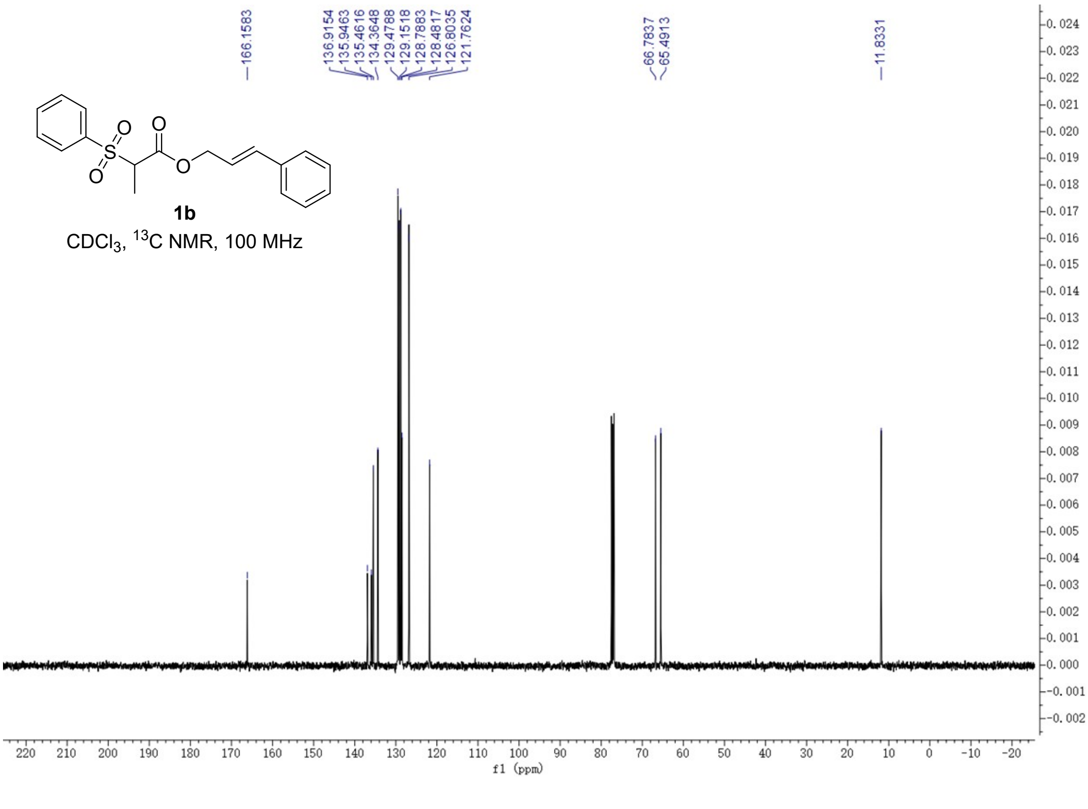
166.1583
136.9154
135.9463
135.4616
134.3648
129.4788
129.1518
128.7883
128.4817
126.8035
121.7624
66.7837
65.4913
11.8331
1b
CDCl3, 13C NMR, 100 MHz
f1 (ppm)

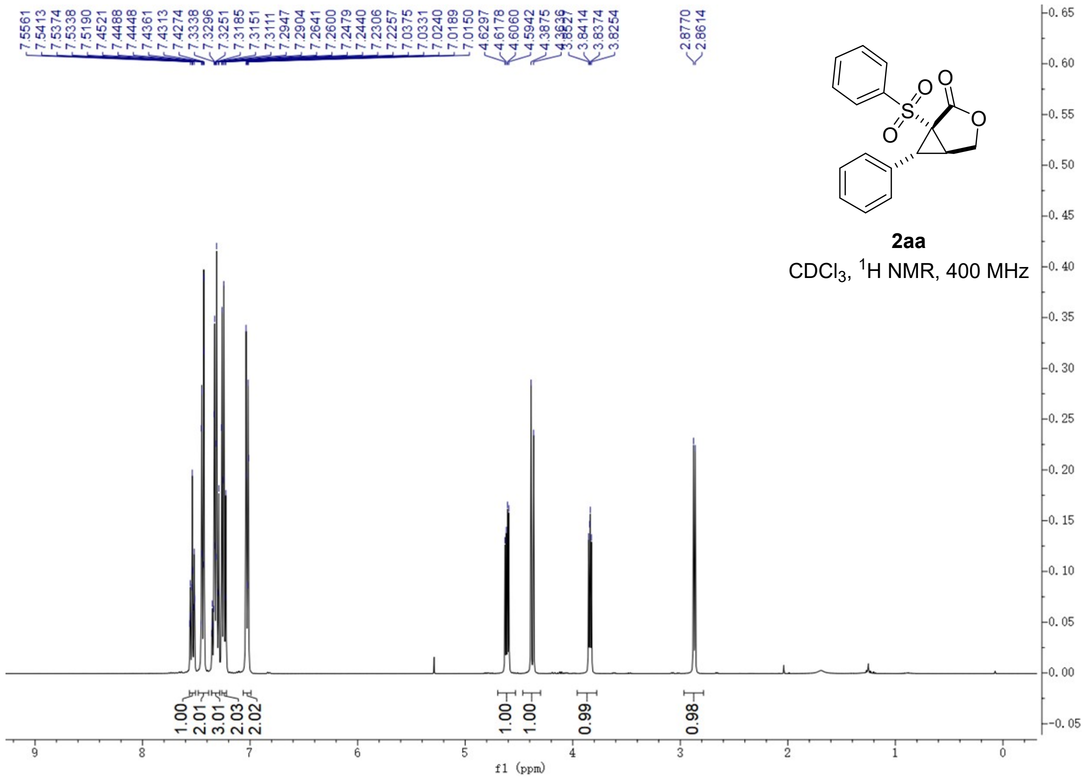
2aa
CDCl3, 1H NMR, 400 MHz
f1 (ppm)

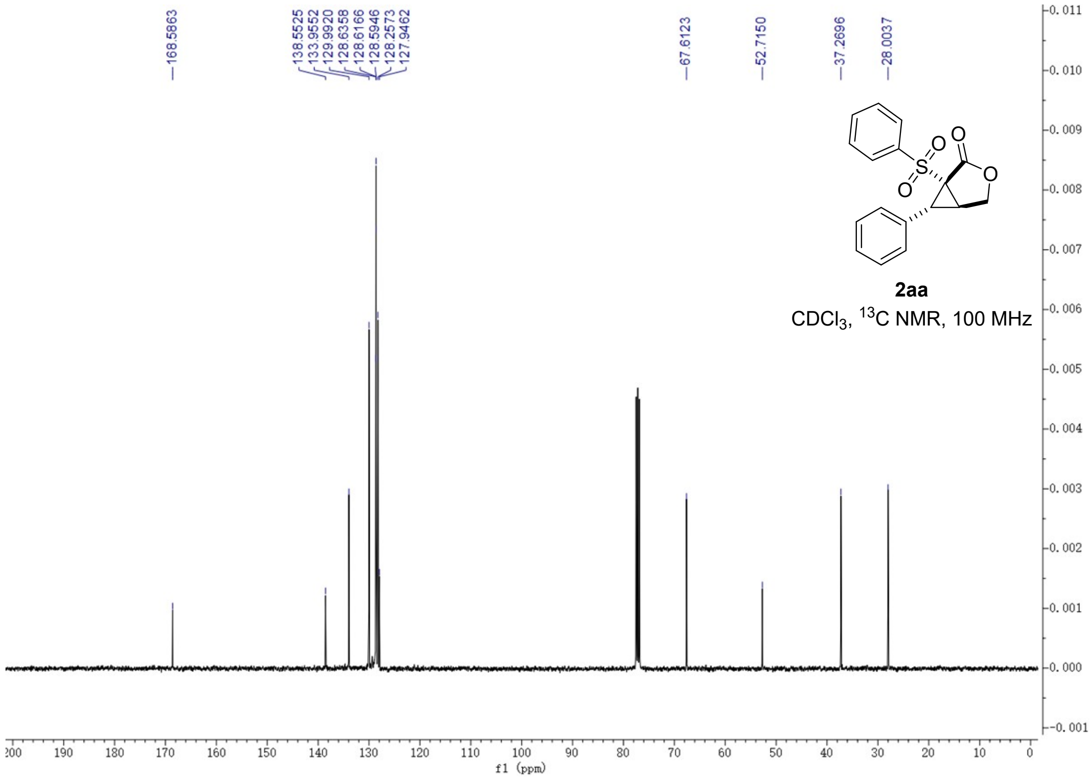
2aa
CDCl3, 13C NMR, 100 MHz
f1 (ppm)

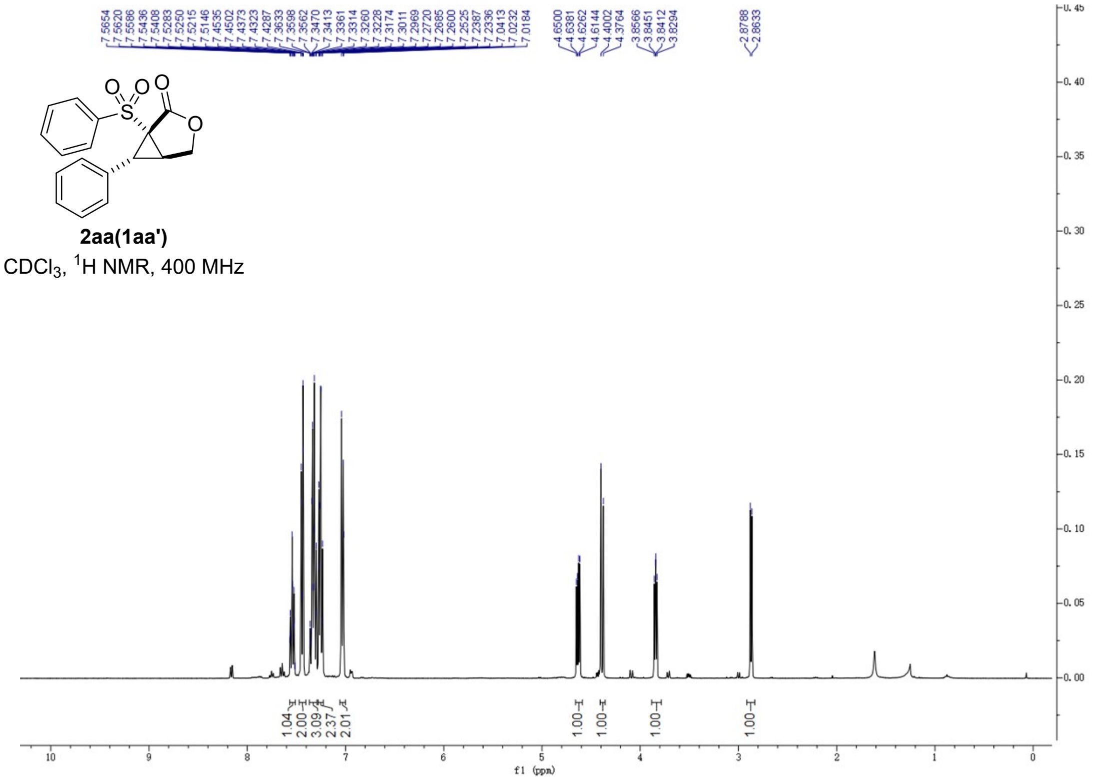
7.5654 7.5620 7.5586 7.5436 7.5408 7.5283 7.5250 7.5215 7.5146 7.4535 7.4502 7.4373 7.4323 7.4287 7.3633 7.3598 7.3562 7.3470 7.3413 7.3361 7.3314 7.3260 7.3228 7.3174 7.3011 7.2969 7.2720 7.2685 7.2600 7.2525 7.2387 7.2336 7.0413 7.0232 7.0184
4.6500 4.6381 4.6262 4.6144 4.4002 4.3764 3.8566 3.8451 3.8412 3.8294
2.8788 2.8633
1.04 2.00 3.09 2.37 2.01
1.00 1.00 1.00 1.00
f1 (ppm)
2aa(1aa')
CDCl3, 1H NMR, 400 MHz

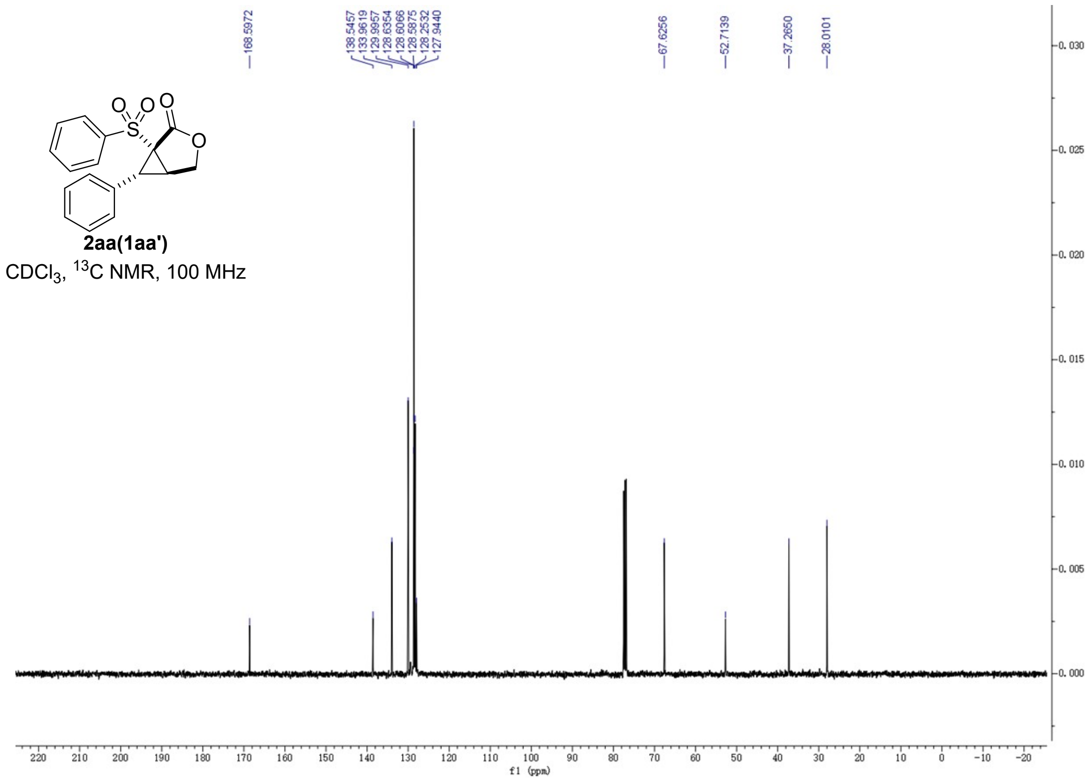
168.5972
138.5457 133.9619 129.9957 128.6354 128.6066 128.5875 128.2532 127.9440
67.6256
52.7139
37.2650
28.0101
f1 (ppm)
2aa(1aa')
CDCl3, 13C NMR, 100 MHz

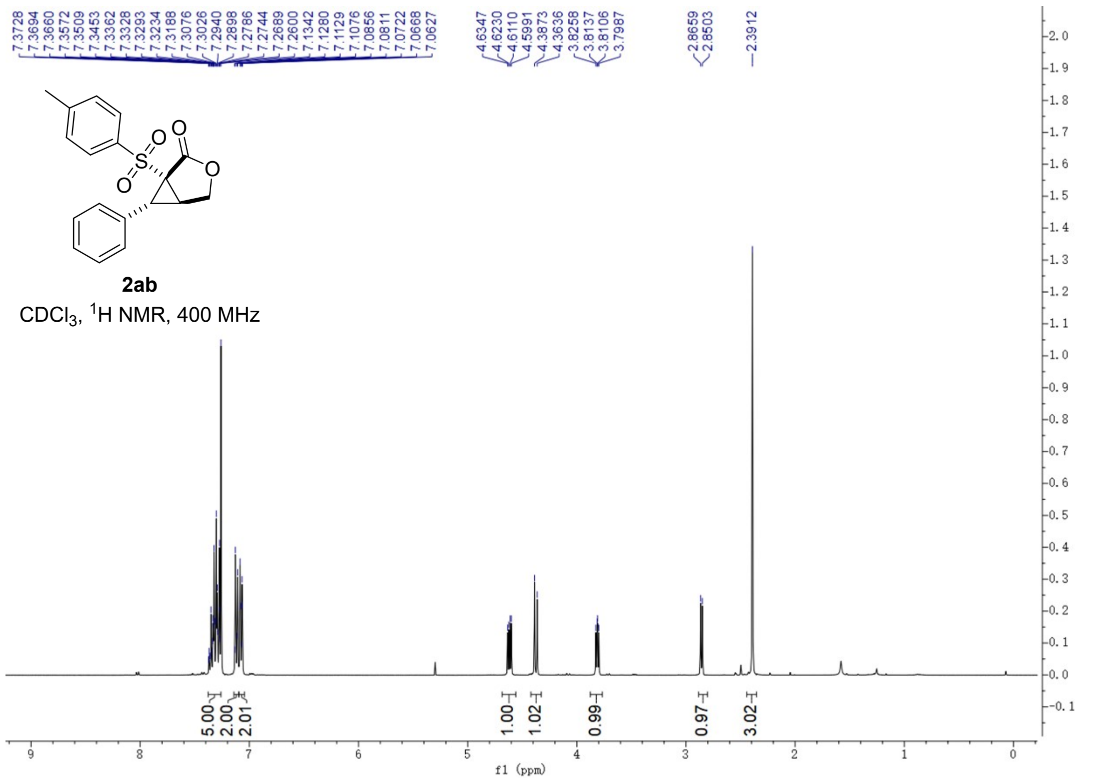

2ab

$CDCl_3$, ^1H NMR, 400 MHz

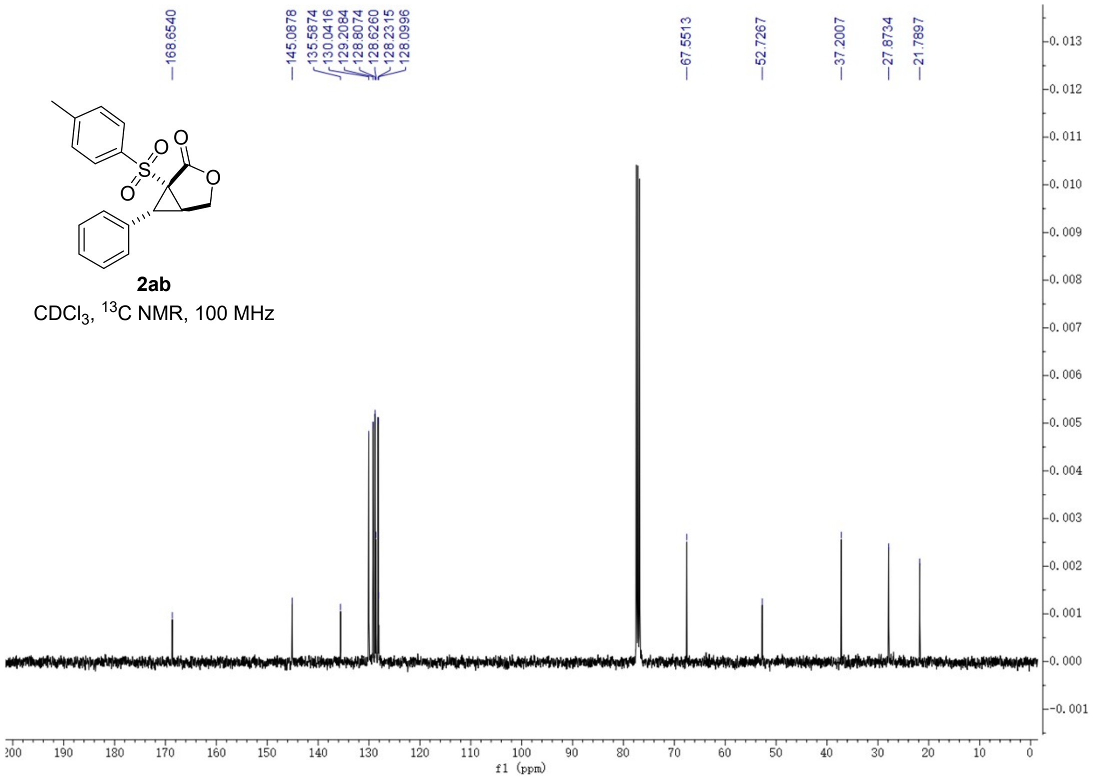

2ab

$CDCl_3$, ^{13}C NMR, 100 MHz

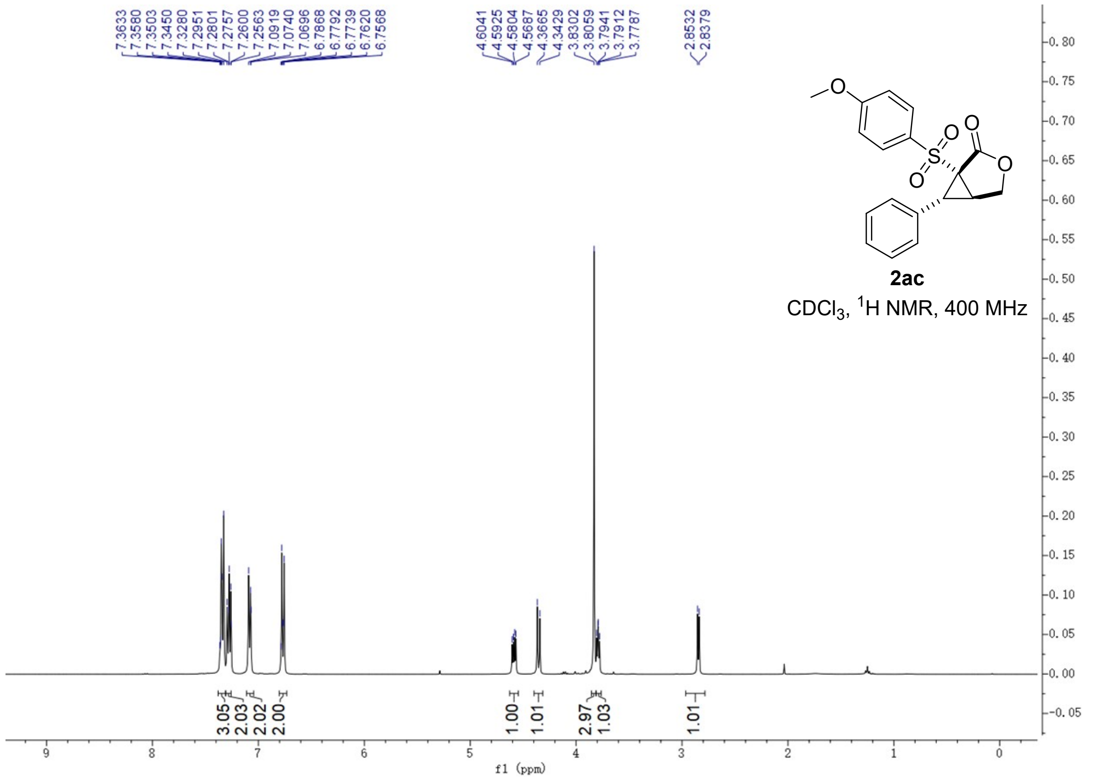
7.3633
7.3580
7.3503
7.3450
7.3280
7.2951
7.2801
7.2757
7.2600
7.2563
7.0919
7.0740
7.0696
6.7868
6.7792
6.7739
6.7620
6.7568
4.6041
4.5925
4.5804
4.5687
4.3665
4.3429
3.8302
3.8059
3.7941
3.7912
3.7787
2.8532
2.8379
2ac
CDCl3, 1H NMR, 400 MHz
3.05
2.03
2.02
2.00
1.00
1.01
2.97
1.03
1.01
f1 (ppm)

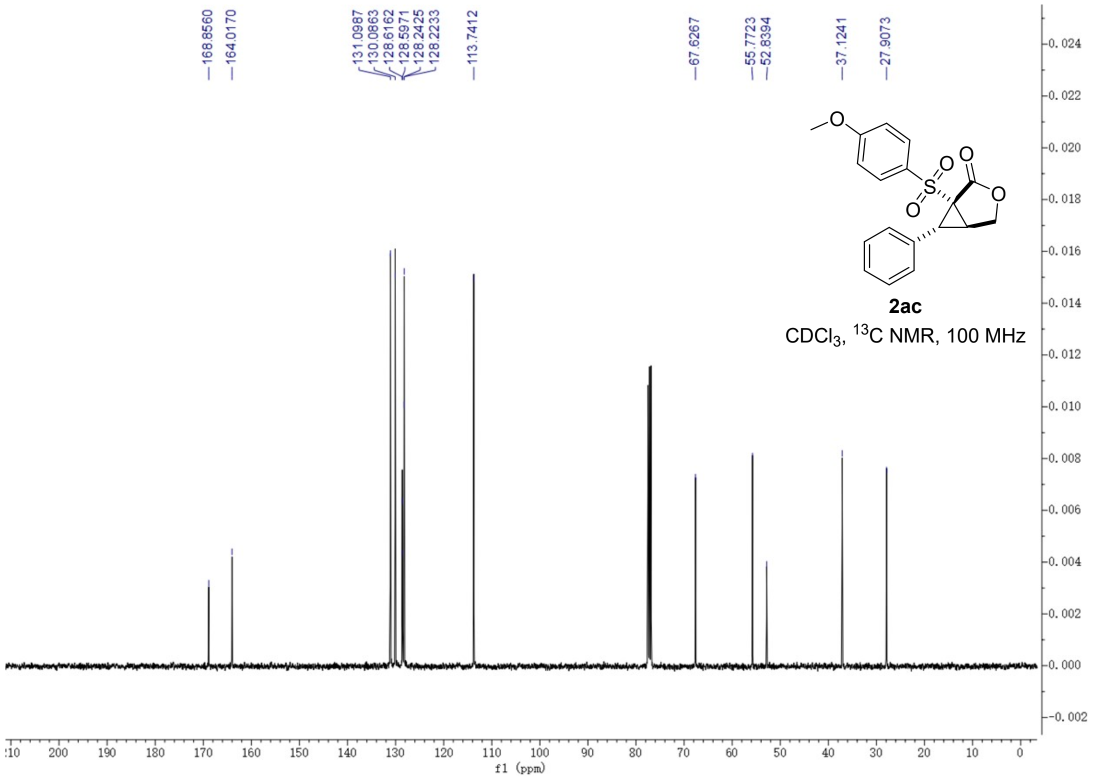
168.8560
164.0170
131.0987
130.0863
128.6162
128.5971
128.2425
128.2233
113.7412
67.6267
55.7723
52.8394
37.1241
27.9073
2ac
CDCl3, 13C NMR, 100 MHz
f1 (ppm)

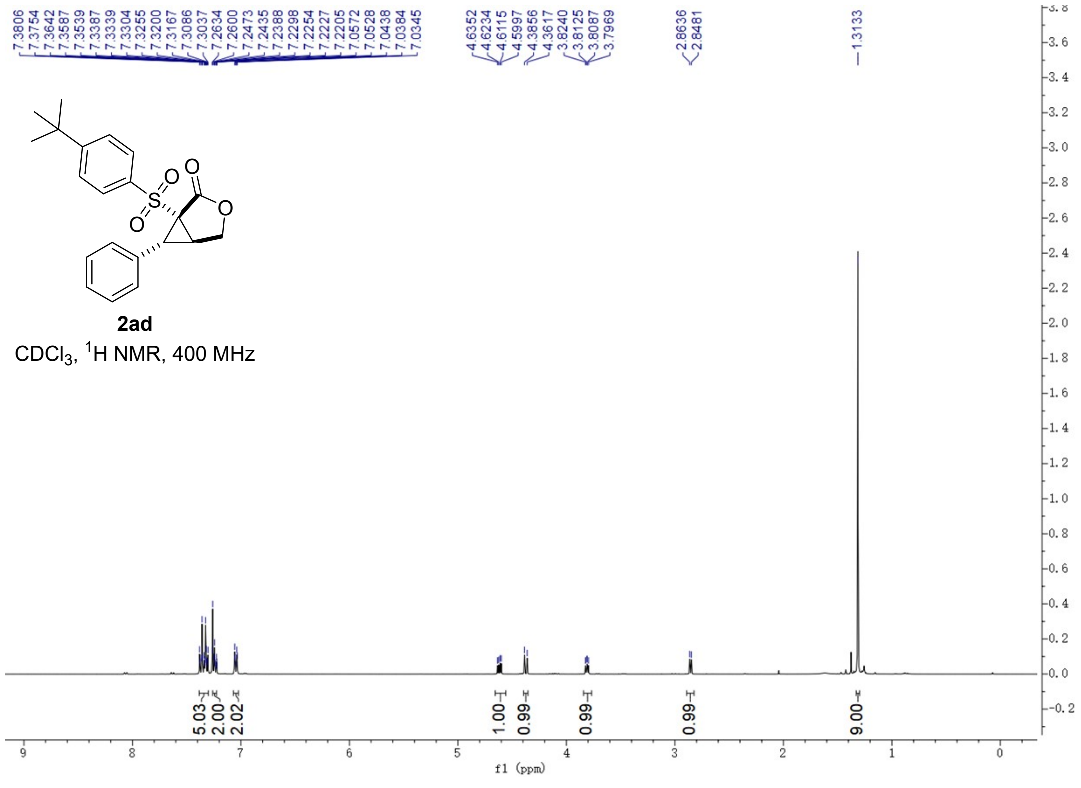

2ad

$CDCl_3$, ^1H NMR, 400 MHz

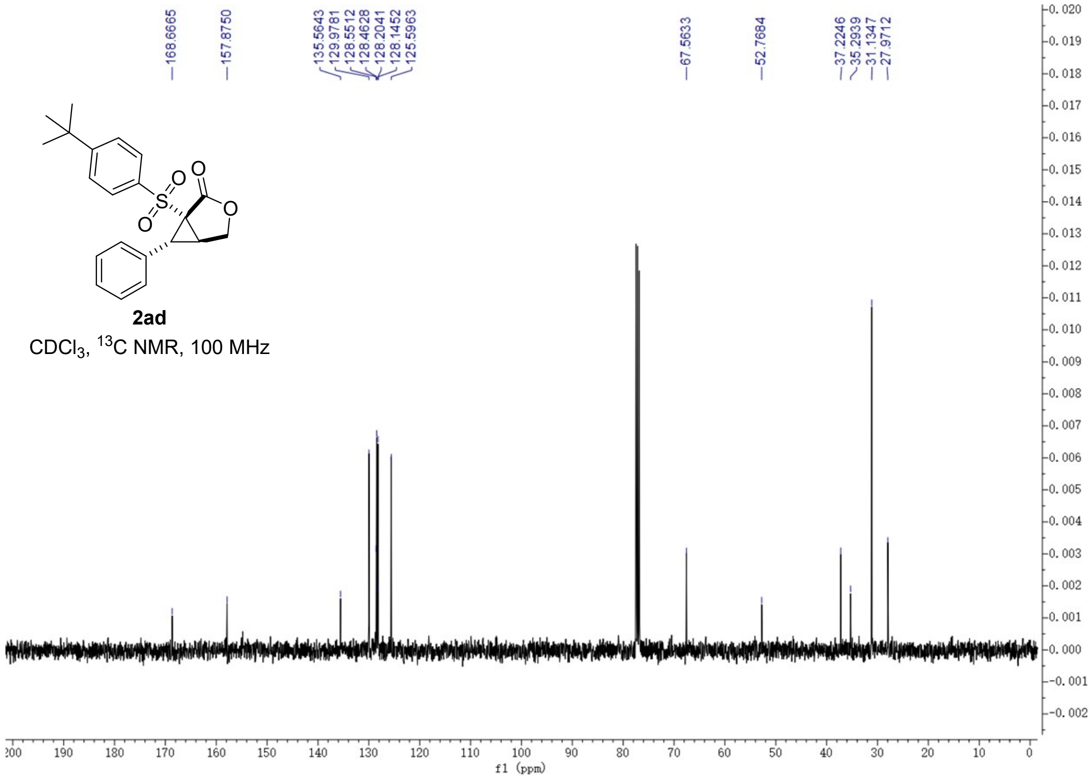

2ad

$CDCl_3$, ^{13}C NMR, 100 MHz

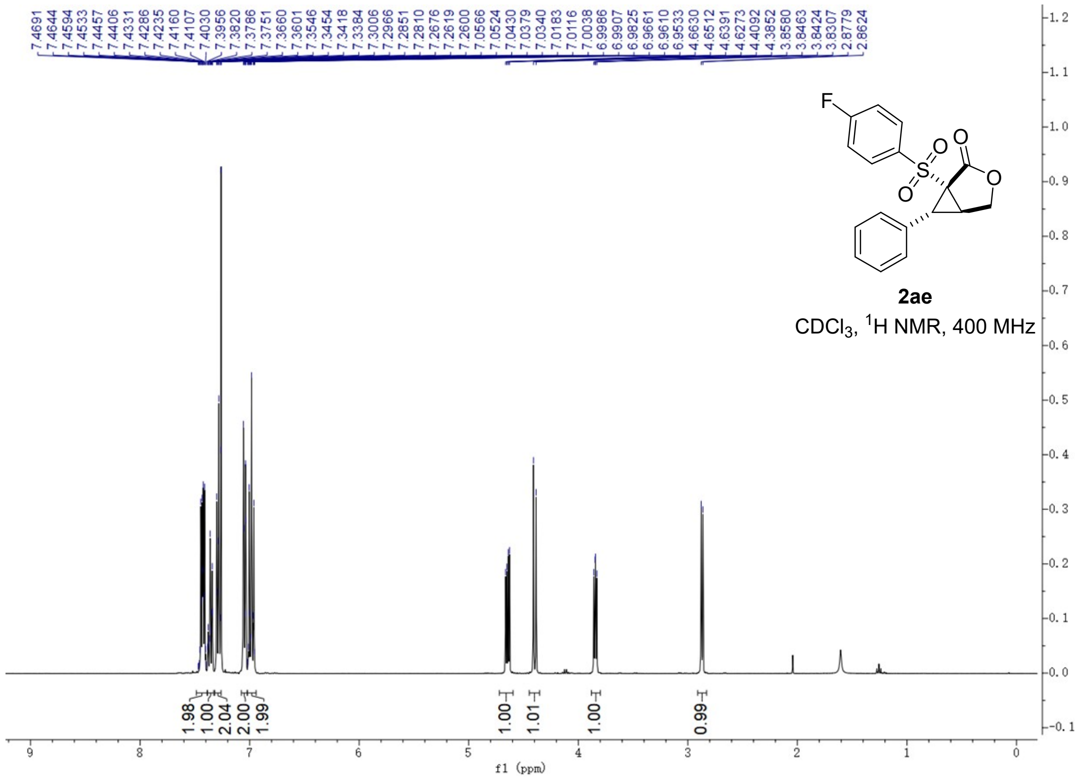

2ae

$CDCl_3$, ^1H NMR, 400 MHz

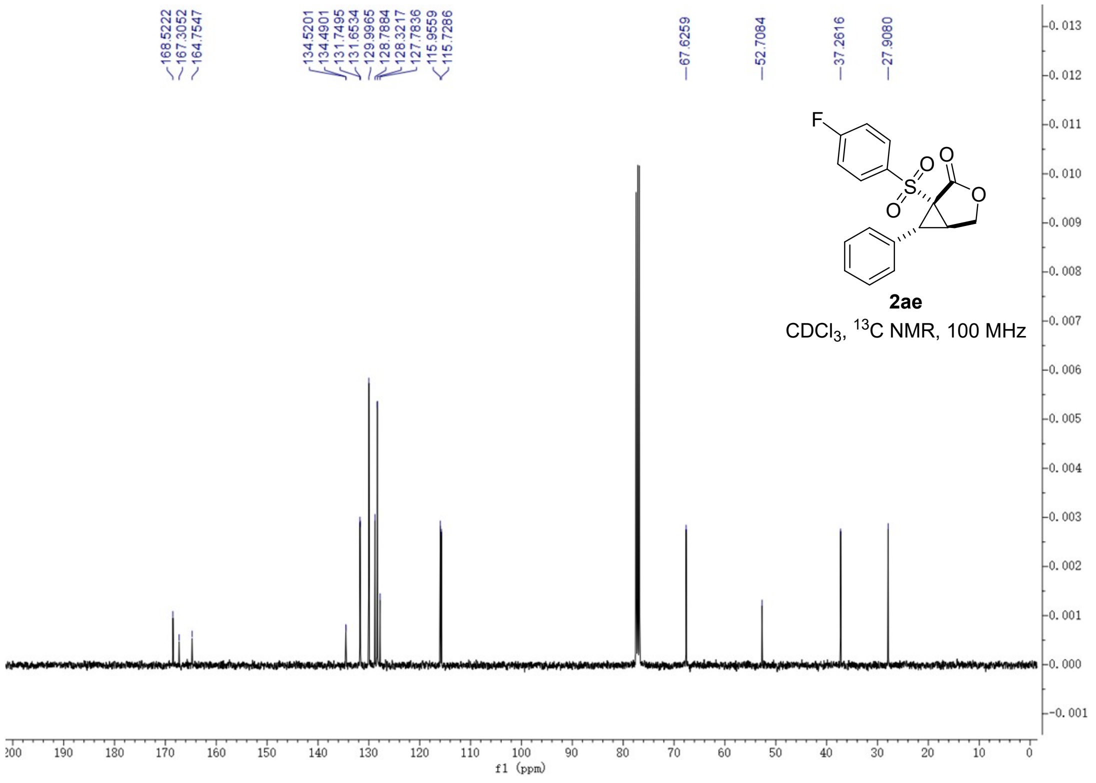

2ae

$CDCl_3$, ^{13}C NMR, 100 MHz

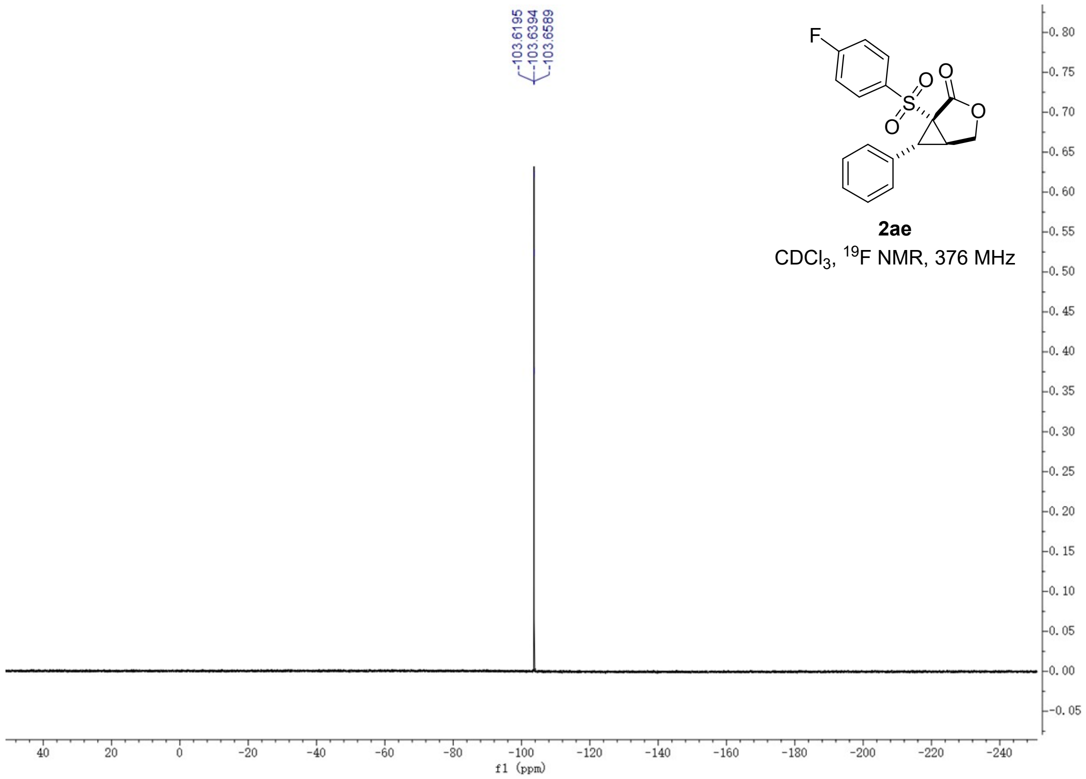
-103.6195
-103.6394
-103.6589
2ae
CDCl$_3$, ^{19}F NMR, 376 MHz
f1 (ppm)

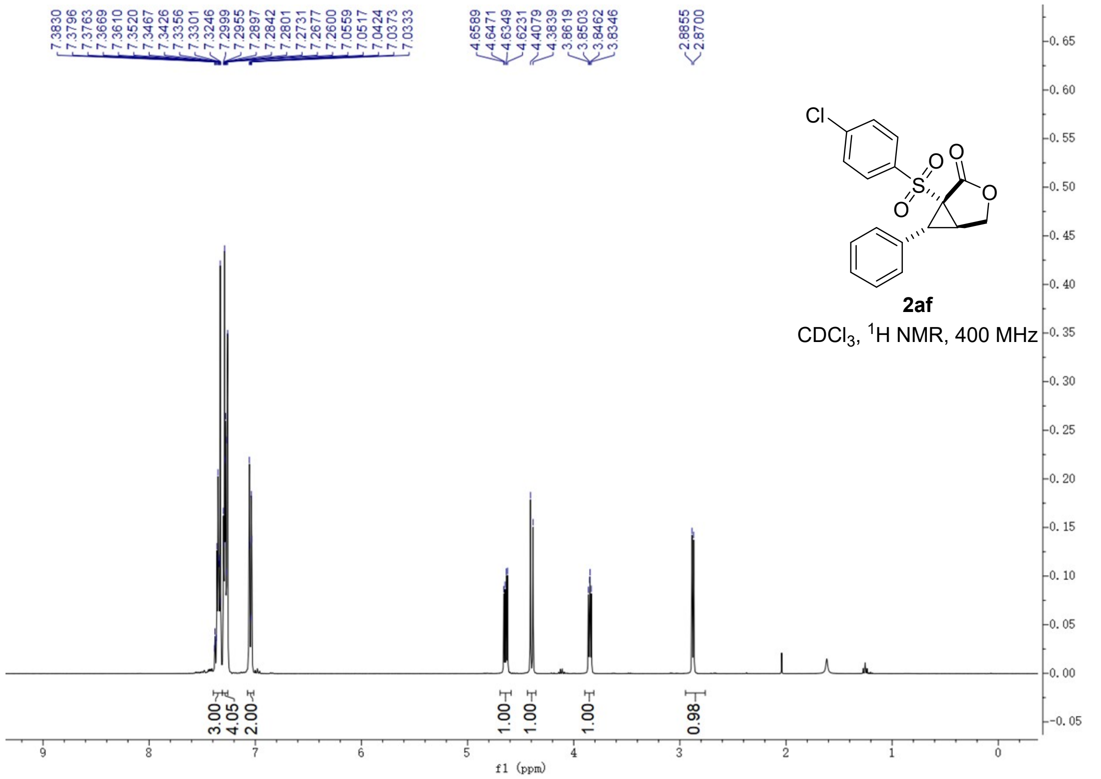
7.3830 7.3796 7.3763 7.3669 7.3610 7.3520 7.3467 7.3426 7.3356 7.3301 7.3246 7.2999 7.2955 7.2897 7.2842 7.2801 7.2731 7.2677 7.2600 7.0559 7.0517 7.0424 7.0373 7.0333
4.6589 4.6471 4.6349 4.6231 4.4079 4.3839 3.8619 3.8503 3.8462 3.8346
2.8855 2.8700
3.00 4.05 2.00 1.00 1.00 1.00 0.98
f1 (ppm)
2af
CDCl3, 1H NMR, 400 MHz

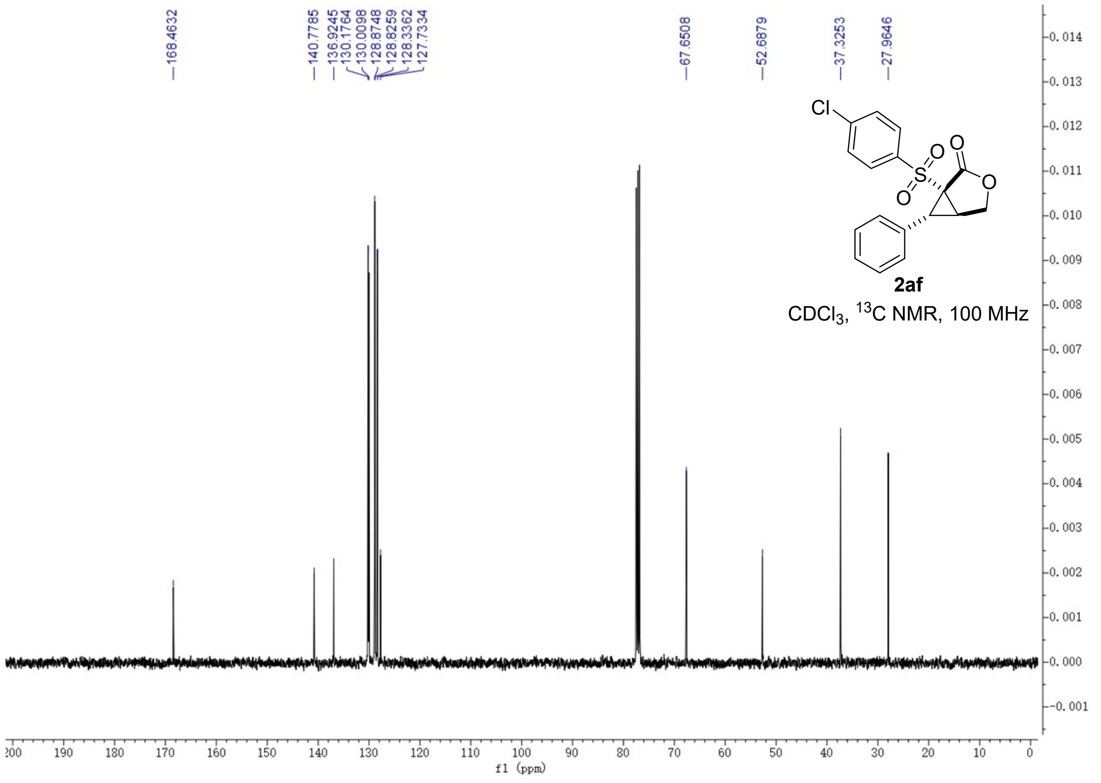
168.4632
140.7785 136.9245 130.1764 130.0098 128.8748 128.8259 128.3362 127.7334
67.6508
52.6879
37.3253
27.9646
f1 (ppm)
2af
CDCl3, 13C NMR, 100 MHz

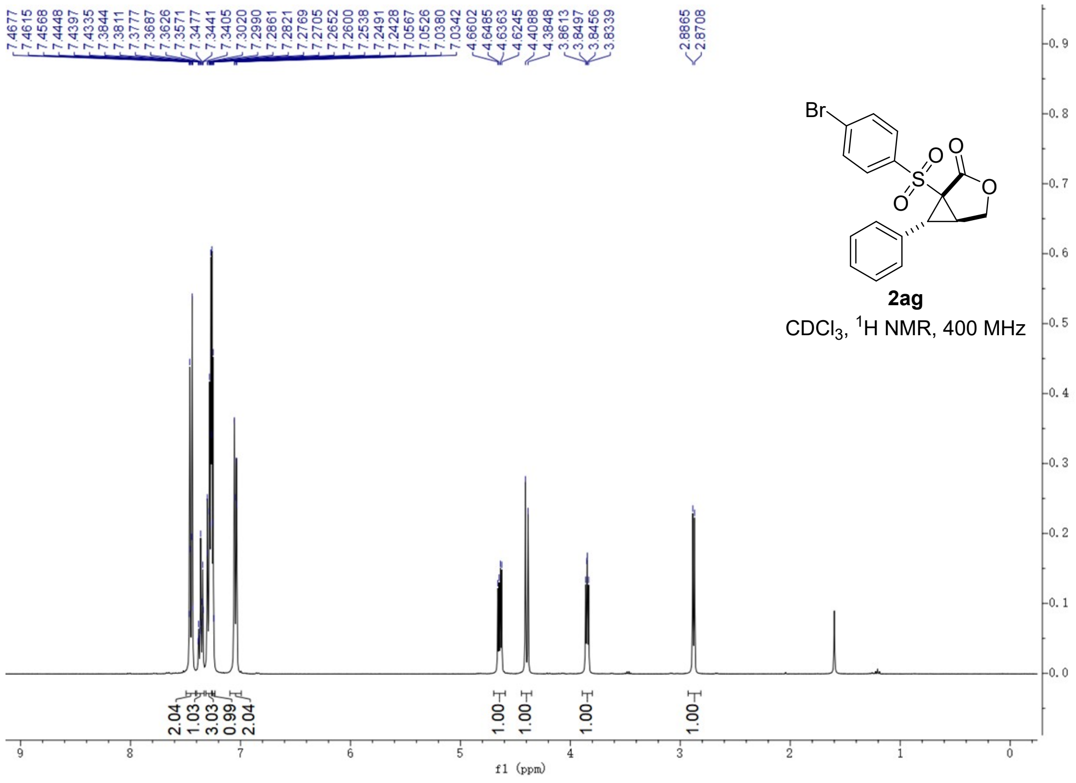

2ag

$CDCl_3$, ^1H NMR, 400 MHz

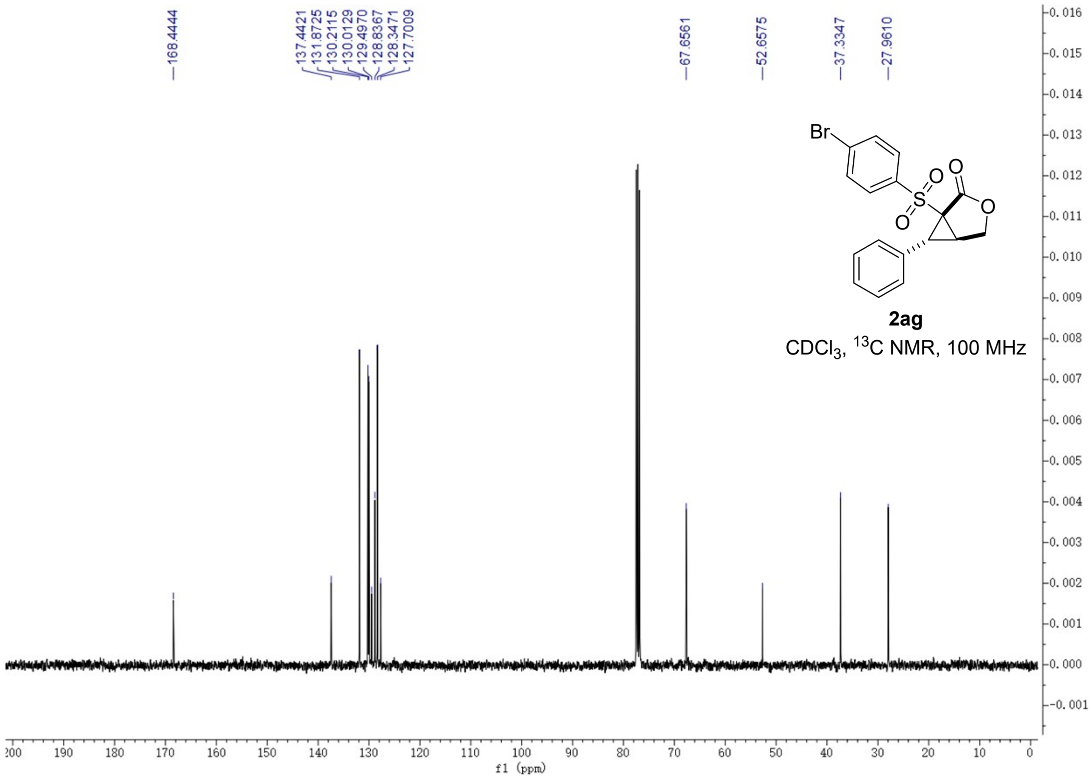

2ag

$CDCl_3$, ^{13}C NMR, 100 MHz

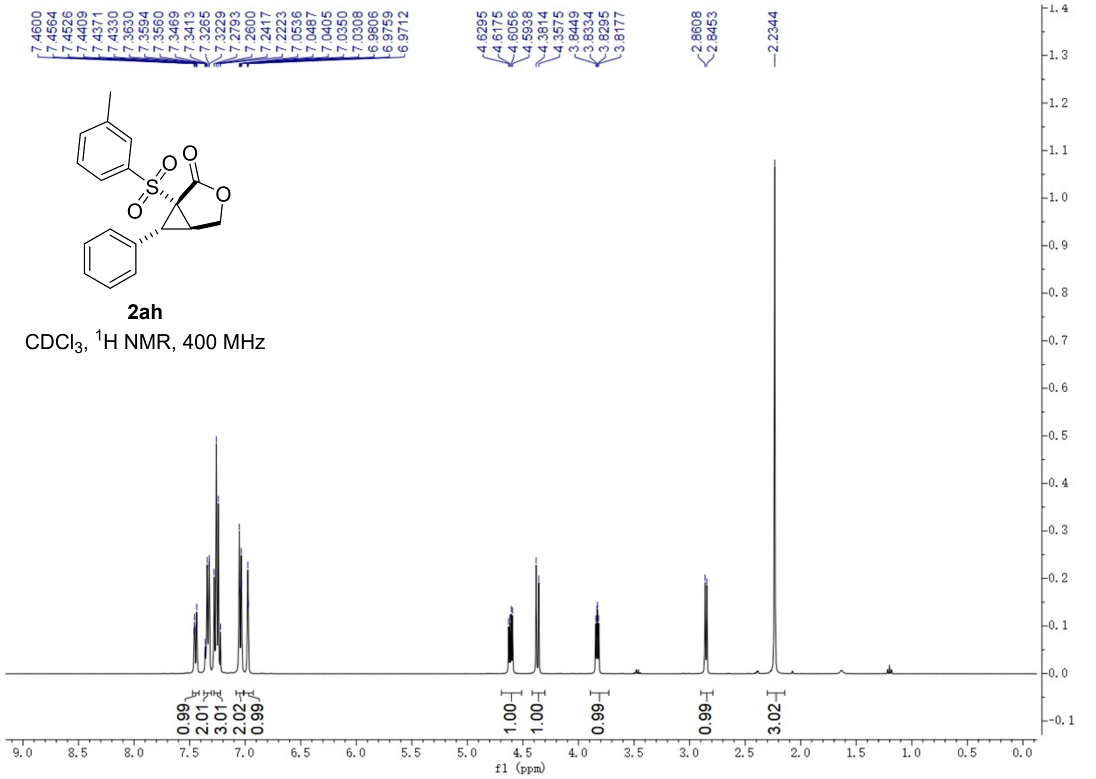
2ah
CDCl3, 1H NMR, 400 MHz
f1 (ppm)

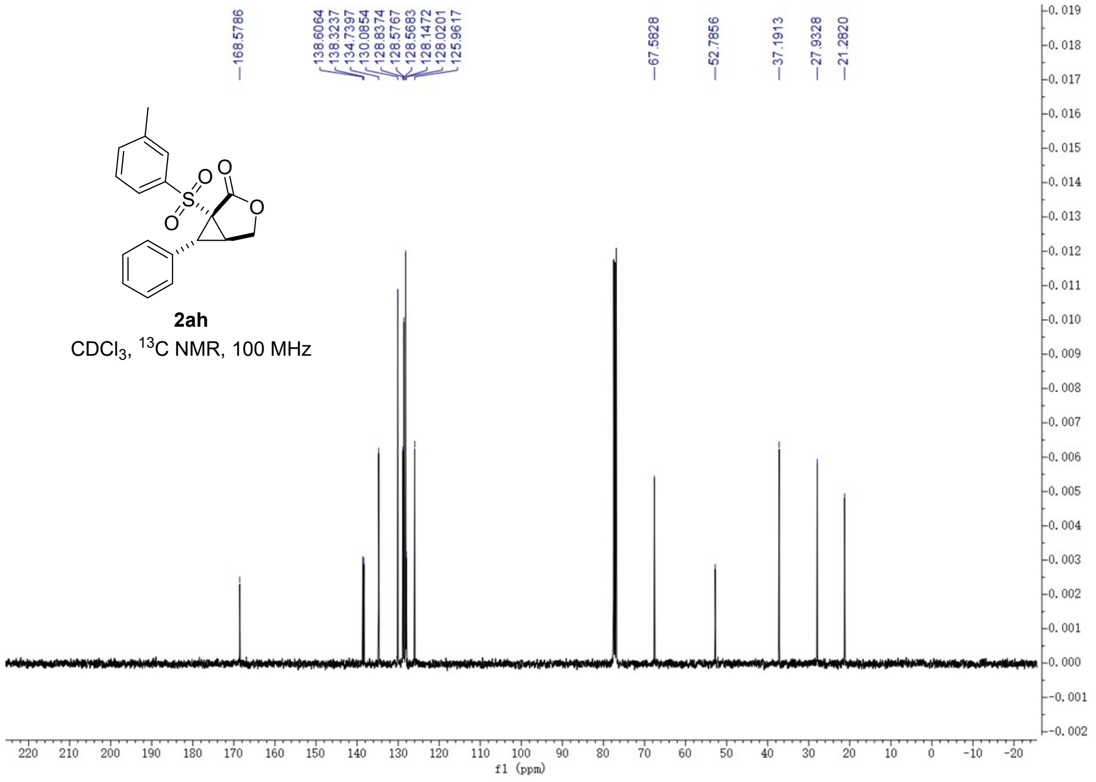
2ah
CDCl3, 13C NMR, 100 MHz
f1 (ppm)

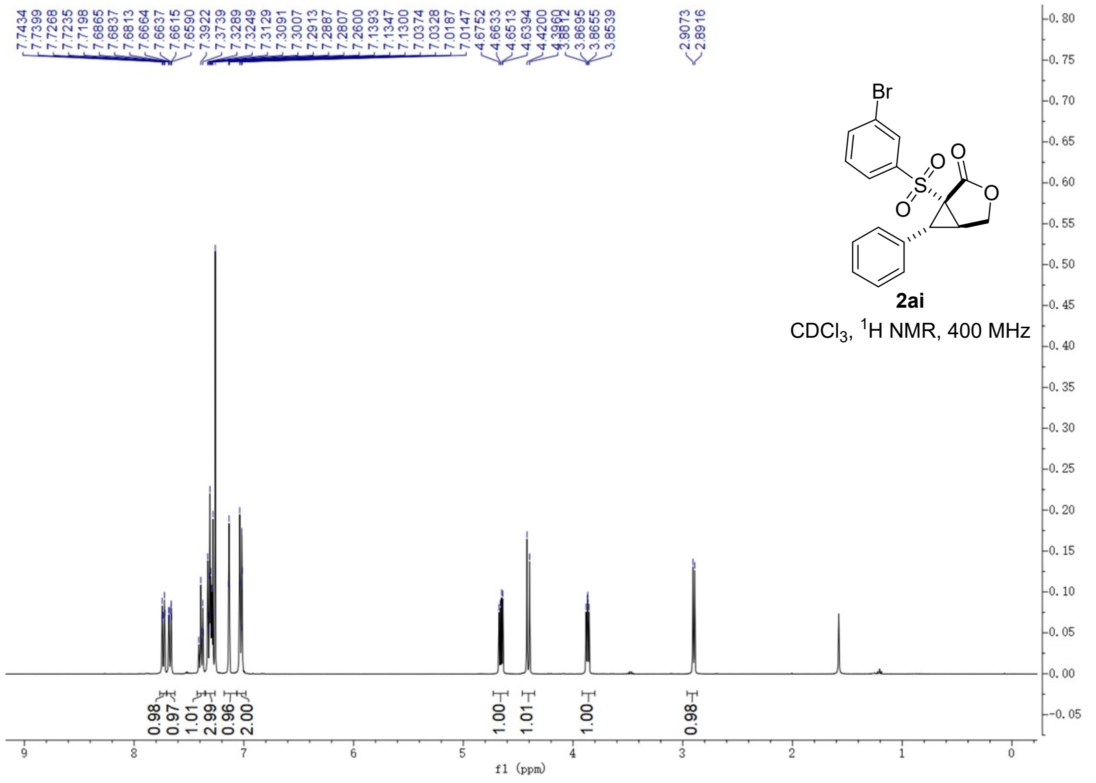
7.7434
7.7399
7.7268
7.7235
7.7198
7.6865
7.6837
7.6813
7.6664
7.6637
7.6615
7.6590
7.3922
7.3739
7.3289
7.3249
7.3129
7.3091
7.3007
7.2913
7.2887
7.2807
7.2600
7.1393
7.1347
7.1300
7.0374
7.0328
7.0187
7.0147
4.6752
4.6633
4.6513
4.6394
4.4200
4.3960
3.8812
3.8695
3.8655
3.8539
2.9073
2.8916
Br
O
S
O
O
O
2ai
CDCl3, 1H NMR, 400 MHz
0.98
0.97
1.01
2.99
0.96
2.00
1.00
1.01
1.00
0.98
f1 (ppm)

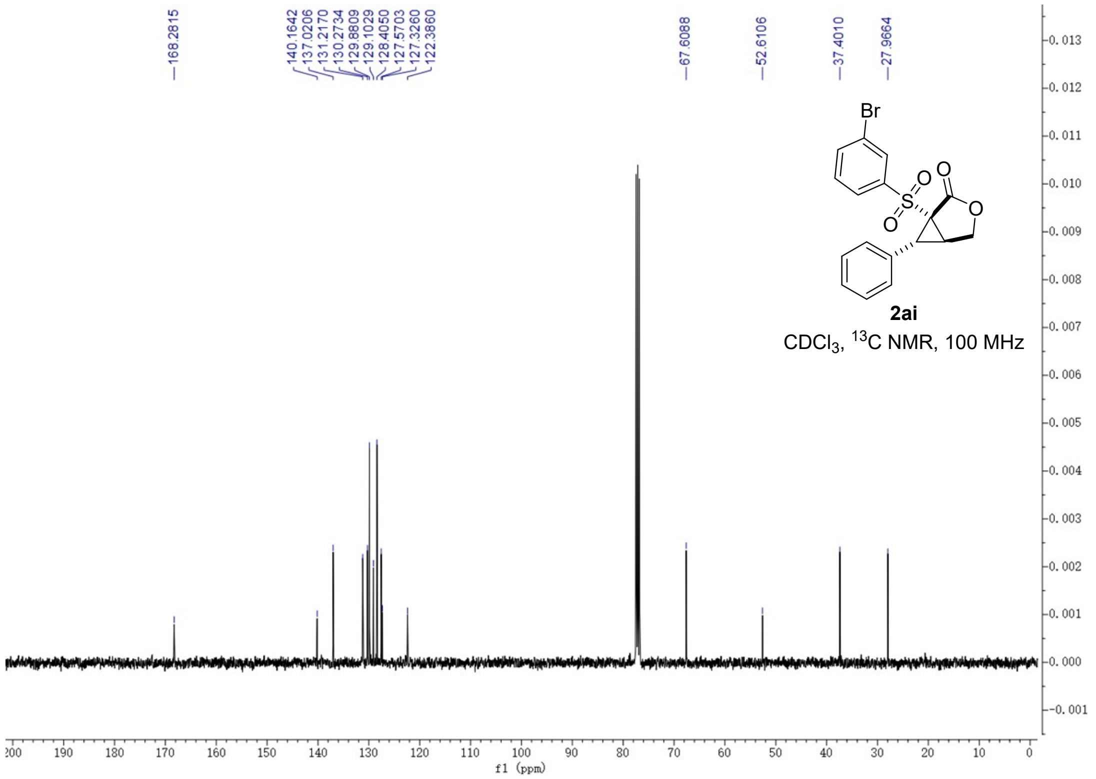
168.2815
140.1642
137.0206
131.2170
130.2734
129.8809
129.1029
128.4050
127.5703
127.3260
122.3860
67.6088
52.6106
37.4010
27.9664
Br
O
S
O
O
O
2ai
CDCl3, 13C NMR, 100 MHz
f1 (ppm)

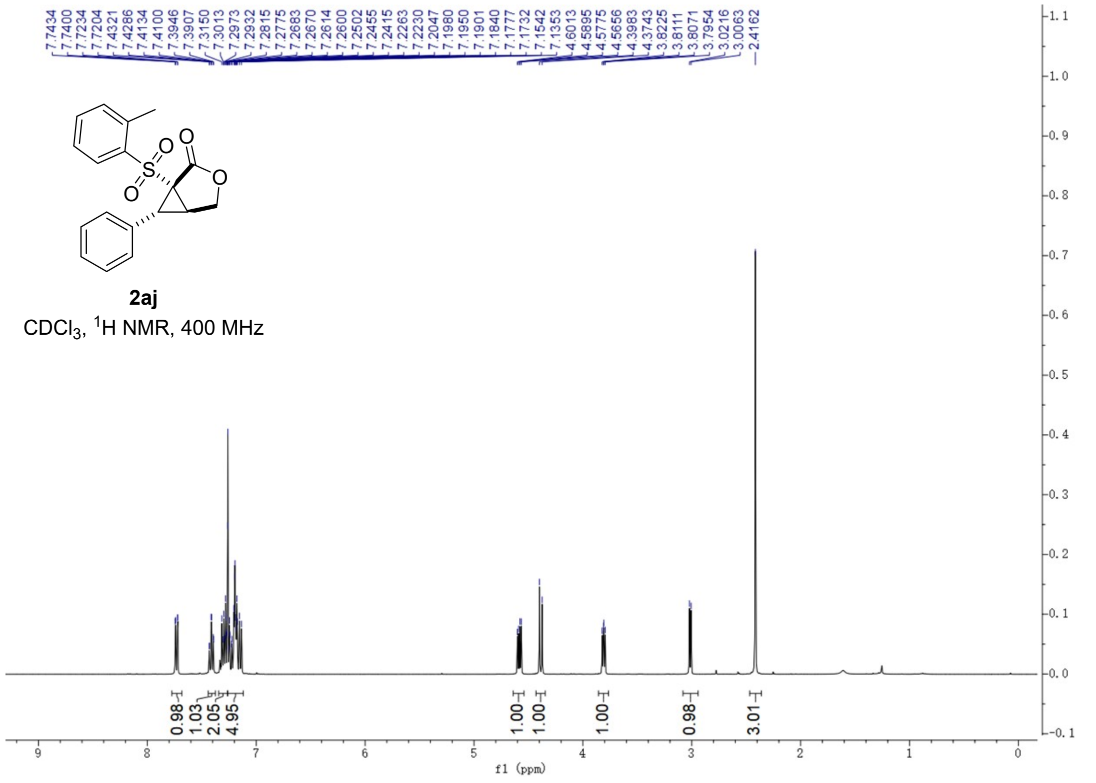
7.7434
7.7400
7.7234
7.7204
7.4321
7.4286
7.4134
7.4100
7.3946
7.3907
7.3150
7.3013
7.2973
7.2932
7.2815
7.2775
7.2683
7.2670
7.2614
7.2600
7.2502
7.2455
7.2415
7.2263
7.2230
7.2047
7.1980
7.1950
7.1901
7.1840
7.1777
7.1732
7.1542
7.1353
4.6013
4.5895
4.5775
4.5656
4.3983
4.3743
3.8225
3.8111
3.8071
3.7954
3.0216
3.0063
2.4162
2aj
CDCl3, 1H NMR, 400 MHz
0.98
1.03
2.05
4.95
1.00
1.00
1.00
0.98
3.01
f1 (ppm)

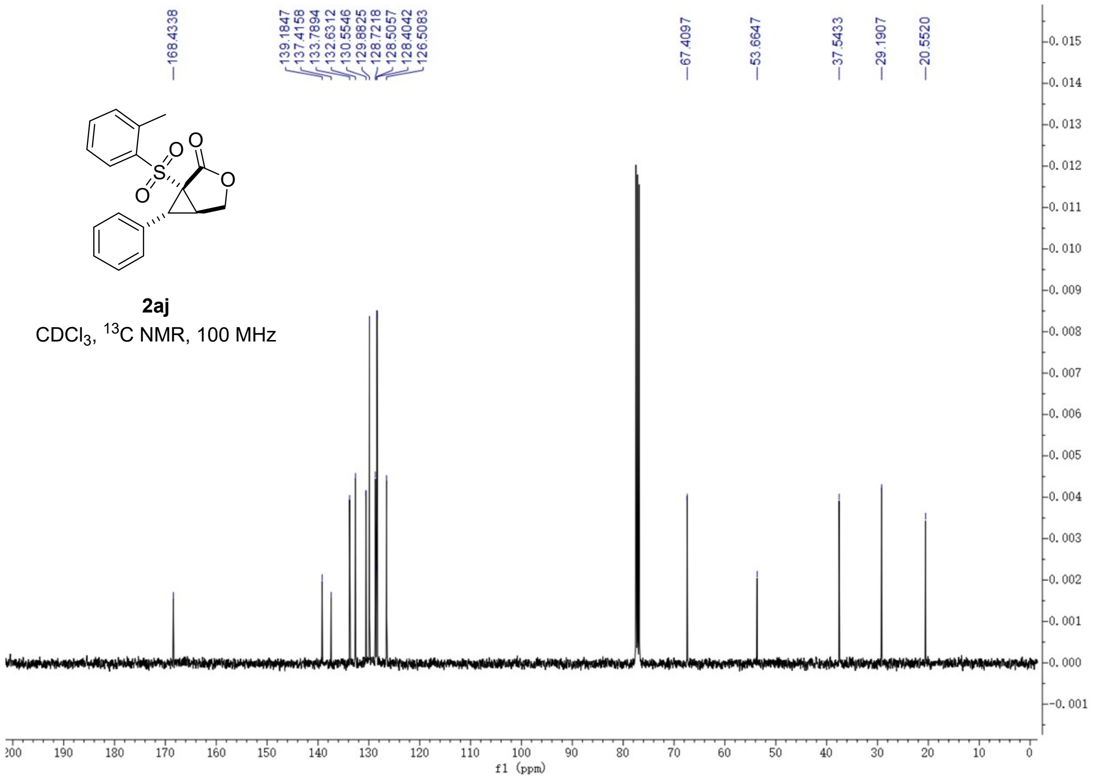
168.4338
139.1847
137.4158
133.7894
132.6312
130.5546
129.8825
128.7218
128.5057
128.4042
126.5083
67.4097
53.6647
37.5433
29.1907
20.5520
2aj
CDCl3, 13C NMR, 100 MHz
f1 (ppm)

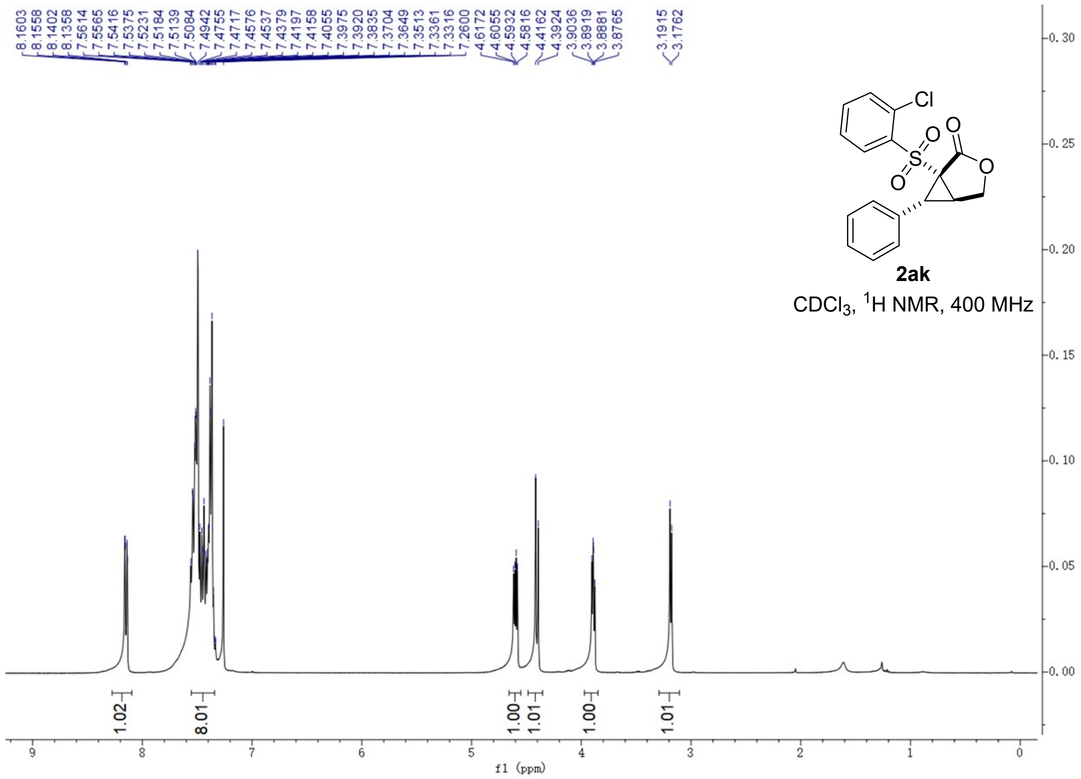
8.1603
8.1558
8.1402
8.1358
7.5614
7.5565
7.5416
7.5375
7.5231
7.5184
7.5139
7.5084
7.4942
7.4755
7.4717
7.4576
7.4537
7.4379
7.4197
7.4158
7.4055
7.3975
7.3920
7.3835
7.3704
7.3649
7.3513
7.3361
7.3316
7.2600
4.6172
4.6055
4.5932
4.5816
4.4162
4.3924
3.9036
3.8919
3.8881
3.8765
3.1915
3.1762
Cl
O
S
O
O
O
2ak
CDCl3, 1H NMR, 400 MHz
1.02
8.01
1.00
1.01
1.00
1.01
f1 (ppm)

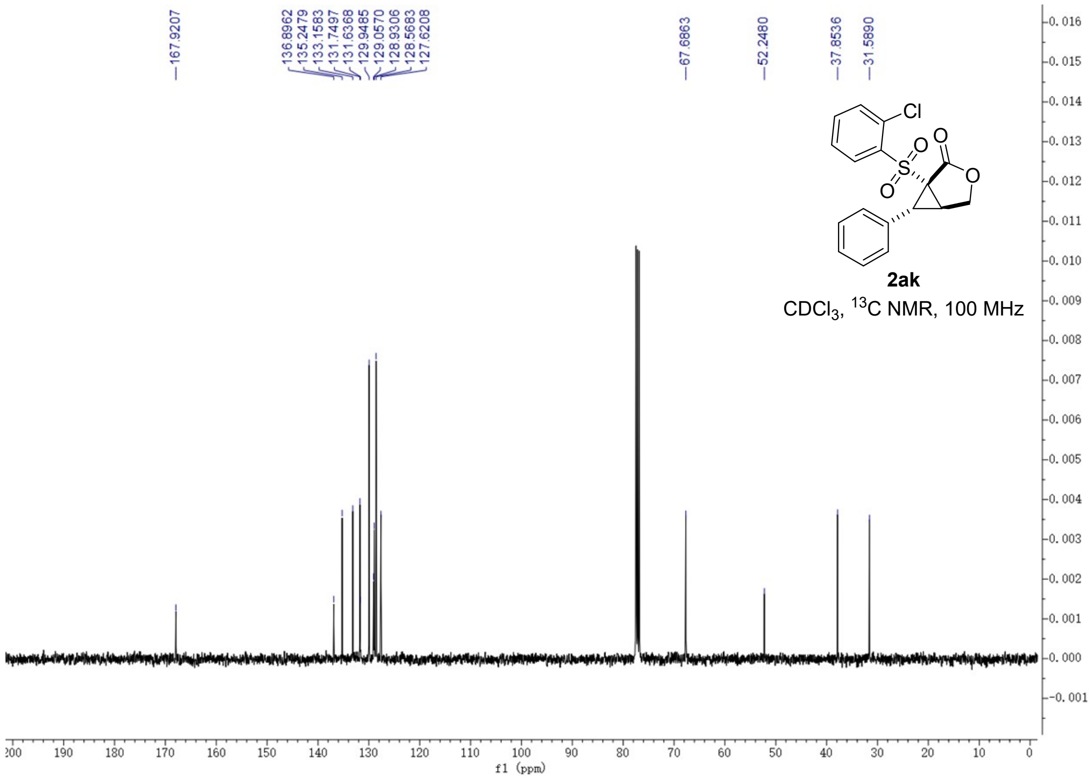
167.9207
136.8962
135.2479
133.1583
131.7497
131.6368
129.9485
129.0570
128.9306
128.5683
127.6208
67.6863
52.2480
37.8536
31.5890
Cl
O
S
O
O
O
2ak
CDCl3, 13C NMR, 100 MHz
f1 (ppm)

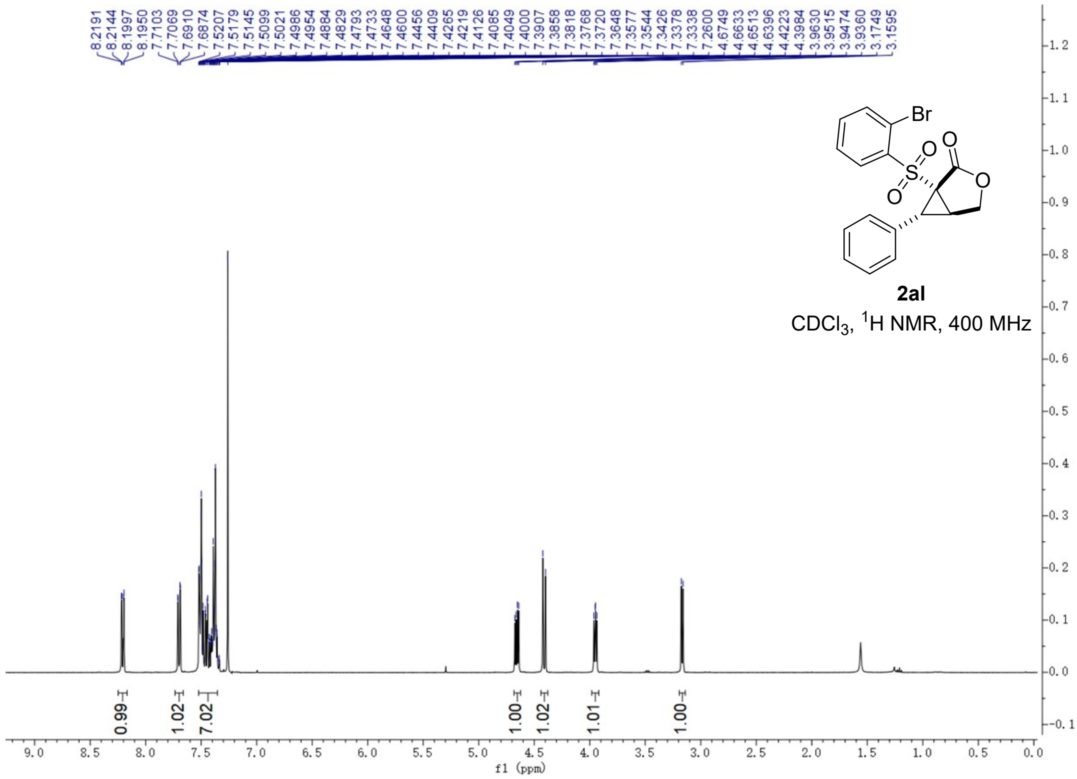
8.2191
8.2144
8.1997
8.1950
7.7103
7.7069
7.6910
7.6874
7.5207
7.5179
7.5145
7.5099
7.5021
7.4986
7.4954
7.4884
7.4829
7.4793
7.4733
7.4648
7.4600
7.4456
7.4409
7.4265
7.4219
7.4126
7.4085
7.4049
7.4000
7.3907
7.3858
7.3818
7.3768
7.3720
7.3648
7.3577
7.3544
7.3426
7.3378
7.3338
7.2600
4.6749
4.6633
4.6513
4.6396
4.4223
4.3984
3.9630
3.9515
3.9474
3.9360
3.1749
3.1595
Br
O
S
O
O
O
2al
CDCl3, 1H NMR, 400 MHz
0.99
1.02
7.02
1.00
1.02
1.01
1.00
f1 (ppm)

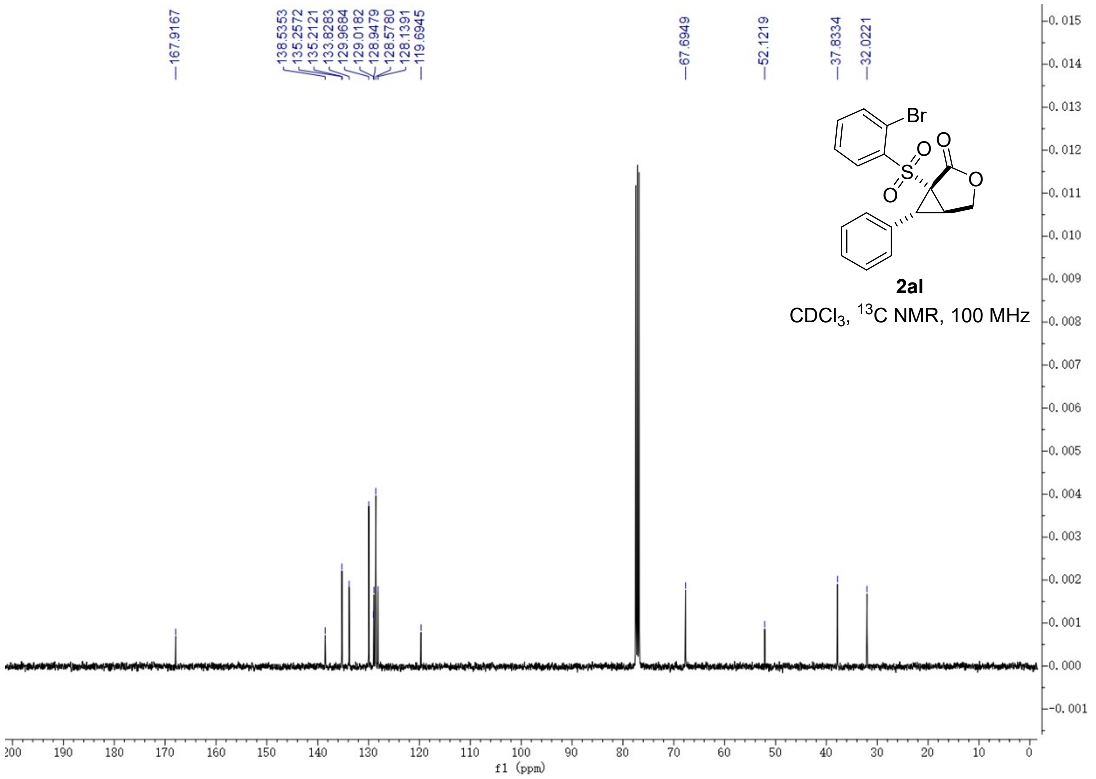
167.9167
138.5353
135.2572
135.2121
133.8283
129.9684
129.0182
128.9479
128.5780
128.1391
119.6945
67.6949
52.1219
37.8334
32.0221
Br
O
S
O
O
O
2al
CDCl3, 13C NMR, 100 MHz
f1 (ppm)

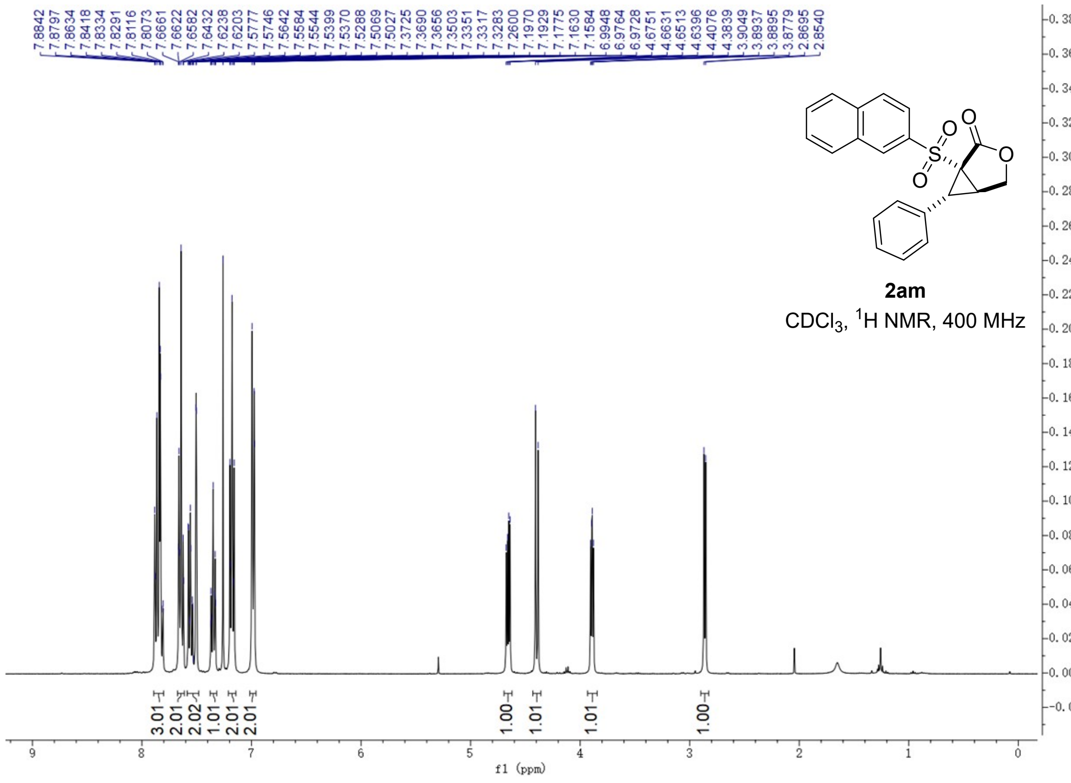
2am
$CDCl_3$, ^1H NMR, 400 MHz
f1 (ppm)

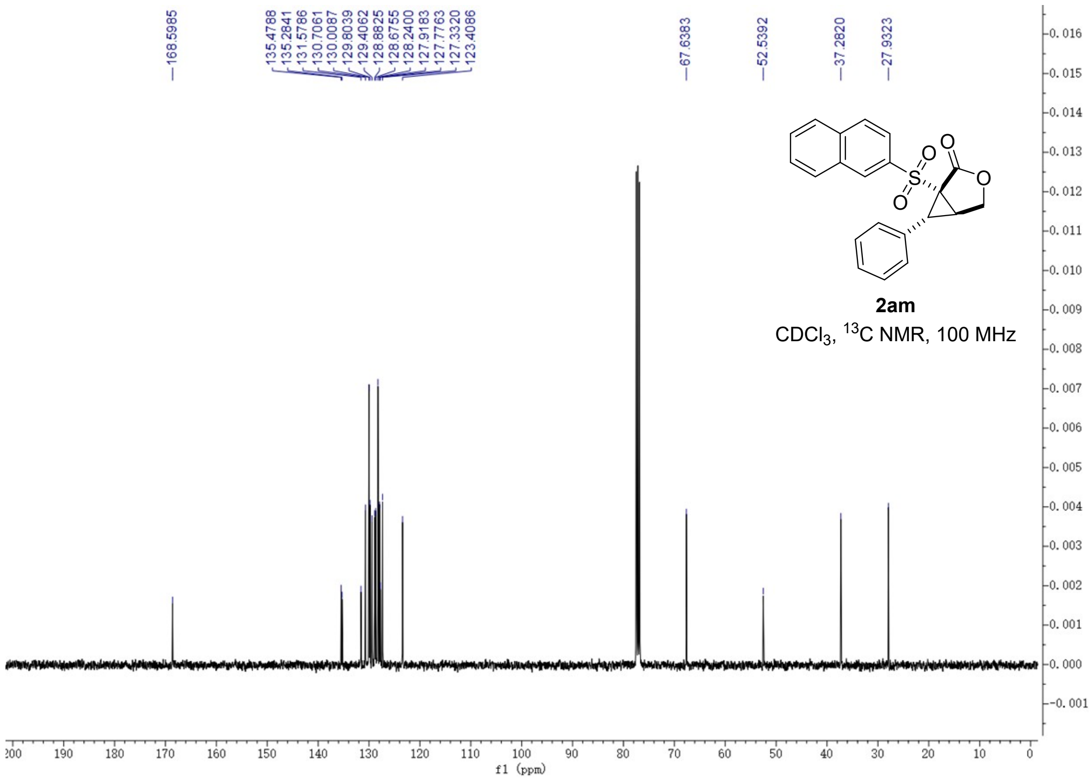
2am
$CDCl_3$, ^{13}C NMR, 100 MHz
f1 (ppm)

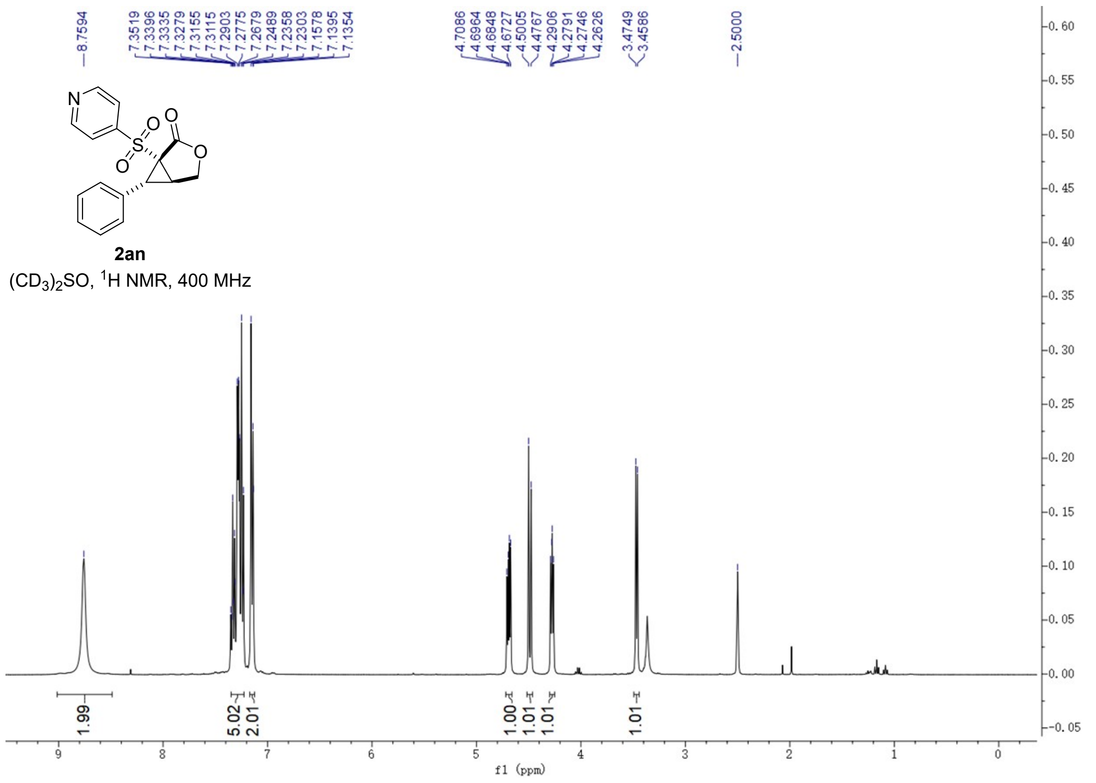
8.7594
7.3519
7.3396
7.3335
7.3279
7.3155
7.3115
7.2903
7.2775
7.2679
7.2489
7.2358
7.2303
7.1578
7.1395
7.1354
4.7086
4.6964
4.6848
4.6727
4.5005
4.4767
4.2906
4.2791
4.2746
4.2626
3.4749
3.4586
2.5000
2an
(CD3)2SO, 1H NMR, 400 MHz
1.99
5.02
2.01
1.00
1.01
1.01
1.01
f1 (ppm)

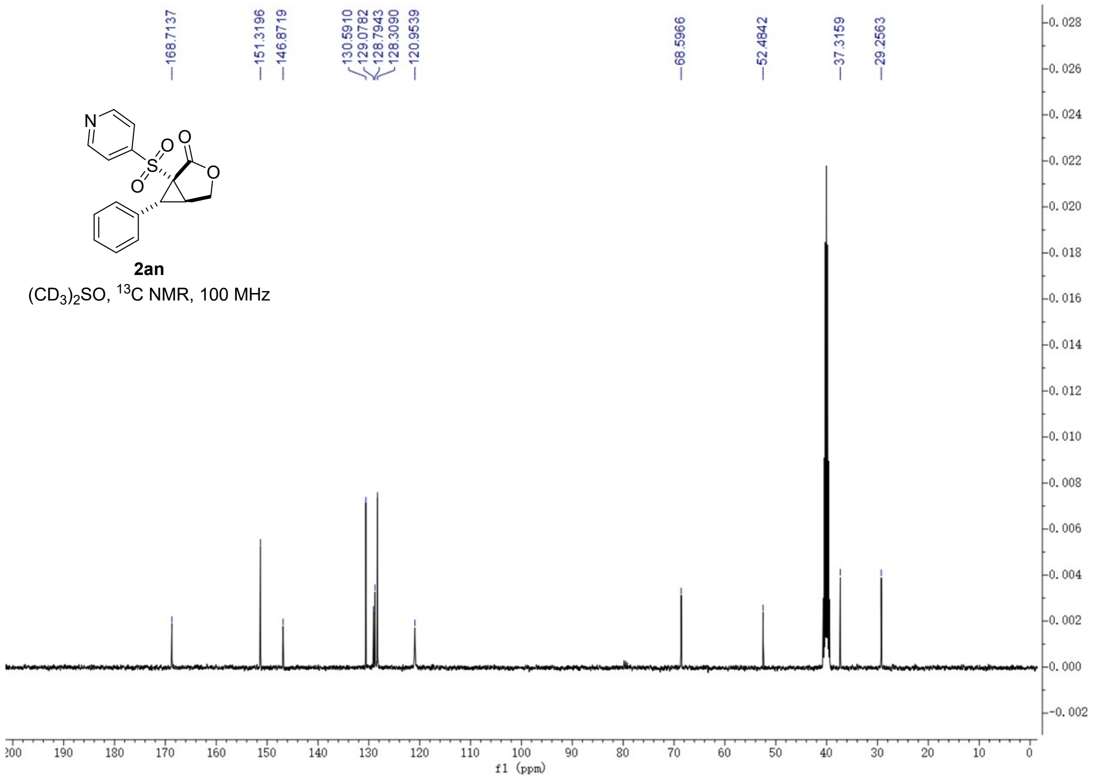
168.7137
151.3196
146.8719
130.5910
129.0782
128.7943
128.3090
120.9539
68.5966
52.4842
37.3159
29.2563
2an
(CD3)2SO, 13C NMR, 100 MHz
f1 (ppm)

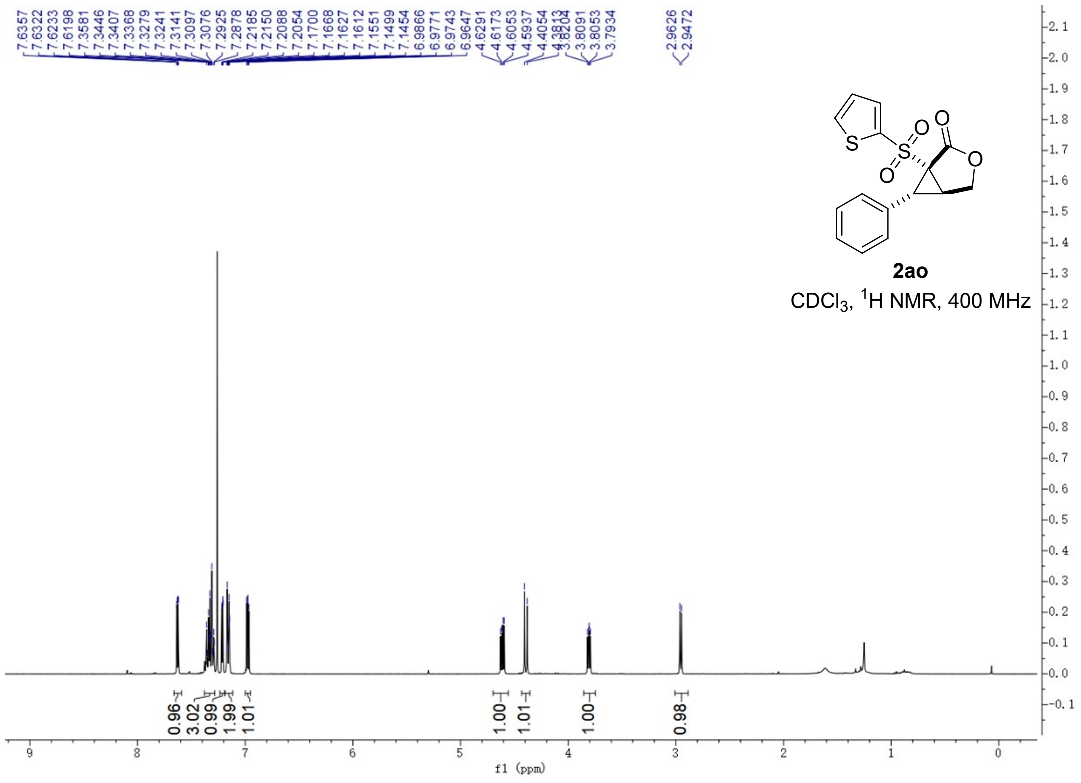
2ao
CDCl3, 1H NMR, 400 MHz
f1 (ppm)

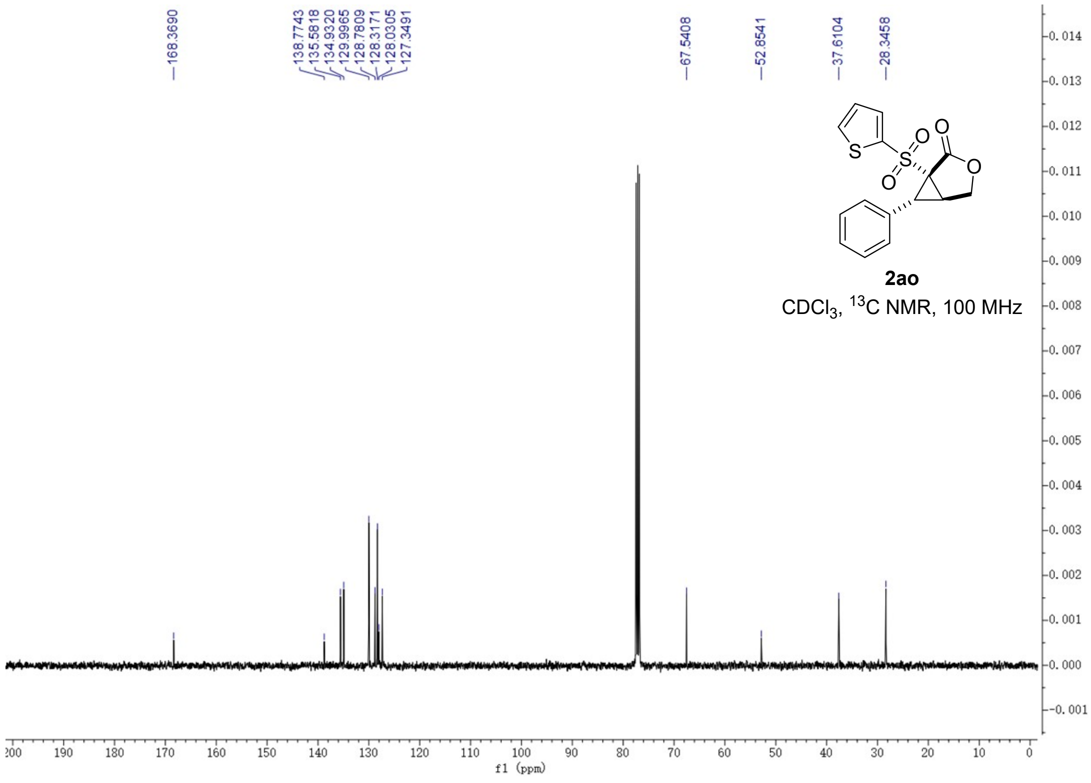
2ao
CDCl3, 13C NMR, 100 MHz
f1 (ppm)

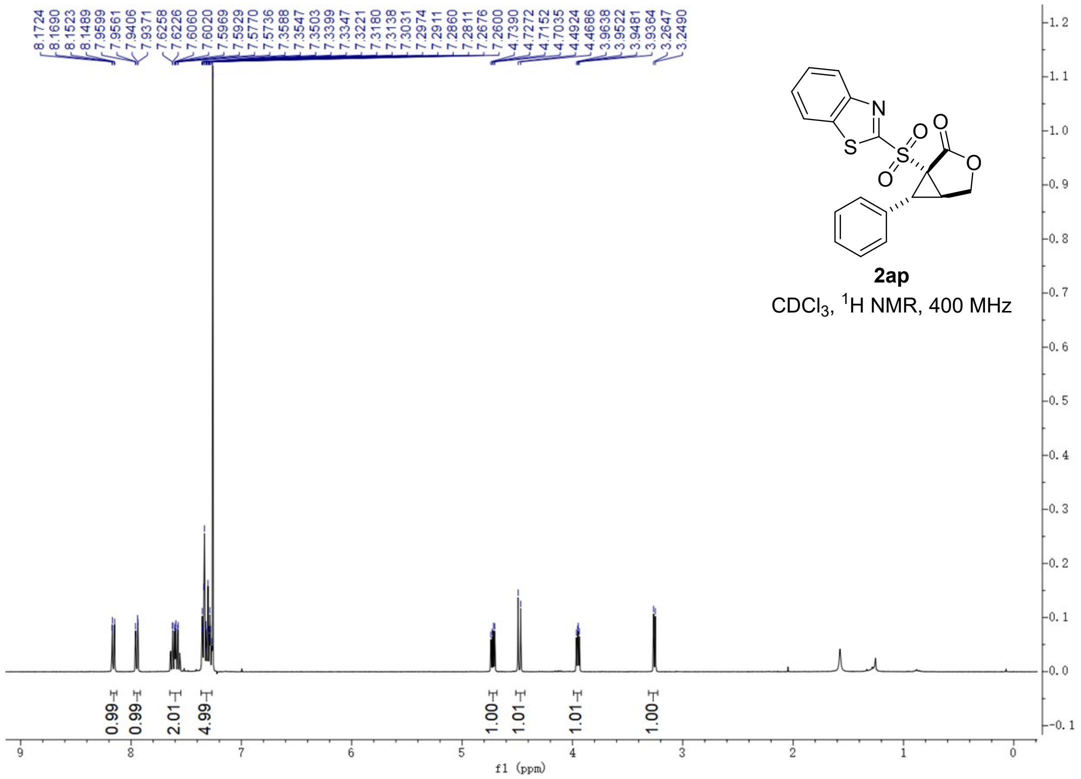

2ap
CDCl3, 1H NMR, 400 MHz
f1 (ppm)

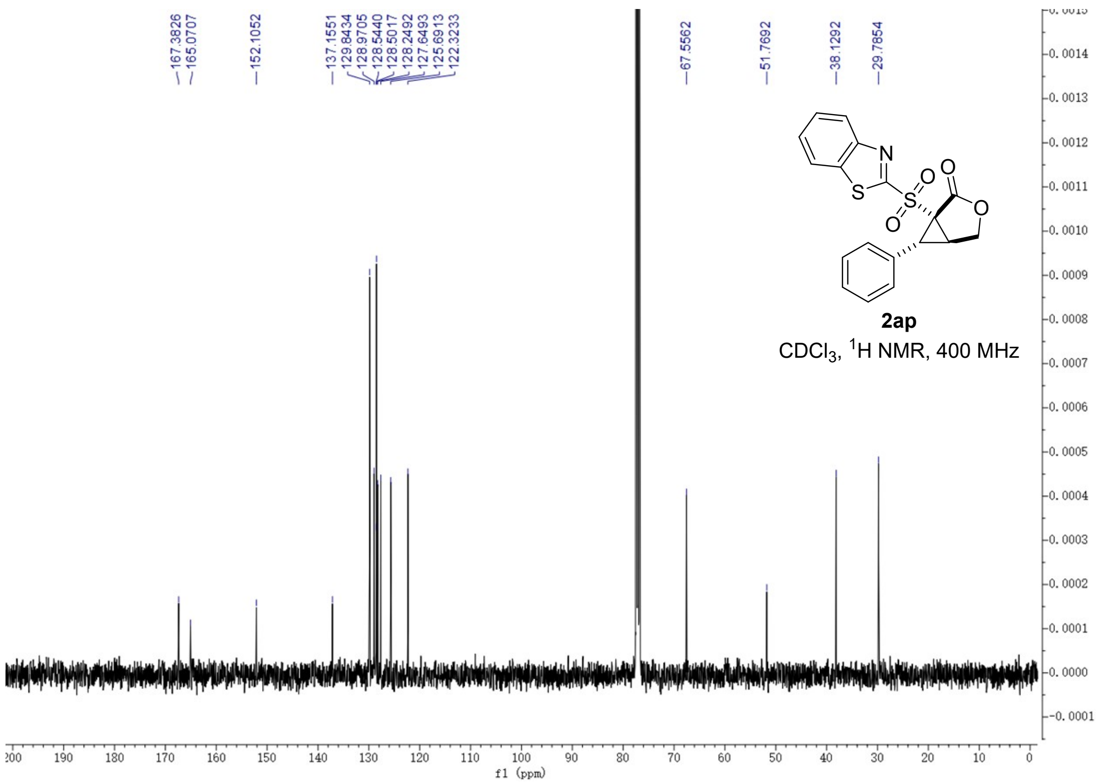

2ap
CDCl3, 1H NMR, 400 MHz
f1 (ppm)

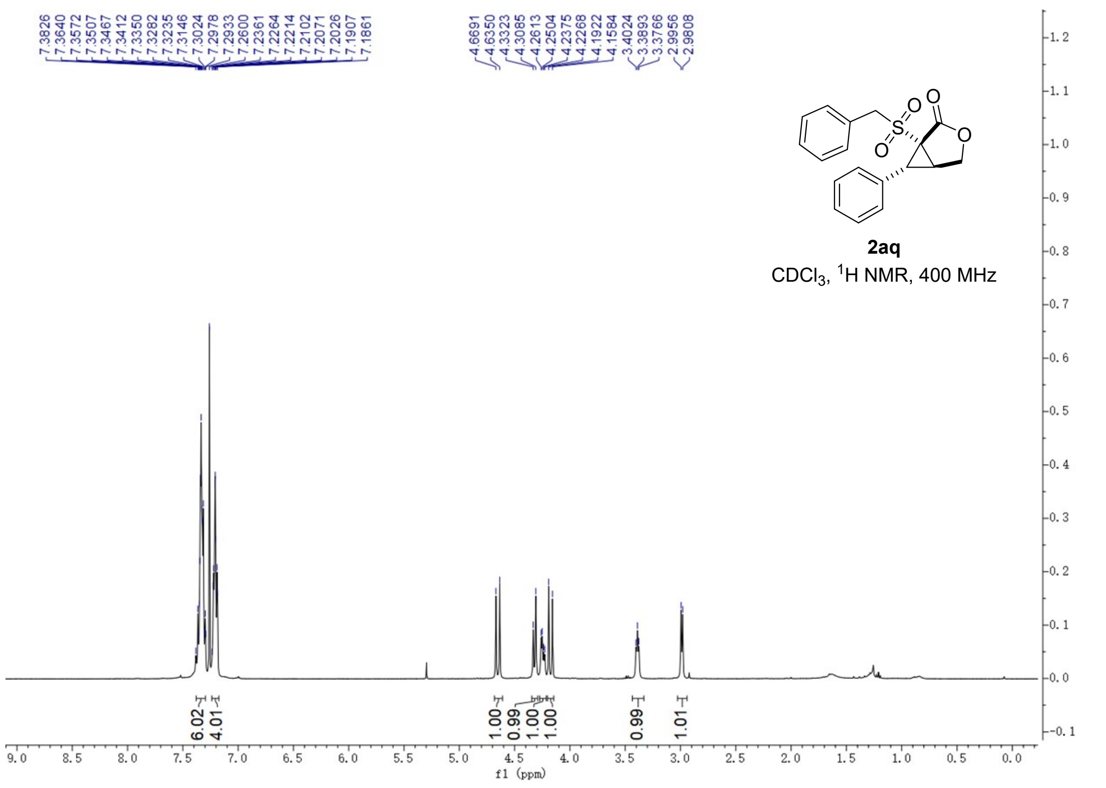
2aq
CDCl3, 1H NMR, 400 MHz

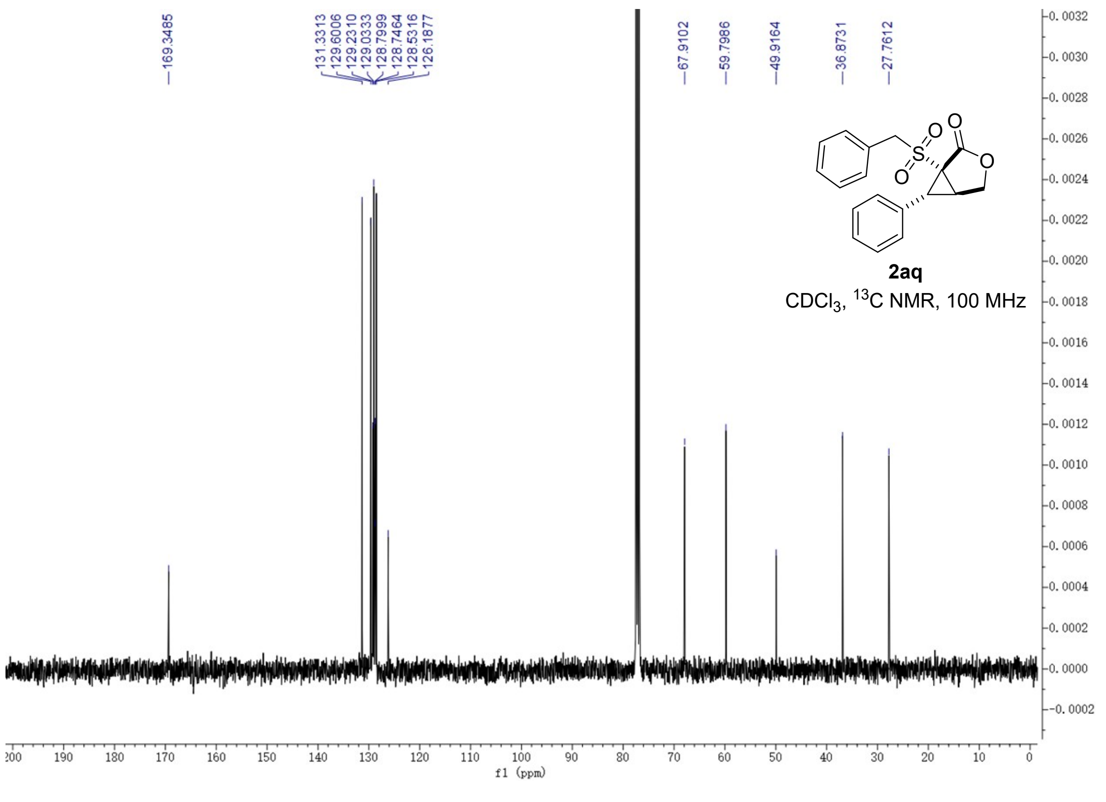
2aq
CDCl3, 13C NMR, 100 MHz

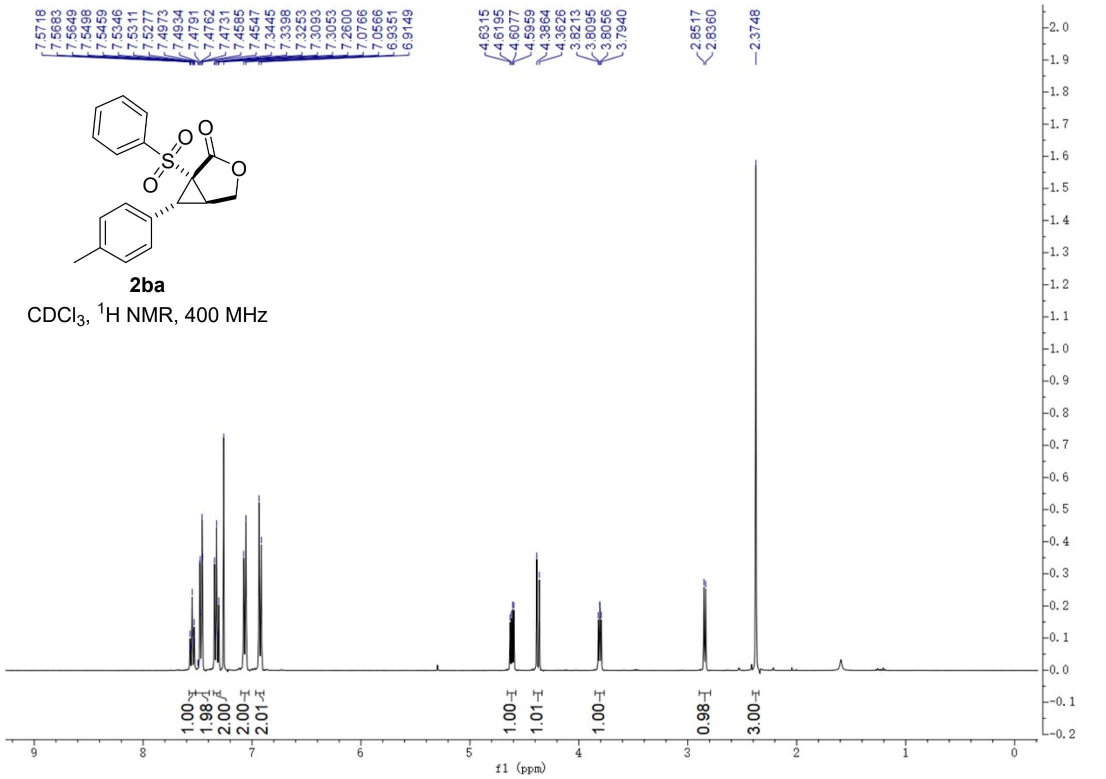

2ba

$CDCl_3$, ^1H NMR, 400 MHz

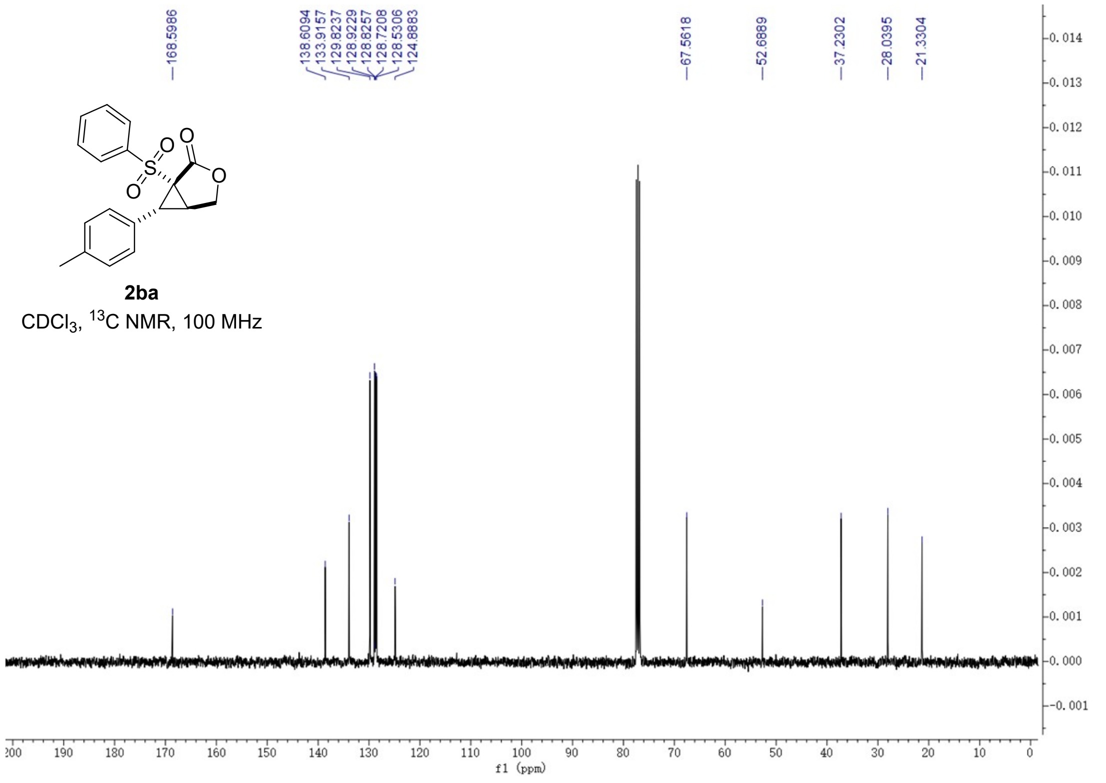

2ba

$CDCl_3$, ^{13}C NMR, 100 MHz

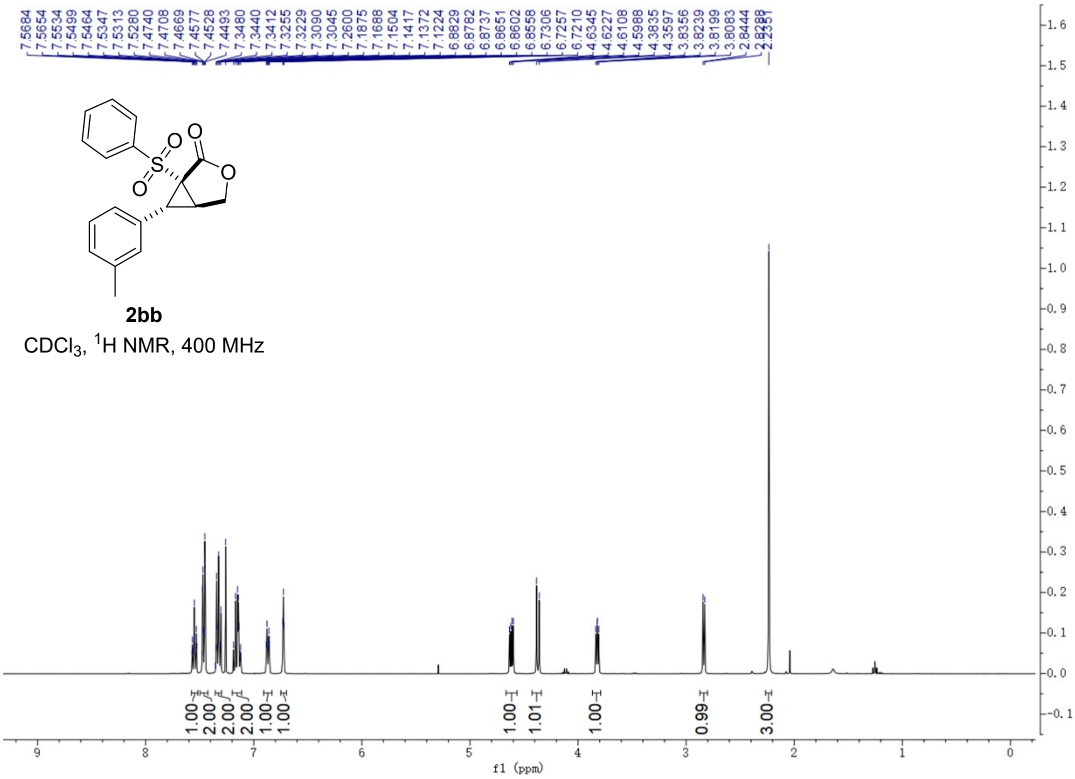
7.5684
7.5654
7.5534
7.5499
7.5464
7.5347
7.5313
7.5280
7.4740
7.4708
7.4669
7.4577
7.4528
7.4493
7.3480
7.3440
7.3412
7.3255
7.3229
7.3090
7.3045
7.2600
7.1875
7.1688
7.1504
7.1417
7.1372
7.1224
6.8829
6.8782
6.8737
6.8651
6.8602
6.8558
6.7306
6.7257
6.7210
4.6345
4.6227
4.6108
4.5988
4.3835
4.3597
3.8356
3.8239
3.8199
3.8083
2.8444
2.8288
2.2351
2bb
CDCl3, 1H NMR, 400 MHz
1.00
2.00
2.00
2.00
1.00
1.00
1.00
1.01
1.00
0.99
3.00
f1 (ppm)

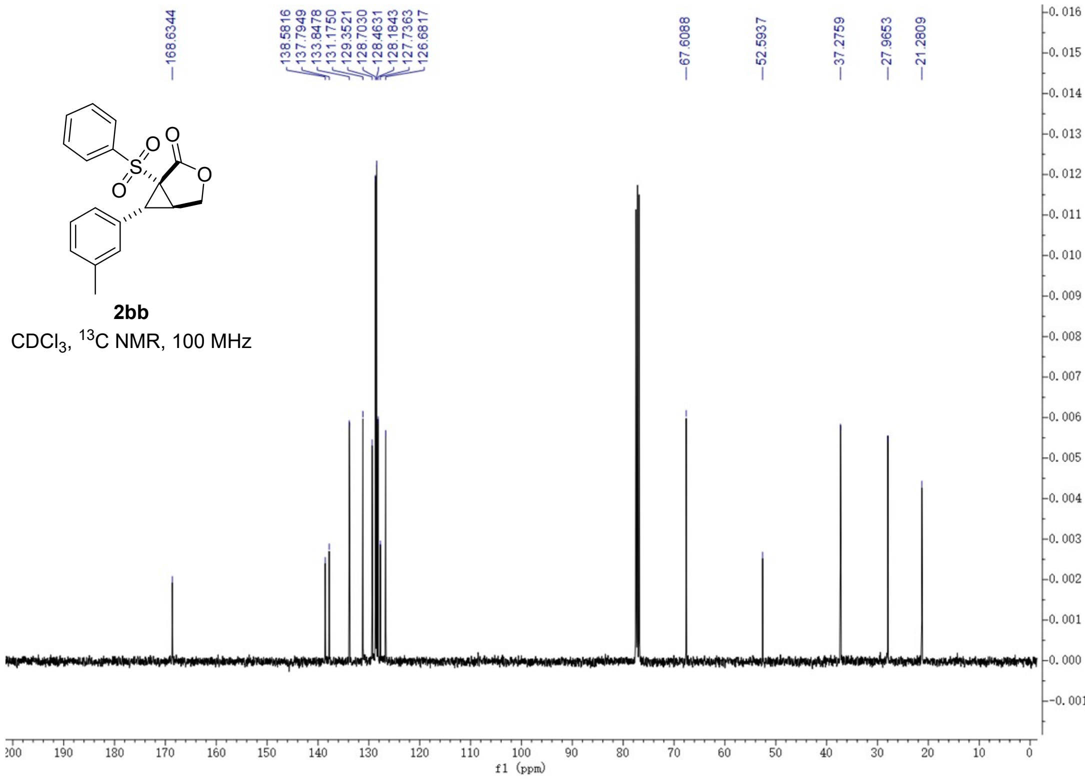
168.6344
138.5816
137.7949
133.8478
131.1750
129.3521
128.7030
128.4631
128.1843
127.7363
126.6817
67.6088
52.5937
37.2759
27.9653
21.2809
2bb
CDCl3, 13C NMR, 100 MHz
f1 (ppm)

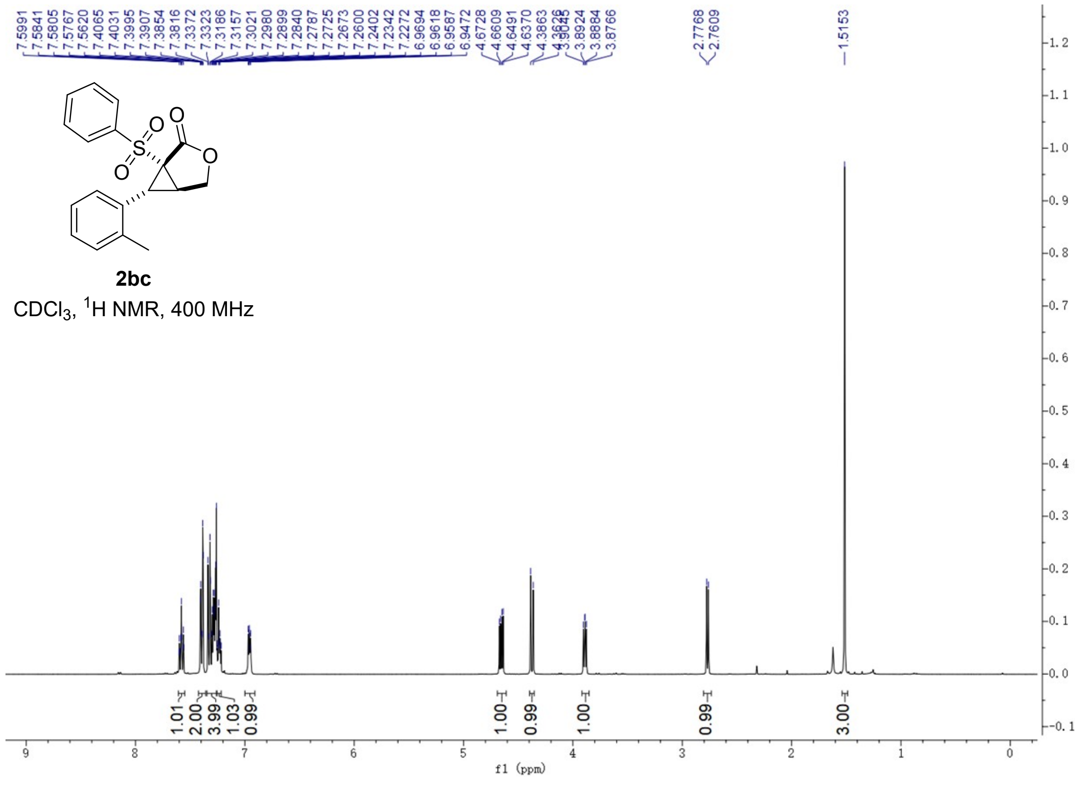
2bc
CDCl3, 1H NMR, 400 MHz
f1 (ppm)

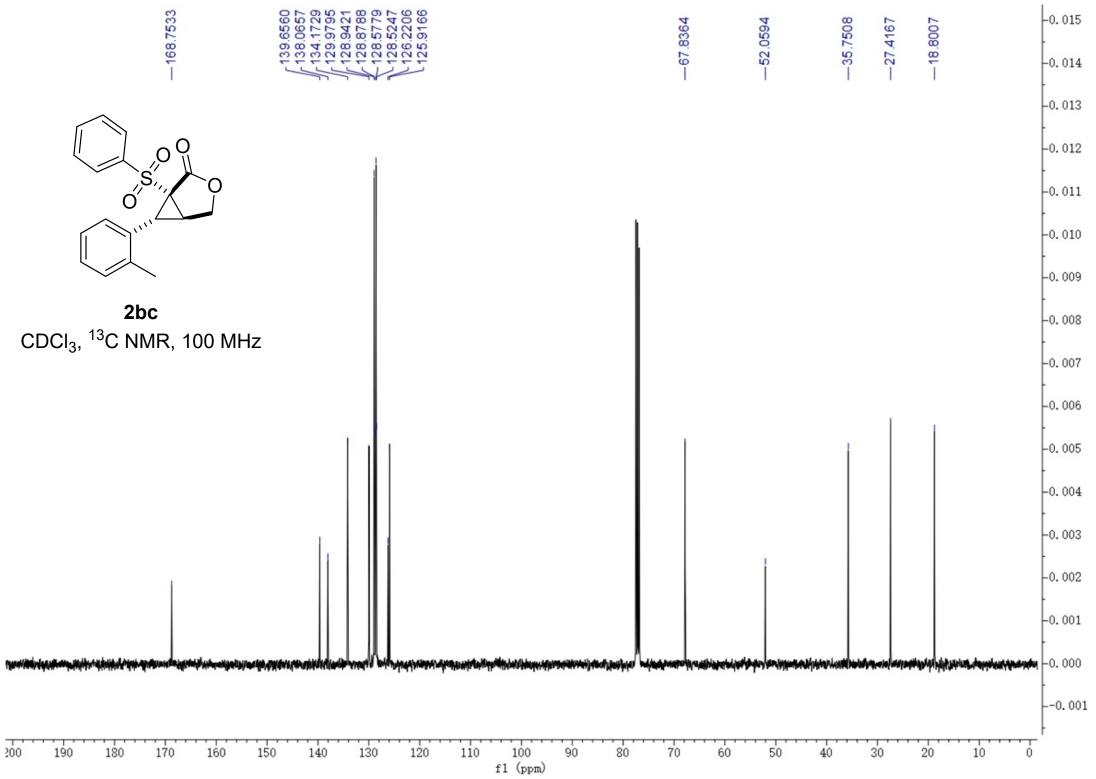
2bc
CDCl3, 13C NMR, 100 MHz
f1 (ppm)

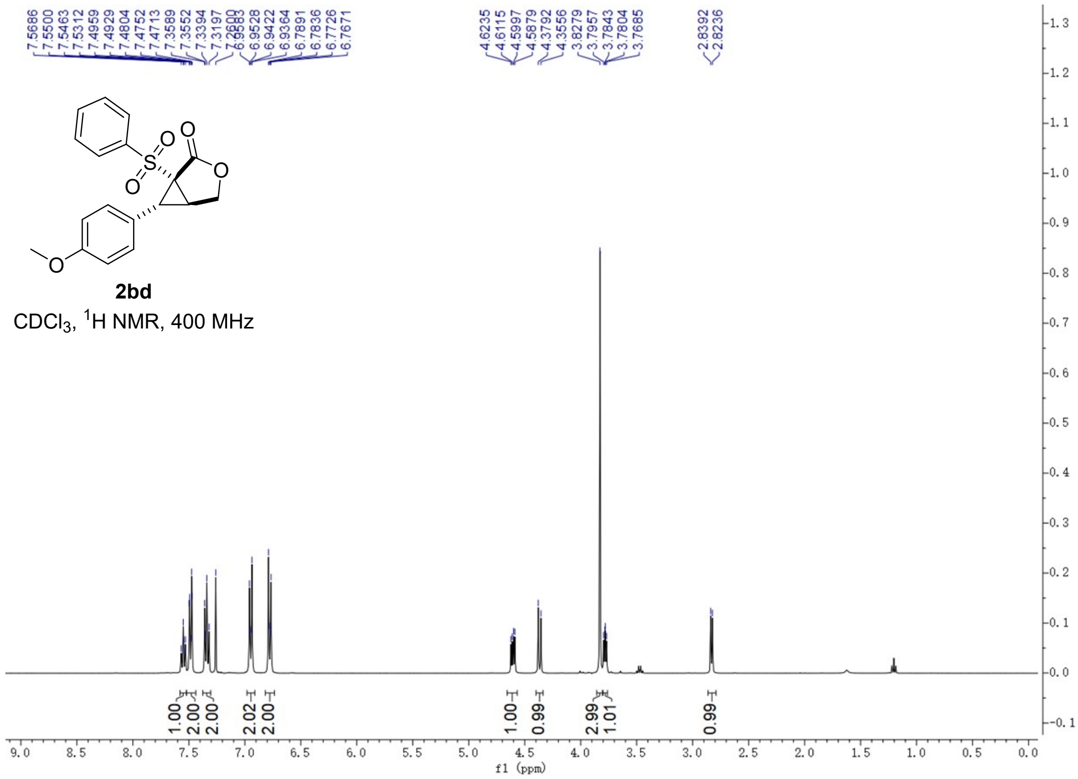

2bd

$CDCl_3$, ^{1}H NMR, 400 MHz

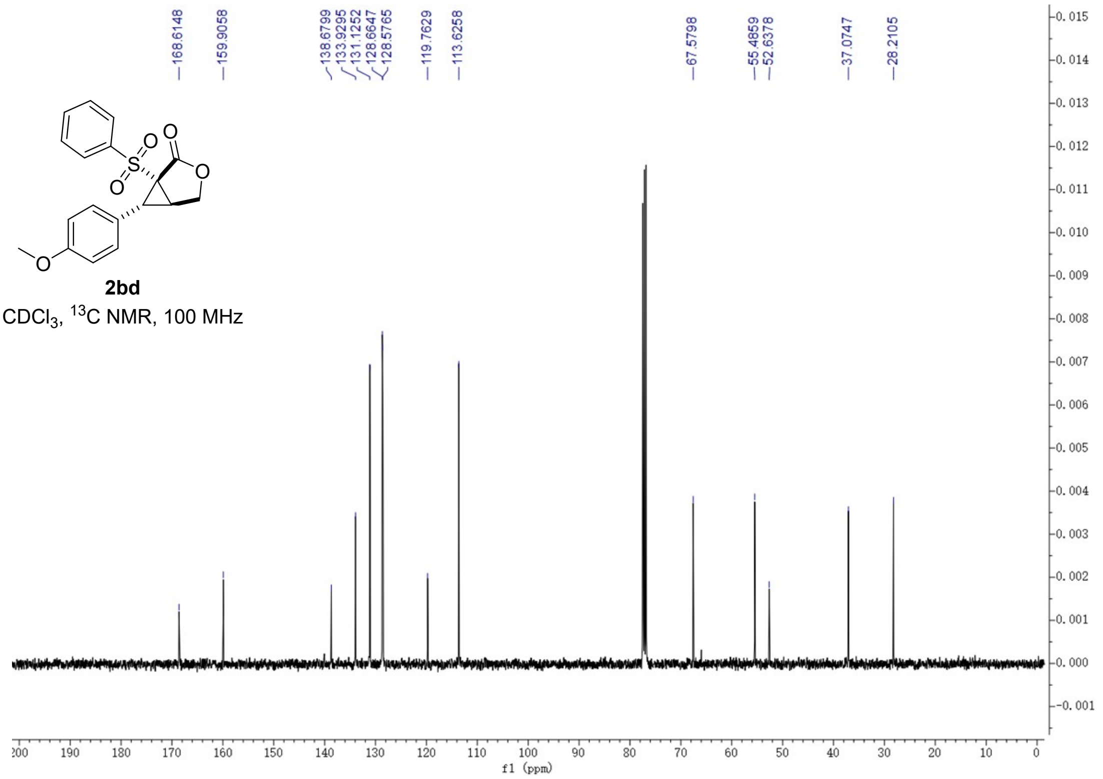

2bd

$CDCl_3$, ^{13}C NMR, 100 MHz

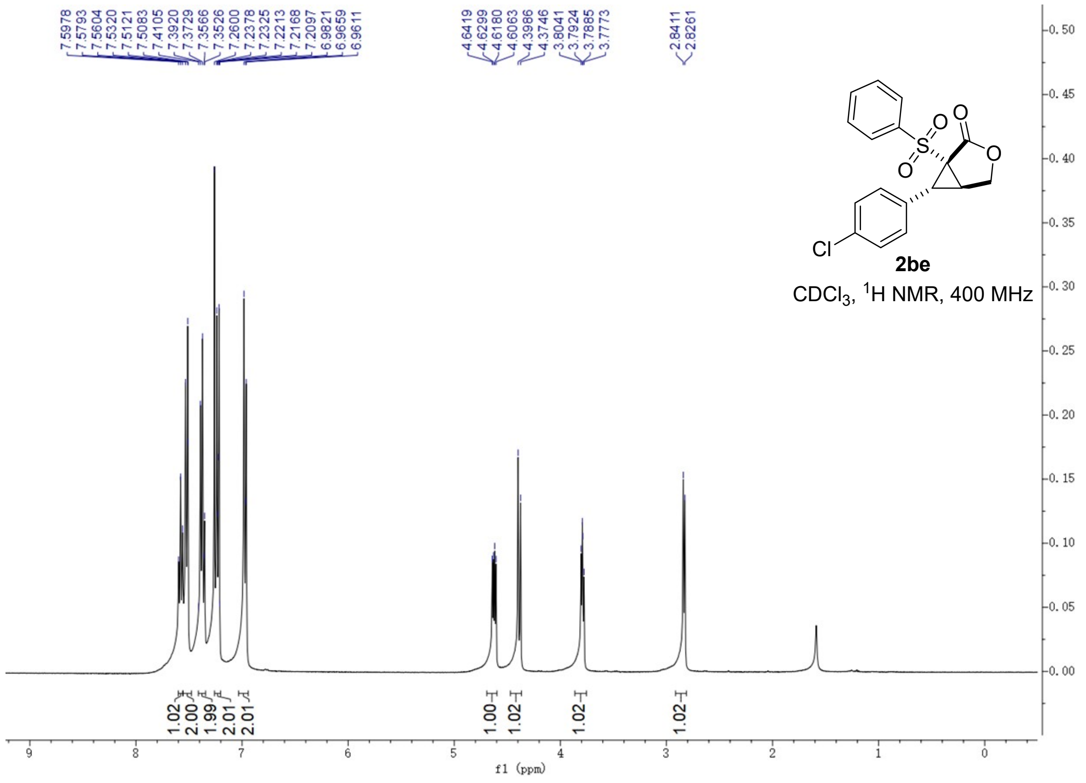
7.5978
7.5793
7.5604
7.5320
7.5121
7.5083
7.4105
7.3920
7.3729
7.3566
7.3526
7.2600
7.2378
7.2325
7.2213
7.2168
7.2097
6.9821
6.9659
6.9611
4.6419
4.6299
4.6180
4.6063
4.3986
4.3746
3.8041
3.7924
3.7885
3.7773
2.8411
2.8261
2be
CDCl3, 1H NMR, 400 MHz
1.02
2.00
1.99
2.01
2.01
1.00
1.02
1.02
1.02
f1 (ppm)

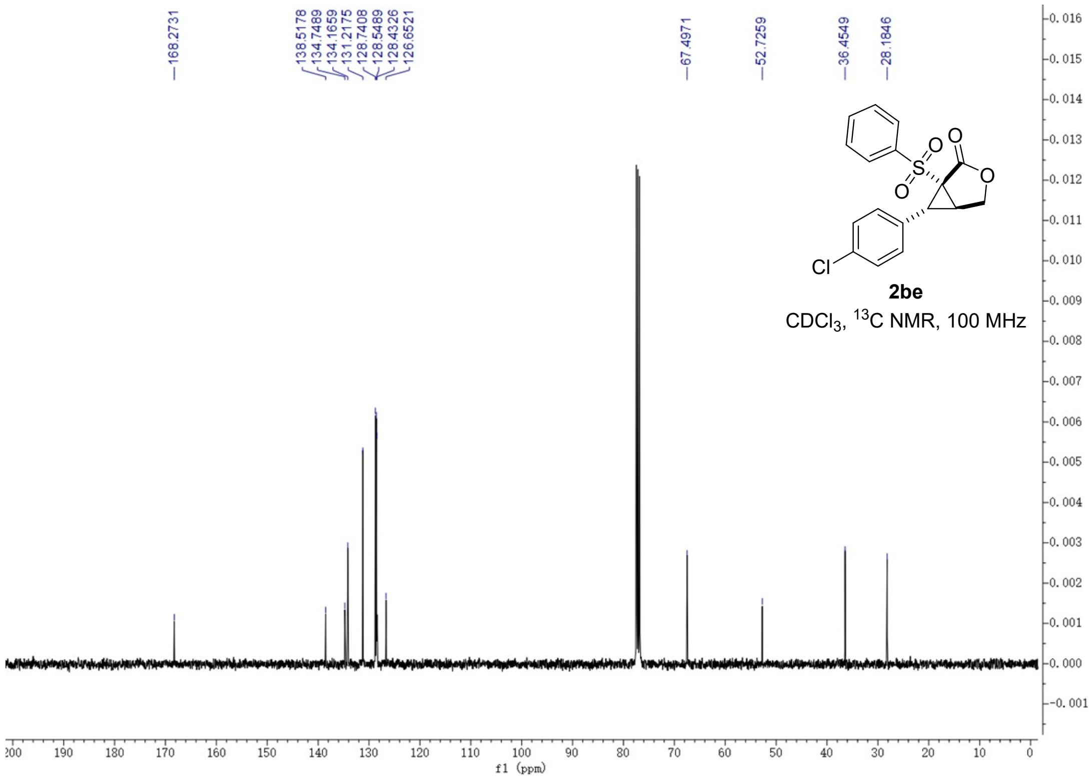
168.2731
138.5178
134.7489
134.1659
131.2175
128.7408
128.5489
128.4326
126.6521
67.4971
52.7259
36.4549
28.1846
2be
CDCl3, 13C NMR, 100 MHz
f1 (ppm)

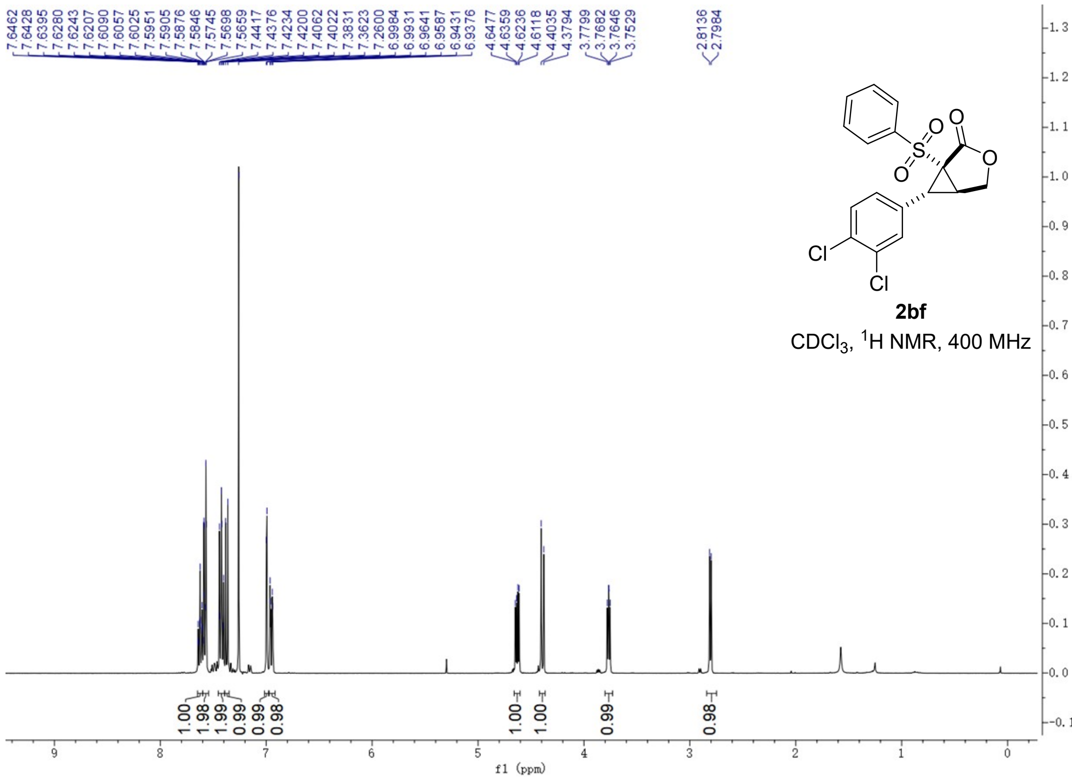
2bf
CDCl3, 1H NMR, 400 MHz
f1 (ppm)

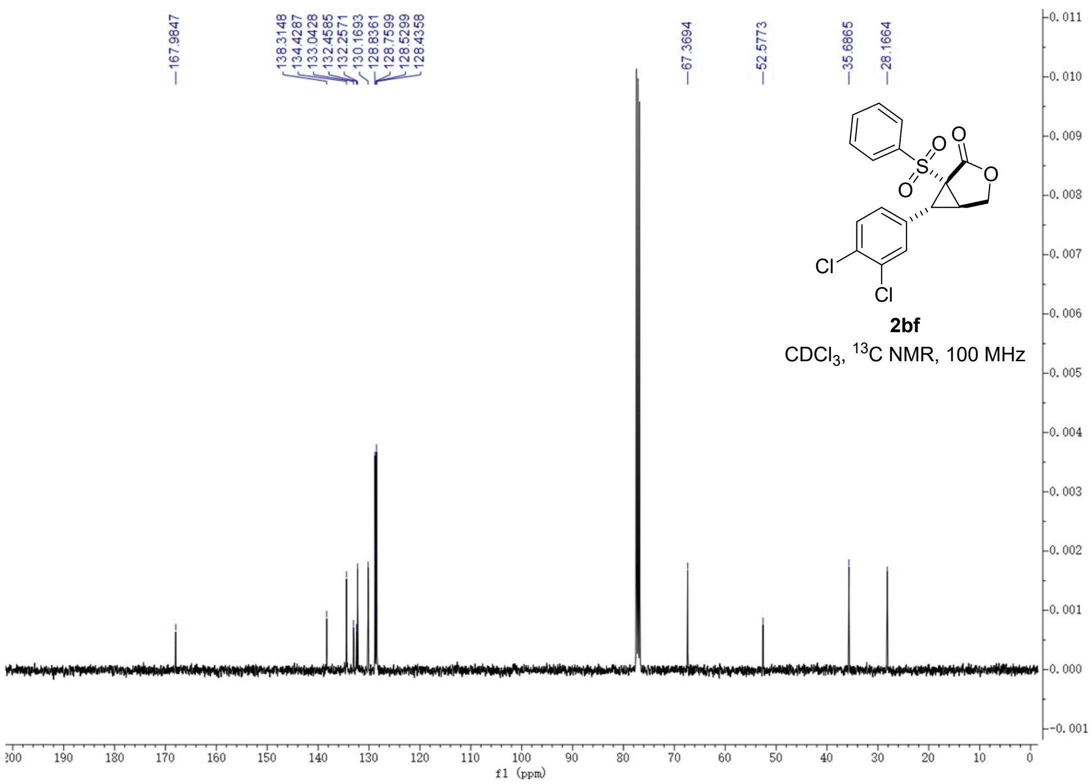
2bf
CDCl3, 13C NMR, 100 MHz
f1 (ppm)

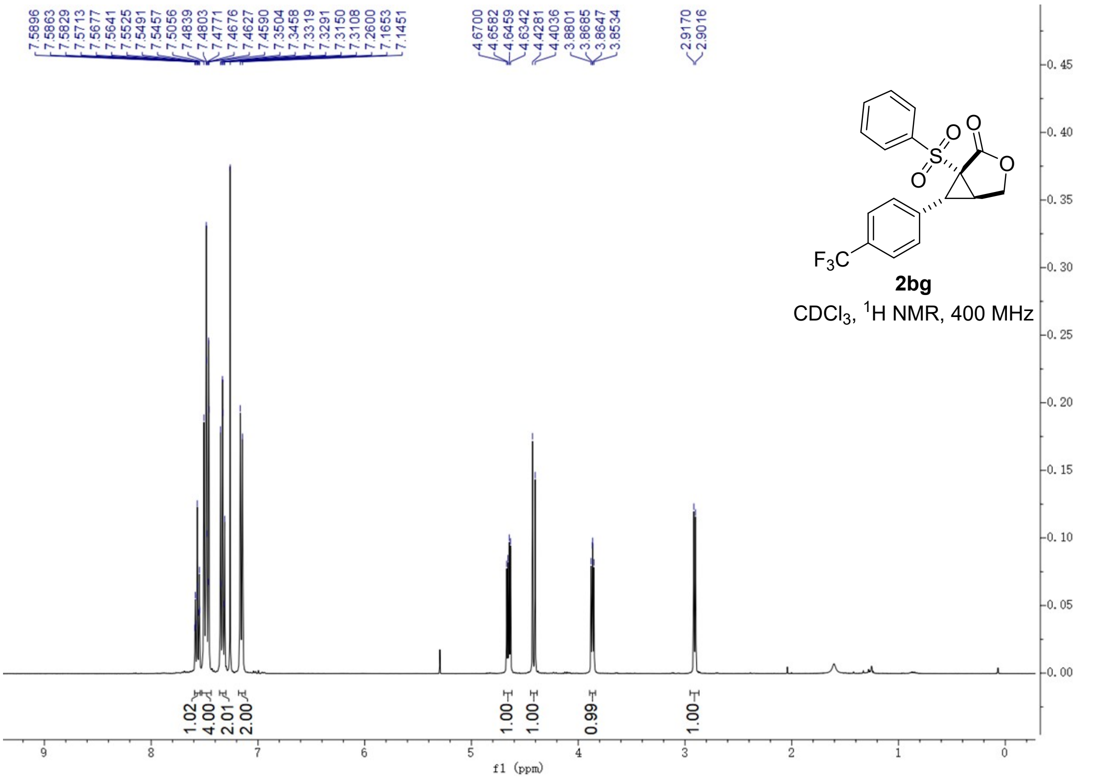
7.5896
7.5863
7.5829
7.5713
7.5677
7.5641
7.5525
7.5491
7.5457
7.5056
7.4839
7.4803
7.4771
7.4676
7.4627
7.4590
7.3504
7.3458
7.3319
7.3291
7.3150
7.3108
7.2600
7.1653
7.1451
4.6700
4.6582
4.6459
4.6342
4.4281
4.4036
3.8801
3.8685
3.8647
3.8534
2.9170
2.9016
F3C
2bg
CDCl3, 1H NMR, 400 MHz
1.02
4.00
2.01
2.00
1.00
1.00
0.99
1.00
f1 (ppm)

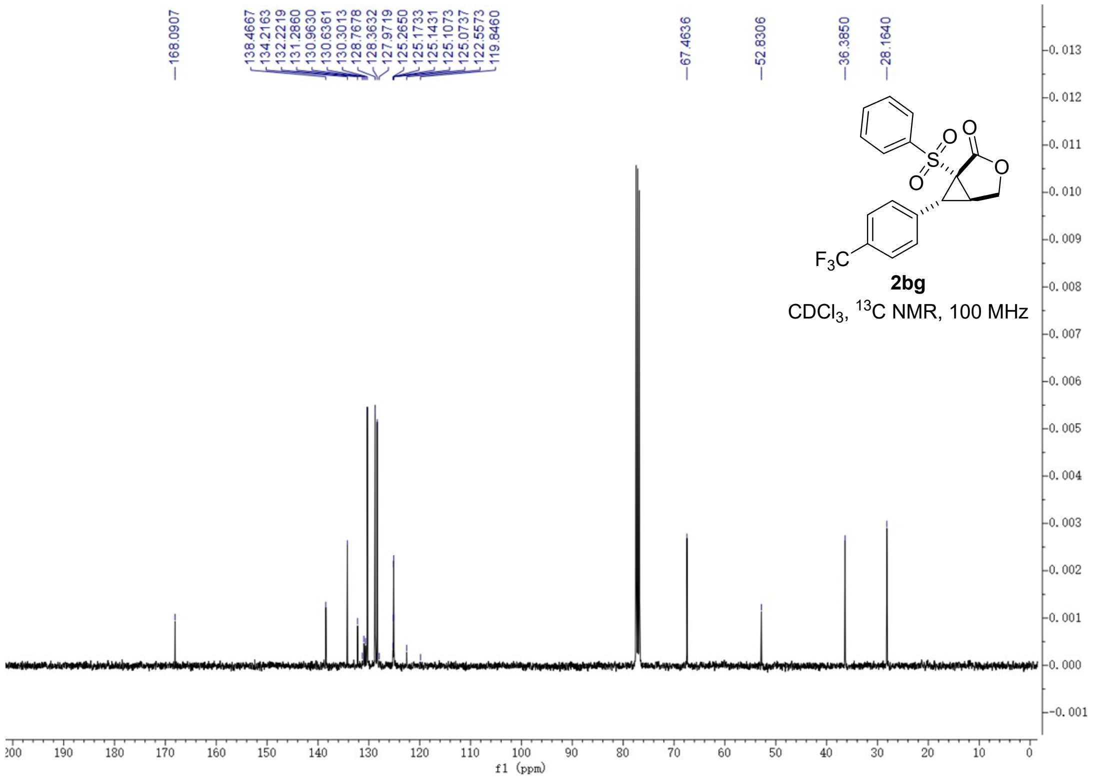
168.0907
138.4667
134.2163
132.2219
131.2860
130.9630
130.6361
130.3013
128.7678
128.3632
127.9719
125.2650
125.1733
125.1431
125.1073
125.0737
122.5573
119.8460
67.4636
52.8306
36.3850
28.1640
F3C
2bg
CDCl3, 13C NMR, 100 MHz
f1 (ppm)

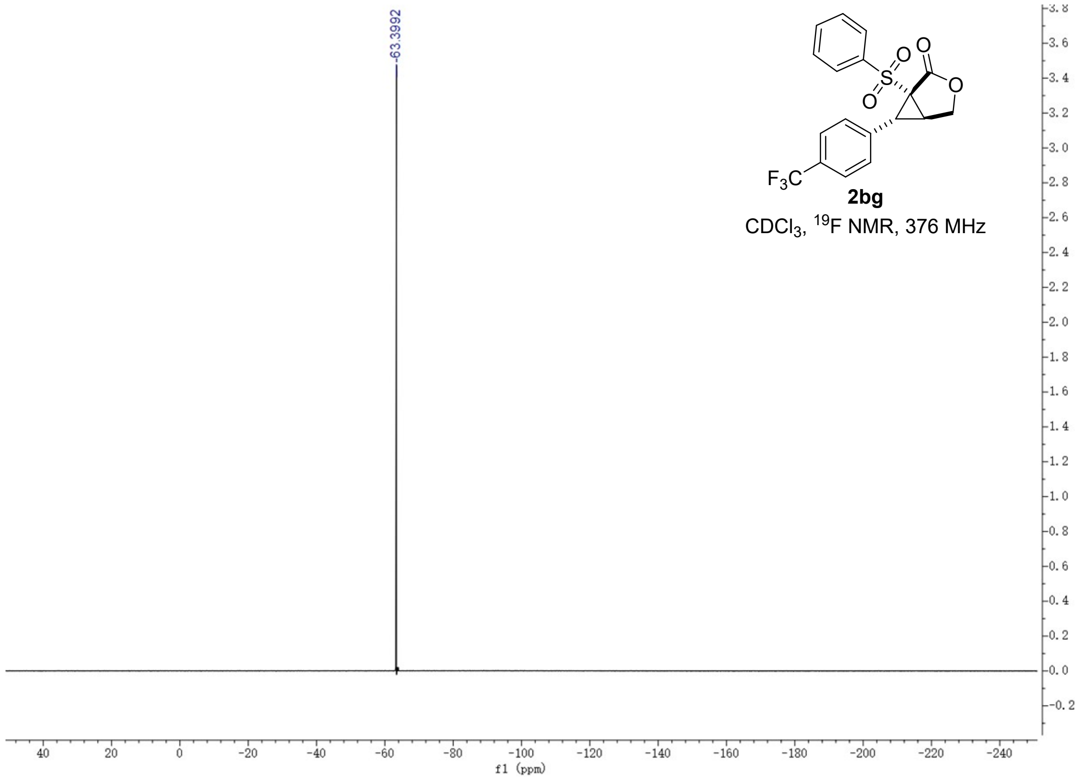
-63.3992
F3C
2bg
CDCl3, 19F NMR, 376 MHz
f1 (ppm)

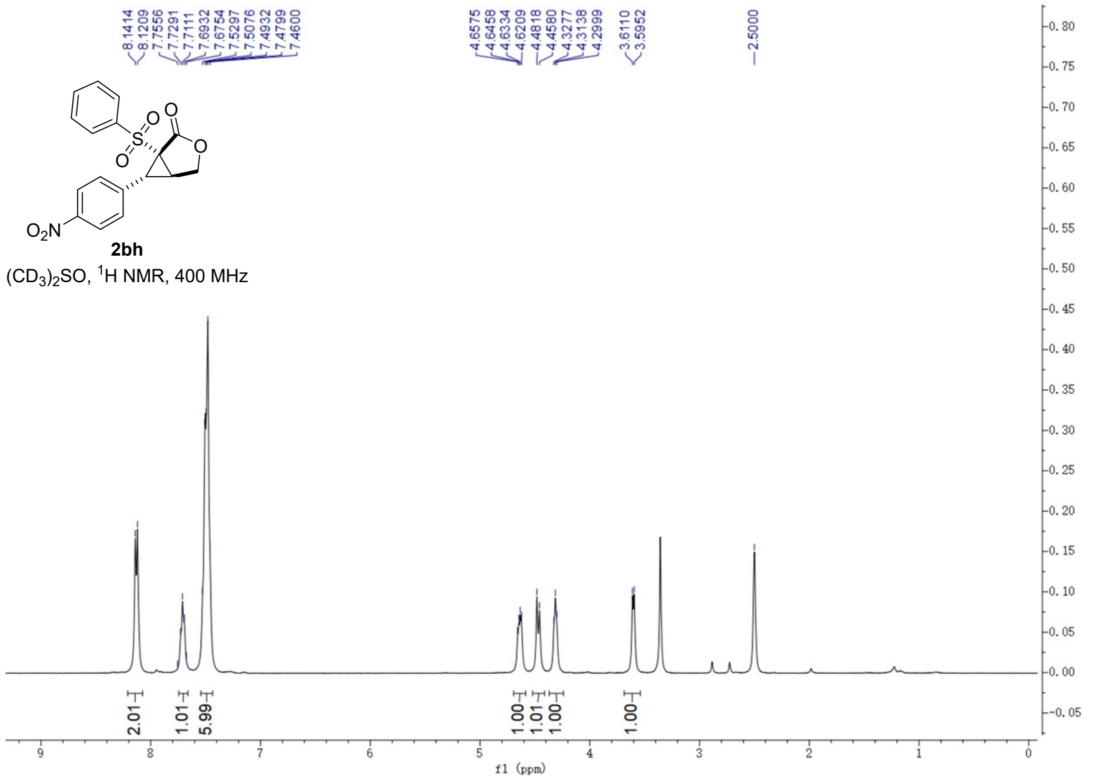
8.1414
8.1209
7.7556
7.7291
7.7111
7.6932
7.6754
7.5297
7.5076
7.4932
7.4799
7.4600
4.6575
4.6458
4.6334
4.6209
4.4818
4.4580
4.3277
4.3138
4.2999
3.6110
3.5952
2.5000
O
S
O
O
O
O_2N
2bh
$(CD_3)_2SO$, ^1H NMR, 400 MHz
2.01
1.01
5.99
1.00
1.01
1.00
1.00
f1 (ppm)

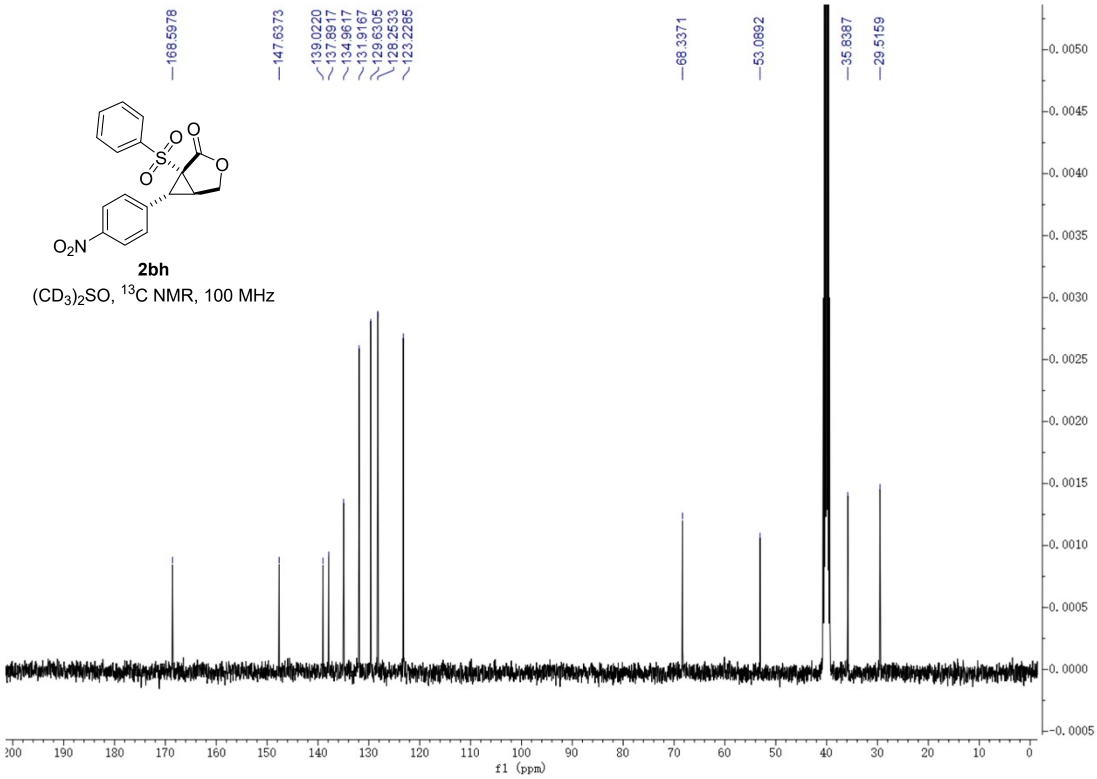
168.5978
147.6373
139.0220
137.8917
134.9617
131.9167
129.6305
128.2533
123.2285
68.3371
53.0892
35.8387
29.5159
O
S
O
O
O
O_2N
2bh
$(CD_3)_2SO$, ^{13}C NMR, 100 MHz
f1 (ppm)

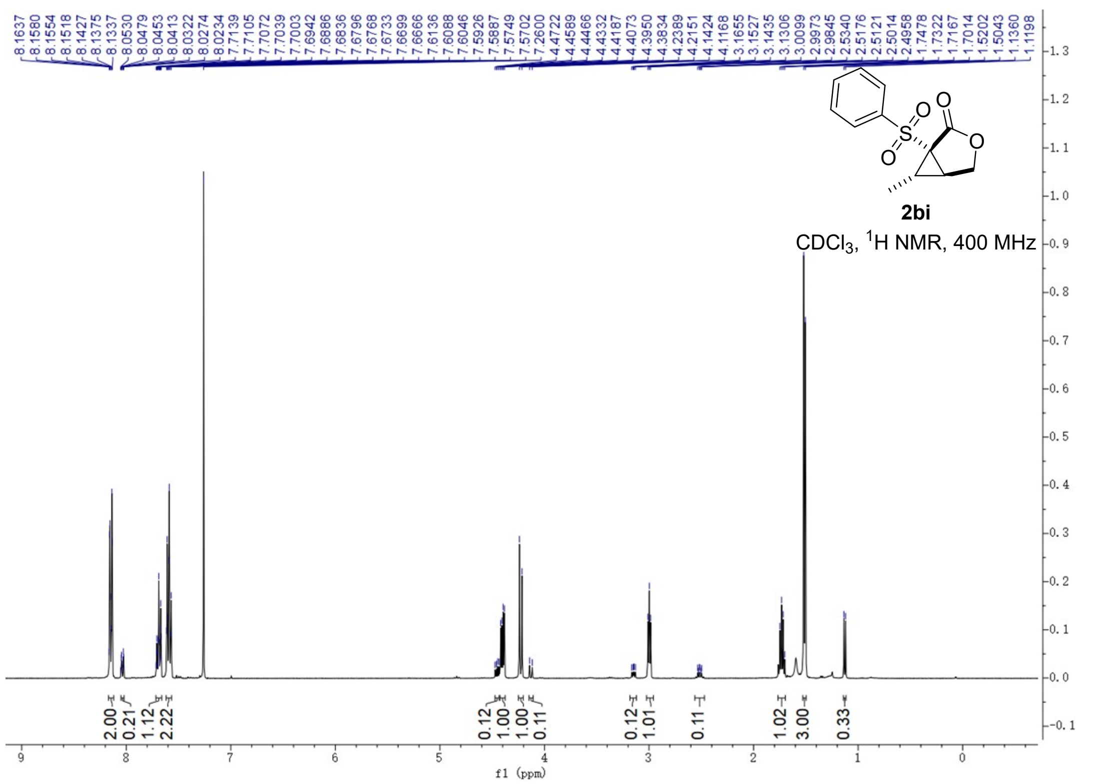
2bi
CDCl3, 1H NMR, 400 MHz
f1 (ppm)

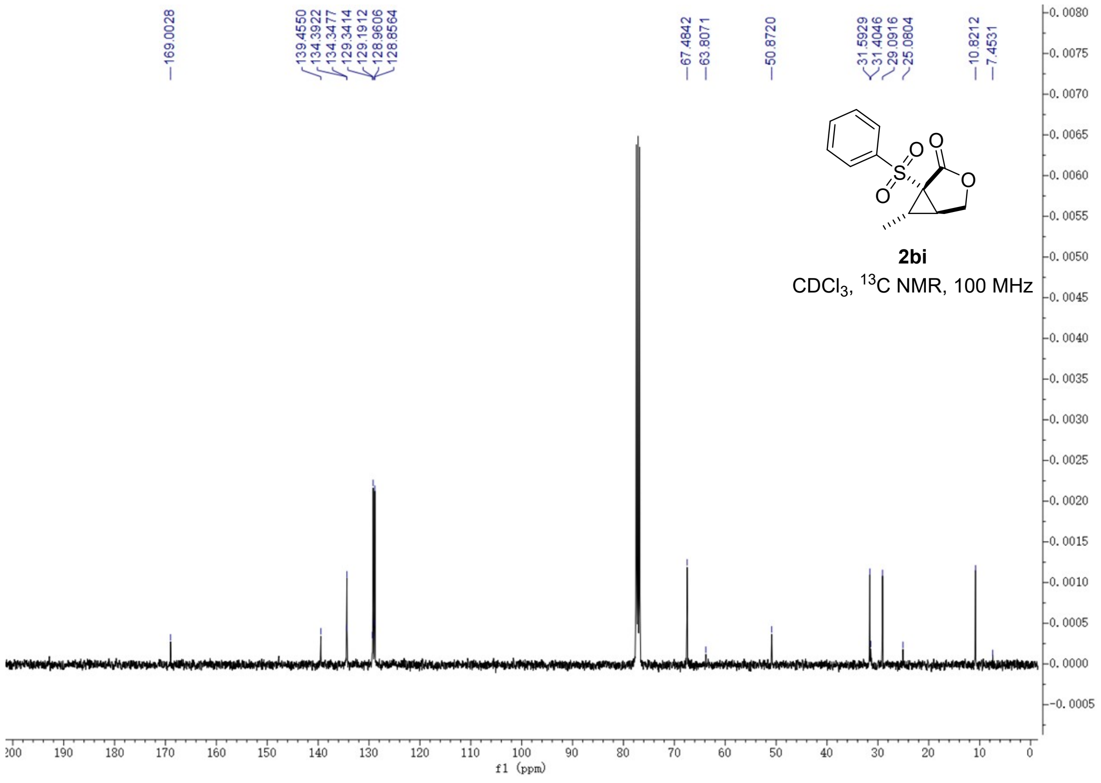
2bi
CDCl3, 13C NMR, 100 MHz
f1 (ppm)

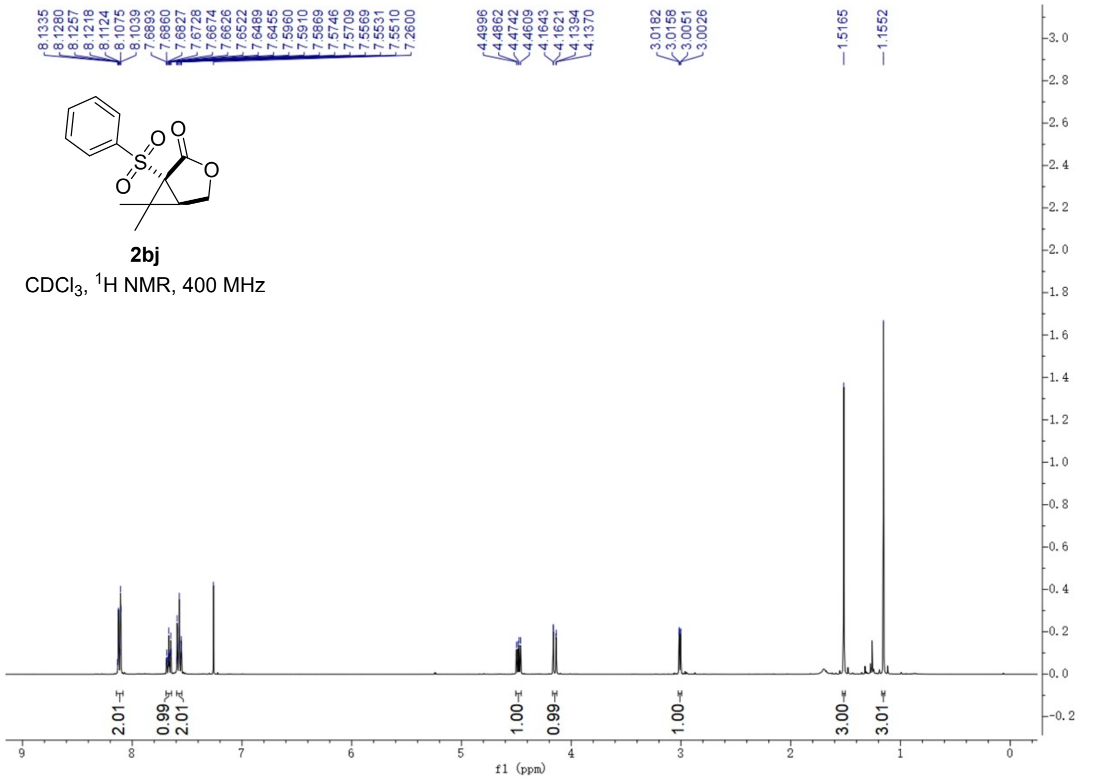
8.1335
8.1280
8.1257
8.1218
8.1124
8.1075
8.1039
7.6893
7.6860
7.6827
7.6728
7.6674
7.6626
7.6522
7.6489
7.6455
7.5960
7.5910
7.5869
7.5746
7.5709
7.5569
7.5531
7.5510
7.2600
4.4996
4.4862
4.4742
4.4609
4.1643
4.1621
4.1394
4.1370
3.0182
3.0158
3.0051
3.0026
1.5165
1.1552
2bj
CDCl3, 1H NMR, 400 MHz
2.01
0.99
2.01
1.00
0.99
1.00
3.00
3.01
f1 (ppm)

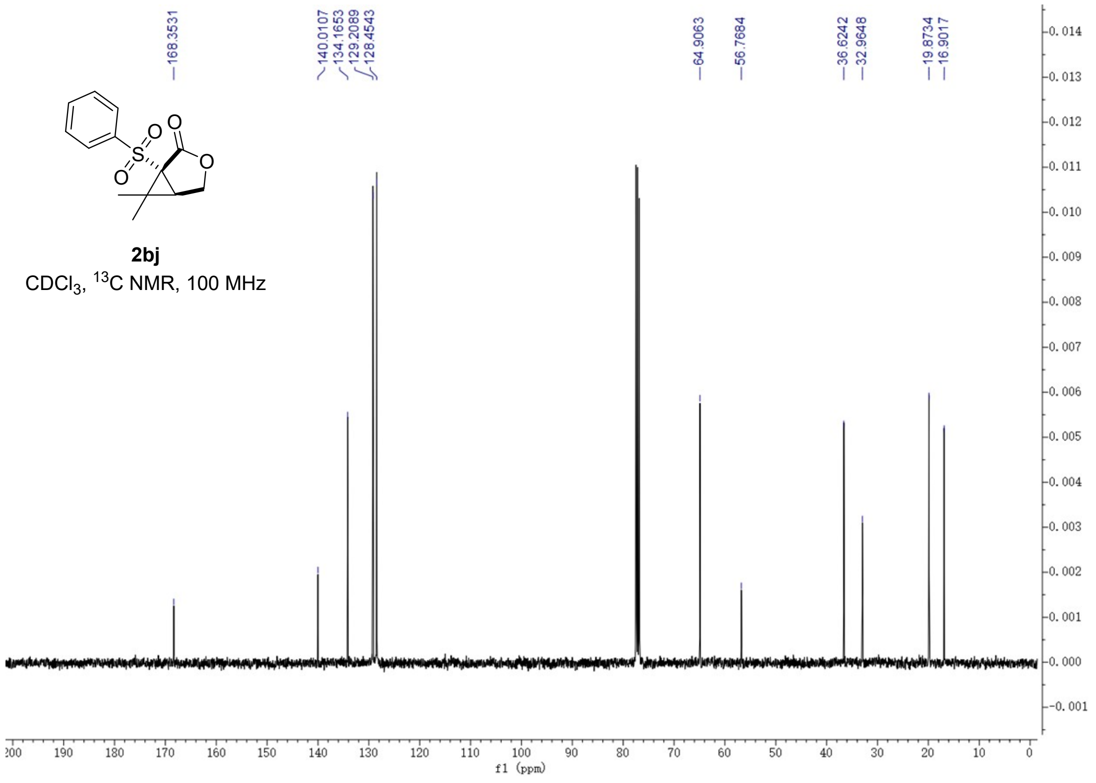
168.3531
140.0107
134.1653
129.2089
128.4543
64.9063
56.7684
36.6242
32.9648
19.8734
16.9017
2bj
CDCl3, 13C NMR, 100 MHz
f1 (ppm)

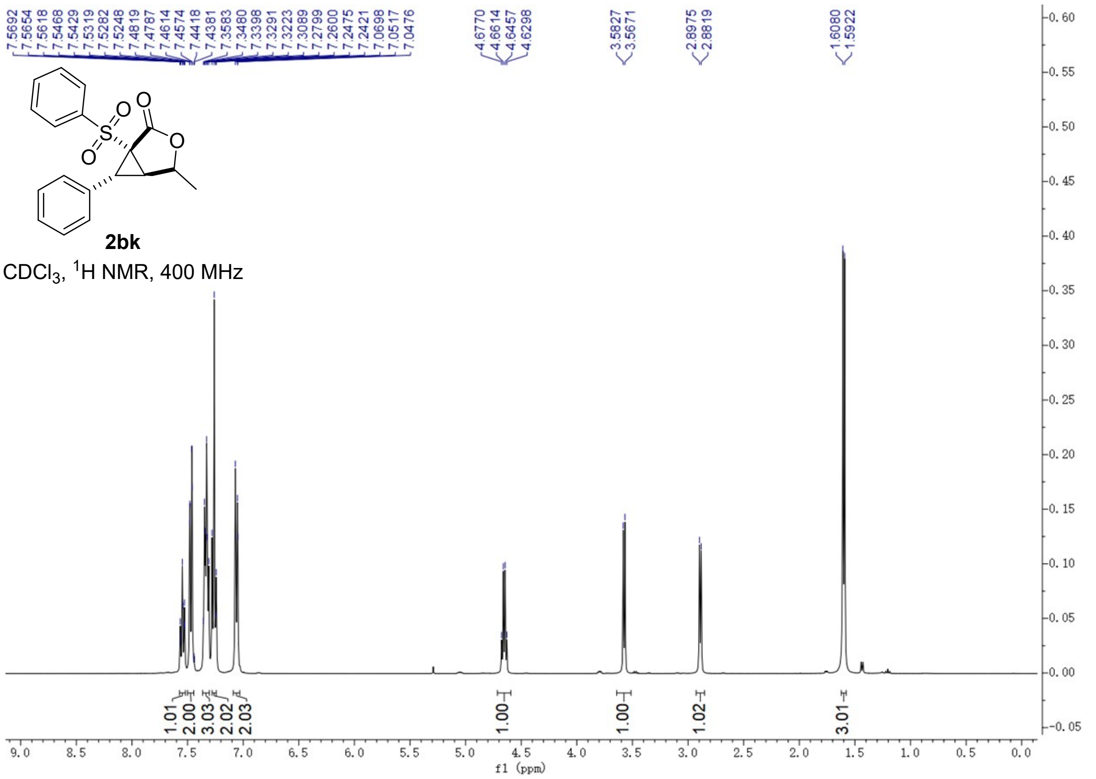

2bk

$CDCl_3$, ^1H NMR, 400 MHz

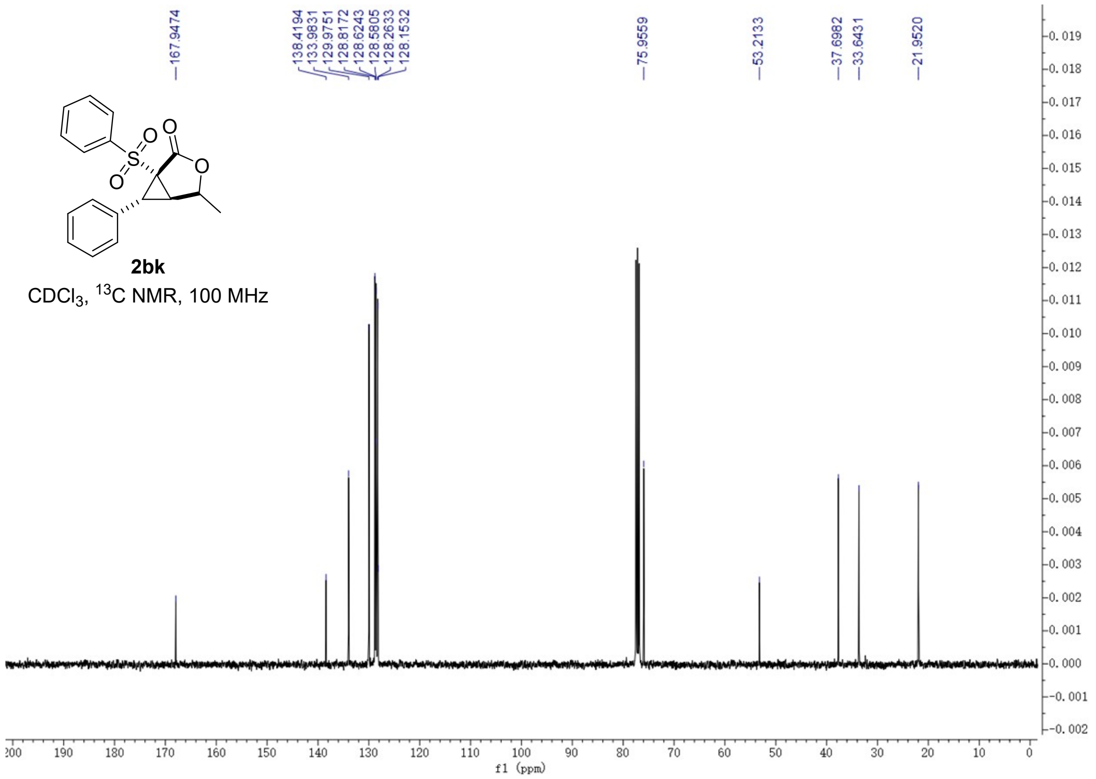

2bk

$CDCl_3$, ^{13}C NMR, 100 MHz

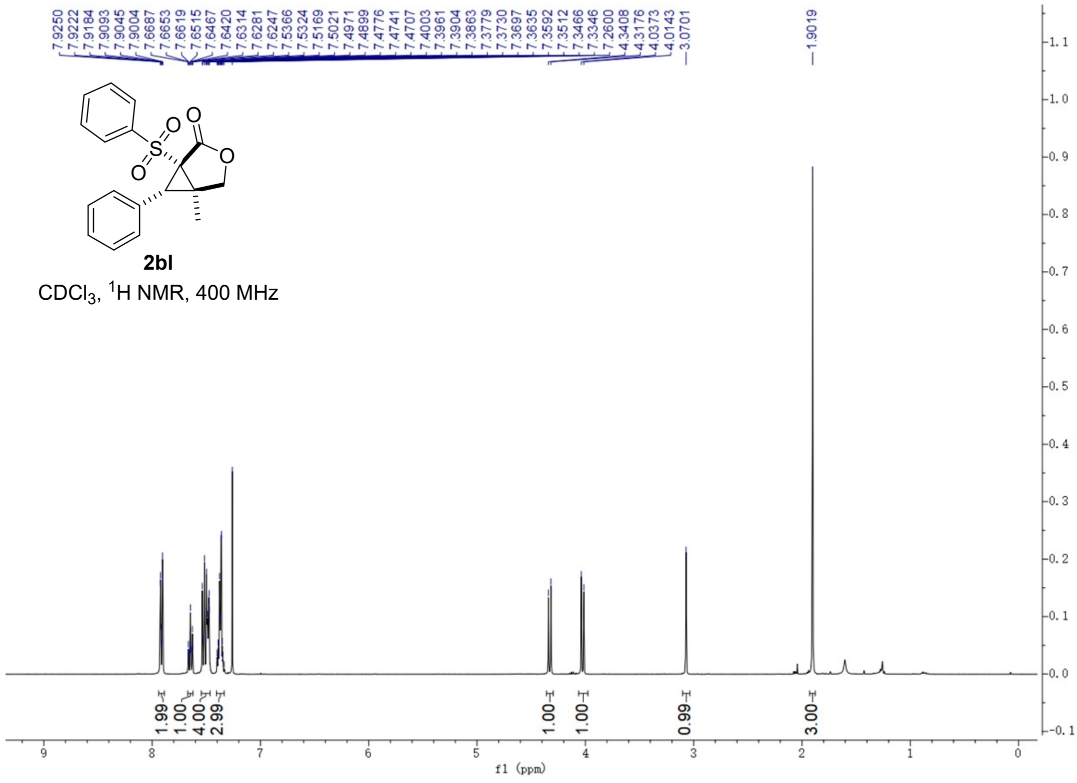
7.9250
7.9222
7.9184
7.9093
7.9045
7.9004
7.6687
7.6653
7.6619
7.6515
7.6467
7.6420
7.6314
7.6281
7.6247
7.5366
7.5324
7.5169
7.5021
7.4971
7.4899
7.4776
7.4741
7.4707
7.4003
7.3961
7.3904
7.3863
7.3779
7.3730
7.3697
7.3635
7.3592
7.3512
7.3466
7.3346
7.2600
4.3408
4.3176
4.0373
4.0143
3.0701
1.9019
2bl
CDCl3, 1H NMR, 400 MHz
1.99
1.00
4.00
2.99
1.00
1.00
0.99
3.00
f1 (ppm)

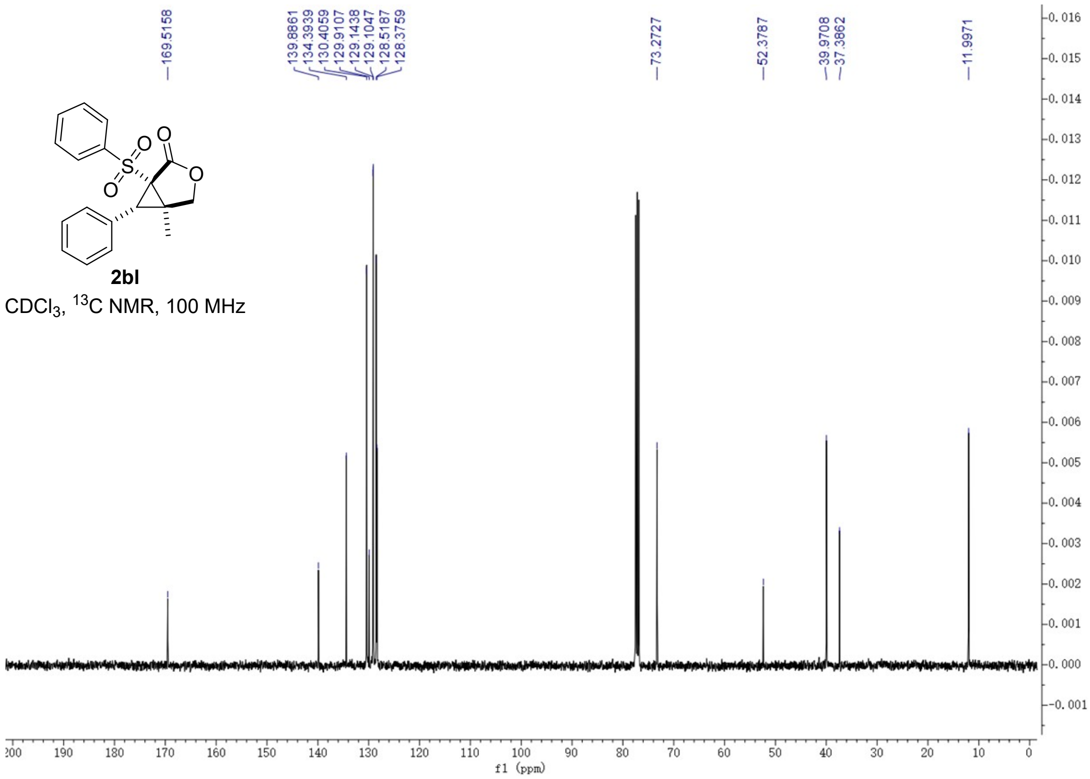
169.5158
139.8861
134.3939
130.4059
129.9107
129.1438
129.1047
128.5187
128.3759
73.2727
52.3787
39.9708
37.3862
11.9971
2bl
CDCl3, 13C NMR, 100 MHz
f1 (ppm)

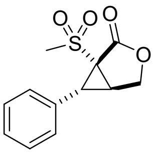

2bm

CDCl$_3$, ^{1}H NMR, 400 MHz

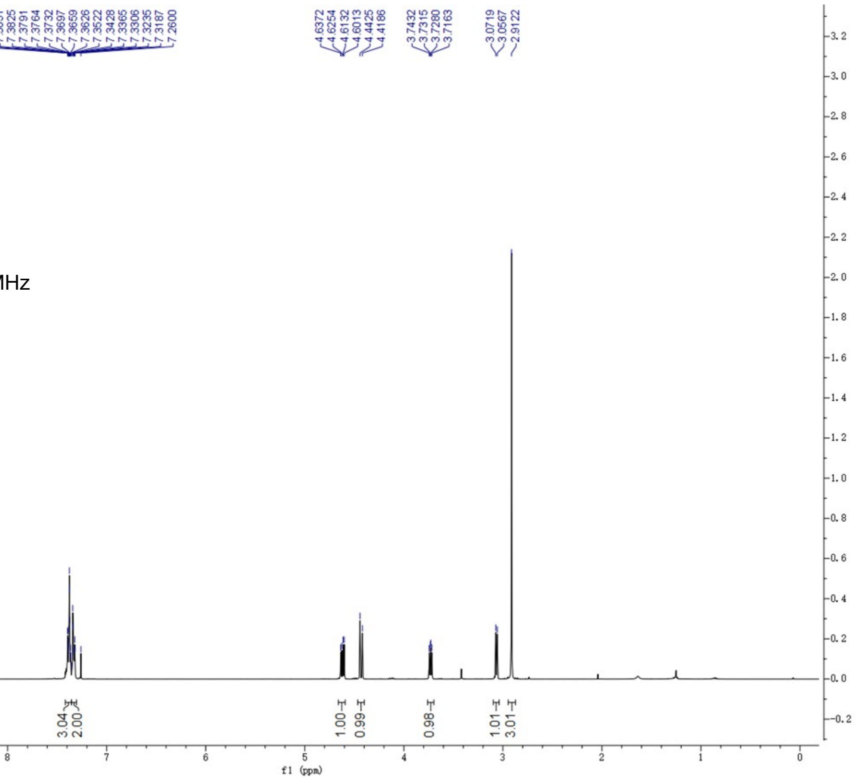

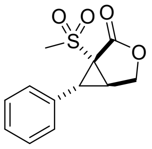

2bm

CDCl$_3$, ^{13}C NMR, 100 MHz

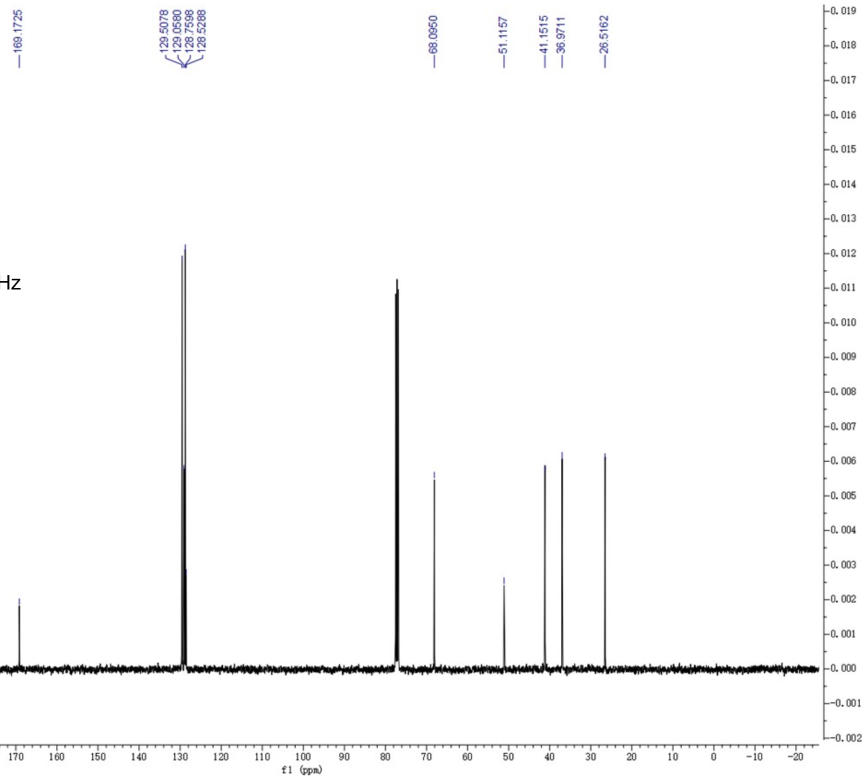

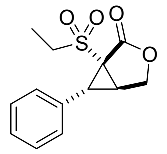

2bn

CDCl$_3$, ^{1}H NMR, 400 MHz

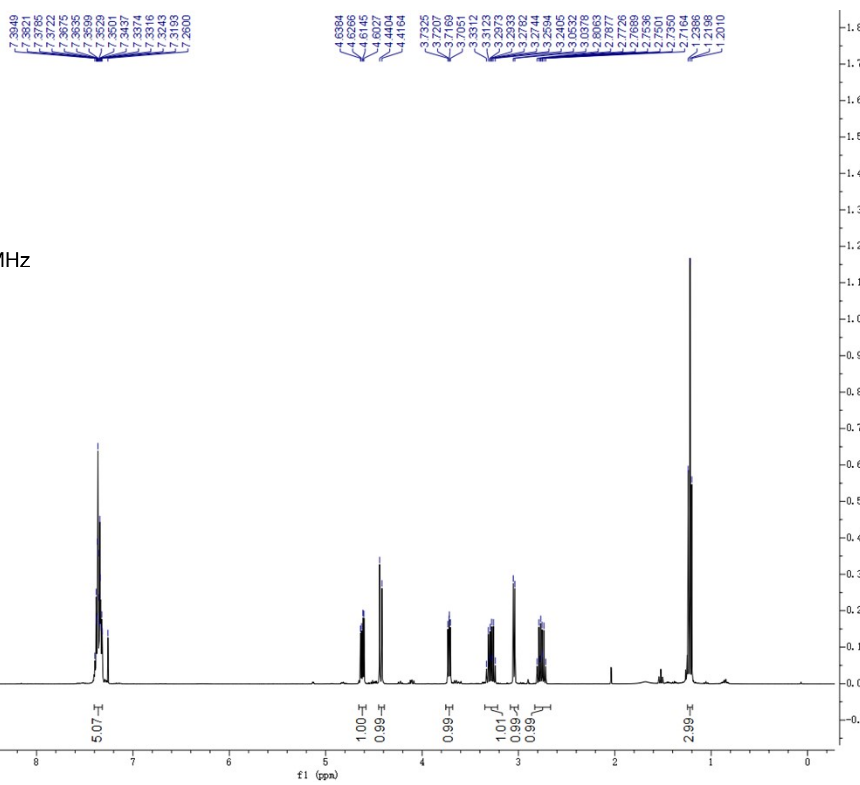

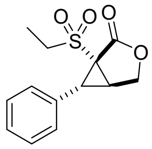

2bn

CDCl$_3$, ^{13}C NMR, 100 MHz

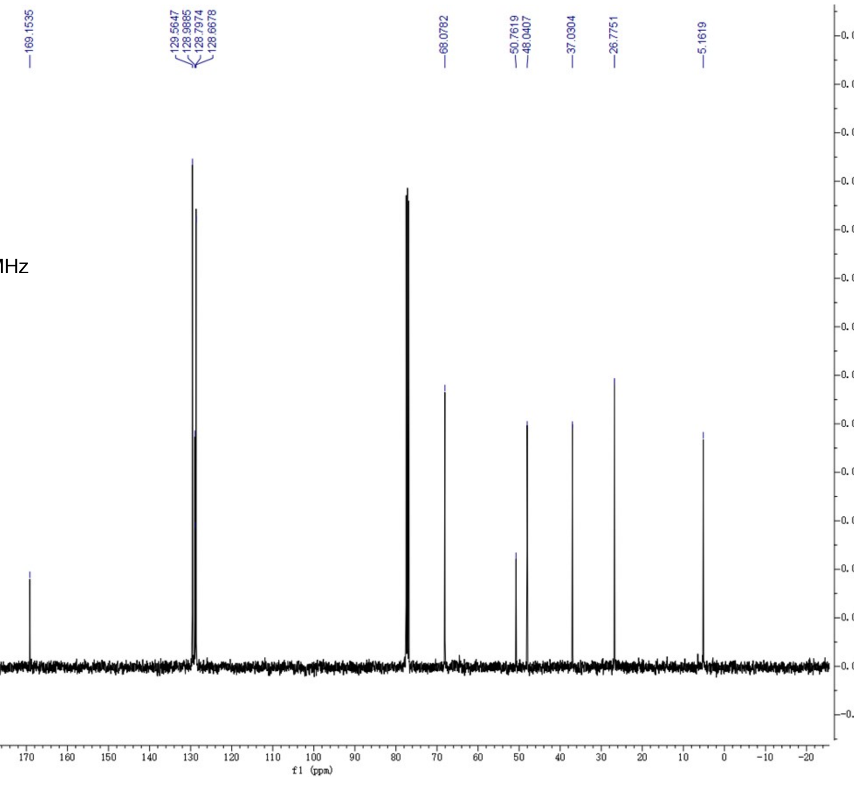

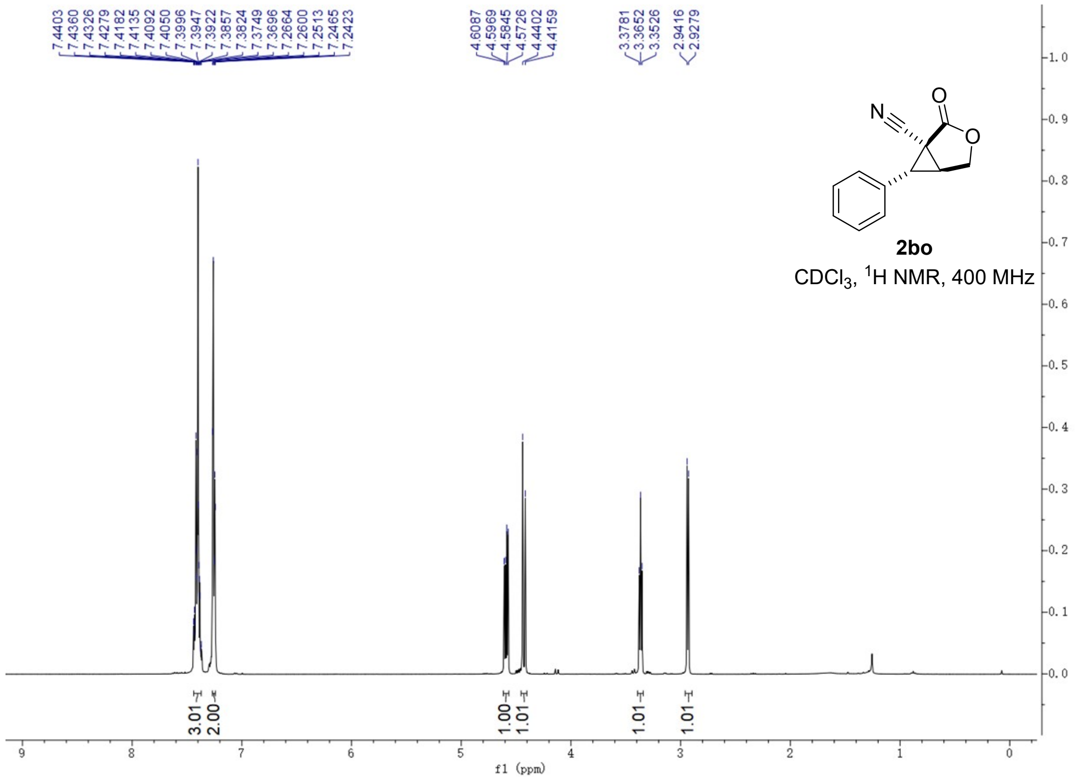
2bo
CDCl3, 1H NMR, 400 MHz
f1 (ppm)

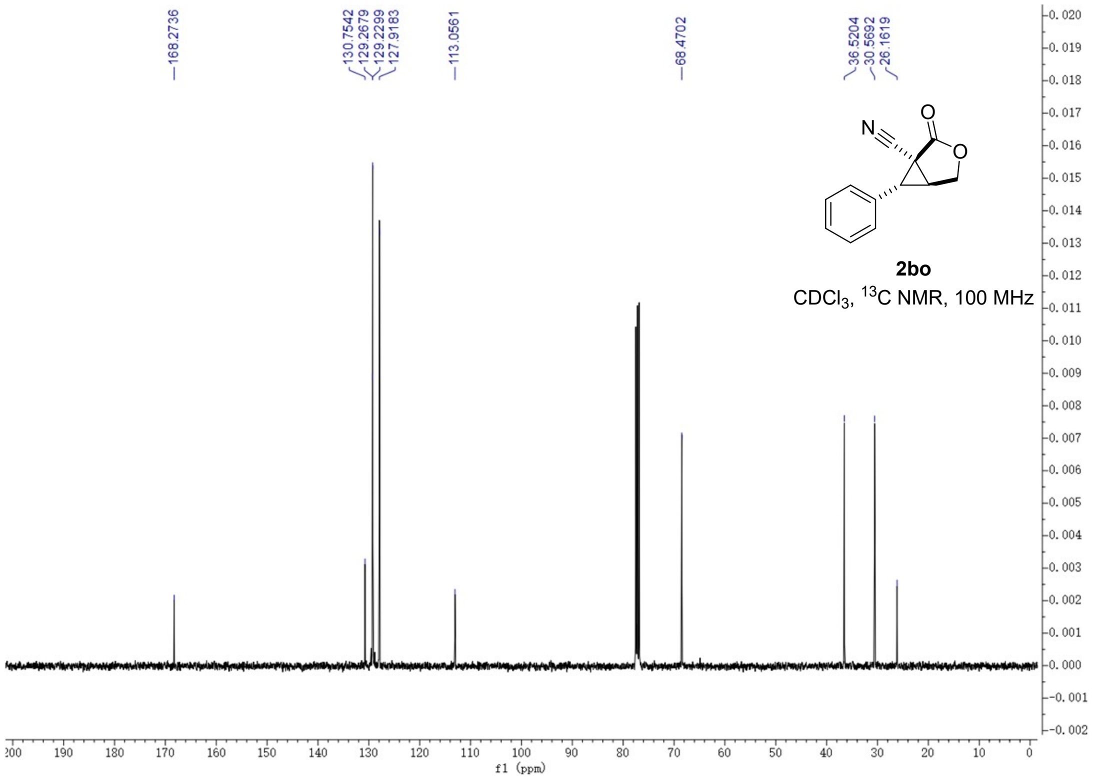
2bo
CDCl3, 13C NMR, 100 MHz
f1 (ppm)

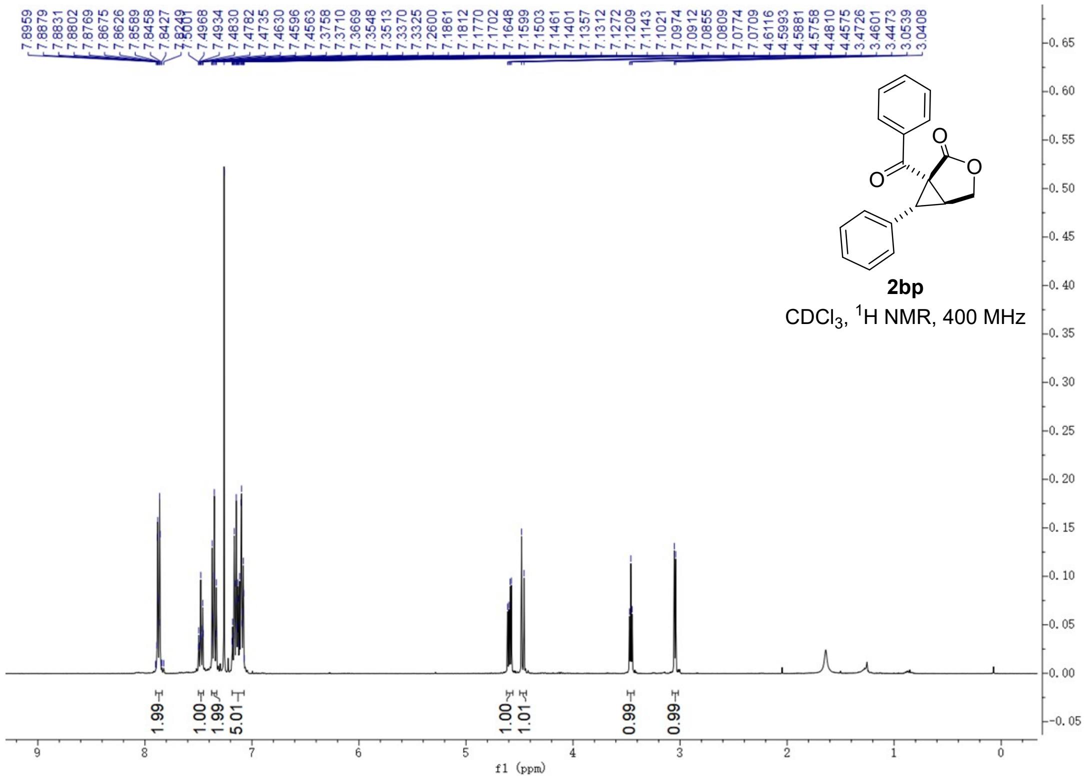
2bp
$CDCl_3$, ^1H NMR, 400 MHz

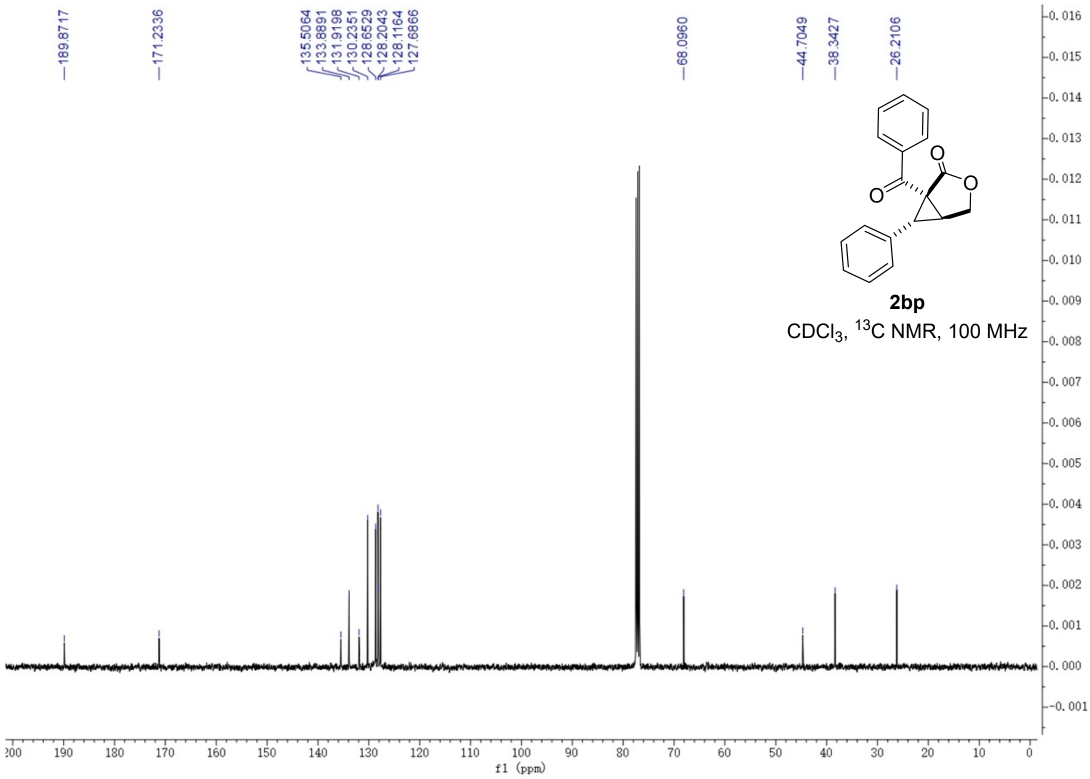
2bp
$CDCl_3$, ^{13}C NMR, 100 MHz

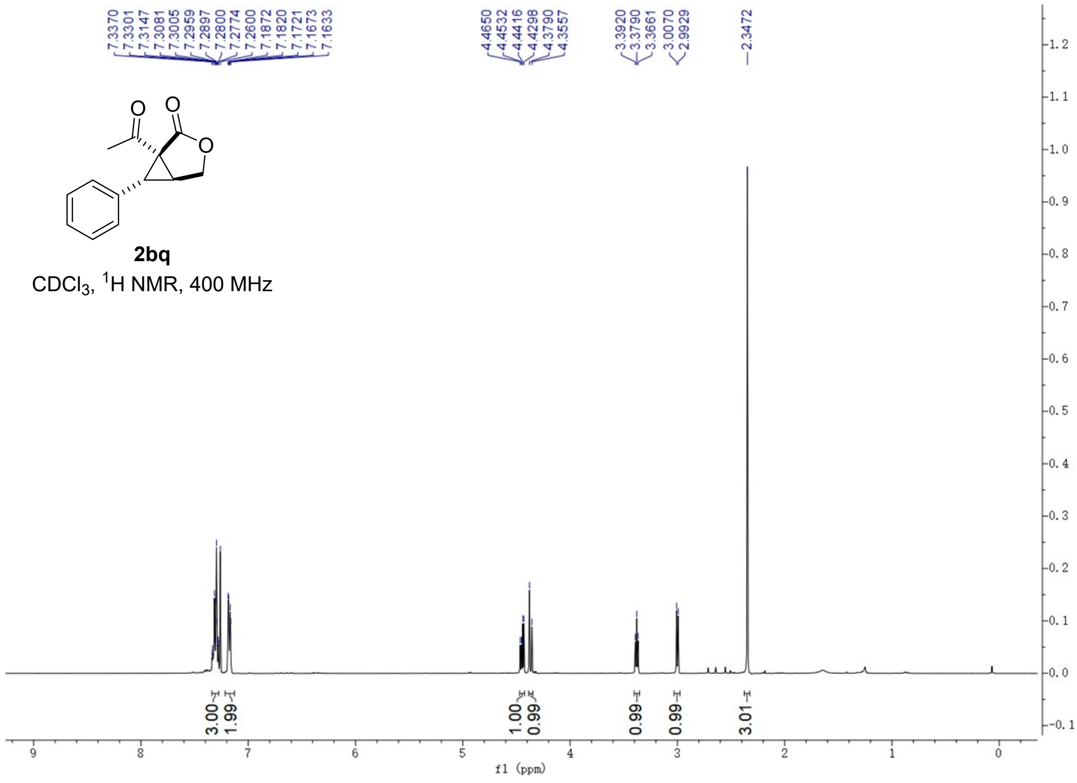

2bq

$CDCl_3$, ^1H NMR, 400 MHz

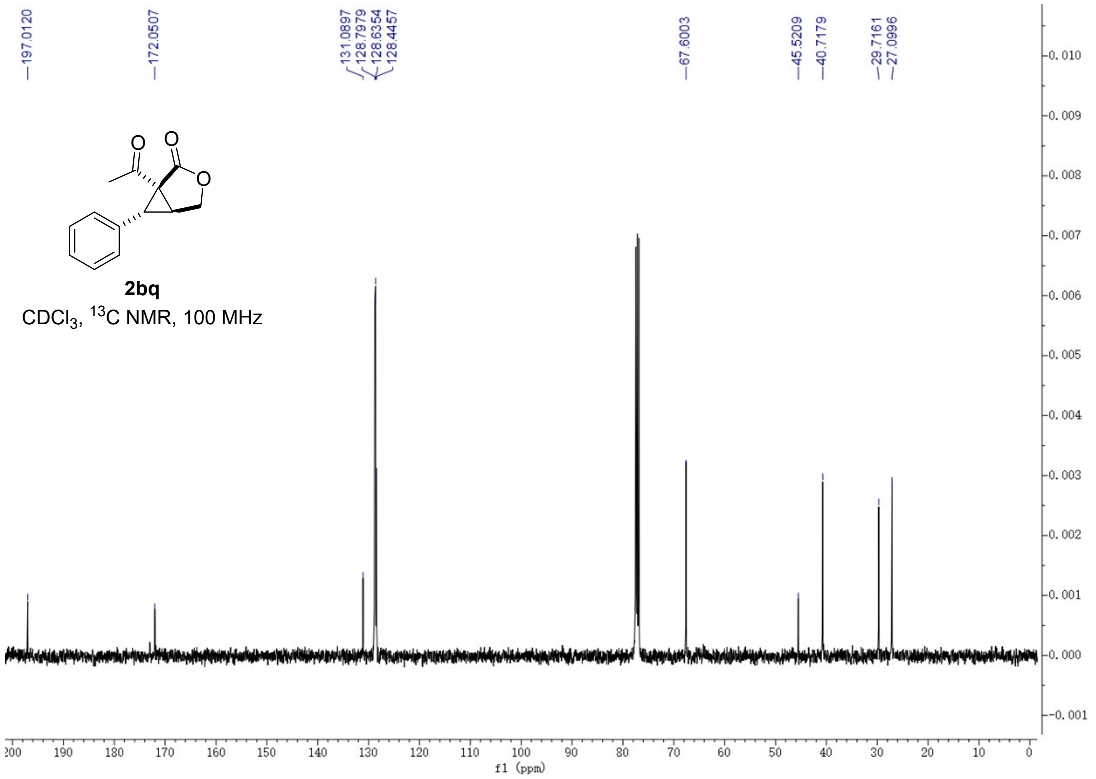

2bq

$CDCl_3$, ^{13}C NMR, 100 MHz

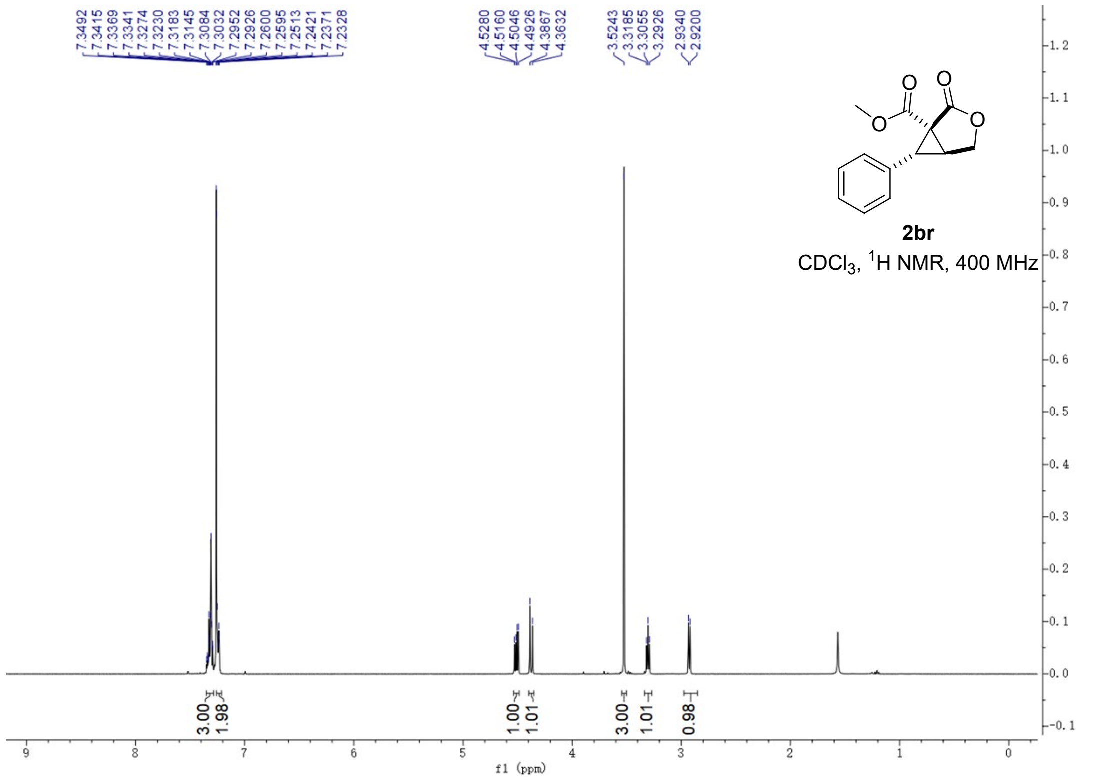
7.3492
7.3415
7.3369
7.3341
7.3274
7.3230
7.3183
7.3145
7.3084
7.3032
7.2952
7.2926
7.2600
7.2595
7.2513
7.2421
7.2371
7.2328
4.5280
4.5160
4.5046
4.4926
4.3867
4.3632
3.5243
3.3185
3.3055
3.2926
2.9340
2.9200
3.00
1.98
1.00
1.01
3.00
1.01
0.98
O
O
O
O
2br
CDCl3, 1H NMR, 400 MHz
f1 (ppm)

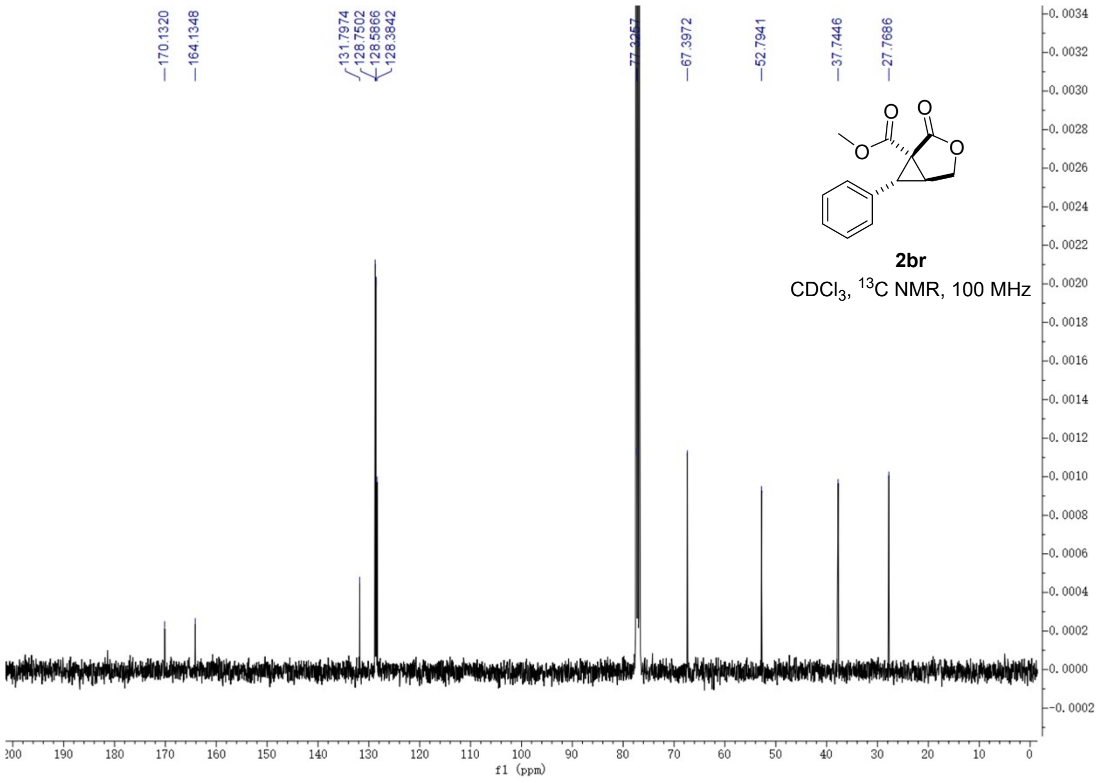
170.1320
164.1348
131.7974
128.7502
128.5866
128.3842
77.3257
67.3972
52.7941
37.7446
27.7686
O
O
O
O
2br
CDCl3, 13C NMR, 100 MHz
f1 (ppm)

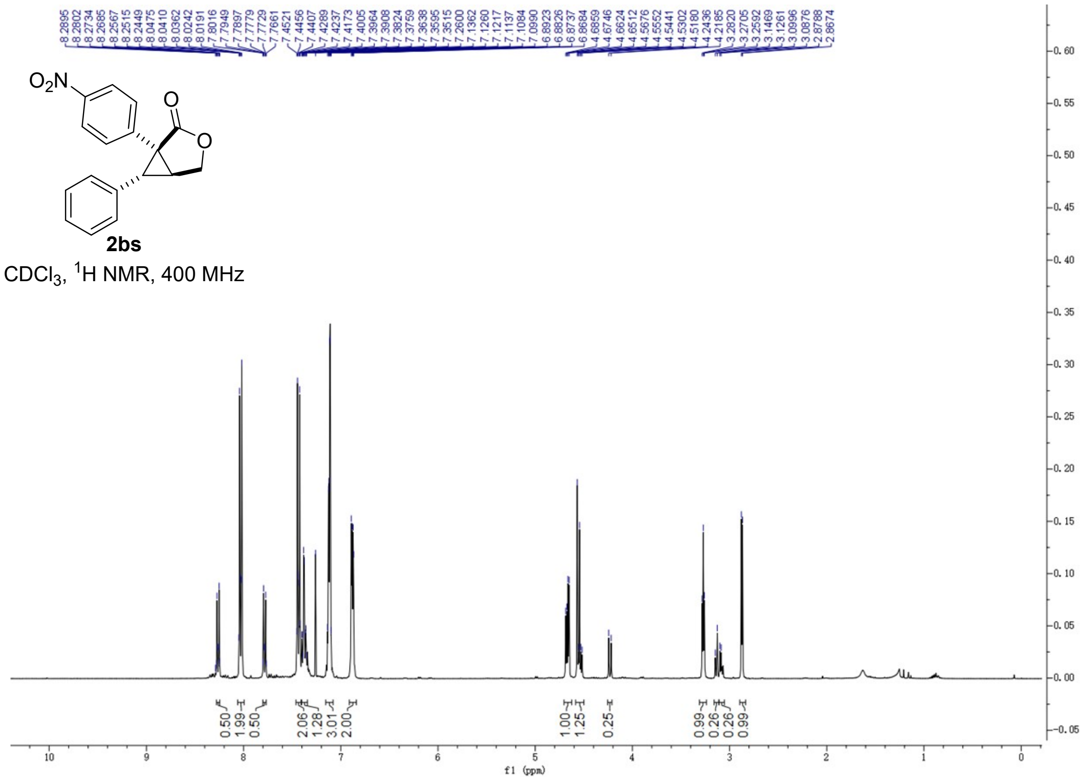
2bs
CDCl$_3$, ^{1}H NMR, 400 MHz
f1 (ppm)

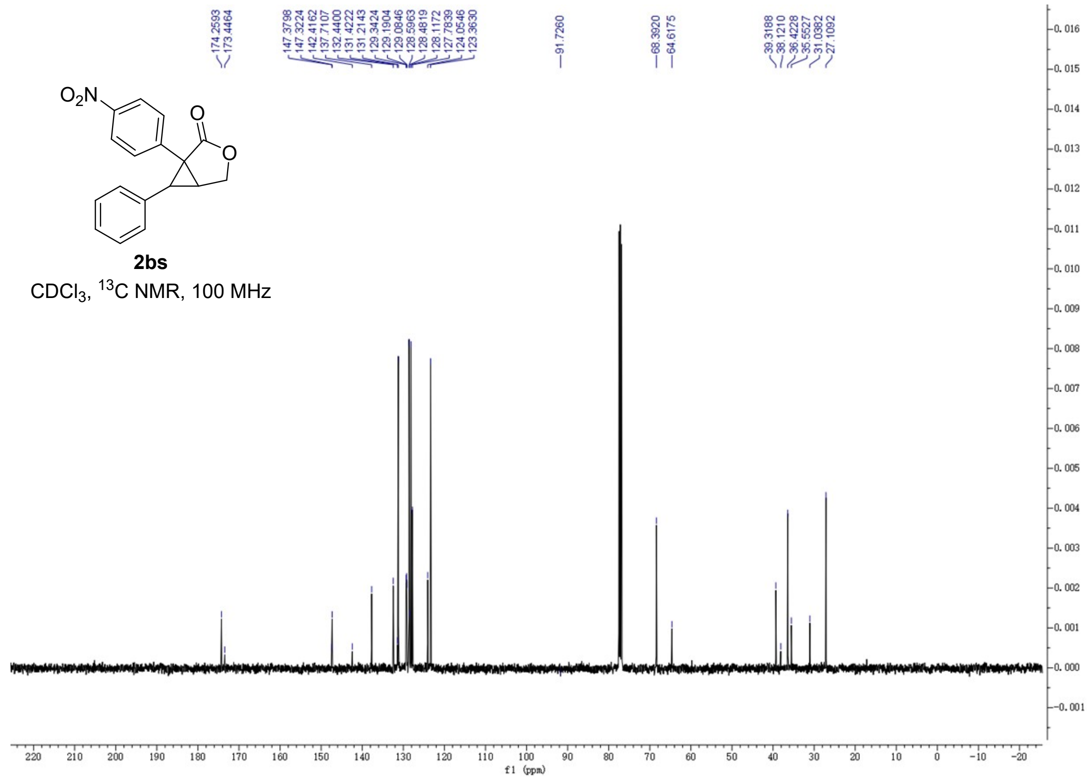
2bs
CDCl$_3$, ^{13}C NMR, 100 MHz
f1 (ppm)

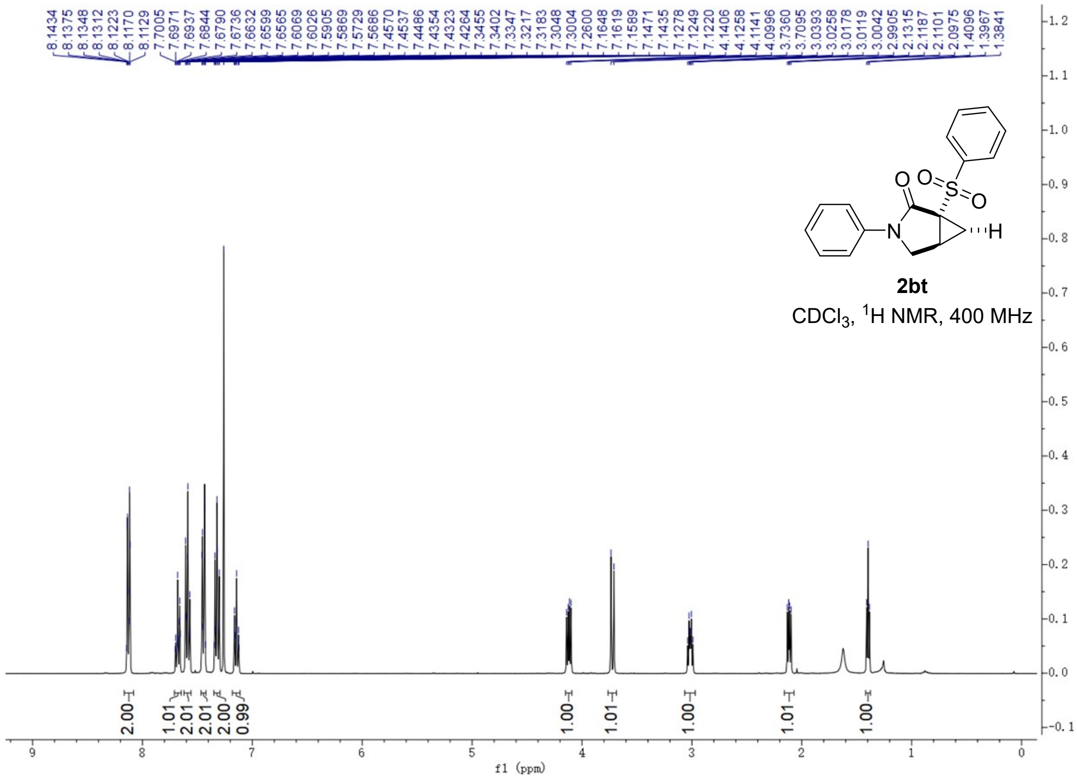

2bt

$CDCl_3$, ^1H NMR, 400 MHz

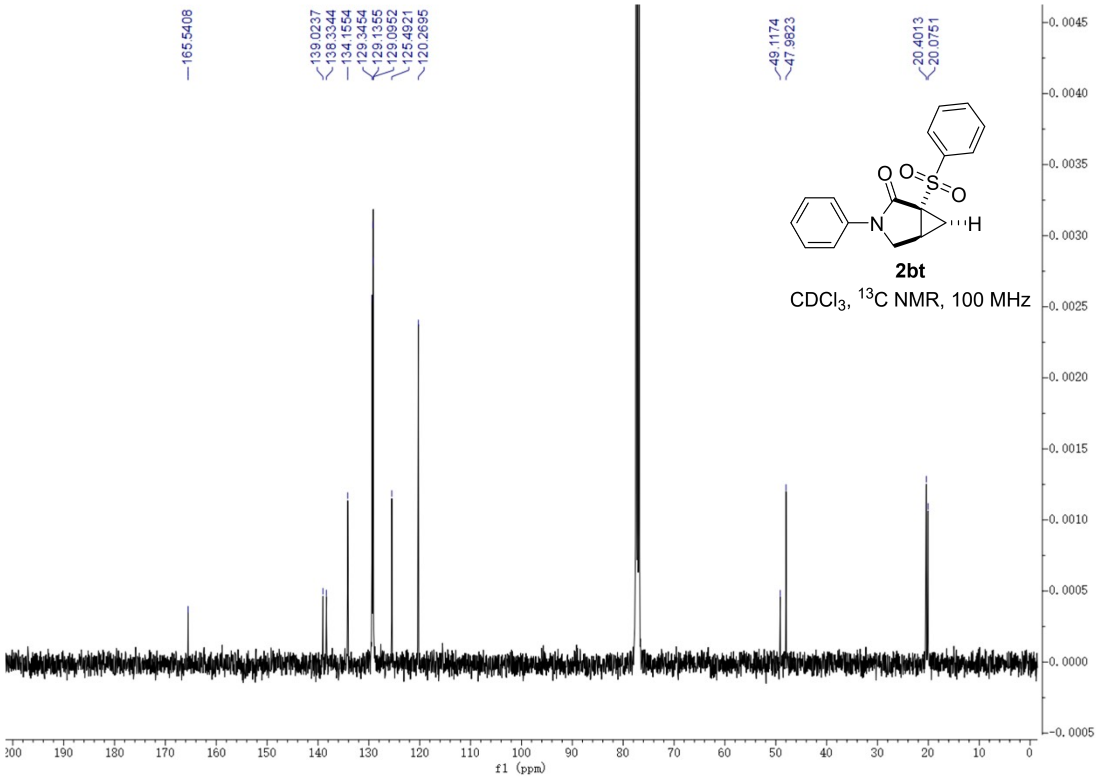

2bt

$CDCl_3$, ^{13}C NMR, 100 MHz

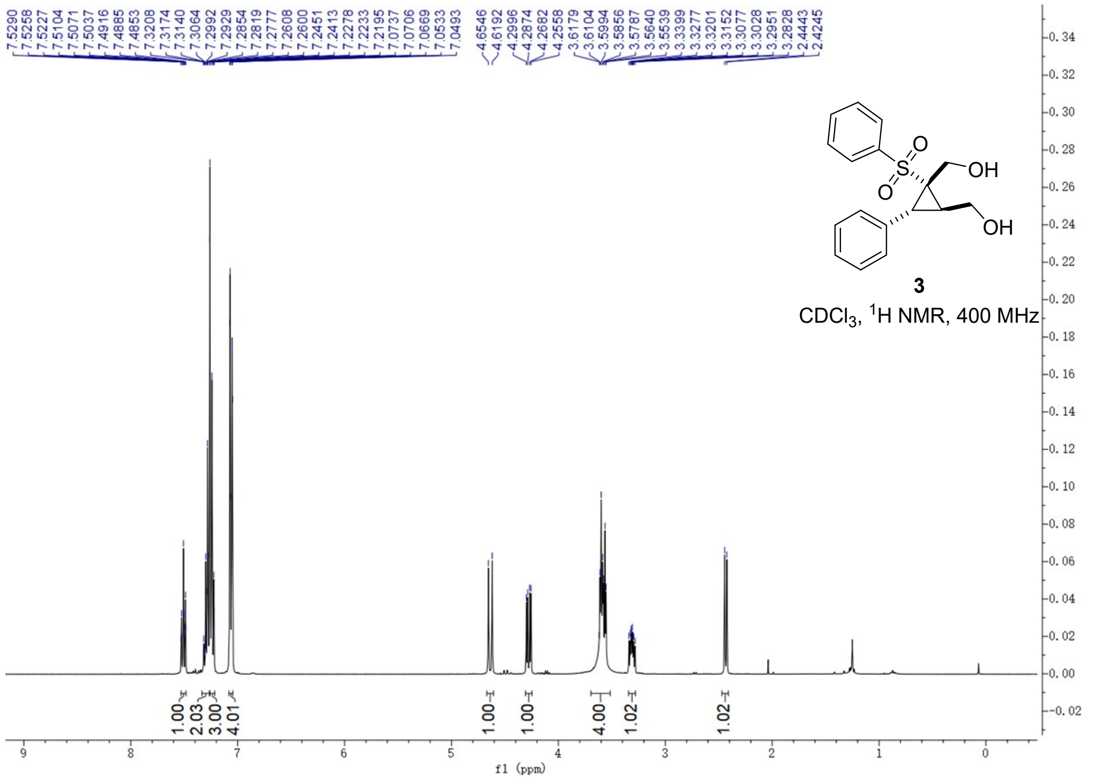
7.5290
7.5258
7.5227
7.5104
7.5071
7.5037
7.4916
7.4885
7.4853
7.3208
7.3174
7.3140
7.3064
7.2992
7.2929
7.2854
7.2819
7.2777
7.2608
7.2600
7.2451
7.2413
7.2278
7.2233
7.2195
7.0737
7.0706
7.0669
7.0533
7.0493
4.6546
4.6192
4.2996
4.2874
4.2682
4.2558
3.6179
3.6104
3.5994
3.5856
3.5787
3.5640
3.5539
3.3399
3.3277
3.3201
3.3152
3.3077
3.3028
3.2951
3.2828
2.4443
2.4245
OH
OH
3
CDCl3, 1H NMR, 400 MHz
1.00
2.03
3.00
4.01
1.00
1.00
4.00
1.02
1.02
f1 (ppm)

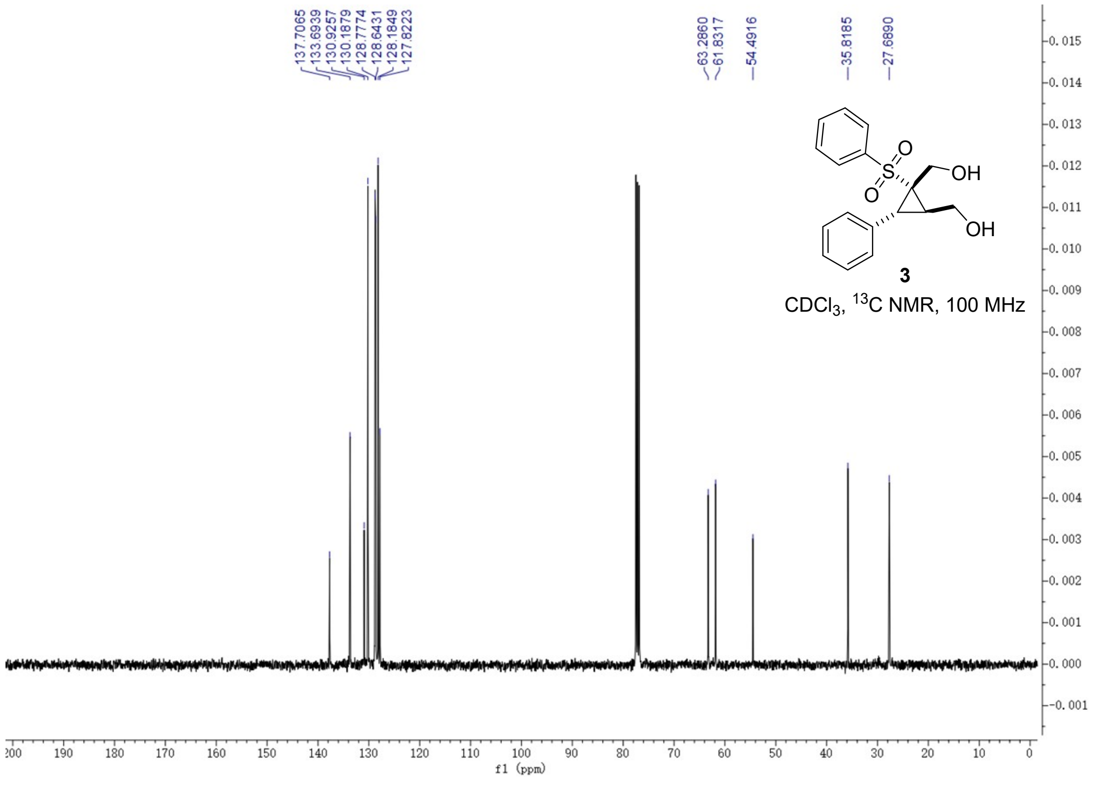
137.7065
133.6939
130.9257
130.1879
128.7774
128.6431
128.1849
127.8223
63.2860
61.8317
54.4916
35.8185
27.6890
OH
OH
3
CDCl3, 13C NMR, 100 MHz
f1 (ppm)

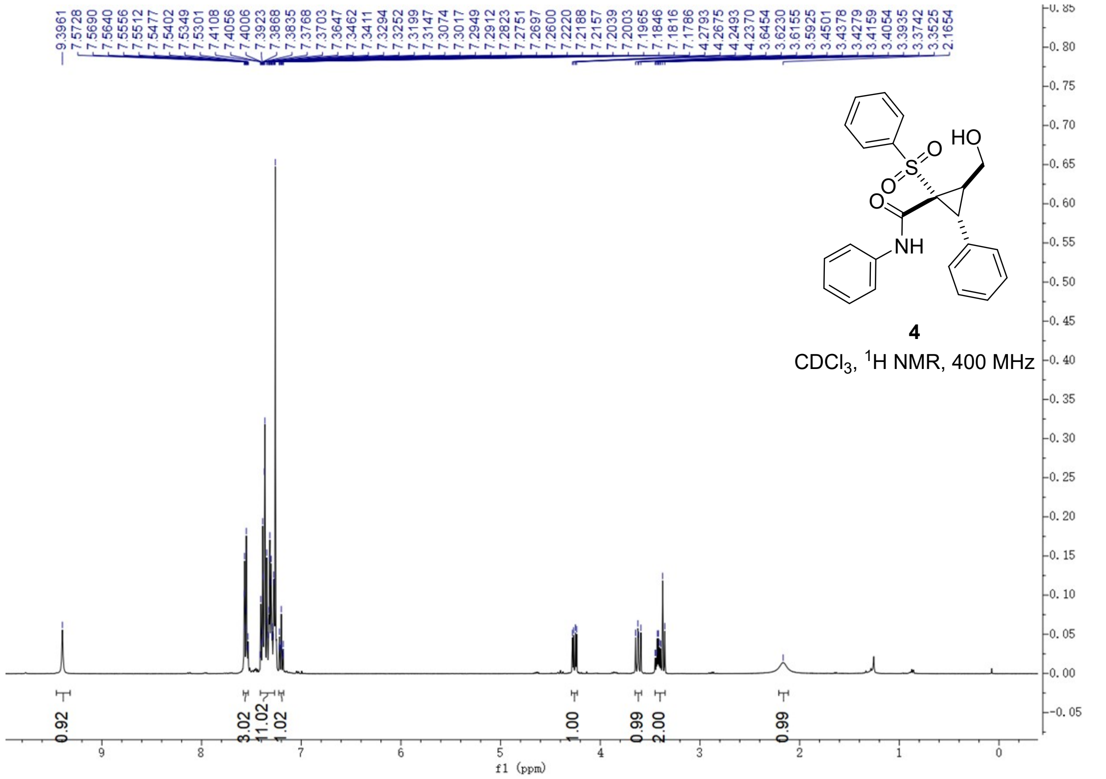
HO
O
S
O
O
NH
4
CDCl3, 1H NMR, 400 MHz
f1 (ppm)

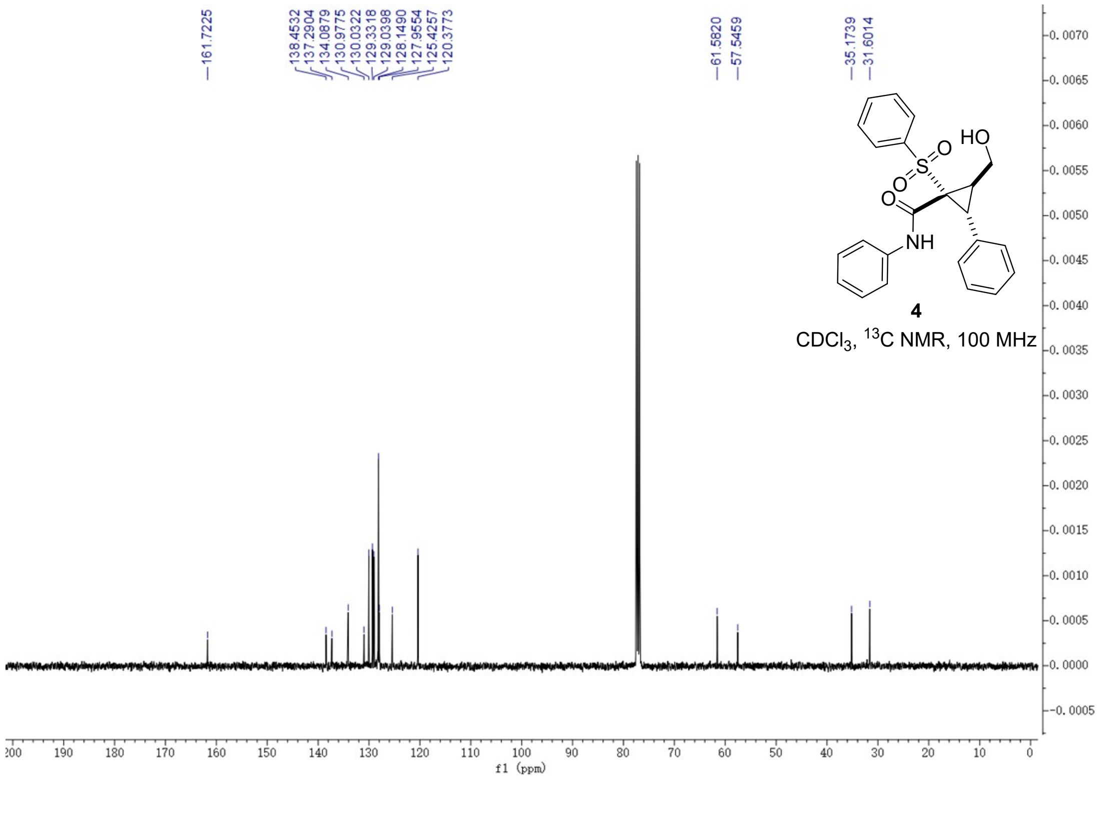
HO
O
S
O
O
NH
4
CDCl3, 13C NMR, 100 MHz
f1 (ppm)

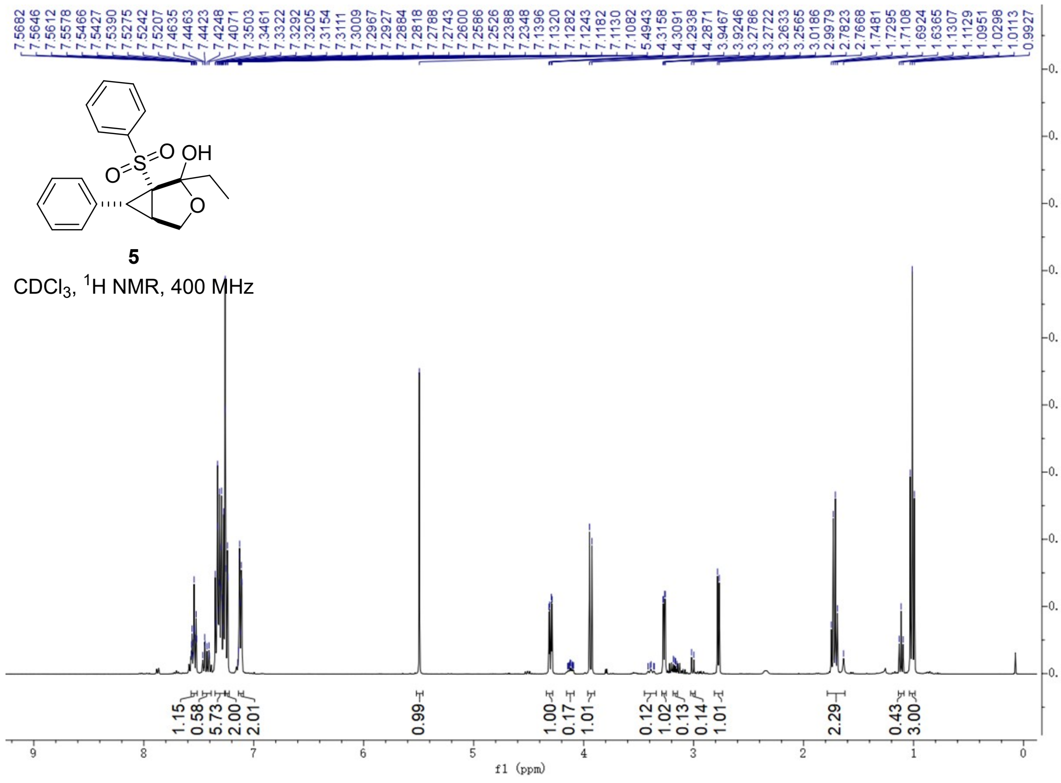

OH
O
O
S
O
5
CDCl3, 1H NMR, 400 MHz
f1 (ppm)

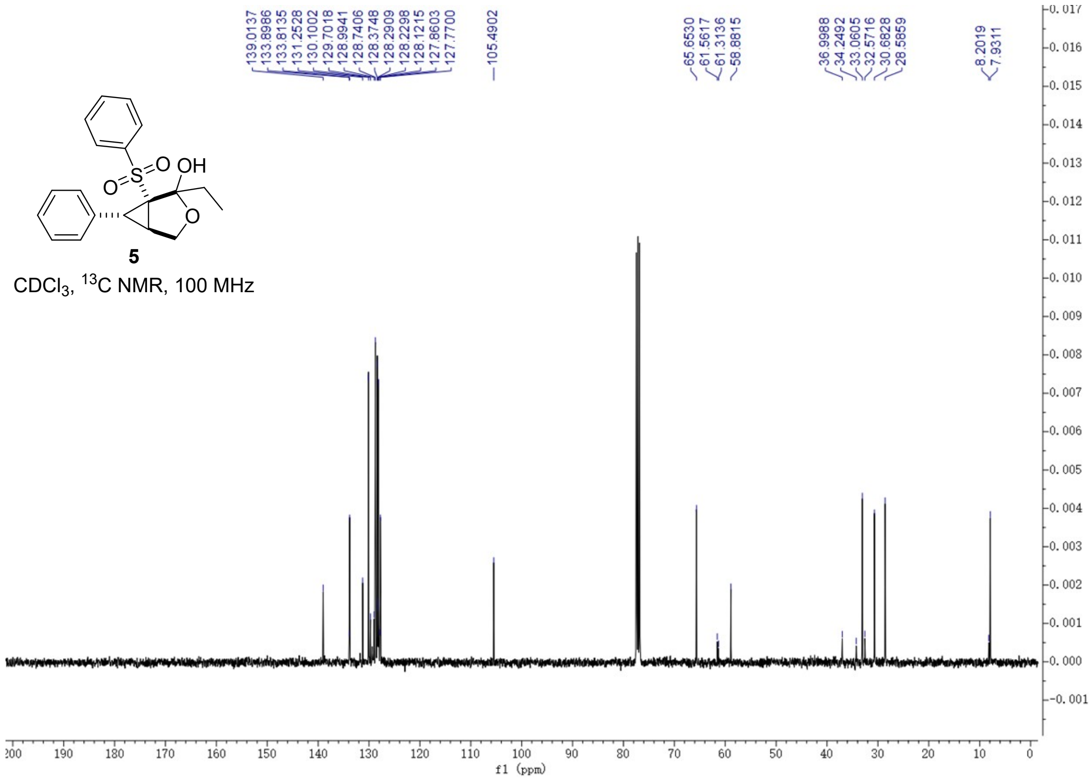

OH
O
O
S
O
5
CDCl3, 13C NMR, 100 MHz
f1 (ppm)

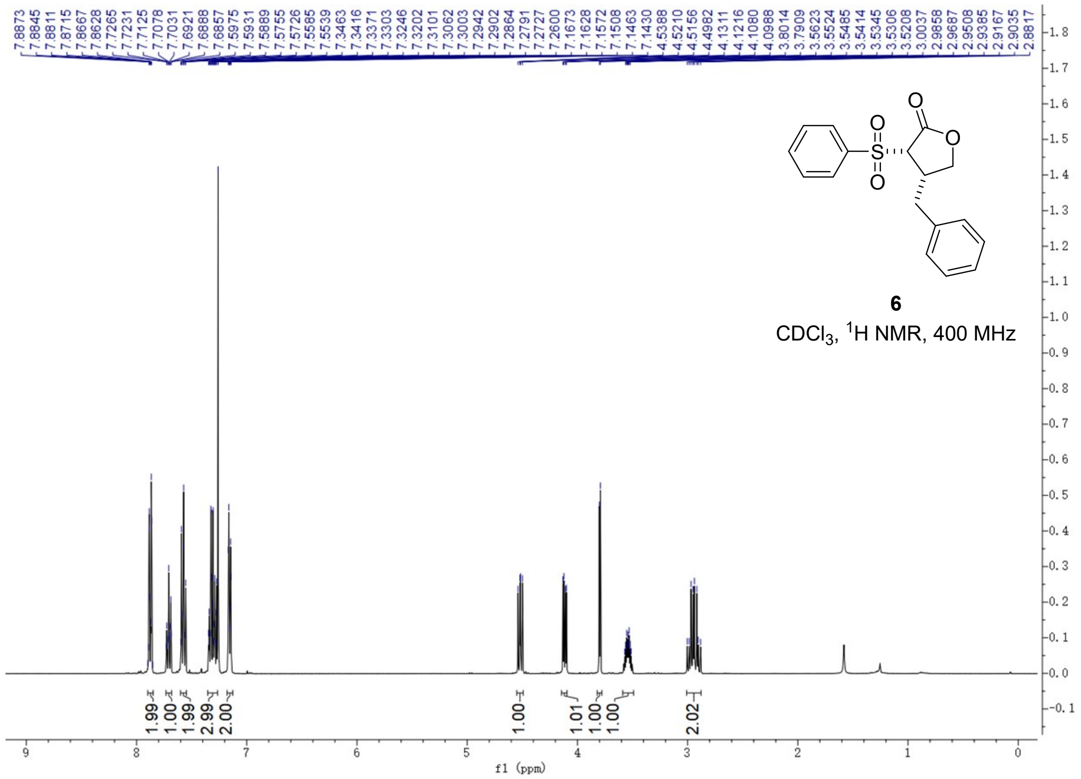
6
$CDCl_3$, ^1H NMR, 400 MHz
f1 (ppm)

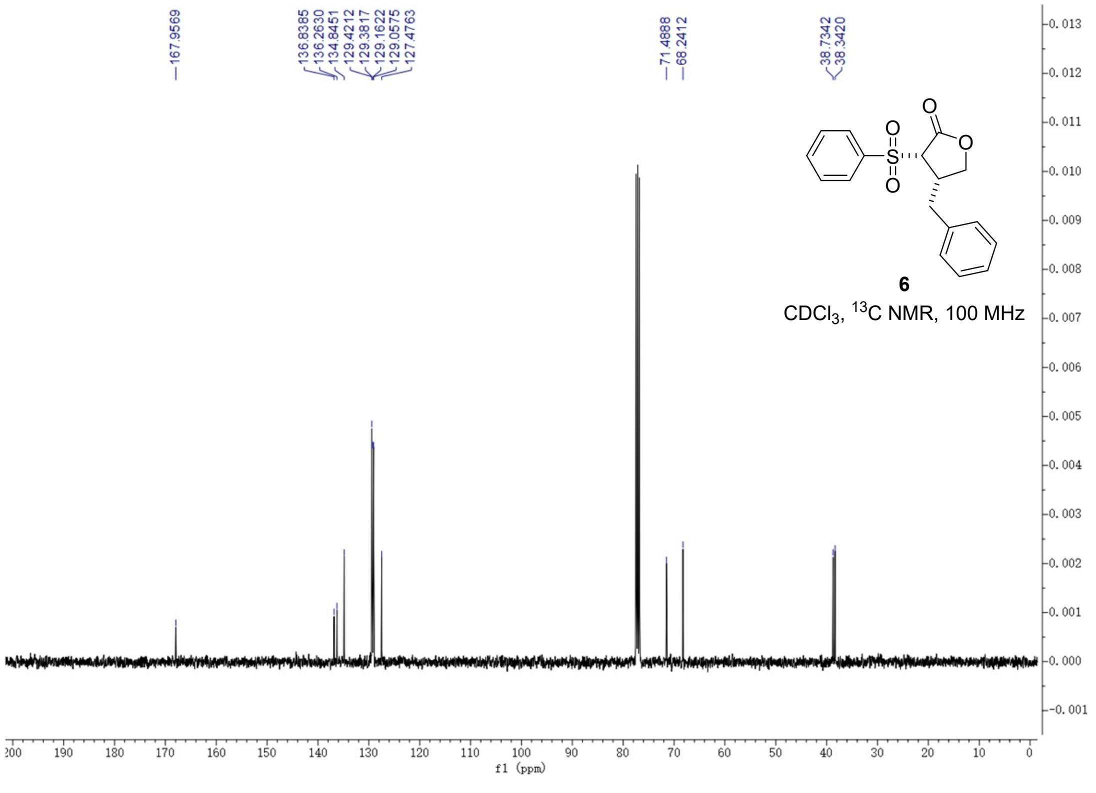
6
$CDCl_3$, ^{13}C NMR, 100 MHz
f1 (ppm)

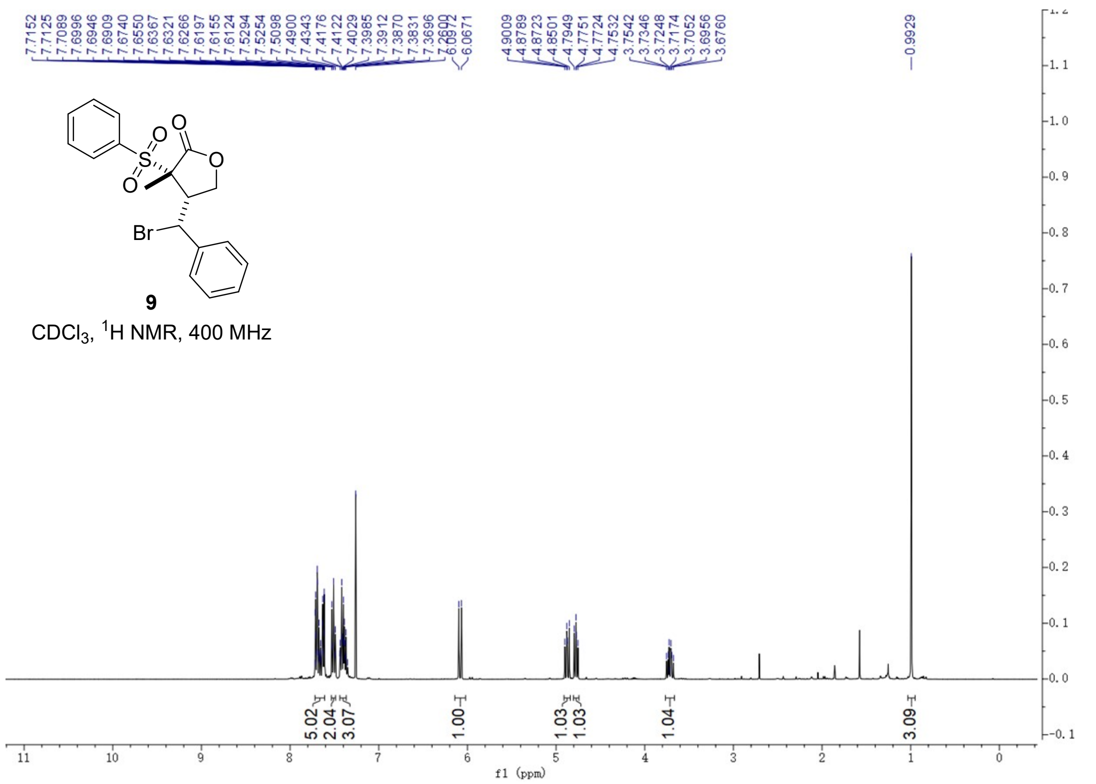

7.7152
7.7125
7.7089
7.6996
7.6946
7.6909
7.6740
7.6550
7.6367
7.6321
7.6266
7.6197
7.6155
7.6124
7.5294
7.5254
7.5098
7.4900
7.4343
7.4176
7.4122
7.4029
7.3985
7.3912
7.3870
7.3831
7.3696
7.2600
6.0872
6.0671
4.9009
4.8789
4.8723
4.8501
4.7949
4.7751
4.7724
4.7532
3.7542
3.7346
3.7248
3.7174
3.7052
3.6956
3.6760
0.9929
Br
9
CDCl3, 1H NMR, 400 MHz
5.02
2.04
3.07
1.00
1.03
1.03
1.04
3.09
f1 (ppm)

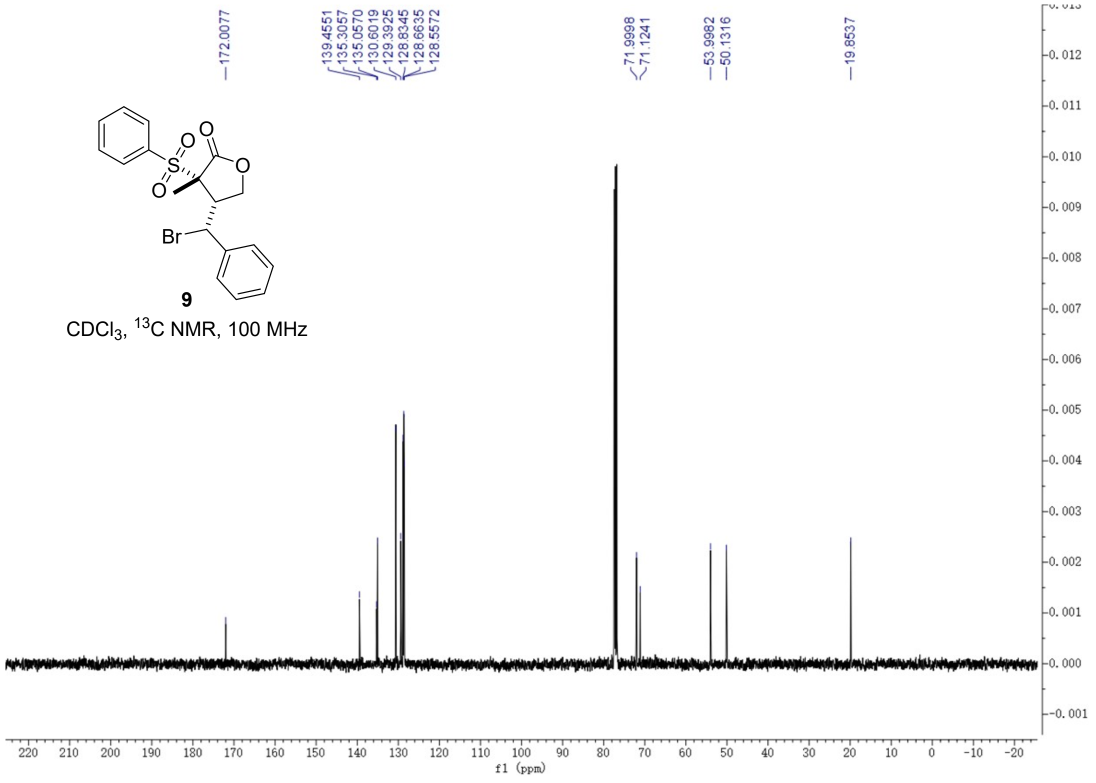

172.0077
139.4551
135.3057
135.0570
130.6019
129.3925
128.8345
128.6635
128.5572
71.9998
71.1241
53.9982
50.1316
19.8537
Br
9
CDCl3, 13C NMR, 100 MHz
f1 (ppm)

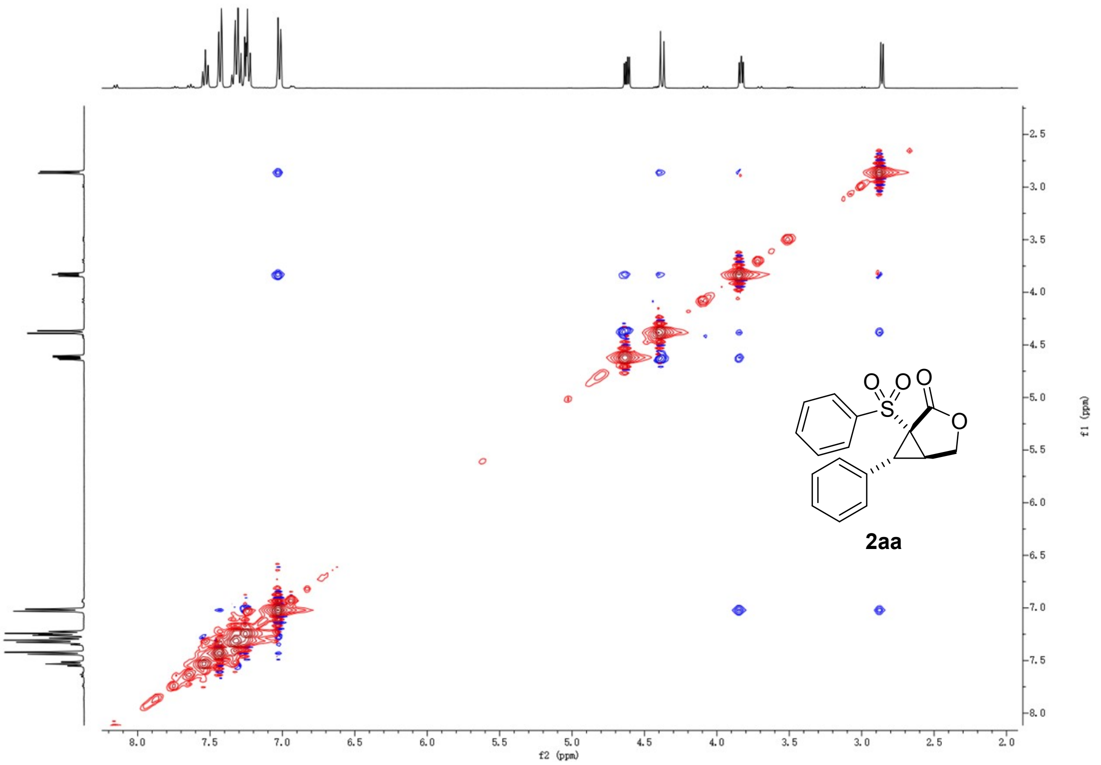

NOESY for compound **2aa**

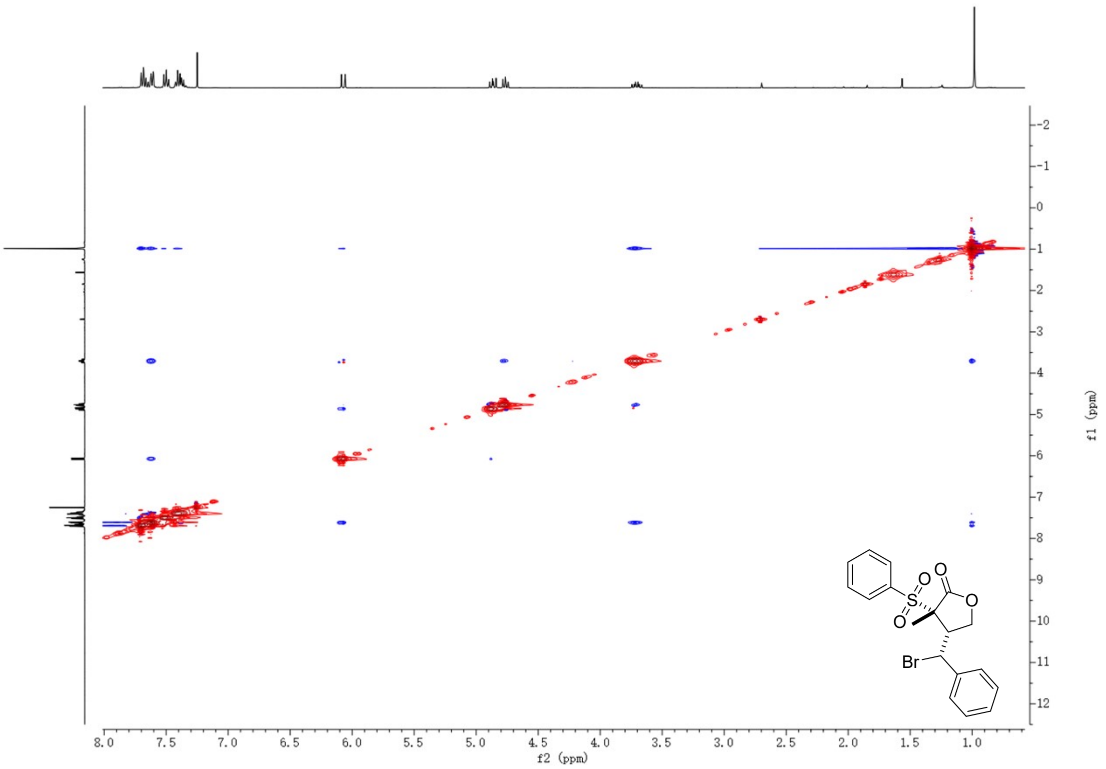

NOESY for compound **9**
